# Biomimetic Synthesis of Seven Halenaquinone Meroterpenoids

**DOI:** 10.1021/jacs.6c06760

**Published:** 2026-05-28

**Authors:** Jacob D. Hart, Jay K. Lawrence, Peter N. Franqui, Douglas R. Perrott, Bin Yi, Yaguang Xi, Christopher J. Sumby, Christopher G. Newton, Jonathan H. George

**Affiliations:** ‡ Department of Chemistry, 1066 The University of Adelaide, Adelaide, South Australia 5005, Australia; § Department of Chemistry, 1355 University of Georgia, Athens, Georgia 30602, United States; ∥ Department of Pharmaceutical and Biomedical Sciences, 1355 University of Georgia, Athens, Georgia 30602, United States; ⊥ Innovations in Drug Discovery (IDD) Program, 1355 University of Georgia, Athens, Georgia 30602, United States; # School of Chemistry and Chemical Engineering, 7423 University of Southampton, Highfield, Southampton SO17 1BJ, United Kingdom

## Abstract

Structurally similar natural products arising in phylogenetically distant organisms may reflect convergent evolutionary pressure toward privileged biological scaffolds. The marine sponge-derived halenaquinone meroterpenoids exemplify this phenomenon, sharing a highly electrophilic and strained diketofuran motif with both the viridin/wortmannin and hibiscone families of natural products. Guided by biosynthetic reasoning, we report herein a biomimetic route to the halenaquinone family. Central to the strategy is an aryne–furan Diels–Alder reaction that rapidly constructs the tetracyclic carbon framework, together with oxidative decarboxylation of a diosphenol intermediate, followed by divergent late-stage oxidative cyclizations to furnish either xestoquinone or halenaquinone. This platform enabled the synthesis of seven natural products, three structural revisions, and the discovery of potent cytotoxic activity for several family members.

## Graphical abstract

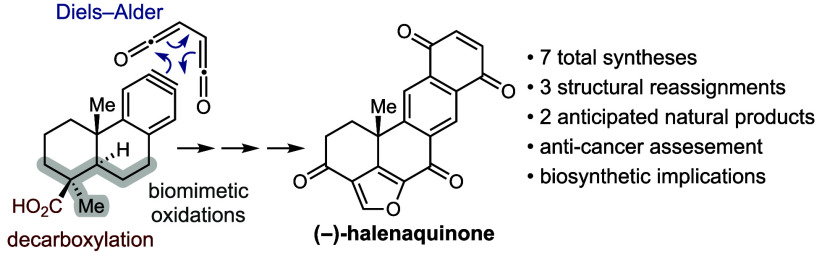

## Introduction

Closely related organisms, such as species within the same genus or family, often harbor conserved biosynthetic gene clusters inherited from a common ancestor, enabling the production of identical or closely related secondary metabolites.^1^ Similar outcomes can also arise through horizontal gene transfer, although such events are most common within related lineages.^2^ In contrast, the occurrence of structurally related natural products in distantly related organisms, particularly when assembled via distinct biosynthetic pathways, is rare.^3^ Given the widely recognized role of secondary metabolites in conferring evolutionary advantages, such instances of “convergent evolution” in natural product biosynthesis may serve as indicators of molecules with significant biological function. Herein, we explore this hypothesis in the context of the viridin, hibiscone, and halenaquinone families of natural products, which share a distinctive structural motif: a strained diketofuran embedded within a decalin framework.

While the pathways to these natural products differ, they appear to follow a common two-phase biosynthetic logic (Figure 1a).^4^ An initial cyclase phase generates a complex polycyclic scaffold incorporating a fused decalin ring system, which is subsequently elaborated in an oxidase phase to install the strained diketofuran motif. For example, in the terrestrial fungus *Gliocladium virens*, the triterpenoid viridin arises from polycyclization of squalene to lanosterol, followed by a sequence of C–H oxidations and oxidative cleavage steps.

**Figure 1:**
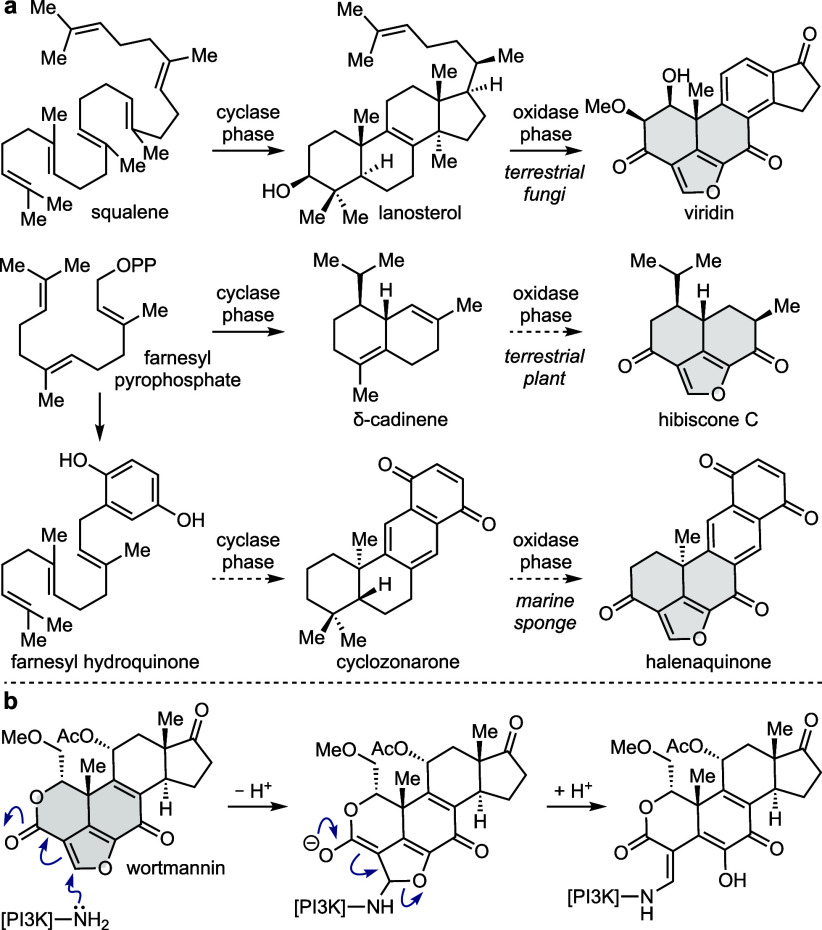
(a) Biosynthetic origins of viridin, hibiscone, and halenaquinone natural products. (b) Mechanism of PI3K inhibition.

Although gene cluster analyses have identified several intermediates linking lanosterol to viridin,^5^ some gaps remain. By contrast, the sesquiterpenoid hibiscone C, isolated from *Hibiscus elatus* and *Gmelina arborea*,^6^ is proposed to derive from oxidative elaboration of δ-cadinene, a common metabolite in related flowering plants,^7^ formed via cyclization of farnesyl pyrophosphate.^8^ Halenaquinone, by comparison, has been isolated from several *Xestospongia* marine sponges collected from Caribbean and Polynesian coral reefs.^9^ As a meroterpenoid, it could arise from cyclization of farnesyl hydroquinone to cyclozonarone,^10^ followed by extensive oxidative tailoring to furnish the final scaffold. Notably, halenaquinone possesses the opposite absolute configuration at the shared tricyclic furanone core relative to viridin and hibiscone.^11^

Viridin and its closely related fungal metabolite wortmannin exhibit nanomolar *in vitro* inhibition of phosphoinositide 3-kinase (PI3K), one of the most commonly activated signaling pathways for human cancers.^12^ Mechanistically, inhibition proceeds via aza-Michael addition of an active-site lysine residue to the strained furan moiety, followed by irreversible C–O bond cleavage (Figure 1b). Comparable activity has been reported for hibiscone C and halenaquinone.^13^ Beyond PI3K inhibition, these natural products display a broad spectrum of biological activities, including antimalarial,^14^ anti-inflammatory,^15^ antifungal,^16^ and anticancer^17^ effects, although detailed structure–activity relationships remain underexplored.

### Meroterpenoid Biosynthesis

Owing to the presence of four carbonyl groups and a furan ring, halenaquinone (**1**) was initially proposed to arise from a purely polyketide pathway.^9^ However, the subsequent identification of a series of structurally related but less highly oxidized *Xestospongia* metabolites^18^ more obviously derived from a mixed polyketide–terpene biosynthetic pathway led to its reclassification as a meroterpenoid.^19^ Although detailed biosynthetic investigations are lacking, extensive isolation efforts reveal a network of marine merosesquiterpenoids connecting cyclozonarone (**2**) to halenaquinone through a series of oxidations (Figure 2a). The 6–6–6–6 ABCD ring system of cyclozonarone, with a characteristic aromatic C ring, is conserved across the halenaquinone family. Oxidation of **2** at the C9 benzylic and C21 methyl positions gives neopetrosiquinone A (**3**), a meroterpenoid isolated from a deep-water *Neopetrosia* sponge.^20^ Further oxidation at C8 and C21 is proposed to yield intermediate **4**, bearing an axial C2 carboxylic acid predisposed to heterolytic decarboxylation,^21^ facilitated by the adjacent diosphenol.^22^ This process generates an enediol intermediate that, upon oxidation and tautomerization, gives a mixture of exocyclic alkene **6** (a proposed natural product) and endocyclic alkene **5**, which is the *Xestospongia* meroterpenoid orhalquinone.^23^ The divergent reactivity of diosphenol tautomers **5** and **6** may underpin the biosynthesis of halenaquinone and the closely related xestoquinone. Thus, epoxidation of the C1–C2 alkene of **6** followed by cyclization and aromatization could generate xestoquinone (**7**).^24^ Alternatively, oxidation of the C2–C3 alkene of **5** could furnish prehalenaquinone (**8**),^25^ which upon further oxidation yields halenaquinone (**1**). Reduced hydroquinone forms of several intermediates in this series, including halenaquinol, xestoquinol, and sulfated derivatives thereof, have also been isolated.^26^ Furthermore, additional *Xestospongia* metabolites lacking the canonical ABCD carbocyclic framework have been reported, including xestoquinolide A (**9**), bearing a strained seven-membered vinyl lactone,^27^ and noelaquinone (**10**), which features an unusual 1,2,4-triazine ring system.^28^ The deviation of **9** and **10** from meroterpenoid biosynthetic logic encouraged reanalysis of these putative natural product structures.

**Figure 2:**
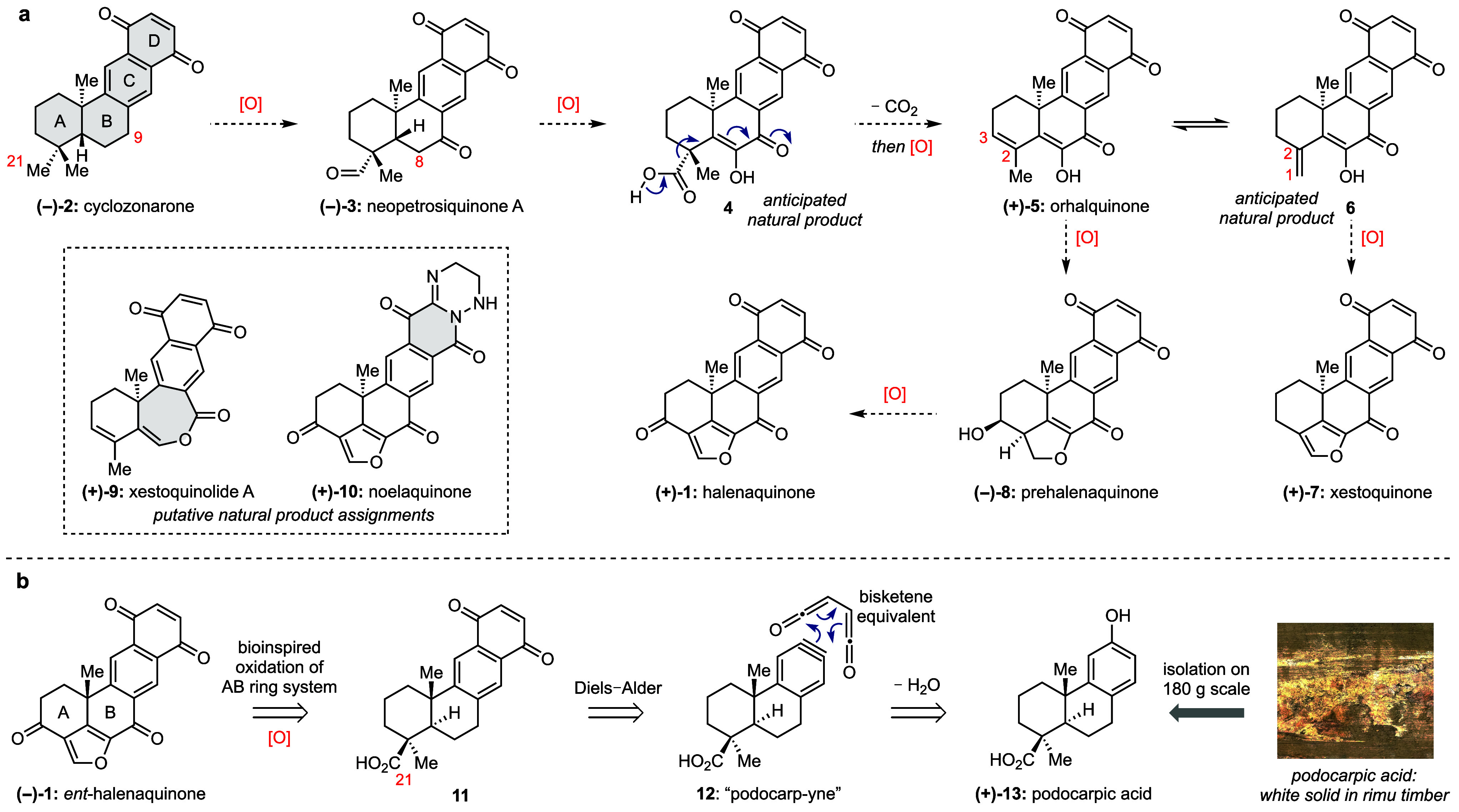
(a) Detailed biosynthetic proposal for halenaquinone and related meroterpenoids via sequential oxidation of cyclozonarone. (b) Bioinspired retrosynthetic analysis of *ent*-halenaquinone enabled by a Diels–Alder disconnection to “podocarp-yne”.

### Retrosynthetic Analysis

Guided by this proposal, we envisioned an efficient total synthesis of halenaquinone based on a sequence of predisposed oxidations of the terpene framework. Such a biomimetic approach would also enable chemical interrogation of the proposed pathway, allowing the inherent reactivity of putative intermediates to be probed and rationalizing the origin of the strained diketofuran warhead. Recognizing that oxidation of the unactivated C21 methyl group would pose a significant hurdle, we initially targeted the synthesis of *ent*-halenaquinone (−)-**1** from C21 carboxylic acid **11** (Figure 2b). This intermediate was envisioned to arise from an intermolecular Diels–Alder reaction between “podocarp-yne” **12** and a suitable bisketene equivalent. Benzyne intermediate **12** could, in turn, be accessed by formal dehydration of (+)-podocarpic acid **13**, a readily available terpene natural product that accumulates in timber from the New Zealand rimu tree, *Dacrydium cupressinum* (see the Supporting Information for a 180 g scale isolation).^29^

## Results and Discussion

### Biomimetic Synthesis of the Halenaquinone Family

Application of our retrosynthetic analysis to the total synthesis of the halenaquinone family is summarized in Figure 3. Aryne precursor selection was guided by our prior work in the furan/aryne Diels–Alder space.^30^ Accordingly, podocarpic acid **13** was advanced to iodotriflate **14** through a high-yielding, one-pot, *ortho*-iodination/phenol triflation protocol, for which both the base and triflation reagent proved critical (see the Supporting Information for more details). Subsequent treatment of **14** with excess *n*-butyllithium at a low temperature in the presence of 2-methoxyfuran effected aryne generation and concomitant Diels–Alder cycloaddition. *In situ* acid-mediated cleavage of the resulting oxa-bridge was followed by global methylation to furnish annulated intermediate **15**, thereby generating the complete ABCD tetracyclic carbon framework of the halenaquinone family on a gram scale in only two steps.

**Figure 3:**
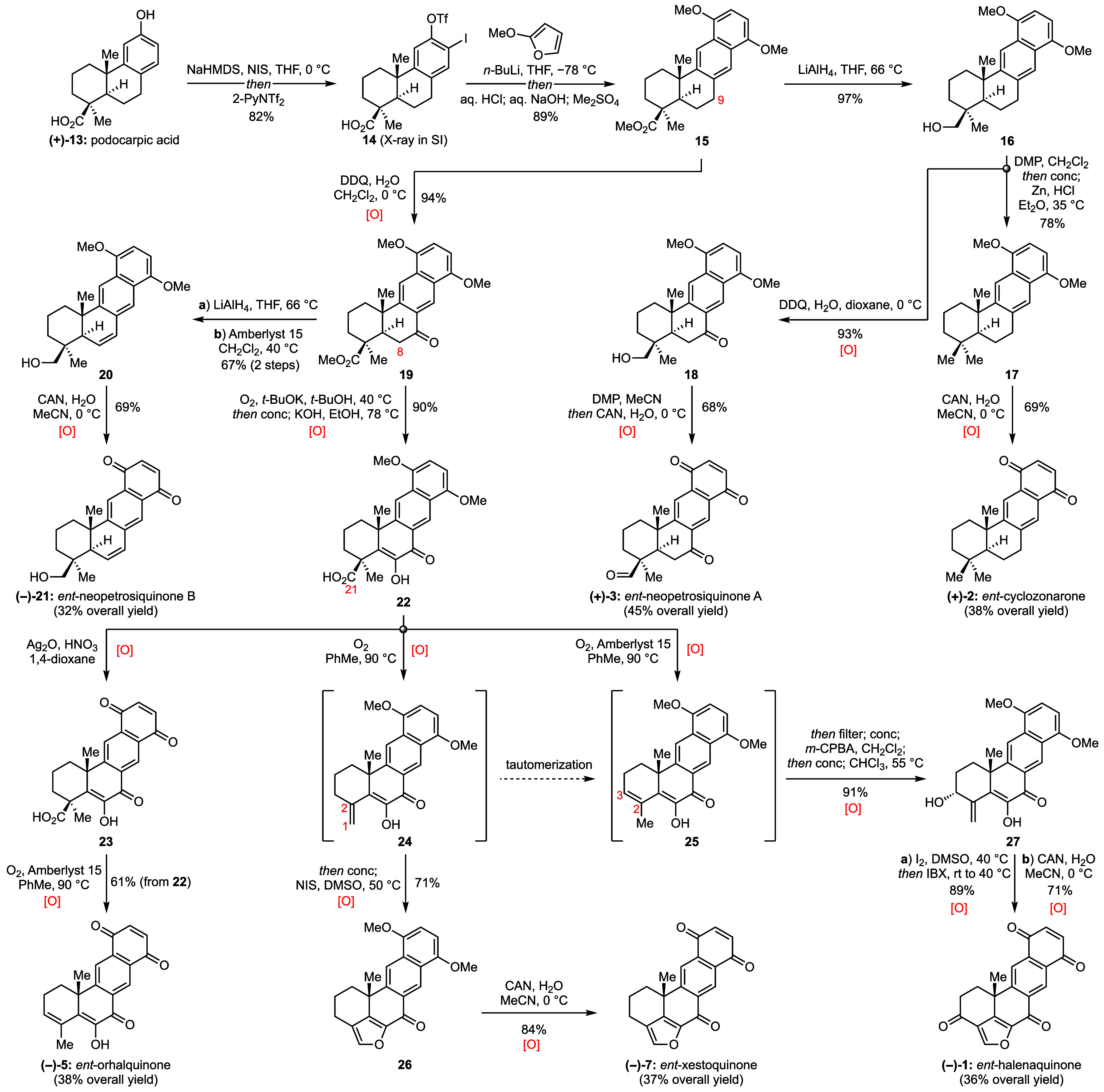
Collective synthesis of cyclozonarone, neopetrosiquinones A and B, orhalquinone, xestoquinone, and halenaquinone.

The three simplest natural product targets, namely, cyclozonarone and neopetrosiquinones A and B, were accessed in short order via a series of carefully orchestrated redox manipulations. In the case of cyclozonarone, reduction of the ester present in **15** furnished primary alcohol **16**, which was then subjected to a Clemmensen-based deoxygenation protocol to install the *gem*-dimethyl fragment in **17**. CAN-promoted deprotection then delivered cyclozonarone (**2**).^31^ Alcohol **16** also served as a precursor to neopetrosiquinone A (**3**). Here, a DDQ-mediated benzylic oxidation^32^ furnished ketone **18**, which was converted to the natural product via a one-pot alcohol oxidation/deprotection procedure. In contrast, neopetrosiquinone B was obtained by reversing the order of the redox operations. Specifically, benzylic oxidation of **15** gave ketone **19**, which upon exposure to excess lithium aluminum hydride afforded the corresponding diol (not shown). Acid-promoted elimination of the benzylic alcohol provided olefin **20**, which was oxidatively deprotected to furnish neopetrosiquinone B (**21**).^33^

Installation of the requisite diosphenol motif in key intermediate **22** was achieved via a high-yielding and chemoselective aerobic oxidation of the enolate derived from **19**,^34^ followed by solvent exchange and ester saponification. Access to the remaining targets hinged upon the selective oxidative decarboxylation of **22**. Remarkably, this process occurred spontaneously at room temperature. While decarboxylation was slow and unselective under these conditions, we found that simply heating in toluene under an oxygen atmosphere cleanly promoted the desired conversion to exocyclic olefin **24** in a high yield. Notably, attempts to purify this compound by flash chromatography usually resulted in some isomerization of disubstituted olefin into the A ring, generating protected orhalquinone **25**. This acid-catalyzed tautomerization could be achieved more efficiently by conducting aerobic decarboxylation of **22** under acidic conditions. Direct oxidation of **25** to orhalquinone proved low yielding, but reversing the sequence overcame this limitation: oxidative deprotection of **22** yielded quinone **23**, which upon aerobic decarboxylation in the presence of acid furnished orhalquinone (**5**), marking its first total synthesis. Because of its sensitivity, further elaboration of orhalquinone to xestoquinone or halenaquinone was not extensively pursued.

Despite several individual syntheses of xestoquinone and halenaquinone (comprehensively reviewed in the Supporting Information), there are no reports of a late-stage intermediate serving as a precursor to both natural products. This can be traced to the sensitivity of the highly reactive diketofuran Michael acceptor embedded in halenaquinone, which thwarted its preparation via benzylic oxidation of the furan moiety of xestoquinone. However, selective access to exocyclic alkene **24** and endocyclic alkene **25** via aerobic decarboxylation of **22** mirrors the key branch point in our proposed biosynthesis of xestoquinone and halenaquinone, enabling divergent syntheses of the natural products. Thus, oxidative cyclization of exocyclic alkene **24** to give the corresponding furan of protected xestoquinone **26** was achieved using a modified iodine/DMSO protocol,^35^ with the reaction presumably occurring via iodonium formation, followed by cyclization and elimination. Standard oxidative deprotection conditions then yielded xestoquinone (**7**).^36^ Next, we reasoned that access to the corresponding C3-allylic alcohol derivative of intermediate **24** should enable a similar iodine-promoted cyclization to give halenaquinone. While direct allylic oxidation of **24** was unproductive, we experienced success via epoxidation of the more stable endocyclic olefin isomer **25**, followed by epoxide ring opening to give **27**. Our optimized sequence proceeds via aerobic decarboxylation of **22** in the presence of Amberlyst 15, followed by filtration of the acidic solid support, solvent exchange, and addition of *m*-CPBA to generate a highly sensitive epoxide intermediate. A final solvent switch, followed by warming in chloroform, led to the desired allylic alcohol **27**. Overall, this three-transformation sequence avoids the isolation of unstable intermediates and requires only one chromatographic purification. Finally, the same iodonium-mediated oxidative cyclization for furan formation worked efficiently on allylic alcohol **27**, which was telescoped with an IBX oxidation of the newly benzylic alcohol at C3 to generate protected halenaquinone, and oxidative deprotection gave natural product **1**.^37^

### Natural Product Structural Reassignments

With access to nearly the entire meroterpenoid pathway to halenaquinone, we were positioned to re-evaluate the biosynthetically questionable structures assigned to xestoquinolide A and noelaquinone. First, we noticed that the NMR data for synthetic *ent*-orhalquinone (−)-**5** showed excellent agreement with those reported for both natural orhalquinone (+)-**5** (isolated from an unidentified *Xestospongia* sp. by Bourguet-Kondracki and co-workers^23^) and natural xestoquinolide A (+)-**9** (isolated from *Xestospongia* cf. *carbonaria* by Crews and co-workers^27^), indicating that these natural products are identical (Figure 4a). The presence of an exchangeable proton at δ_H_ 7.09 ppm lacking a HSQC correlation supports assignment of an enolic OH at C8 in **5** rather than the proposed enol lactone structure of **9**.

**Figure 4:**
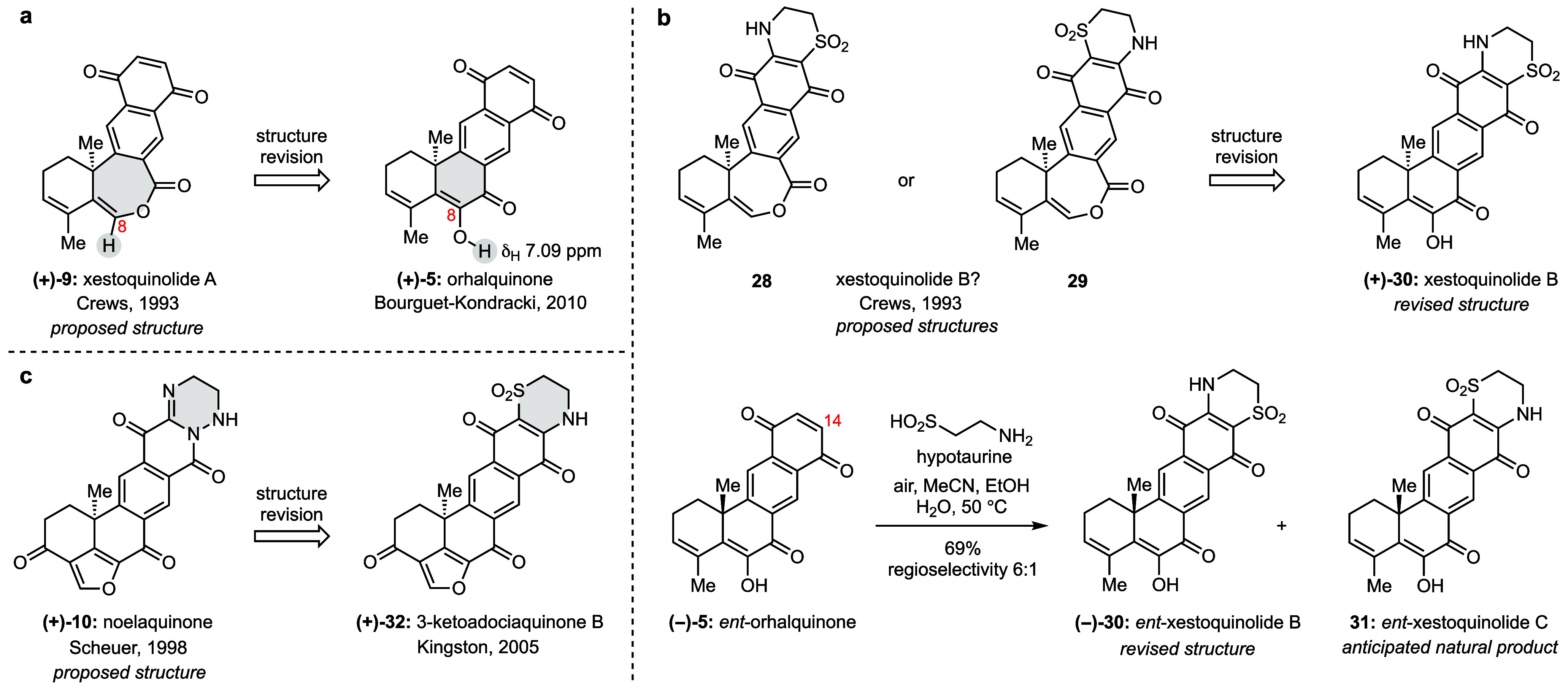
(a) Structural reassignment of xestoquinolide A. (b) Structure elucidation of xestoquinolide B enabled by total synthesis of its enantiomer. (c) Structural reassignment of noelaquinone.

Xestoquinolide B, a more complex meroterpenoid also reported by Crews, was proposed to arise from oxidative cycloaddition of hypotaurine with xestoquinolide A (Figure 4b). Several structurally related hypotaurine–benzoquinone meroterpenoids have been previously isolated from marine sponges.^38^ Although two candidate structures (**28** and **29**) were suggested, limited material precluded definitive assignment. Total synthesis was therefore an essential tool for the full structural assignment of xestoquinolide B.^39^ Treatment of *ent*-orhalquinone (−)-**5** with hypotaurine under aerobic conditions gave a 6:1 mixture of **30** and **31** in a 69% combined yield.^40^ Careful separation of this mixture by column chromatography gave a pure sample of the major regioisomer (−)-**30**, which was identical by ^1^H NMR with natural xestoquinolide B. 2D NMR analysis, in particular, key HMBC correlations from the N–H proton, allowed the relative orientation of the hypotaurine and orhalquinone substructures of **30** and **31** to be determined.^41^ These data support a revised structure for xestoquinolide B (+)-**30** arising from preferential nucleophilic addition of the hypotaurine sulfinic acid group to benzoquinone of **5** at C14.^42^ We predict that minor regioisomer **31** is likely to be a previously unreported *Xestospongia* metabolite, which we have named xestoquinolide C.^43^

Finally, we re-examined NMR data for noelaquinone **10**, isolated from an unidentified *Xestospongia* sp. by Scheuer and co-workers^28^ (Figure 4c). Although this natural product has attracted considerable synthetic interest,^44^ its unique 1,2,4-triazine ring system is difficult to reconcile with established meroterpenoid biosynthetic logic. Indeed, close examination of NMR data instead shows excellent agreement with 3-ketoadociaquinone B (**32**), a hypotaurine–halenaquinone adduct that was later isolated from a *Xestospongia* sp. by Kingston and co-workers.^45^ The originally assigned molecular formula and structure of noelaquinone can be attributed to misinterpretation of mass spectral data, as this class of compounds undergoes facile SO_2_ loss upon ionization. Thus, the true structures of xestoquinolides A and B and noelaquinone all contain the 6–6–6–6 ABCD ring system characteristic of the halenaquinone meroterpenoid family. Any proposed variations to this ring system within *Xestospongia* meroterpenoids isolated in the future should be critically evaluated.

### Biological Assessment

The biomimetic route described above enabled a preliminary evaluation of structure–activity relationships as a function of biosynthetic progression. All natural products, their quinone-protected derivatives, podocarpic acid, a commercial sample of wortmannin, and 5-fluorouracil (a chemotherapy drug employed as a reference control), were evaluated against a panel of human colon cancer cell lines (HCT116, SW620, RKO, and HT-29), with a subset also tested against a healthy colon cell line (CRL-1790) (Table 1). With only one exception (halenaquinone), the quinone-protected derivatives exhibited reduced anticancer activity. The impact of D-ring modification was also evident in comparisons of orhalquinone to xestoquinolides B and C. The mean IC_50_ values of xestoquinolides B and C are approximately 18 μM, whereas orhalquinone displays a mean IC_50_ of ca. 0.8 μM across the four colon cancer cell lines, corresponding to a 22-fold enhancement in potency. Given that hypotaurine incorporation is likely non-enzymatic, the reduced activity of these congeners is consistent with a convergent evolutionary rationale. With respect to the other family members, many displayed promising biological activity with IC_50_ values below 1 μM, with xestoquinone showing the highest degree of selectivity for cancerous versus healthy cell lines. Interestingly, introduction of the second benzylic ketone in halenaquinone resulted in a notable decrease in cytotoxicity, with the average IC_50_ across the four colon cancer cell lines exceeding 50 μM. As discussed above, this functionality contributes to the Michael acceptor pharmacophore implicated in phosphoinositide 3-kinase inhibition, suggesting that late-stage biosynthesis may reflect a shift in the defensive strategy. This behavior is reminiscent of the divergent biosynthesis of linear and angular furanocoumarins, which coexist within a single organism but operate via distinct defensive mechanisms.^46^ While this interpretation is consistent with the observed bioactivity profile of wortmannin, further studies are required to draw more definitive conclusions.

**Table 1:**
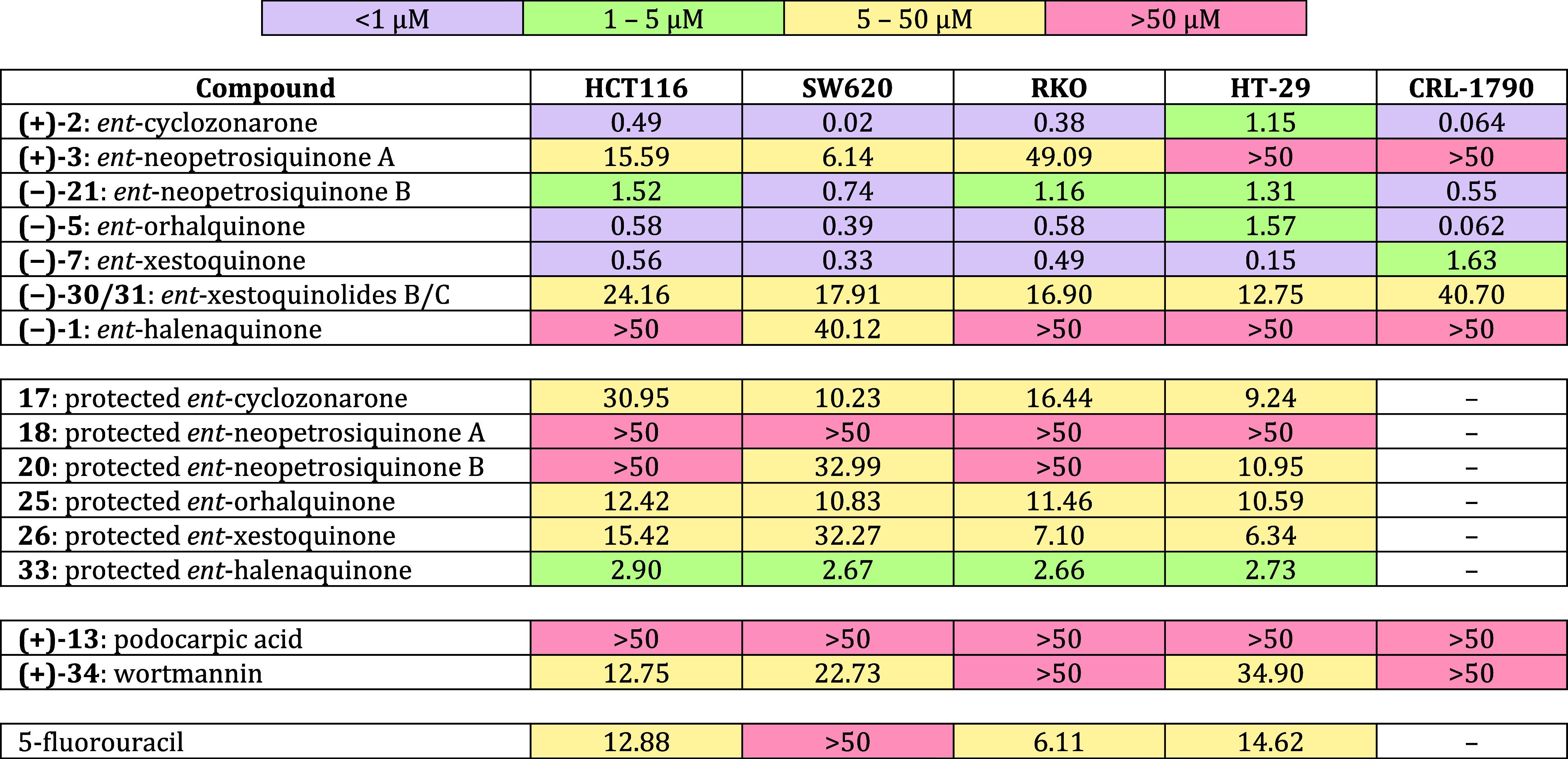
IC_50_ Values of Halenaquinone Family Members and Related Natural Products^a^

a See the Supporting Information for 95% confidence intervals.

## Conclusions

We report a collective total synthesis of seven members of the halenaquinone family, which enabled evaluation of their anticancer activity. In the abiotic “cyclase phase”, the characteristic 6–6–6–6 ABCD ring system of this meroterpenoid family was rapidly assembled through an intermolecular Diels–Alder reaction between a bisketene equivalent and “podocarp-yne”, a reactive benzyne intermediate generated by formal dehydration of the sustainable chiral pool starting material podocarpic acid. Inspired by the convergent evolution of structurally similar covalent PI3K inhibitors viridin and wortmannin, the subsequent biomimetic “oxidase phase” comprises a divergent sequence of chemoselective oxidations that parallels the proposed biosynthetic pathway, culminating in late-stage unveiling of the electrophilic diketofuran warhead. The sequence exploits the intrinsic reactivity of biosynthetic intermediates and proceeds with simple oxidants, including several aerobic conditions, thereby streamlining access to complex meroterpenoids under operationally simple conditions. Strikingly, the optimized route to halenaquinone features seven consecutive oxidation events, exemplifying excellent redox economy.^47^ The biosynthetic proposal is further supported by the structural reassignment of three natural products, noelaquinone and xestoquinolides A and B, and suggests the existence of several as yet undiscovered members of this family. Ongoing studies are directed toward extending this strategy to the synthesis of hibiscone and viridin natural products^48^ as well as more detailed biological assessments of all natural product targets.

## Supplementary Material



## Notes

Experimental details, characterization data, spectra, and anticancer assessment (PDF)
