# Supplementary material for: Biomimetic Synthesis of Seven Halenaquinone Meroterpenoids
Source: J Am Chem Soc. 2026 May 28;148(22):23316–24. doi: 10.1021/jacs.6c06760 (PMC13266709; doi:10.1021/jacs.6c06760)
Supplement: Supplementary file 1 [file ja6c06760_si_001.pdf]

Biomimetic Synthesis of Seven Halenaquinone Meroterpenoids

Jacob D. Hart,[a,§] Jay K. Lawrence,[b,§] Peter N. Franqui,[b] Douglas R. Perrott,[a] Bin Yi,[c,d] Yaguang Xi,[c,d] Christopher J. Sumby,[a] Christopher G. Newton[b,*] and Jonathan H. George[a,e*]

[a] *Department of Chemistry, The University of Adelaide, Adelaide, SA 5005, Australia*

[b] *Department of Chemistry, University of Georgia, Athens, Georgia 30602, United States*

[c] *Department of Pharmaceutical and Biomedical Sciences, University of Georgia, Athens, Georgia 30602, United States*

[d] *Innovations in Drug Discovery (IDD) Program, University of Georgia, Athens, Georgia 30602, United States*

[e] *School of Chemistry and Chemical Engineering, University of Southampton, Highfield, Southampton SO17 1BJ, United Kingdom*

[*] Email: chris.newton@uga.edu

[*] Email: jonathan.george@southampton.ac.uk

[§] *J.D.H. and J.K.L. contributed equally to this work and are listed in alphabetical order*

PREVIOUS SYNTHESES **3**
Summary 3
Cyclozonarone 4
Neopetrosiquinones A and B 6
Xestoquinone 7
Halenaquinone 12
3-Ketoadociaquinones A and B 17
GENERAL EXPERIMENTAL **18**
ISOLATION AND CHARACTERIZATION OF PODOCARPIC ACID **20**
SYNTHETIC PROCEDURES AND CHARACTERIZATION DATA **23**
Part 1: A Diels–Alder Disconnection 23
Part 2: Cyclozonarone and Neopetrosiquinones A and B 30
Cyclozonarone *30*
Neopetrosiquinone A *35*
Neopetrosiquinone B *39*
Part 3: Orhalquinone, Xestoquinone, and Halenaquinone 42
Orhalquinone *42*
Xestoquinone *46*
Halenaquinone *50*
Part 4: Xestoquinolides B and C 56
COMPARISON OF ISOLATED NATURAL PRODUCTS WITH SYNTHETIC SAMPLES **58**
Cyclozonarone 58
Neopetrosiquinone A 59
Neopetrosiquinone B 62
Orhalquinone 65
Xestoquinone 68
Halenaquinone 69
Xestoquinolide B 70
NATURAL PRODUCT STRUCTURAL REVISIONS **72**
Xestoquinolide A is Orhalquinone 72
Noelaquinone is 3-Ketoadociaquinone A 74
X-RAY CRYSTALLOGRAPHIC DATA **75**
ANTICANCER ASSESSMENT **79**
Procedure 79
Summary 80
Response Curves 81
NMR SPECTRA **85**
Isolation and Characterization of Podocarpic Acid 85
Part 1: A Diels–Alder Disconnection 96
Part 2: Cyclozonarone and Neopetrosiquinones A and B 115
Part 3: Orhalquinone, Xestoquinone, and Halenaquinone 145
Part 4: Xestoquinolide B 179
REFERENCES **187**

Previous Syntheses

Total step count (reported as longest linear sequence) and yields are measured from either (i) isolated natural products, or (ii) commercially available starting materials through incorporation of literature procedures referenced within each synthesis. In some cases, total step count and total yield are underestimated due to ambiguity and/or missing details. Unless stated otherwise, if multiple successful approaches are reported within the same publication, we present only the shortest route.

Summary

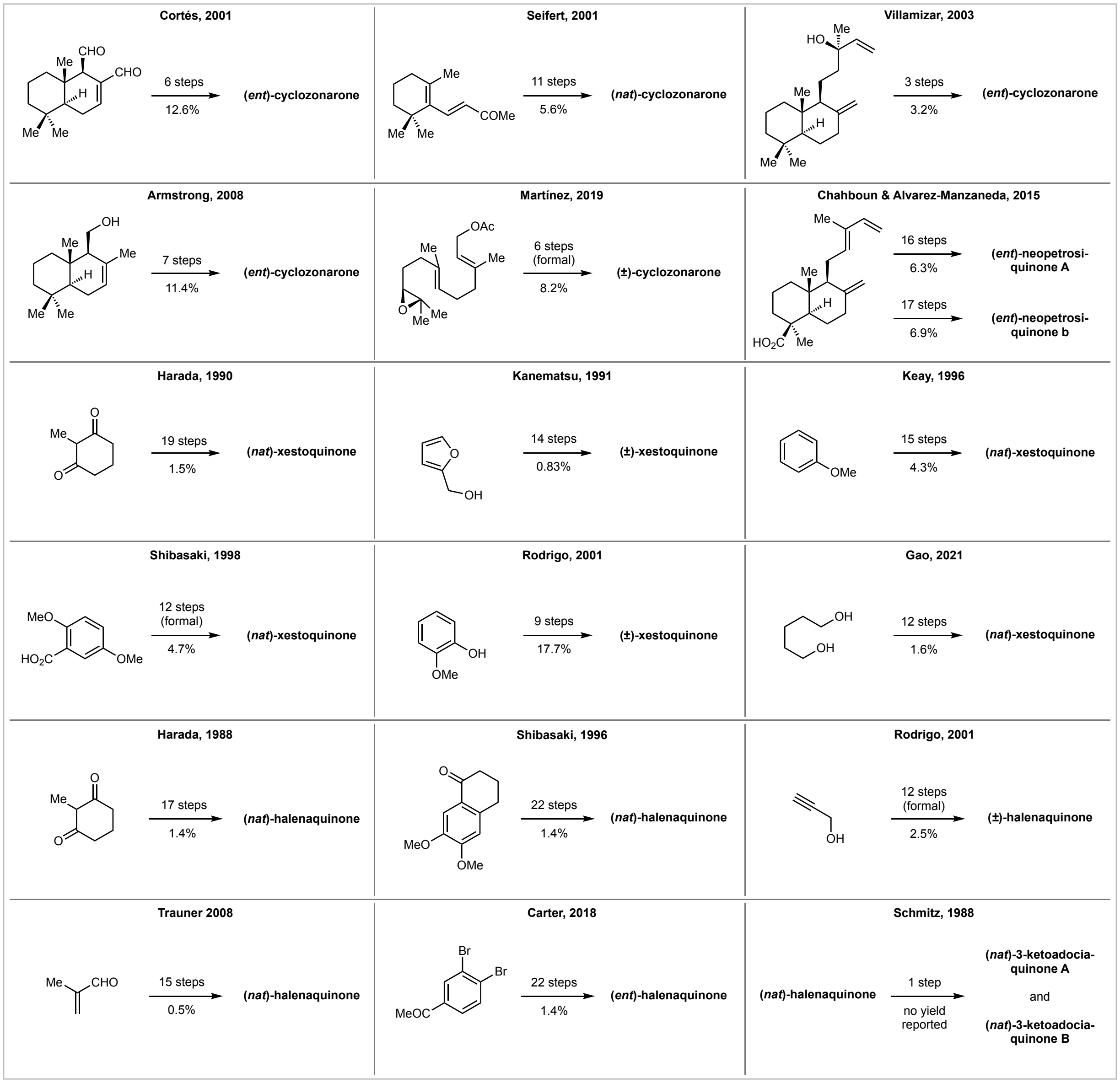

Cyclozonarone

Cortés, 2001[1]

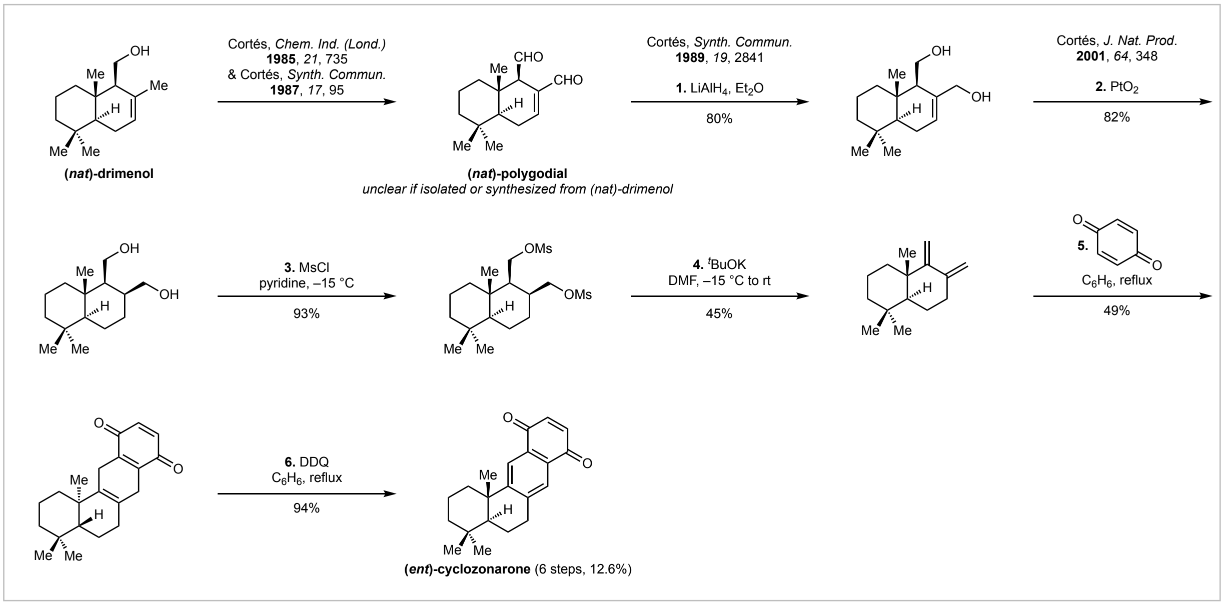

Seifert, 2001[2]

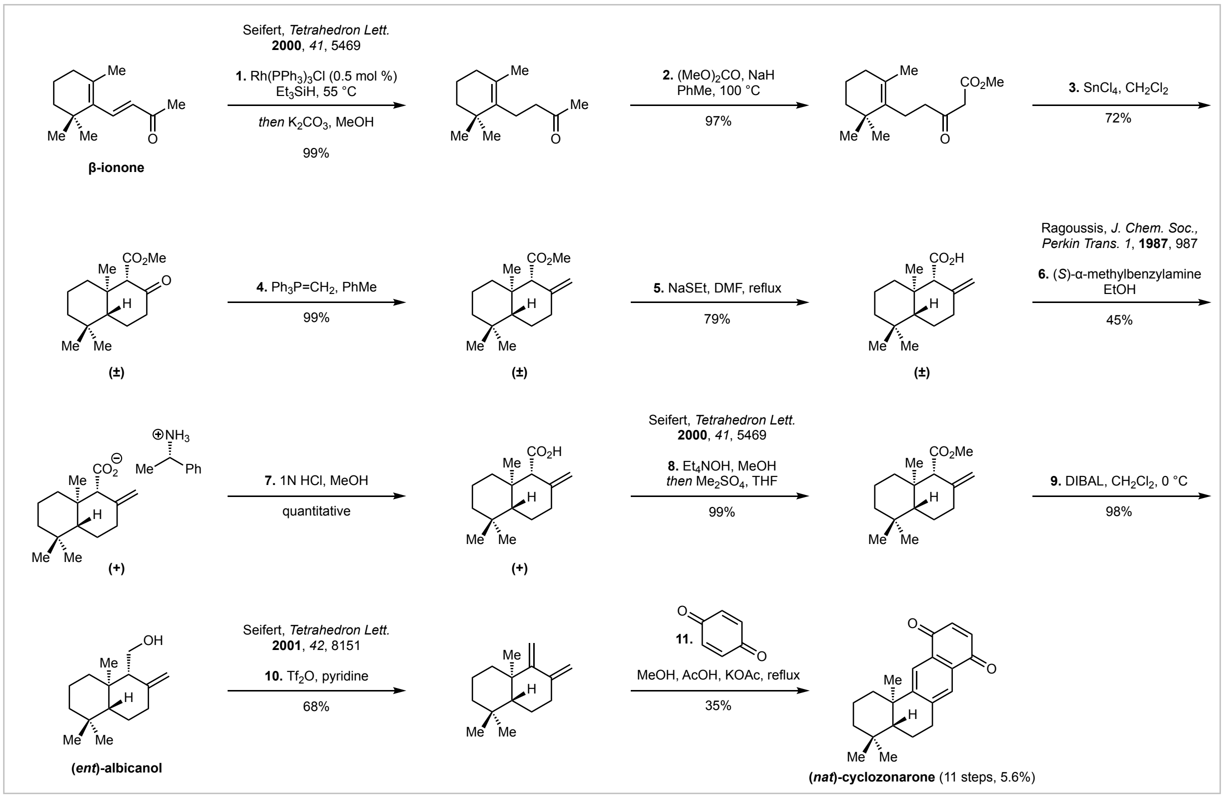

Villamizar, 2003[3]

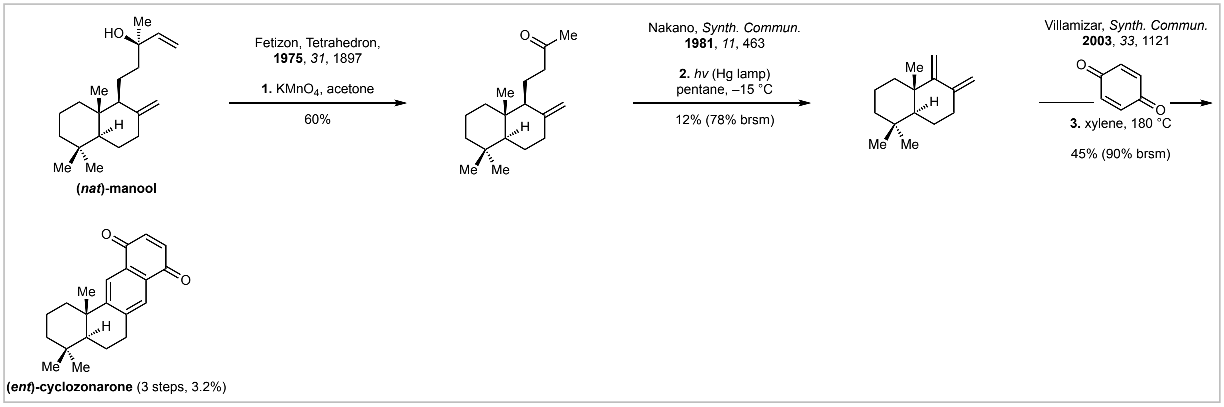

Armstrong, 2008[4]

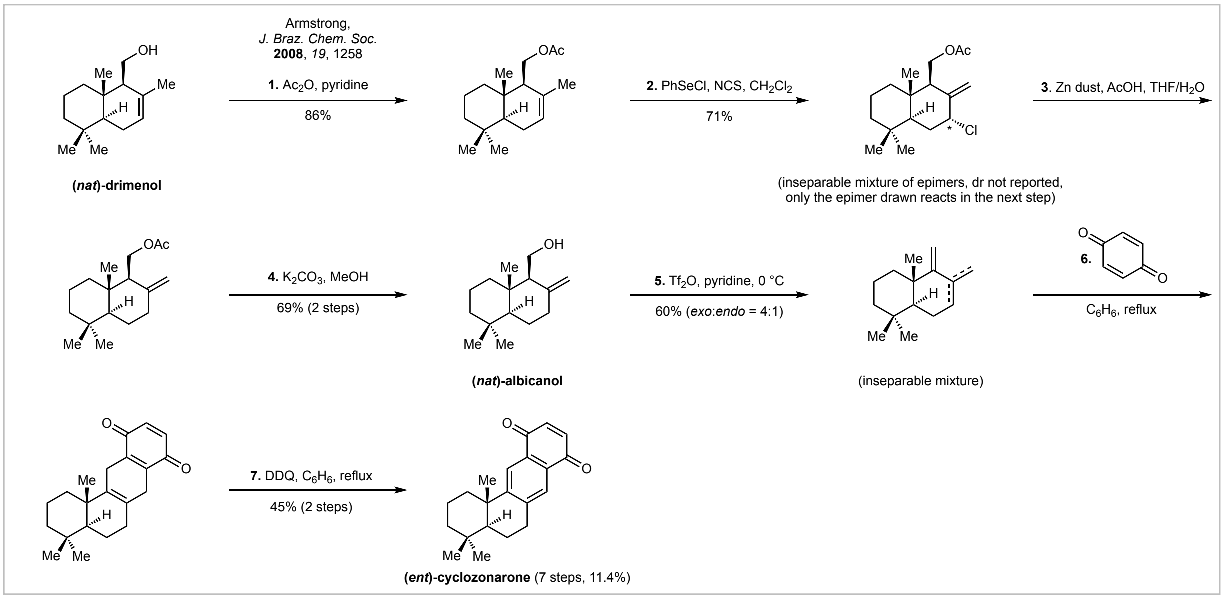

Martínez, 2019[5]

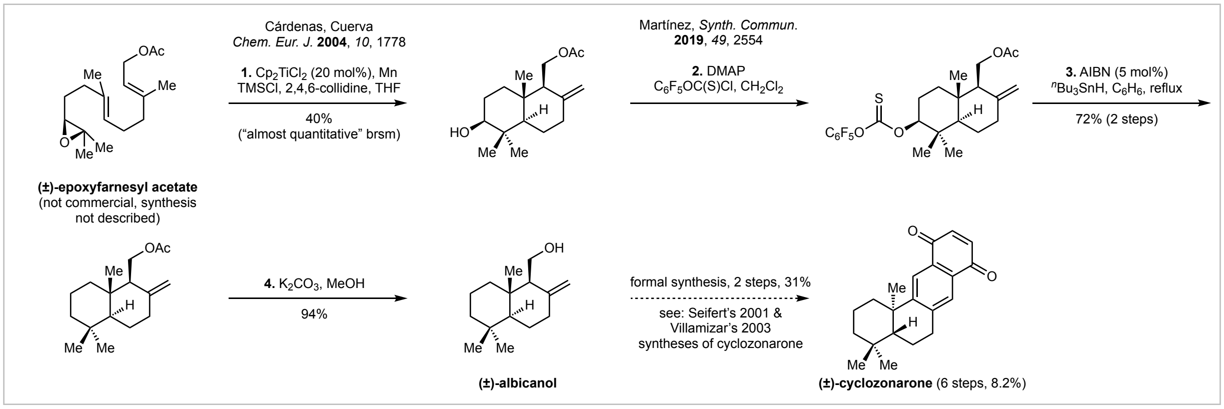

Neopetrosiquinones A and B

Chahboun and Alvarez-Manzaneda, 2015[6]

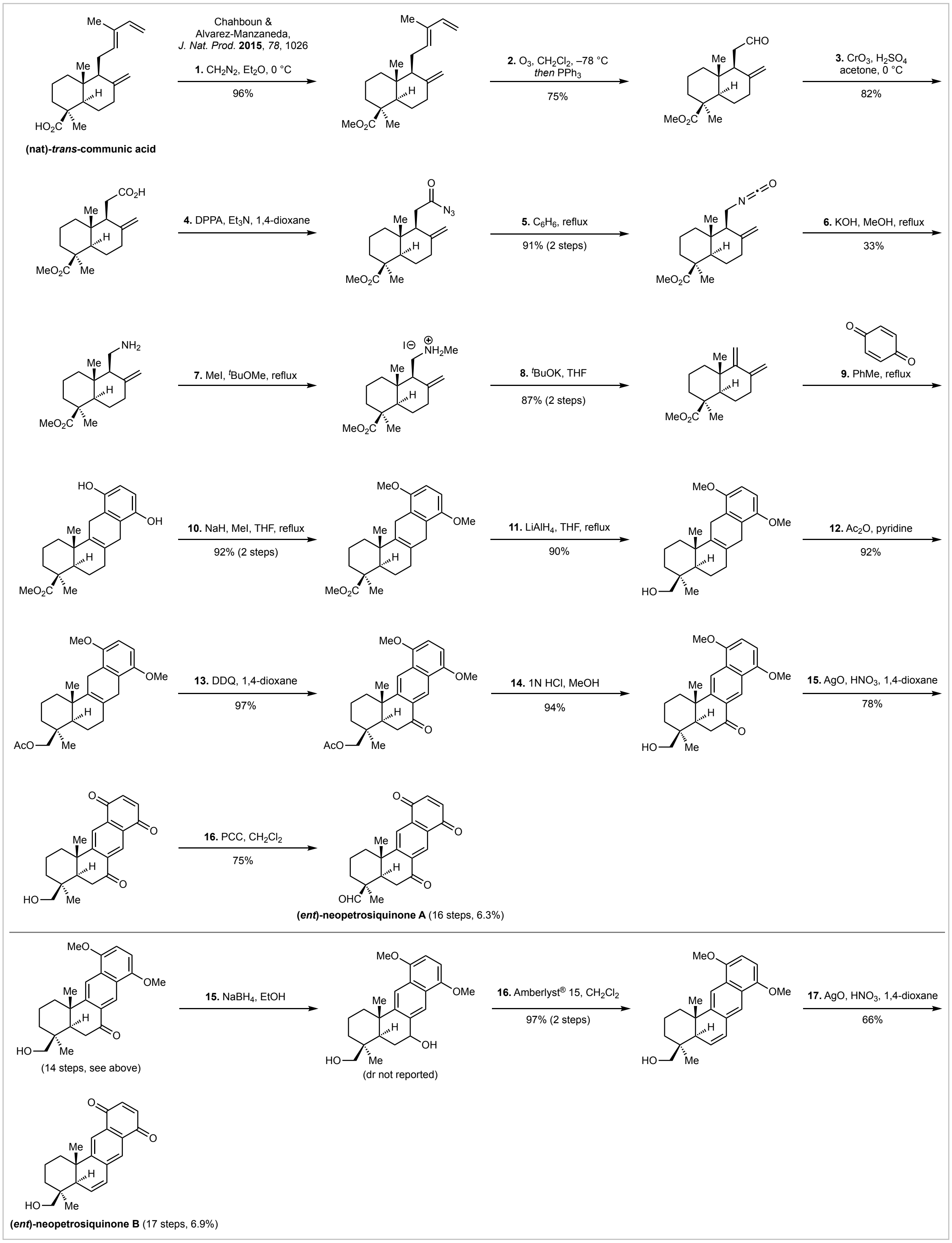

Xestoquinone

Harada, 1990[7]

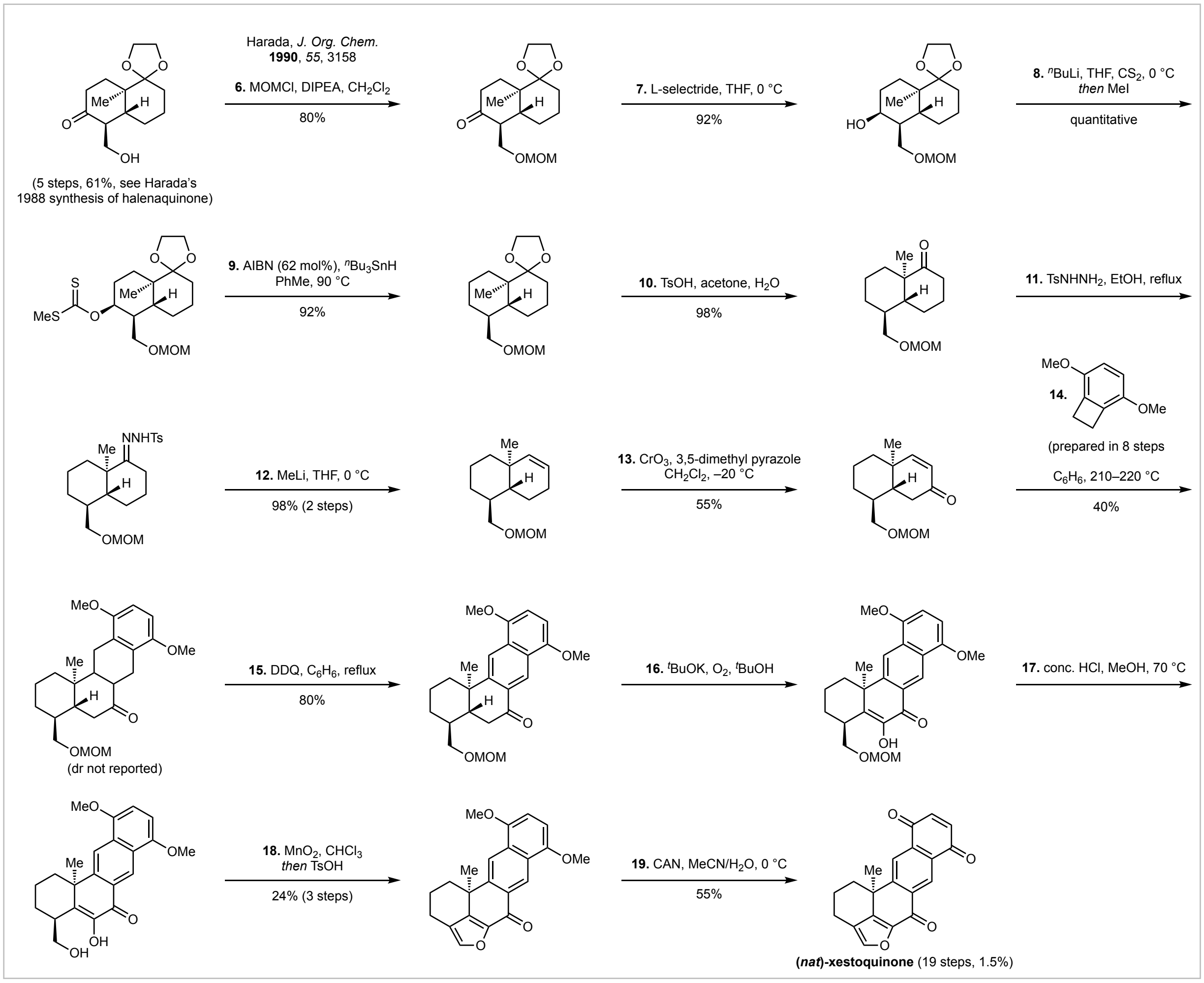

Kanematsu, 1991[8]

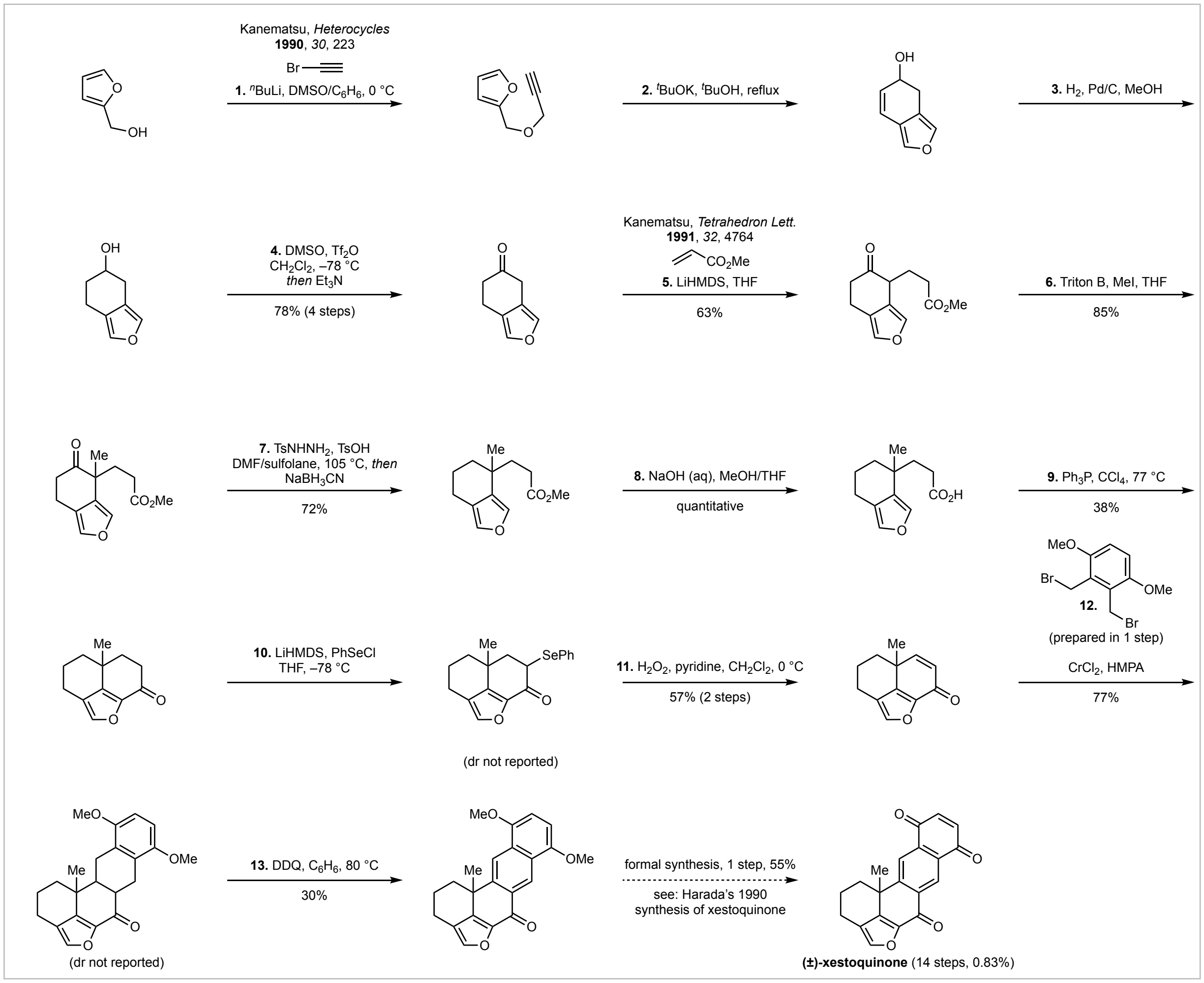

Keay, 1996[9]

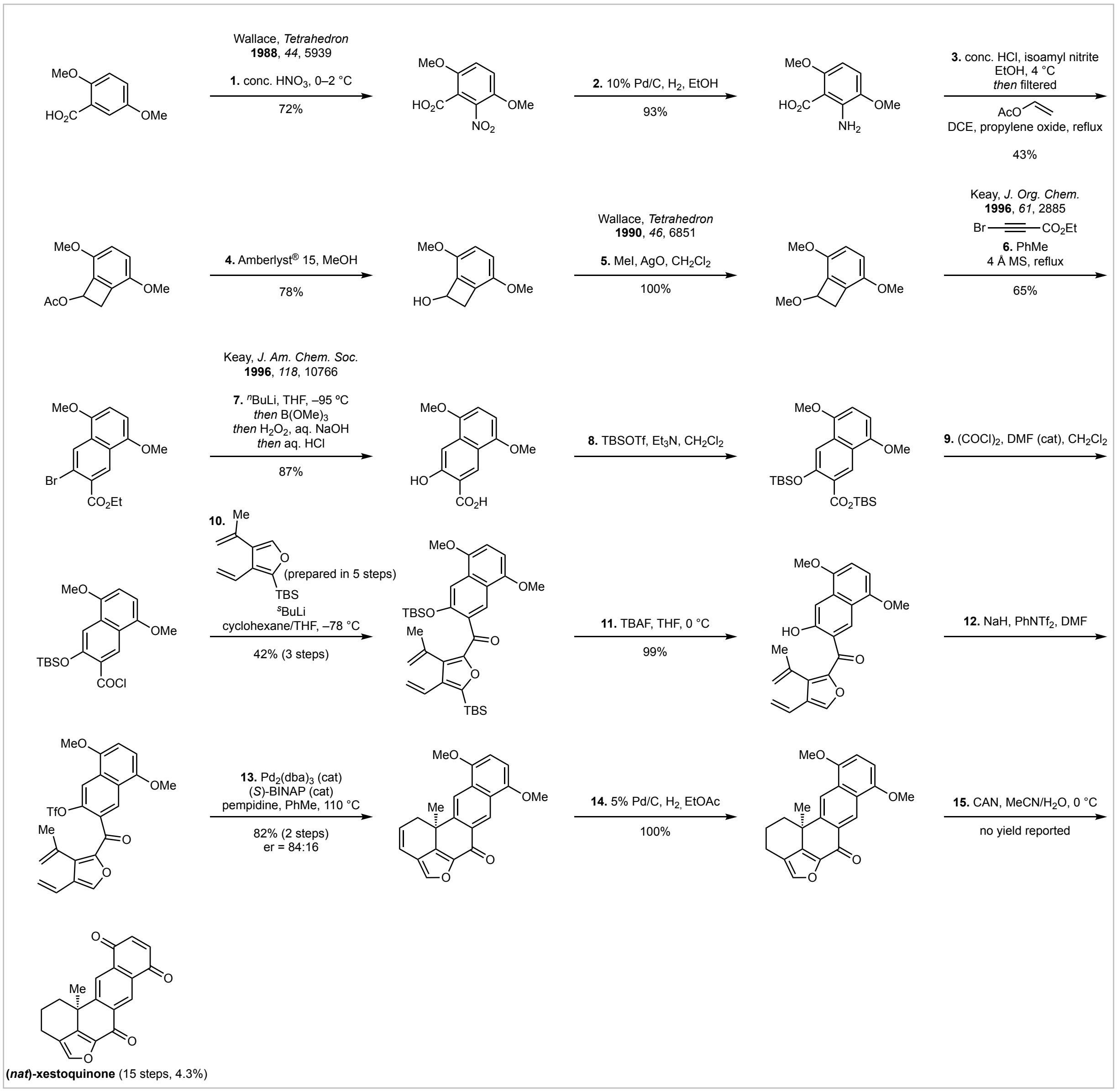

Wallace, Tetrahedron 1988, 44, 5939
1. conc. HNO3, 0–2 °C
72%
2. 10% Pd/C, H2, EtOH
93%
3. conc. HCl, isoamyl nitrite EtOH, 4 °C then filtered
DCE, propylene oxide, reflux
43%
4. Amberlyst® 15, MeOH
78%
Wallace, Tetrahedron 1990, 46, 6851
5. MeI, AgO, CH2Cl2
100%
Keay, J. Org. Chem. 1996, 61, 2885
6. PhMe 4 Å MS, reflux
65%
Keay, J. Am. Chem. Soc. 1996, 118, 10766
7. nBuLi, THF, –95 ºC then B(OMe)3 then H2O2, aq. NaOH then aq. HCl
87%
8. TBSOTf, Et3N, CH2Cl2
9. (COCl)2, DMF (cat), CH2Cl2
10.
(prepared in 5 steps)
sBuLi cyclohexane/THF, –78 °C
42% (3 steps)
11. TBAF, THF, 0 °C
99%
12. NaH, PhNTf2, DMF
13. Pd2(dba)3 (cat) (S)-BINAP (cat) pempidine, PhMe, 110 °C
82% (2 steps) er = 84:16
14. 5% Pd/C, H2, EtOAc
100%
15. CAN, MeCN/H2O, 0 °C
no yield reported
(nat)-xestoquinone (15 steps, 4.3%)

Shibasaki, 1998[10]

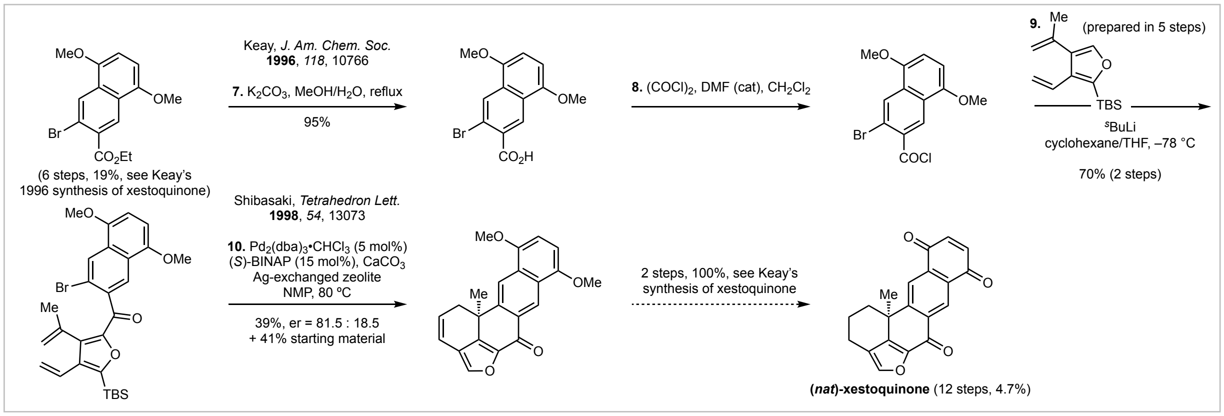

Rodrigo, 2001[11]

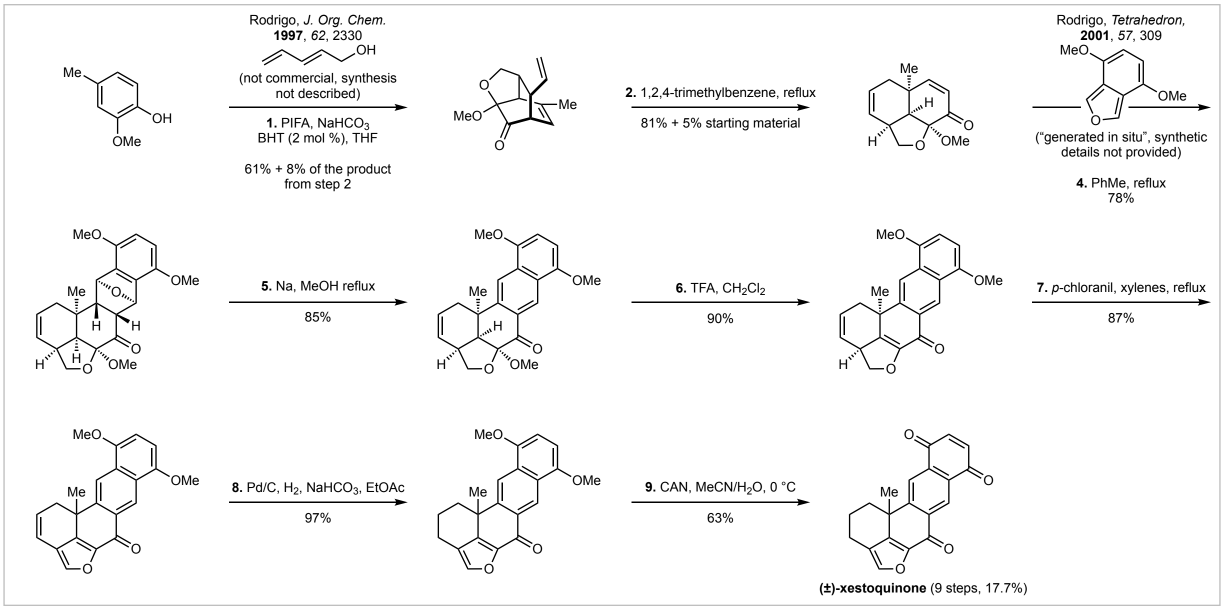

Gao, 2021[12]

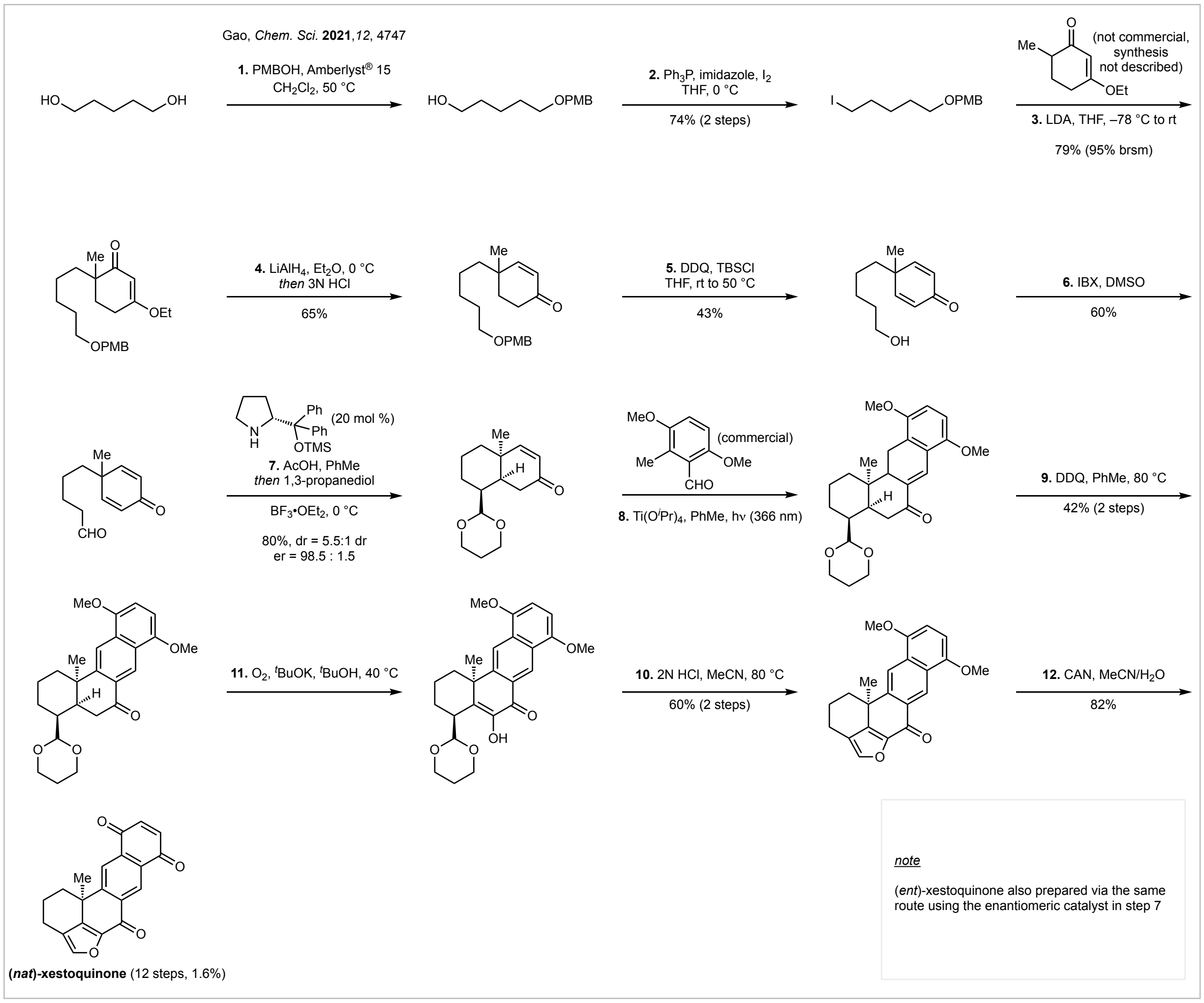
Gao, Chem. Sci. 2021,12, 4747
1. PMBOH, Amberlyst® 15
CH2Cl2, 50 °C
2. Ph3P, imidazole, I2
THF, 0 °C
74% (2 steps)
(not commercial, synthesis not described)
3. LDA, THF, –78 °C to rt
79% (95% brsm)
4. LiAlH4, Et2O, 0 °C
then 3N HCl
65%
5. DDQ, TBSCl
THF, rt to 50 °C
43%
6. IBX, DMSO
60%
(20 mol %)
7. AcOH, PhMe
then 1,3-propanediol
BF3•OEt2, 0 °C
80%, dr = 5.5:1 dr
er = 98.5 : 1.5
(commercial)
8. Ti(OiPr)4, PhMe, hν (366 nm)
9. DDQ, PhMe, 80 °C
42% (2 steps)
11. O2, tBuOK, tBuOH, 40 °C
10. 2N HCl, MeCN, 80 °C
60% (2 steps)
12. CAN, MeCN/H2O
82%
(nat)-xestoquinone (12 steps, 1.6%)
note
(ent)-xestoquinone also prepared via the same route using the enantiomeric catalyst in step 7

Halenaquinone

Harada, 1988[13]

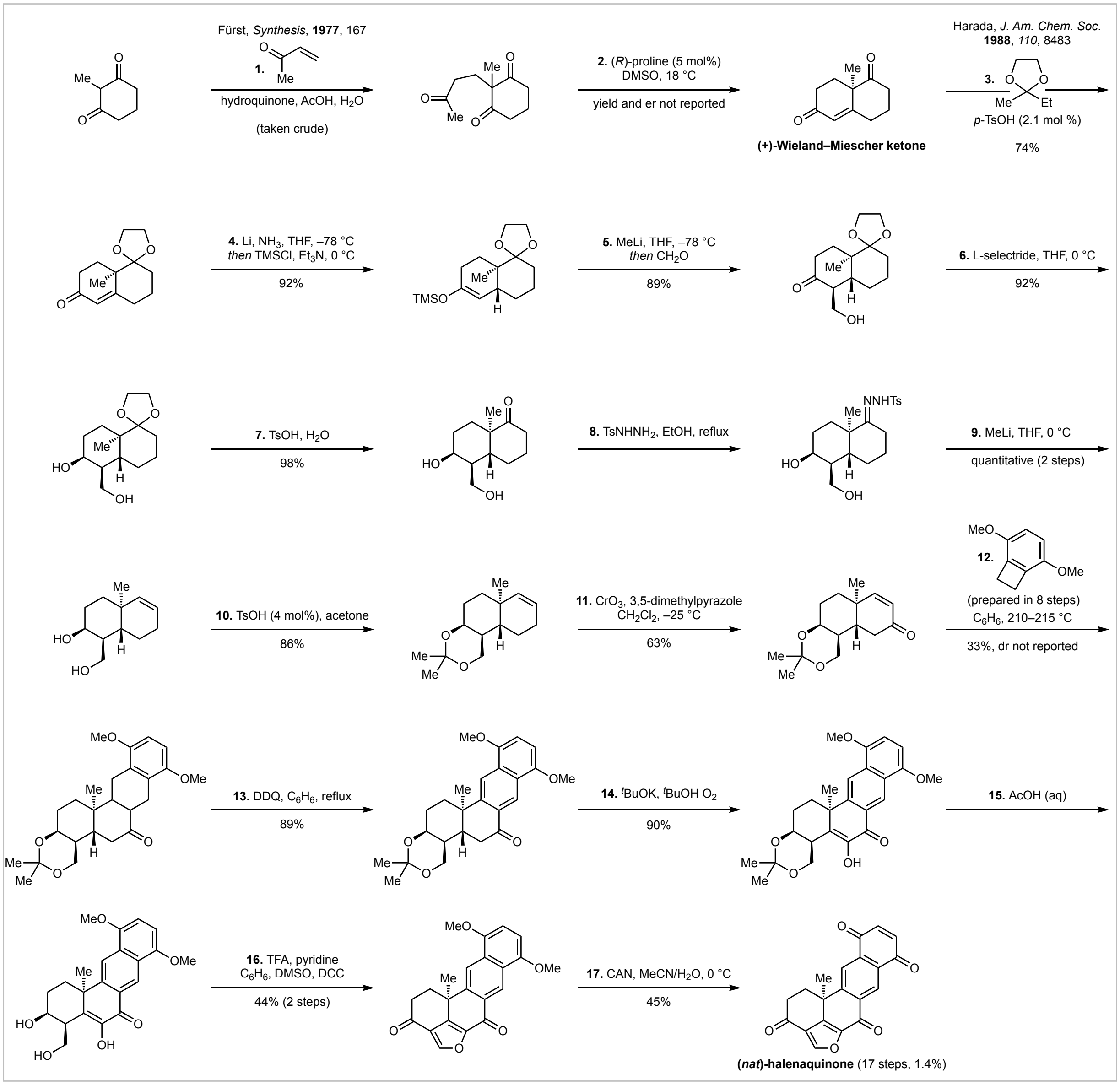

Shibasaki, 1996[14]

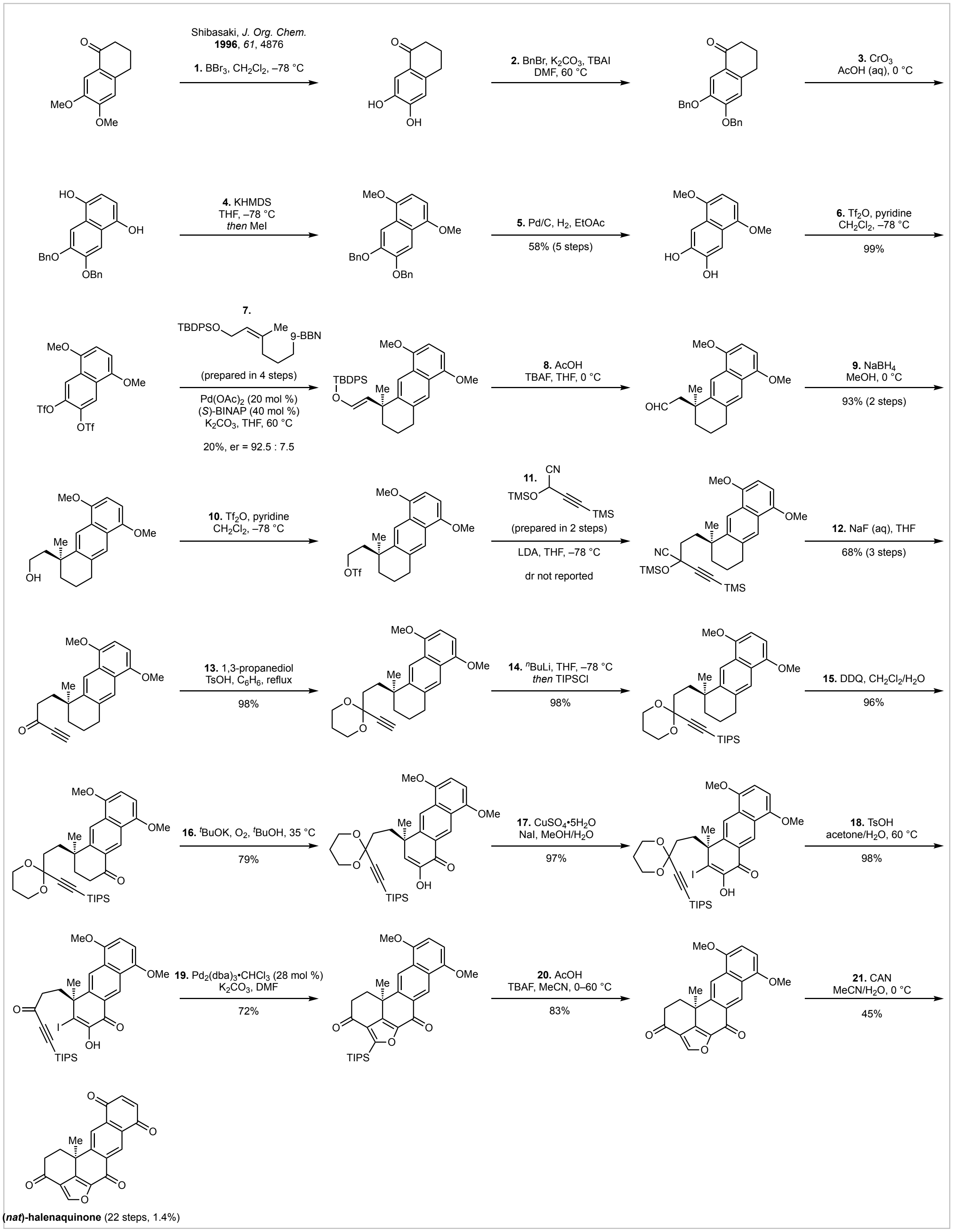

Rodrigo, 2001[15]

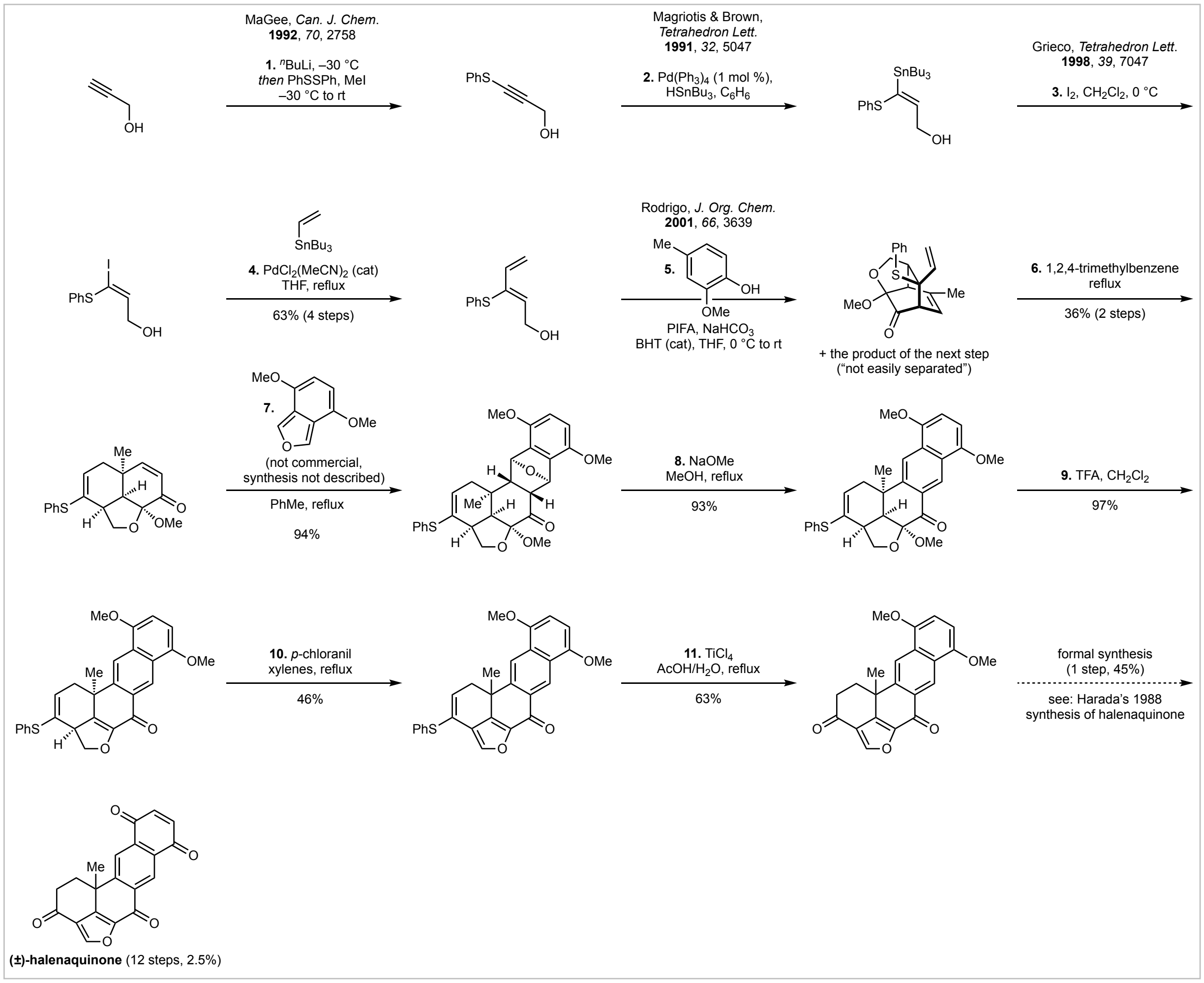

Trauner, 2008[16]

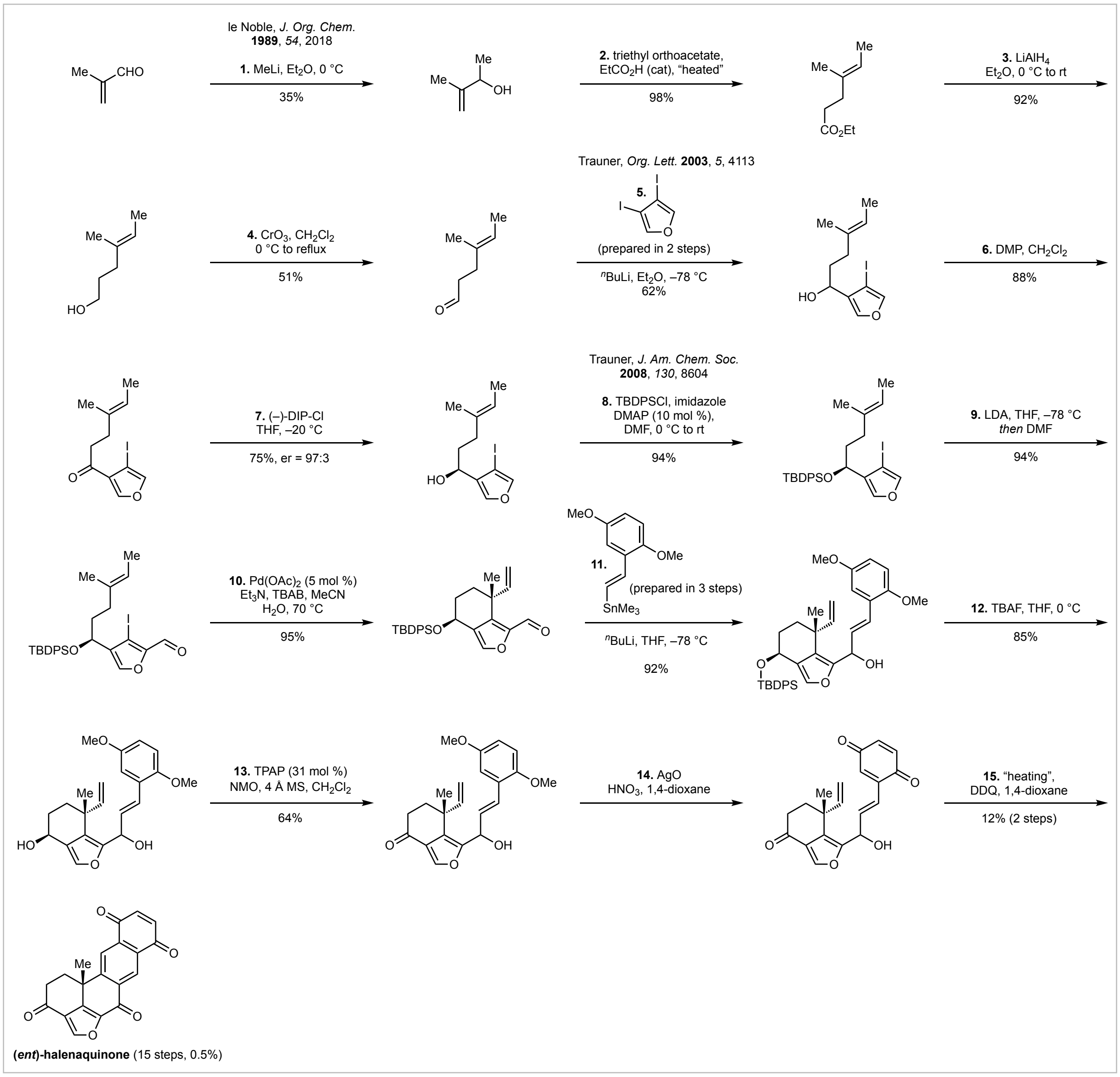

Carter, 2018[17]

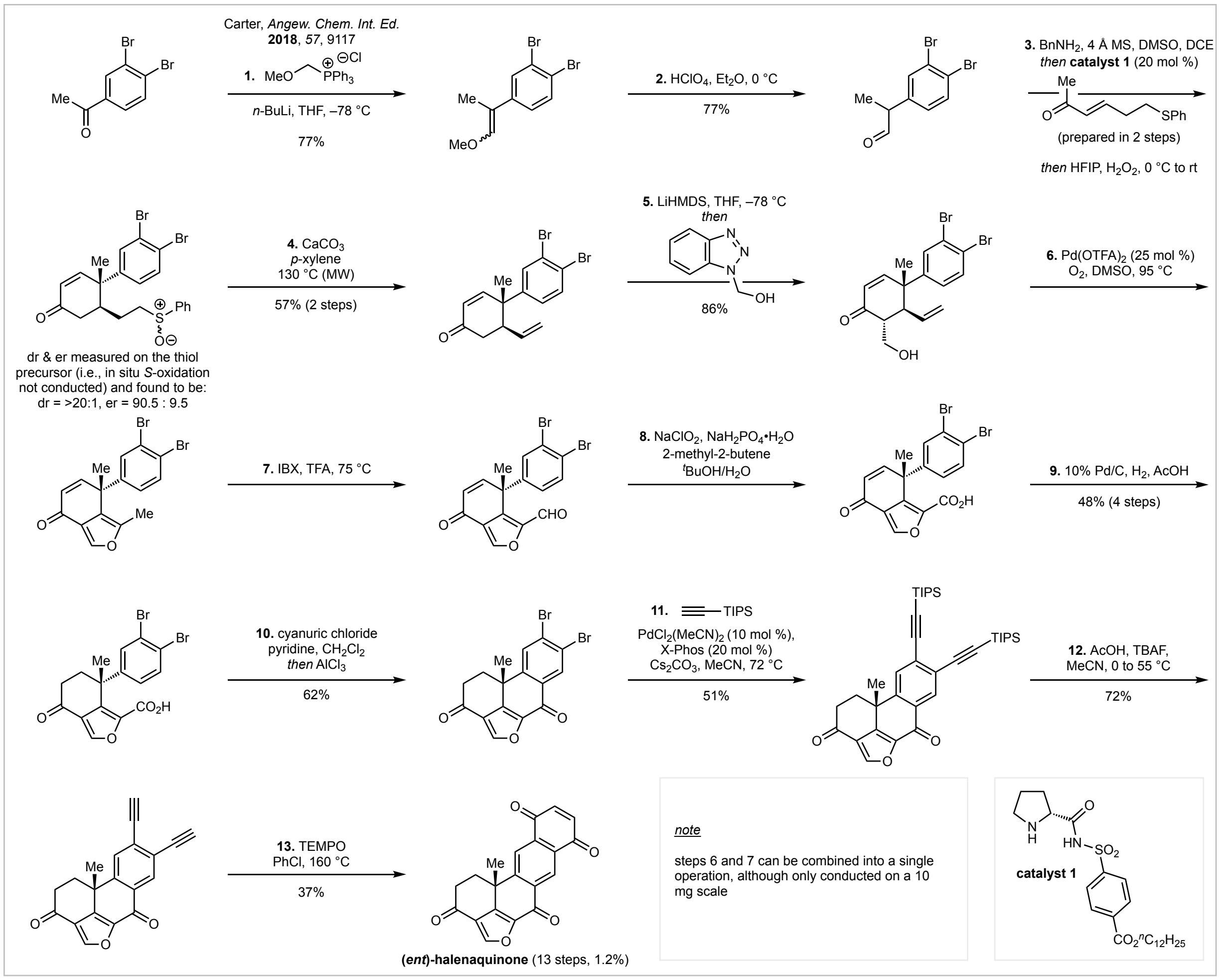

3-Ketoadociaquinones A and B

Schmitz, 1988[18]

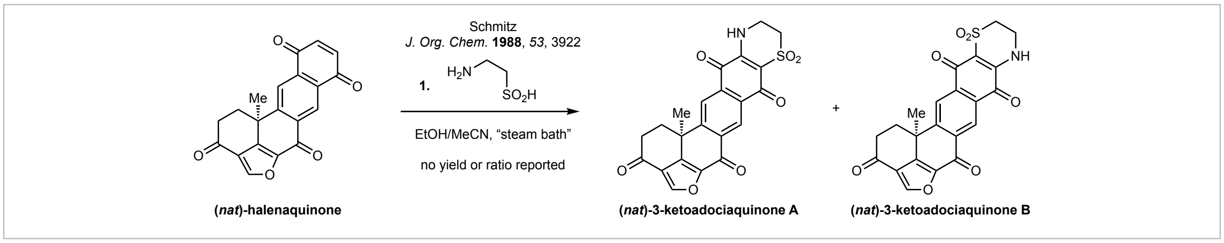

General Experimental

NMR Spectroscopy

^{1}H NMR spectra were recorded using either a Bruker 600 MHz Avance NEO console with an Oxford AS600 magnet, Bruker 600 MHz Avance NEO console with a Varian Premium Shielded 600 MHz magnet, Bruker 900 MHz Avance NEO console with an Oxford 900 MHz magnet, Avance 400 MHz III HD console with a Bruker Ascend 400 MHz magnet, or Agilent 600 MHz DD2 console with an Oxford 600 MHz magnet. ^{13}C NMR spectra were recorded using either a Bruker 600 MHz Avance NEO console with an Oxford AS600 magnet at 150 MHz, Bruker 600 MHz Avance NEO console with a Varian Premium Shielded 600 MHz magnet at 150 MHz, Bruker 900 MHz Avance NEO console with an Oxford 900 MHz magnet at 225 MHz, or Avance 400 MHz III HD console with a Bruker Ascend 400 MHz magnet at 100 MHz. ^{19}F NMR spectra were recorded using an Avance 400 MHz III HD console on a Bruker Ascend 400 MHz magnet at 377 MHz. Residual solvent peaks were used as an internal reference for ^{1}H NMR spectra [$CDCl_3$ δ 7.26 ppm, CD_3CN δ 1.94 ppm, $(CD_3)_2CO$ δ 2.05 ppm, $(CD_3)_2SO$ δ 2.50 ppm, or CD_2Cl_2 δ 5.32 ppm] and ^{13}C NMR spectra [$CDCl_3$ δ 77.16 ppm, CD_3CN δ 118.26 ppm, $(CD_3)_2CO$ δ 29.84 ppm, $(CD_3)_2SO$ δ 39.52 ppm, or CD_2Cl_2 δ 53.80 ppm]. ^{19}F NMR spectra were reported relative to the ^{19}F resonance of CF_3CO_2H [$CDCl_3$ δ −75.39 ppm].[19] Coupling constants (*J*) were quoted to the nearest 0.1 Hz. For ^{13}C NMR, coupling constants were included only in the case of coupling with ^{19}F nuclei. The following abbreviations (or combinations thereof) were used to describe ^{1}H NMR multiplicities: s = singlet, d = doublet, t = triplet, q = quartet, p = pentet, m = multiplet, br = broad.

Infrared Spectroscopy

IR spectra were recorded neat on a Thermo Nicolet iS10 spectrometer and are reported in wavenumbers (cm^{-1}).

Mass Spectrometry

High resolution mass spectrometry (HRMS) data were acquired via electrospray ionization (ESI) using either a Q-TOF mass spectrometer produced by Bruker model Impact II or Bruker Solarix XR 12 T FTICR MS.

Chromatography

Flash chromatography was performed with SiliaFlash® P60 silica, 0.040–0.063 mm grade. Analytical thin-layer chromatography was performed with commercial glass sheets coated with 0.25 mm silica gel (SiliaPlate™, silica gel 60, F254). Compounds were either visualized under UV-light at 254 nm, or by dipping the plates in an aqueous potassium permanganate solution followed by heating, unless stated otherwise. All R_f values were measured to the nearest 0.1 cm.

Melting Points

Melting points were measured on a DigiMelt melting point apparatus, model SRS MPA161, and are uncorrected.

Optical Rotations

Optical rotations were recorded on an AUTOPOL III Automatic Polarimeter with a path length of 10 dm at the specified temperature and concentration (in g/100 mL).

Preparation of Phosphate Buffered Silica (pH = 7)

Prepared according to the method of Newton.[20] To a 5-L Erlenmeyer flask open to air and equipped with a stir bar was added deionized water (380 mL). Sodium phosphate dibasic (114 g, 800 mmol) was slowly added with vigorous stirring, followed by additional deionized water to reach a total volume of 4000 mL (0.200 M). Once fully dissolved (ca. 10 minutes), 400 g of silica gel was slowly added. The pH of the mixture was measured (pH paper, range 1–13) to confirm the solution is neutral. If basic, the mixture was neutralized by either: (i) dropwise addition of phosphoric acid or (ii) addition of further silica gel (10.0 g portions). If acidic, additional phosphate buffer (0.200 M, prepared as above) is added. Once neutral, the mixture was filtered through a 2.00-L, 13.5-cm diameter sintered funnel (medium grit), then air was pulled through the funnel for 30 minutes. The phosphate buffered silica was transferred to a 2.00-L shallow glass dish and placed in an oven (105 °C) for three days, stirring every day to ensure even drying. The phosphate buffered silica was allowed to cool to ambient temperature and was sifted through a sieve into a container for long term storage. **Appearance**: White, free-flowing solid that partially adheres to glass when wet with solvent. **Note**: When using phosphate buffered silica, the addition of acidified sand to ensure a level silica gel line should be avoided. Instead, we recommend using additional phosphate buffered silica as a sand replacement.

Experimental Procedures and Reagents

Commercially available chemicals were used as purchased or, where specified, purified by standard techniques. Solvent compositions are given in v/v. All reactions were carried out under an atmosphere of argon in flame-dried glassware unless otherwise indicated. All reactions underwent magnetic stirring employing an IKA plate, with heating facilitated by either an OptiTherm® heating mantle or a silicon oil bath when required. Anhydrous acetonitrile, dichloromethane, tetrahydrofuran, diethyl ether, and toluene were purified by an Mbraun solvent purification system. All other solvents were used as purchased or, where specified, purified by standard techniques.

Isolation and Characterization of Podocarpic Acid

Large Scale Isolation of Podocarpic Acid from Rimu (*Dacrydium cupressinum*)

A. Jonathan Singh, Tracey J. Bell, Simon F. Hinkley, Ferrier Research Institute, Victoria University of Wellington, New Zealand

Background

In Aotearoa New Zealand, the heartwood of the rimu tree (*Dacrydium cupressinum*, family Podocarpaceae) is highly valued for its strength and appearance as a furniture and construction medium. Within the heartwood, shakes—cracks within the material—contain concentrated deposits of the abietene diterpenoid, podocarpic acid, which can be readily identified as veins of off-white solids.

Procedure

Equipment

- Mallet
- Chisel (or similar)
- Collecting tray
- Small paintbrush (or similar)
- Pestle and mortar
- Sieves (850 and 500 micron)
- Thick gloves (e.g., gardening gloves)
- Safety glasses
- Dust mask (N95)

Harvesting

A section of one salvaged rimu beam (**Figure 1**) showed a prominent vein of podocarpic acid and was targeted for harvest. The off-white deposits in the shakes were removed by hand from the wooden beam using a mallet and a chisel to pry open the heartwood. Recovered material was ground using a pestle and mortar and passed through sieves (850 then 500 micron) to separate the deposits from wooden shards. A total of 215.02 g of powdered crude material was obtained.

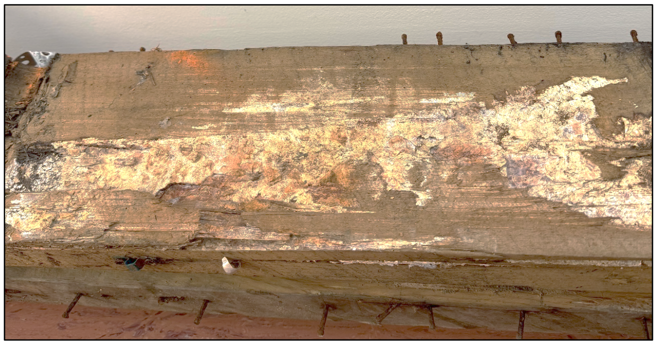

Figure 1. Rimu beams salvaged from the demolition of a ca. 1960s prefabricated building destined for disposal; shakes presented as an off-white solid.

Purification

Powdered crude material (210.98 g) and EtOAc (500 mL) were combined and stirred at room temperature for two hours. The resulting viscous red-brown solution was vacuum filtered through Celite to remove insoluble material, and concentrated to produce a tan, brown solid (201.19 g, **Figure 2a**). The solids were transferred with warm ethanol (300 mL) to a 1-L conical flask and heated to 75 °C while stirring. In a procedure adapted from Easterfield,[21] hot distilled water (350 mL total) was added to the ethanolic solution until persistently opaque. Ethanol was added to recover the solution, the mixture was cooled to 60 °C, and a seed crystal of podocarpic acid was added to induce crystallization. Within 30 seconds a precipitate formed, at which point the mixture was left to cool overnight with stirring (**Figure 2b**). The resulting solids (small, fine off-white needles) were filtered and washed with ice-cold EtOH/water (1:1, approximately 120 mL). The first crop of crystalline material (184.79 g) was desiccated for 19 hours, yielding 180.72 g of podocarpic acid (**Figure 2c**). The filtrate and washings from this process appeared milky and eventually produced solids, indicating the availability of a second crop (retained, but not processed).

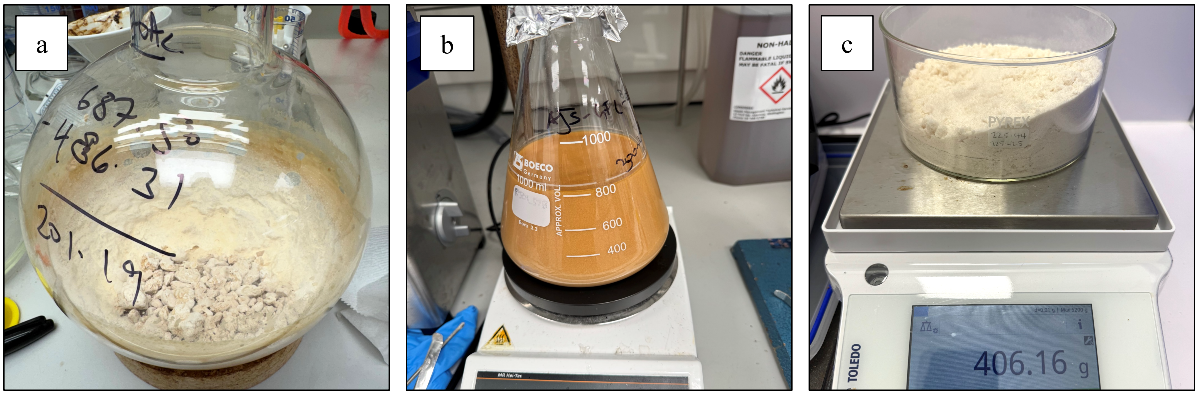

Figure 2. a) Crude rimu shake deposits. b) Purification of podocarpic acid. c) Pure podocarpic acid.

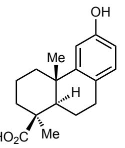

(+)-13: podocarpic acid

Characterization data matched those reported by Parish.[22]

Appearance: white solid;

R*f*: 0.29 (3:7 diethyl ether:petroleum ether);

M.p.: 193.6 – 195.2 °C;

^{1}H NMR [600 MHz, $(CD_3)_2CO$]: δ 6.83 (d, *J* = 8.2 Hz, 1H), 6.76 (d, *J* = 2.5 Hz, 1H), 6.56 (dd, *J* = 8.2, 2.6 Hz, 1H), 2.78 (ddd, *J* = 16.3, 5.6, 2.1 Hz, 1H), 2.72 – 2.65 (m, 1H), 2.25 – 2.20 (m, 2H), 2.19 – 2.16 (m, 1H), 2.09 – 1.97 (m, 2H), 1.66 – 1.55 (m, 1H), 1.53 (dd, *J* = 12.4, 1.9 Hz, 1H), 1.36 (ddd, *J* = 13.5, 4.3, 4.3 Hz, 1H), 1.29 (s, 3H), 1.12 (s, 3H), 1.11 (ddd, *J* = 13.5, 4.1, 4.1 Hz, 1H) ppm;

IR: 3305, 3152, 2963, 2854, 1695, 1582, 1499, 1471, 1342, 1330, 1211, 1177, 1145, 802, 781, 720 cm^{-1};

HRMS (ESI): calculated for $[C_{17}H_{22}O_3{-}H]^-$: 273.1496, found: 273.1490;

$[\alpha]_D^{25}$: 37.3° (c = 0.950, CH_3OH).

To the best of our knowledge, only partial ^{1}H NMR data for podocarpic acid has been reported in the literature.[22] While our NMR data above is in agreement, we felt it prudent to also report full NMR data in both CD_3OD and $(CD_3)_2SO$ (vide infra).

^{1}H NMR (500 MHz, CD_3OD): δ 6.82 (d, *J* = 8.3 Hz, 1H), 6.69 (d, *J* = 2.0 Hz, 1H), 6.50 (dd, *J* = 8.2, 2.2 Hz, 1H), 2.78 (dd, *J* = 16.1, 4.5 Hz, 1H), 2.67 (td, *J* = 16.2, 14.5, 5.8 Hz, 1H), 2.22 (d, *J* = 13.2 Hz, 2H), 2.17 (dd, *J* = 13.7, 5.2 Hz, 1H), 2.10 – 1.94 (m, 2H), 1.59 (d, *J* = 13.9 Hz, 1H), 1.51 (d, *J* = 12.1 Hz, 1H), 1.36 (ddd, *J* = 13.0, 13.0, 3.1 Hz, 1H), 1.28 (s, 3H), 1.12 (s, 3H), 1.10 (ddd, *J* = 14.0, 14.0, 3.9 Hz, 1H) ppm;

^{13}C NMR (125 MHz, CD_3OD): δ 181.6, 156.2, 150.5, 130.9, 127.4, 114.0, 112.8, 54.3, 44.9, 40.8, 39.8, 38.8, 32.3, 29.3, 23.7, 22.5, 21.2 ppm.

^{1}H NMR [500 MHz, $(CD_3)_2SO$]*:* δ 12.04 (s, 1H), 8.90 (s, 1H), 6.78 (d, *J* = 8.3 Hz, 1H), 6.63 (d, *J* = 2.5 Hz, 1H), 6.48 (dd, *J* = 8.1, 2.6, 1H), 2.71 (ddd, *J* = 16.3, 4.7, 1.4 Hz, 1H), 2.63 – 2.56 (m, 1H), 2.14 – 2.03 (m, 3H), 1.97 – 1.81 (m, 2H), 1.55 – 1.50 (m, 1H), 1.43 (dd, *J* = 12.0, 1.5 Hz, 1H), 1.26 (ddd, *J* = 13.4, 13.4, 4.1 Hz, 1H), 1.20 (s, 3H), 1.04 – 0.99 (m, 1H), 1.03 (s, 3H) ppm;

^{13}C NMR [125 MHz, $(CD_3)_2SO$]*:* δ 178.5, 155.1, 148.9, 129.5, 125.1, 113.0, 111.6, 51.9, 43.0, 39.1, 38.1, 37.1, 30.7, 28.4, 23.0, 21.0, 19.7 ppm.

Synthetic Procedures and Characterization Data

Part 1: A Diels–Alder Disconnection

optimized route

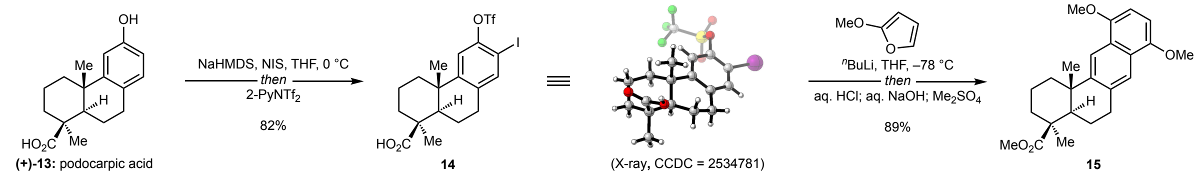

select route scouting

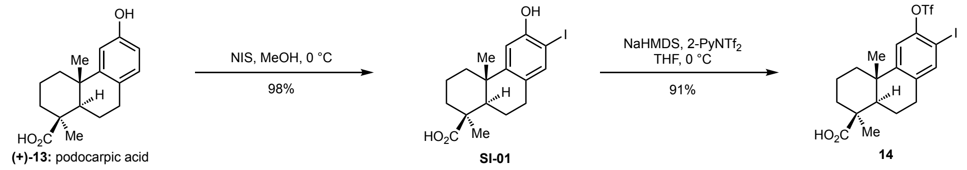

notes

• two-step route abandoned once conditions to telescope both steps were developed

• reproducibility issues were observed when employing NaH during the triflation step (tentatively attributed to decomposition of triflating reagents)

• purification of triflate was more difficult with PhNTf$_2$ versus 2-PyNTf$_2$

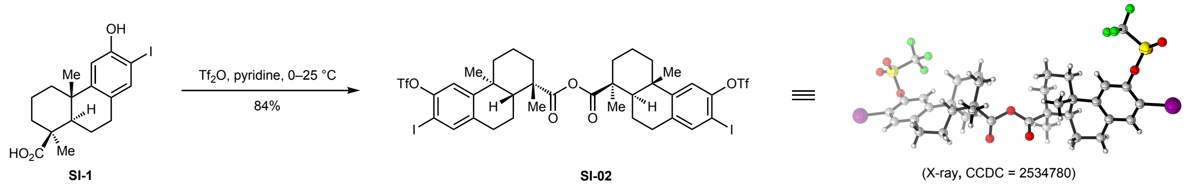

notes

• undesired product generated, exploratory attempts to use the triflate dimer in further reactions were unfruitful

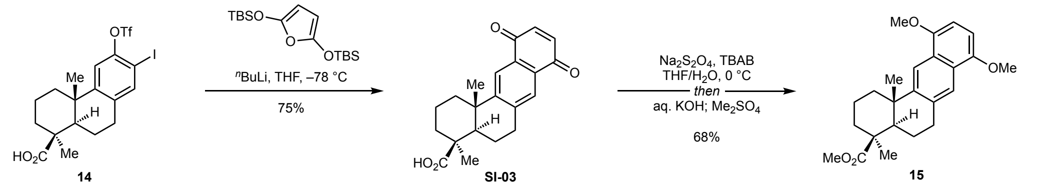

notes

• difficulty scaling Diels–Alder reaction beyond 1.5 g of aryl triflate – on these scales mixtures of **SI-3** and **SI-4** (drawn below) were observed – while not necessarily a dead end, route abandoned once conditions employing 2-methoxyfuran as diene were developed

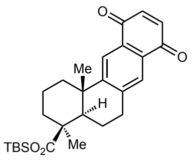

SI-04 (tentative assignment, not fully characterized)

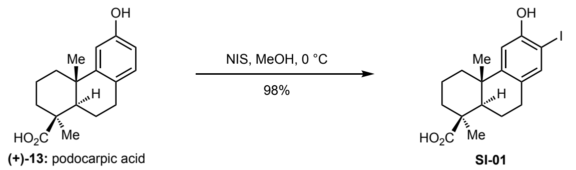

To a 1000-mL one-neck round-bottom flask open to air and equipped with a stir bar was added (+)-podocarpic acid **13** (10.1 g, 36.7 mmol, 1.00 equiv) and methanol (245 mL, 0.150 M). The flask was submerged in an ice/water bath, and *N*-iodosuccinimide (8.68 g, 38.6 mmol, 1.05 equiv) was added as one portion over approximately 30 seconds. The flask was stoppered, and the reaction was stirred for 1.5 hours by which time complete consumption of starting material was observed via ^{1}H NMR aliquot (CD_3OD). The reaction was quenched with 10% aqueous sodium dithionite, diluted with ethyl acetate, washed with water (×1) and brine (×1), dried over magnesium sulfate, filtered, and concentrated under reduced pressure. The crude yellow oil was dissolved in the minimum volume of toluene and purified via flash column chromatography (ratio of silica to crude mass = 10:1, eluted with diethyl ether).

Yield: 14.4 g, 36.1 mmol, 98%;

Appearance: off-white solid;

R*f*: 0.26 (2:3 diethyl ether:petroleum ether);

^{1}H NMR (500 MHz, CD_3OD): δ 7.31 (s, 1H), 6.75 (s, 1H), 2.75 (dd, *J* = 16.0, 4.7 Hz, 1H), 2.64 (ddd, *J* = 14.7, 14.7, 6.0 Hz, 1H), 2.22 – 2.12 (m, 3H), 2.09 – 1.92 (m, 2H), 1.59 (ddd, *J* = 13.5, 2.6, 2.6 Hz, 1H), 1.49 (d, *J* = 12.2 Hz, 1H), 1.36 (ddd, *J* = 13.4, 13.4, 4.1 Hz, 1H), 1.27 (s, 3H), 1.11 (s, 3H), 1.07 (app dd, *J* = 13.5, 4.2 Hz, 1H) ppm;

^{13}C NMR (125 MHz, CD_3OD): δ 181.4, 155.8, 151.2, 140.2, 130.0, 112.8, 81.6, 53.9, 44.8, 40.6, 39.8, 38.7, 31.8, 29.2, 23.7, 22.3, 21.1 ppm;

IR: 3222, 2959, 2929, 1692, 1479, 1402, 1263, 1215, 1191, 1080, 975, 889, 742 cm^{-1};

HRMS (ESI): calculated for $[C_{17}H_{21}IO_3+H]^+$: 401.0608, found: 401.0594;

$[\alpha]_D^{25}$: +112.8° (c = 1.00, CH_3OH).

For convenience, we also report ^{1}H NMR data in $CDCl_3$.

^{1}H NMR (500 MHz, $CDCl_3$): δ 7.33 (s, 1H), 6.89 (s, 1H), 5.01 (br s, 1H), 2.82 (dd, J = 16.1, 5.4 Hz, 1H), 2.70 (ddd, *J* = 12.3, 12.3, 6.2 Hz 1H), 2.26 (d, *J* = 13.4 Hz, 1H), 2.22 – 2.11 (m, 2H), 2.00 (ddd, *J* = 14.4, 12.8, 5.3 Hz, 2H), 1.63 (d, *J* = 14.4 Hz, 1H), 1.51 (d, J = 12.3 Hz, 1H), 1.38 (ddd, *J* = 13.6, 13.6, 3.9 Hz, 1H), 1.33 (s, 3H), 1.11 (s, 3H), 1.08 (app dd, J = 13.4, 4.4 Hz, 1H) ppm.

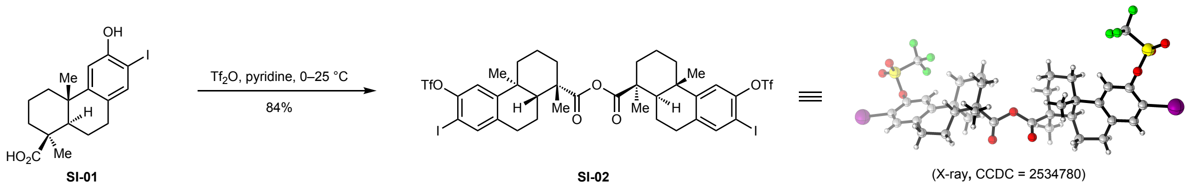

To a flame-dried 100-mL one-neck round-bottom flask under argon and equipped with a stir bar was added aryl iodide **SI-01** (0.200 g, 0.729 mmol, 1.00 equiv) and anhydrous pyridine (30 mL, 0.024 M). The flask was submerged in an ice/water bath, and trifluoromethanesulfonic anhydride (0.150 mL, 0.911 mmol, 1.25 equiv) was added dropwise. The reaction was stirred for 10 minutes, the ice/water bath removed, and the reaction was stirred for an additional 16 hours. The reaction was diluted with ethyl acetate, washed with water (×1), 2 N HCl (×1), saturated aqueous sodium bicarbonate (×1), and brine (×1), dried over magnesium sulfate, filtered, and concentrated under reduced pressure. The crude residue was dissolved in the minimum volume of dichloromethane and purified via flash column chromatography (ratio of silica to crude mass = 10:1, eluted with 9:1 hexane:ethyl acetate).

Yield: 0.321 g, 0.306 mmol, 84%;

Appearance: white solid;

R*f*: 0.36 (1:9 ethyl acetate:petroleum ether);

^{1}H NMR (500 MHz, $CDCl_3$): δ 7.57 (s, 2H), 7.17 (s, 2H), 2.79 (ddd, *J* = 17.6, 12.8, 6.2 Hz, 2H), 2.8 – 2.75 (m, 2H), 2.30 (d, *J* = 13.7 Hz, 2H), 2.23 – 2.15 (m, 4H), 2.11 – 1.92 (m, 4H), 1.71 (d, *J* = 14.2 Hz, 2H), 1.58 (d, *J* = 12.2 Hz, 2H), 1.44 (app dd, *J* = 13.5, 4.2 Hz, 2H), 1.40 (s, 6H), 1.17 (s, 8H) ppm;

^{13}C NMR (125 MHz, $CDCl_3$): δ 172.8, 150.7, 148.7, 140.9, 137.6, 119.4, 118.9 (q, *J* = 320.7 Hz), 85.2, 52.1, 45.7, 38.9, 37.6, 31.0, 27.9, 24.1, 20.5, 19.7 ppm;

^{19}F NMR (470 MHz, $CDCl_3$): δ −164.1 ppm;

IR: 2968, 2930, 2849, 1805, 1743, 1472, 1419, 1384, 1217, 1205, 1135, 1004, 982, 970, 958, 936, 911, 891, 849, 834, 768, 725, 650 cm^{-1};

HRMS (ESI): calculated for $[C_{36}H_{37}O_9F_6I_2S_2+H]^+$: 1047.0024, found = 1047.0024;

$[\alpha]_D^{25}$: +87.6° (c = 1.00, CH_3OH).

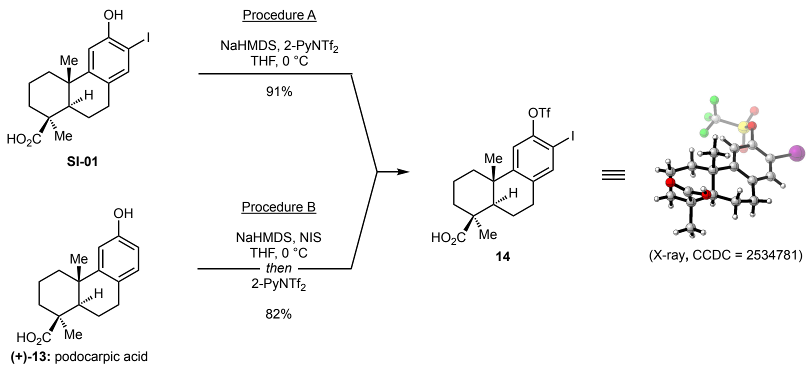

Procedure A

To a flame-dried 500-mL one-neck round-bottom flask under argon and equipped with a stir bar was added aryl iodide **SI-01** (8.430 g, 21.06 mmol, 1.00 equiv) and anhydrous tetrahydrofuran (195 mL, 0.108 M). The flask was submerged in an ice/water bath, and sodium hexamethyldisilazide (31.6 mL, 63.2 mmol, 2 M in tetrahydrofuran, 3.00 equiv) was added via syringe pump over 15 minutes. The resulting slurry was stirred vigorously for an additional 5 minutes, then *N*-(2-pyridyl)bis(trifluoromethanesulfonimide) (11.31 g, 31.6 mmol, 1.50 equiv) was added as one portion over approximately 10 seconds. The reaction was stirred for 2 hours, quenched with 2 N HCl, and extracted with diethyl ether (×3). The combined organics were washed with brine (×1), dried over magnesium sulfate, filtered, and partially concentrated under reduced pressure to afford a pale-yellow solution with white precipitate. The mixture was filtered, concentrated under reduced pressure, dissolved in diethyl ether (approximately 150 mL), filtered, and concentrated under reduced pressure again. The residue was dissolved in the minimum volume of diethyl ether (trace insoluble impurities) and purified via flash column chromatography (ratio of silica to crude mass = 10:1, eluted with 2:1 hexane:diethyl ether).

Yield: 10.54 g, 11.21 mmol, 94%.

Procedure B

To a flame-dried 250-mL one-neck round-bottom flask under argon and equipped with a stir bar was added (+)-podocarpic acid **13** (1.00 g, 3.63 mmol, 1.00 equiv) and anhydrous tetrahydrofuran (70 mL, 0.052 M). The flask was submerged in an ice/water bath, and sodium hexamethyldisilazide (3.75 mL, 10.9 mmol, 2 M in tetrahydrofuran, 3.00 equiv) was added dropwise via syringe pump over 10 minutes. *N*-iodosuccinimide (0.902 g, 4.01 mmol, 1.10 equiv) was added as one portion over approximately 10 seconds, the reaction was stirred vigorously for 2 hours, and *N*-(2-pyridyl)bis(trifluoromethanesulfonimide) (1.34 g, 5.45 mmol, 1.50 eq) was added as one portion over approximately 10 seconds. The reaction was stirred for 30 minutes, quenched with 2 N HCl, extracted with diethyl ether (×3), washed with brine (×1), dried over magnesium sulfate, filtered, and partially concentrated under reduced pressure to afford a pale-yellow solution with white precipitate. The mixture was filtered, concentrated under reduced pressure, dissolved in diethyl ether (approximately 100 mL), filtered, and concentrated under reduced pressure again. The crude residue was dissolved in the minimum volume of diethyl ether (trace insoluble impurities) and purified via flash column chromatography (ratio of silica to crude mass = 10:1, eluted with 2:1 hexane:diethyl ether).

Yield: 1.59 g, 2.99 mmol, 82%;

Appearance: white solid;

R*f*: 0.22 (dichloromethane);

^{1}H NMR (600 MHz, $CDCl_3$): δ 11.21 (br s, 1H), 7.56 (s, 1H), 7.15 (s, 1H), 2.89 (dd, *J* = 17.1, 4.6 Hz, 1H), 2.75 (ddd, *J* = 17.8, 12,8, 6.5 Hz, 1H), 2.27 (d, *J* = 13.6 Hz, 1H), 2.19 (dd, *J* = 14.0, 6.4 Hz, 1H), 2.14 (d, *J* = 12.8 Hz, 1H), 2.09 – 1.95 (m, 2H), 1.69 – 1.63 (m, 1H), 1.52 (dd, *J* = 12.3, 1.6 Hz, 1H), 1.40 (ddd, *J* = 13.3, 13.3, 4.0 Hz, 1H), 1.34 (s, 3H), 1.13 – 1.06 (m, 4H) ppm;

^{13}C NMR (150 MHz, $CDCl_3$): δ 183.3, 150.9, 148.6, 140.9, 138.0, 119.4, 118.9 (q, *J* = 320.7 Hz), 85.0, 52.0, 43.9, 39.0 (2 coincident peaks), 37.2, 31.0, 28.7, 23.2, 20.5, 19.8 ppm;

^{19}F NMR (470 MHz, $CDCl_3$) δ −164.0 ppm;

IR: 2964, 2936, 2853, 1693, 1473, 1423, 1208, 1137, 911, 854, 837, 733 cm^{-1};

HRMS (ESI): calculated for $[C_{18}H_{20}F_3IO_5S{-}H]^-$: 530.9956, found: 530.9982;

$[\alpha]_D^{25}$: +92.5° (c = 1.00, CH_3OH).

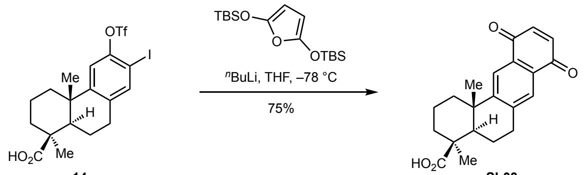

14 **SI-03**

Conducted according to a modification of related procedures reported by Newton.[20, 23] To a flame-dried 250-mL two-neck round-bottom flask under argon and equipped with a stir bar and a low-temperature glass thermometer was added aryl triflate **14** (1.53 g, 2.88 mmol, 1.00 equiv) and anhydrous tetrahydrofuran (58 mL, 0.050 M). The flask was submerged in a dry ice/acetone bath and 2,5-bis((*tert*-butyldimethylsilyl)oxy)furan[23] (1.89 g, 5.76 mmol, 2.00 equiv) was added as a solution in anhydrous tetrahydrofuran (1 mL, 5.76 M). Freshly titrated[24] *n*-butyllithium (3.92 mL, 7.20 mmol, 1.83 M in tetrahydrofuran, 2.50 equiv) was added at such a rate (approximately 10 minutes) to maintain an internal reaction temperature between −78 and −50 °C. The reaction was stirred for an additional 10 minutes before being quenched with saturated aqueous ammonium chloride. The flask was warmed to room temperature and diluted with ethyl acetate. The layers were separated, and the organic layer was washed with 2 N HCl (×1) and brine (×1), dried over magnesium sulfate, filtered, and concentrated under reduced pressure. The crude residue was dissolved in the minimum volume of dichloromethane and purified via flash column chromatography (ratio of silica to crude mass = 10:1, eluted with a gradient of dichloromethane to 9:1 dichloromethane:ethyl acetate).

Yield: 0.731 g, 2.16 mmol, 75%;

Appearance: yellow solid;

R*f*: 0.39 (1:9 ethyl acetate:dichloromethane);

M.p.: 109.8 – 120.5 °C;

^{1}H NMR (500 MHz, $CDCl_3$): δ 11.22 (br s, 1H), 7.99 (s, 1H), 7.75 (s, 1H), 6.90 (app s, 2H), 3.08 (dd, J = 17.7, 4.8 Hz, 1H), 2.90 (ddd, J = 18.2, 12.6, 6.4 Hz, 1H), 2.43 (d, J = 12.7 Hz, 1H), 2.27 (dd, J = 23.9, 9.8 Hz, 2H), 2.14 – 2.00 (m, 2H), 1.69 (d, J = 14.2 Hz, 1H), 1.58 (d, J = 12.1 Hz, 1H), 1.41 (ddd, J = 13.6, 13.6, 3.8 Hz, 1H), 1.36 (s, 3H), 1.17 (s, 3H), 1.12 (ddd, J = 13.6, 13.6, 4.0 Hz, 1H) ppm;

^{13}C NMR (125 MHz, $CDCl_3$): δ 185.3, 185.3, 183.4, 154.9, 142.9, 139.1, 138.8, 130.0, 129.3, 127.7, 124.5, 52.2, 44.1, 39.6, 39.1, 37.2, 32.4, 28.8, 23.1, 20.5, 19.8 ppm;

IR: 2962, 1693, 1667, 1596, 1336, 1295, 1147, 1055, 908, 843, 728 cm^{-1};

HRMS (ESI): calculated for $[C_{21}H_{22}O_4{-}H]^-$: 337.1445, found: 337.1444;

$[\alpha]_D^{25}$: +115.9° (c = 0.200, CH_3OH).

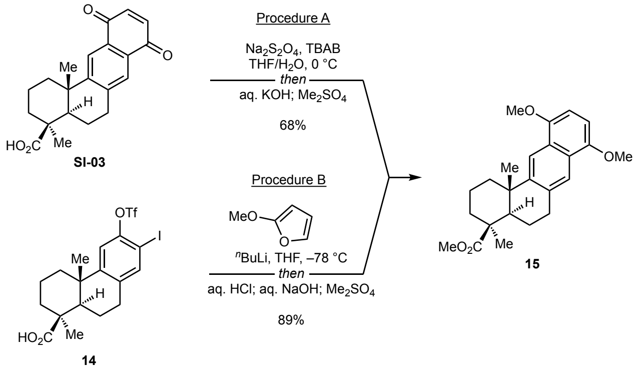

Note: Ester **15** was previously prepared in 11 steps from (*nat*)-*trans*-communic acid by Chahboun and Alvarez-Manzaneda[6] (the first 10 steps of which are presented earlier in this document within our summary of their synthesis of neopetrosiquinone A and B). Our characterization data agrees with those reported by Chahboun and Alvarez-Manzaneda.

<u>Procedure A</u>

To a 50-mL one-neck round-bottom flask open to air and equipped with a stir bar was added benzoquinone **SI-03** (0.471 g, 1.39 mmol, 1.00 equiv), tetrahydrofuran (7.0 mL, 0.20 M), and water (2.8 mL, 0.50 M). The flask was submerged in an ice/water bath, and tetrabutylammonium bromide (44.9 mg, 0.139 mmol, 0.10 equiv) was added, followed by sodium dithionite (1.45 g, 8.35 mmol, 6.00 equiv) as a solution in water (5 mL, 1.67 M). The flask was stoppered, the ice/water bath was removed, and the reaction was stirred for 30 minutes. The flask was resubmerged in the ice/water bath, and potassium hydroxide (1.80 g, 32.0 mmol, 23.0 equiv) was added as a solution in water (2.5 mL, 12.8 M). The ice/water bath was removed, and the reaction was stirred for 5 minutes. The flask was resubmerged in the ice/water bath, and dimethyl sulfate (3.51 g, 27.8 mmol, 20 equiv) was added dropwise. The ice/water bath was removed, and the reaction was stirred for 16 hours. The reaction was diluted with water and dichloromethane, the layers separated, and the aqueous later extracted with dichloromethane (×1). The combined organics were washed with brine (×1), dried over magnesium sulfate, filtered, and concentrated under reduced pressure. The crude residue

was dissolved in the minimum volume of toluene and purified via flash column chromatography (ratio of silica to crude mass = 10:1, eluted with 9:1 hexane:ethyl acetate).

Yield: 0.362 g, 0.947 mmol, 68%.

Procedure B

To a flame-dried 500-mL three-neck round-bottom flask under argon and equipped with a stir bar was added aryl triflate **14** (6.40 g, 12.0 mmol, 1.00 equiv) and anhydrous tetrahydrofuran (120 mL, 0.16 M). The flask was submerged in a dry ice/acetone bath, and 2-methoxyfuran (1.65 mL, 18.0 mmol, 1.50 equiv) was added via syringe as one portion. Freshly titrated[24] *n*-butyllithium (13.5 mL, 32.4 mmol, 2.2 molar in hexane, 2.70 equiv) was added via syringe pump over 30 minutes. The dry ice/acetone bath was removed, and the reaction was stirred for an additional 5 minutes. The reaction was quenched with 2 N HCl (30 mL, 5.00 equiv) and immediately basified to a pH of 12 with a 20% aqueous solution of sodium hydroxide (approximately 40 mL, pH measured using pH paper range 1–13). Dimethyl sulfate (22.8 mL, 239.6 mmol, 20.0 equiv) was added in one portion, the flask was stoppered, and the solution was stirred for an additional 16 hours. The reaction was diluted with dichloromethane, the layers separated, and the aqueous later extracted with dichloromethane (×1). The combined organics were washed with brine (×1), dried over magnesium sulfate, filtered, and concentrated under reduced pressure. The crude residue was dissolved in the minimum volume of dichloromethane and purified via flash column chromatography (ratio of silica to crude mass = 10:1, eluted with 4:1 hexane:diethyl ether).

Yield: 4.09 g, 10.7 mmol, 89%;

Appearance: off-white solid;

$\mathbf{R}_f$: 0.34 (1:9 ethyl acetate:petroleum ether);

^{1}H NMR (500 MHz, $CDCl_3$): δ 8.12 (s, 1H), 7.87 (s, 1H), 6.58 (d, *J* = 8.2 Hz, 1H), 6.55 (d, *J* = 8.2 Hz, 1H), 3.93 (s, 6H), 3.68 (s, 3H), 3.18 (dd, *J* = 16.7, 4.6 Hz, 1H), 2.99 (ddd, *J* = 17.3, 12.9, 6.2 Hz, 1H), 2.55 (d, J = 13.0 Hz, 1H), 2.31 (d, *J* = 13.5 Hz, 1H), 2.25 (dd, *J* = 13.6, 6.0 Hz, 1H), 2.13 – 2.01 (m, 2H), 1.73 – 1.61 (m, 2H), 1.53 (ddd, *J* = 13.4, 13.4, 3.2 Hz, 1H), 1.31 (s, 3H), 1.15 – 1.09 (m, 4H) ppm;

^{13}C NMR (125 MHz, $CDCl_3$): δ 178.1, 149.5, 149.1, 147.5, 134.6, 125.3, 124.8, 121.1, 118.3, 102.4, 101.8, 55.8, 55.7, 53.2, 51.4, 44.3, 40.1, 39.2, 37.8, 32.6, 28.7, 23.9, 21.4, 20.3 ppm;

IR: 2937, 2934, 1724, 1597, 1459, 1434, 1355, 1326, 1251, 1173, 1142, 1110, 1092, 1032, 975, 909, 797, 724 cm^{-1};

HRMS (ESI): calculated for $[C_{24}H_{30}O_4]^-$: 382.2139, found: 382.2139;

$[\alpha]_D^{25}$: +99.0° (c = 0.950, $CHCl_3$).

Part 2: Cyclozonarone and Neopetrosiquinones A and B

Cyclozonarone

optimized route

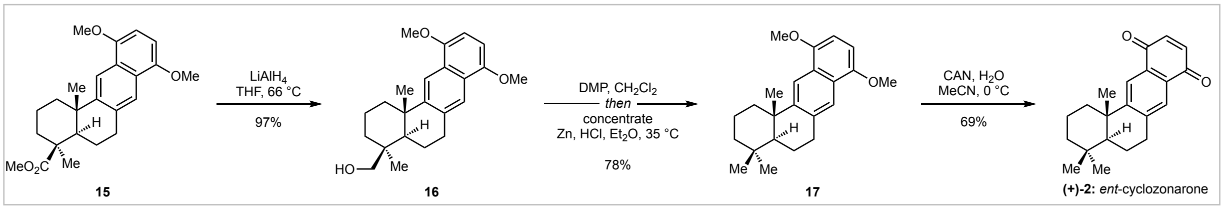

select route scouting

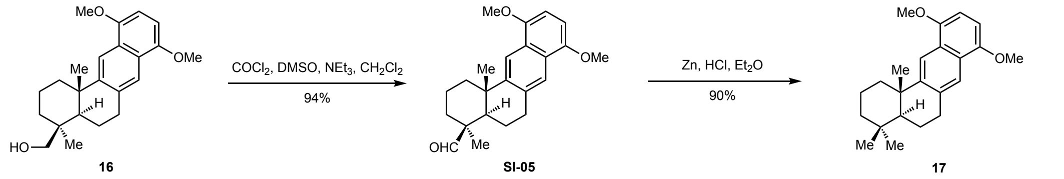

notes

- this two-step route was abandoned once conditions to conduct a one-pot deoxygenation protocol were developed
- a one-step Wolff–Kishner protocol for the conversion of **SI-05** to **17** was also developed (not shown), however lower yields were observed relative to the Clemmensen reduction protocol shown above
- a two-step deoxygenation protocol for the conversion of **SI-05** to **17** was also developed (not shown), proceeding via thioacetal formation then Mozingo reduction with Raney Ni, however this was also dropped in favor of the shorter Clemmensen reduction protocol

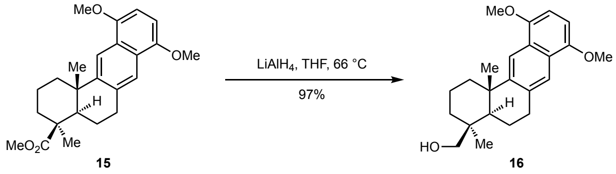

To a flame-dried, 500-mL one-neck round-bottom flask under argon and equipped with a stir bar was added anhydrous tetrahydrofuran (250 mL, 0.500 M). The flask was submerged in an ice/water bath, and lithium aluminum hydride (4.76 g, 125.4 mmol, 6.00 equiv) was added as one portion. To a separate flame-dried, 100-mL one-neck round-bottom flask under argon was added ester **15** (8.00 g, 20.9 mmol, 1.00 equiv) and anhydrous tetrahydrofuran (50 mL, 0.420 M). This solution was transferred dropwise via syringe into the flask containing the lithium aluminum hydride slurry. The ice/water bath was replaced with a heating mantle, and the flask was equipped with a high-efficiency air condenser and warmed to 66 °C for 7 hours. The flask was resubmerged in an ice/water bath and quenched with 2 N HCl, extracted with ethyl acetate (×3), washed with brine (×1), dried over magnesium sulfate, filtered, and concentrated under reduced pressure. The residue was dissolved in the minimum volume of dichloromethane and purified via flash chromatography (ratio of silica to crude mass = 10:1, eluted with a gradient of 100% hexane to 7:3 hexane:ethyl acetate).

Yield: 7.196 g, 20.3 mmol, 97%;

Appearance: white solid;

R*f*: 0.38 (7:3 hexane:ethyl acetate);

^{1}H NMR (500 MHz, $CDCl_3$): δ 8.08 (s, 1H), 7.87 (s, 1H), 6.59 (d, *J* = 8.7 Hz, 1H), 6.56 (d, *J* = 8.2 Hz, 1H), 3.94 (s, 3H), 3.93 (s, 3H), 3.92 (d, *J* = 11.7 Hz, 1H), 3.60 (d, *J* = 10.9 Hz, 1H), 3.19 (dd, J = 17.3, 6.5 Hz, 1H), 3.05 (ddd, *J* = 17.8, 11.2, 7.7 Hz, 1H), 2.59 (d, *J* = 12.8 Hz, 1H), 2.05 (dd, *J* = 12.9, 7.9 Hz, 1H), 1.93 (d, *J* = 13.6 Hz, 1H), 1.85 – 1.75 (m, 2H), 1.73 – 1.67 (m, 1H), 1.61 – 1.58 (m, 2H), 1.25 (s, 3H), 1.08 (s, 3H), 1.05 (dd, *J* = 13.6, 4.2 Hz, 1H) ppm;

^{13}C NMR (125 MHz, $CDCl_3$): δ 149.6, 149.3, 149.1, 134.2, 125.2, 124.8, 121.1, 116.9, 102.4, 101.9, 65.5, 55.8, 55.7, 51.3, 39.5, 39.0, 38.4, 35.8, 31.1, 26.9, 26.3, 19.4, 19.2 ppm;

IR: 3584, 2977, 2912, 2360, 1594, 1455, 1248, 1091, 1048, 1018, 897, 811, 724 cm^{-1};

HRMS (ESI): calculated for $[C_{23}H_{30}O_3{-}H]^-$: 353.2122, found: 353.2109;

$[\alpha]_D^{25}$: +37.6° (c = 0.860, $CHCl_3$).

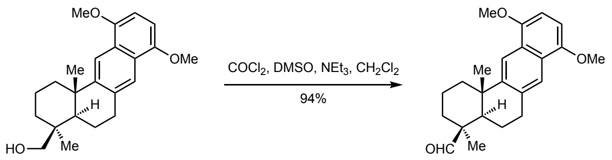

16　　SI-05

To a flame-dried, 10-mL two-neck round-bottom flask under nitrogen and equipped with a stir bar was added anhydrous dimethyl sulfoxide (0.116 mL, 1.63 mmol, 6.00 equiv) and anhydrous dichloromethane (1.4 mL, 0.20 M). The flask was submerged in a dry ice/acetone bath, and oxalyl chloride (0.071 mL, 0.82 mmol, 3.00 equiv) was added dropwise. The reaction was stirred for 5 minutes, then a room temperature solution of alcohol **16** (96.5 mg, 0.272 mmol, 1.00 equiv) in anhydrous dichloromethane (1.4 mL, 0.20 M) was added dropwise via syringe (alcohol **16** is poorly soluble in anhydrous dichloromethane at 0 °C). The reaction was stirred for 15 minutes, then anhydrous triethylamine (0.38 mL, 2.7 mmol, 10.0 equiv) was added. The reaction was stirred for a further 20 minutes before being removed from the dry ice/acetone bath and stirred for an additional 15 minutes. The reaction was quenched with saturated aqueous ammonium chloride, extracted with dichloromethane (×3), washed with brine (×1), dried over magnesium sulfate, filtered, and concentrated under reduced pressure. The residue was dissolved in the minimum volume of toluene and purified via flash chromatography (ratio of silica to crude mass = 10:1, eluted with 9:1 hexane:ethyl acetate).

Yield: 90.6 mg, 0.252 mmol, 94%;

Appearance: colorless oil;

R*f*: 0.24 (19:1 hexane:ethyl acetate);

^{1}H NMR (500 MHz, $CDCl_3$): δ 9.87 (s, 1H), 8.11 (s, 1H), 7.90 (s, 1H), 6.59 (d, *J* = 8.3 Hz, 1H), 6.56 (d, *J* = 8.3 Hz, 1H), 3.93 (s, 3H), 3.93 (s, 3H), 3.24 (dd, *J* = 16.8, 5.7 Hz, 1H), 3.07 (ddd, *J* = 17.7, 12.1, 7.1 Hz, 1H), 2.56 (d, *J* = 12.9 Hz, 1H), 2.28 – 2.24 (m, 2H), 2.11 (ddd, *J* = 19.2, 12.7, 6.3 Hz, 1H), 1.87 – 1.68 (m, 3H), 1.55 (ddd, *J* = 13.3, 13.3, 4.0 Hz, 1H), 1.13 (s, 3H), 1.13 (s, 3H), 1.11 – 1.07 (m, 1H) ppm;

^{13}C NMR (125 MHz, $CDCl_3$): δ 205.9, 149.5, 149.0, 146.9, 133.8, 125.3, 124.9, 121.3, 117.7, 102.5, 102.0, 55.8, 55.7, 52.1, 48.9, 39.0, 38.7, 34.1, 31.6, 24.8, 24.4, 19.4, 19.2 ppm;

IR: 2934, 2832, 1713, 1596, 1460, 1435, 1353, 1328, 1275, 1252, 1204, 1157, 1111, 1092, 1057, 973, 900, 797, 723 cm^{-1};

HRMS (ESI): calculated for $[C_{23}H_{28}O_3]^+$: 352.2033, found: 352.2035;

$[\alpha]_D^{25}$: +52.6° (c = 0.740, $CHCl_3$).

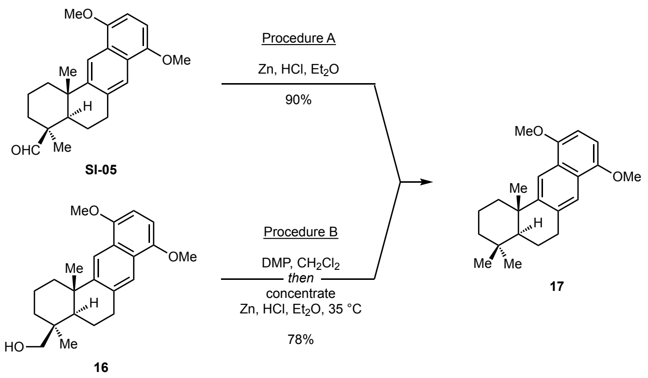

Procedure A

To a 50-mL round-bottom flask open to air equipped with a stir bar was added aldehyde **SI-05** (15.0 mg, 0.0426 mmol, 1.00 equiv), diethyl ether (10.0 mL, 0.004 M), and zinc dust (22.3 mg, 0.341 mmol, 8.00 equiv). The flask was submerged in an ice/water bath, and concentrated HCl (0.0710 mL, 0.852 mmol, 20.0 equiv) was added dropwise (significant bubbling). The flask was stoppered, and the reaction was stirred vigorously for 1.5 hours. Zinc solids were removed via filtration, and the reaction was extracted with diethyl ether (×3), washed with brine (×1), dried over magnesium sulfate, filtered, and concentrated under reduced pressure. The residue was dissolved in the minimum volume of hexane and purified via flash chromatography (ratio of silica to crude mass = 10:1, eluted with 9:1 hexane:diethyl ether).

Yield: 13.0 mg, 0.0384 mmol, 90%.

Procedure B

To a 100-mL round-bottom flask open to air and equipped with a stir bar was added alcohol **16** (190 mg, 0.536 mmol, 1.00 equiv) and dichloromethane (25 mL, 0.021 M). Dess–Martin periodinane (341 mg, 0.804 mmol, 1.50 equiv) was added as one portion over approximately 10 seconds, and the reaction was stirred at room temperature for 1 hour. The stir bar was removed, and the solvent was concentrated under reduced pressure. The stir bar was re-added, the residue was dissolved in diethyl ether (50 mL, 0.010 M), and zinc dust (1.40 g, 21.4 mmol, 40.0 equiv) was added as one portion. Concentrated HCl (4.40 mL, 53.6 mmol, 100 equiv) was added dropwise, resulting in significant bubbling and partial solvent evaporation (approximately 10 mL). A high-efficiency air condenser was attached, and the flask was warmed on a heating mantle at 35 °C for 3 hours, resulting in partial solvent evaporation (approximately 10 mL). Zinc solids were removed via filtration, and the reaction was extracted with diethyl ether (×3), washed with brine (×1),

dried over magnesium sulfate, filtered, and concentrated under reduced pressure. The residue was dissolved in the minimum volume of hexane and purified via flash chromatography (ratio of silica to crude mass = 10:1, eluted with 9:1 hexane:diethyl ether).

Yield: 0.142 g, 0.420 mmol, 78%;

Appearance: colorless oil;

R*f*: 0.29 (49:1 hexane:ethyl acetate);

^{1}H NMR (500 MHz, $CDCl_3$): δ 8.09 (s, 1H), 7.88 (s, 1H), 6.59 (d, *J* = 8.2 Hz, 1H), 6.57 (d, *J* = 8.3 Hz, 1H), 3.95 (s, 3H), 3.94 (s, 3H), 3.21 (dd, *J* = 17.0, 6.8 Hz, 1H), 3.11 – 3.04 (m, 1H), 2.56 (d, *J* = 12.7 Hz, 1H), 1.99 – 1.93 (m, 1H), 1.86 – 1.78 (m, 2H), 1.71 – 1.66 (m, 1H), 1.60 (dd, *J* = 13.0, 3.4 Hz, 1H), 1.52 (d, *J* = 13.1 Hz, 1H), 1.42 (dd, *J* = 12.4, 2.7 Hz, 1H), 1.31 – 1.29 (m, 1H), 1.27 (s, 3H), 0.99 (s, 3H), 0.99 (s, 3H) ppm;

^{13}C NMR (125 MHz, $CDCl_3$): δ 149.8, 149.7, 149.1, 134.7, 125.2, 124.7, 121.0, 116.5, 102.3, 101.8, 55.8, 55.7, 50.3, 42.0, 39.4, 38.5, 33.8, 33.5, 30.5, 25.2, 21.9, 19.5, 19.4 ppm;

IR: 2939, 2832, 1597, 1460, 1433, 1355, 1329, 1274, 1249, 1206, 1191, 1175, 1125, 1105, 1094, 975, 897, 794, 723 cm^{-1};

HRMS (ESI): calculated for $[C_{23}H_{30}O_2]^+$: 338.2240, found: 338.2238;

$[\alpha]_D^{25}$: +46.4° (c = 0.380, $CHCl_3$).

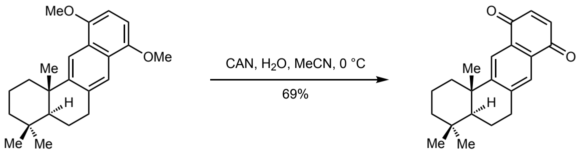

17 **(+)-2:** *ent*-cyclozonarone

To a 100-mL one-neck round-bottom flask open to air and equipped with a stir bar was added **17** (80.0 mg, 0.236 mmol, 1.00 equiv), acetonitrile (15.0 mL, 0.0157 M), and deionized water (5.00 mL, 0.0472 M). The flask was submerged in an ice/water bath, and ceric ammonium nitrate (389 mg, 0.709 mmol, 3.00 equiv) was added as one portion over approximately 5 seconds. The flask was stoppered, and the reaction was stirred for 30 minutes, extracted with ethyl acetate (×3), washed with brine (×1), dried over magnesium sulfate, filtered, and concentrated under reduced pressure. The residue was dissolved in the minimum volume of hexane and purified via flash chromatography (ratio of silica to crude mass = 10:1, eluted with 9:1 hexane:diethyl ether).

Yield: 50.2 mg, 0.163 mmol, 69%;

Appearance: yellow oil;

R*f*: 0.29 (1:19 ethyl acetate:petroleum ether);

^{1}H NMR (500 MHz, $CDCl_3$): δ 7.98 (s, 1H), 7.73 (s, 1H), 6.90 (d, *J* = 10.4 Hz, 1H), 6.88 (d, *J* = 10.4 Hz, 1H), 3.09 (dd, *J* = 18.3, 7.0 Hz, 1H), 2.96 (ddd, *J* = 18.7, 11.1, 7.6 Hz, 1H), 2.43 (d, *J* = 12.8 Hz, 1H), 1.95 (dd, *J* = 13.5, 7.9

Hz, 1H), 1.83 – 1.70 (m, 2H), 1.69 – 1.64 (m, 1H), 1.51 (d, *J* = 14.8 Hz, 1H), 1.42 (ddd, *J* = 13.1, 13.1, 3.9 Hz, 1H), 1.31 (dd, *J* = 12.6, 2.6 Hz, 1H), 1.24 (dd, *J* = 13.6, 4.0 Hz, 1H), 1.20 (s, 3H), 0.97 (s, 3H), 0.95 (s, 3H) ppm;

^{13}C NMR (125 MHz, $CDCl_3$): δ 185.4, 185.4, 157.1, 143.0, 139.0, 138.7, 129.9, 129.2, 127.5, 123.2, 49.9, 41.6, 38.8, 38.7, 33.7, 33.3, 30.8, 24.6, 21.8, 19.2, 18.7 ppm;

IR: 2926, 1669, 1596, 1335, 1310, 1295, 1145, 1054, 843 cm^{-1};

HRMS (ESI): calculated for $[C_{21}H_{24}O_2]^+$: 308.1771, found: 308.1763;

$[\alpha]_D^{25}$: +81.1° (c = 0.240, $CHCl_3$).

Neopetrosiquinone A

optimized route

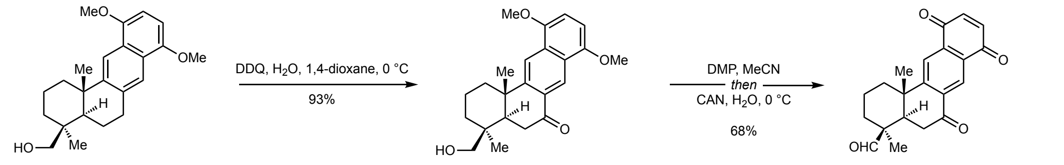

16 **18** **(+)-3:** *ent*-neopetrosiquinone A

select route scouting

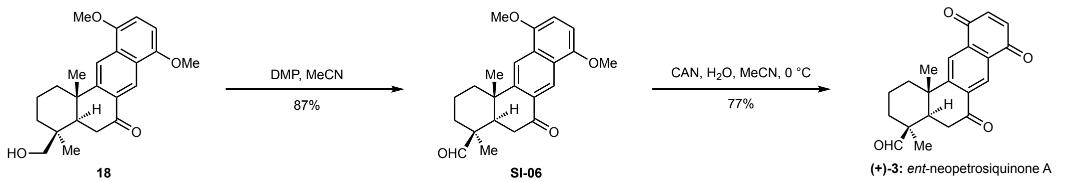

18 **SI-06** **(+)-3:** *ent*-neopetrosiquinone A

notes

• this two-step route abandoned once conditions to telescope both steps were developed

• Chahboun and Alvarez-Manzaneda's conversion of ketone **18** to **(+)-3:** *ent*-neopetrosiquinone A (summarized earlier in this document) proceeds via 1,4-benzoquinone deprotection then alcohol oxidation, however, our attempts to telescope this sequence were met with limited success, prompting us to explore the reverse order of operations (i.e., oxidation then deprotection)

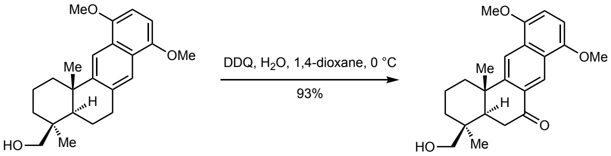

16 **18**

Note: Ketone **18** was previously prepared in 14 steps from (*nat*)-*trans*-communic acid by Chahboun and Alvarez-Manzaneda[6] (presented earlier in this document within our summary of their synthesis of neopetrosiquinone A and B). Our characterization data agrees with those reported by Chahboun and Alvarez-Manzaneda.

Conducted according to a modification of related procedures reported by Harvey[25] and Shibasaki.[14] To a 500-mL one-neck round-bottom flask open to air and equipped with a large stir bar was added alcohol **16** (1.50 g, 4.23 mmol, 1.00 equiv), 1,4-dioxane (200 mL, 0.021 M), and deionized water (50 mL, 0.085 M). The flask was submerged in an ice/water bath, and 2,3-dichloro-5,6-dicyano-1,4-benzoquinone (2.88 g, 12.7 mmol, 3.00 equiv) was added as one portion over approximately 30 seconds. The flask was stoppered, and the reaction was stirred for 2 hours before being quenched with saturated aqueous sodium bicarbonate, extracted with ethyl acetate (×3), washed with brine (×1), dried over magnesium sulfate, filtered, and concentrated under reduced pressure. The residue was dissolved in the minimum volume of dichloromethane and purified via flash chromatography (ratio of silica to crude mass = 10:1, eluted with 4:1 hexane:ethyl acetate).

Yield: 1.44 g, 3.93 mmol, 93%;

Appearance: yellow oil;

R*f*: 0.29 (4:1 dichloromethane:ethyl acetate);

^{1}H NMR (500 MHz, $CDCl_3$): δ 8.95 (s, 1H), 8.14 (s, 1H), 6.77 (d, *J* = 8.5 Hz, 1H), 6.64 (d, *J* = 8.5 Hz, 1H), 3.96 (s, 3H), 3.95 (s, 3H), 3.67 (d, *J* = 10.3 Hz, 1H), 2.92 – 2.77 (m, 2H), 2.62 (d, *J* = 8.0 Hz, 1H), 2.08 (dd, *J* = 14.2, 3.9 Hz, 1H), 1.98 (dd, *J* = 13.8, 3.1 Hz, 1H), 1.82 – 1.74 (m, 3H), 1.30 (s, 3H), 1.27 – 1.25 (m, 1H), 1.14 – 1.10 (m, 1H), 1.07 (s, 3H) ppm;

^{13}C NMR (125 MHz, $CDCl_3$): δ 199.4, 151.4, 151.1, 149.1, 129.2, 128.8, 124.5, 123.8, 116.4, 106.2, 103.1, 65.1, 55.9, 55.8, 49.6, 38.7, 38.5, 38.4, 36.5, 35.3, 26.5, 24.8, 18.8 ppm;

IR: 3447, 2981, 2932, 2360, 1674, 1624, 1460, 1435, 1460, 1332, 1269, 1224, 1112, 1091, 906, 723 cm^{-1};

HRMS (ESI): calculated for $[C_{23}H_{28}O_4+H]^+$: 369.2060, found: 369.2058;

$[\alpha]_D^{25}$: −1.95° (c = 0.560, $CHCl_3$).

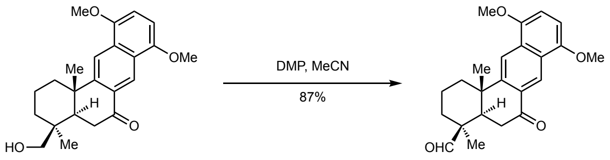

18 **SI-06**

To a 25-mL one-neck round-bottom flask open to air and equipped with a stir bar was added alcohol **18** (68.0 mg, 0.186 mmol, 1.00 equiv) and acetonitrile (6.2 mL, 0.030 M). Dess–Martin periodinane (157 mg, 0.371 mmol, 2.00 equiv) was added as one portion, the flask was stoppered, and the reaction was stirred for 20 minutes before being quenched with 10% aqueous sodium dithionite, extracted with ethyl acetate (×3), washed with brine (×1), dried over magnesium sulfate, filtered, and concentrated under reduced pressure. The residue was dissolved in the minimum volume of dichloromethane and purified via flash chromatography (ratio of silica to crude mass = 10:1, eluted with 4:1 hexane:ethyl acetate).

Yield: 54.4 mg, 0.162 mmol, 87%;

Appearance: yellow oil;

R*f*: 0.47 (4:1 dichloromethane:ethyl acetate)

^{1}H NMR (400 MHz, $CDCl_3$): δ 9.91 (s, 1H), 8.97 (s, 1H), 8.16 (s, 1H), 6.75 (d, *J* = 8.3 Hz, 1H), 6.63 (d, *J* = 8.3 Hz, 1H), 3.95 (s, 3H), 3.94 (s, 3H), 3.20 – 3.03 (m, 2H), 2.61 (ddd, *J* = 12.6, 4.1, 4.1 Hz, 1H), 2.27 – 2.17 (m, 2H), 1.87 – 1.75 (m, 2H), 1.69 (ddd, *J* = 12.7, 12.7, 4.9 Hz, 1H), 1.25 – 1.20 (m, 1H), 1.17 (s, 3H), 1.11 (s, 3H) ppm;

^{13}C NMR (100 MHz, $CDCl_3$): δ 204.8, 197.9, 151.0, 149.5, 149.0, 129.1, 128.6, 124.6, 123.9, 117.1, 106.3, 103.3, 55.8, 55.8, 49.7, 47.9, 38.4, 38.0, 35.8, 34.5, 23.8, 23.6, 19.2 ppm;

IR: 2934, 2875, 2855, 2829, 2720, 1756, 1711, 1674, 1627, 1586, 1460, 1432, 1399, 1379, 1334, 1292, 1269, 1241, 1220, 1199, 1166, 1137, 1115, 1094, 1044, 1033, 1015, 975, 905, 890, 856, 790, 763, 724, 701 cm^{-1};

HRMS (ESI): calculated for $[C_{23}H_{26}O_4+H]^+$: 367.1904, found: 367.1903;

[α]D[25]: +167° (c = 0.530, $CHCl_3$).

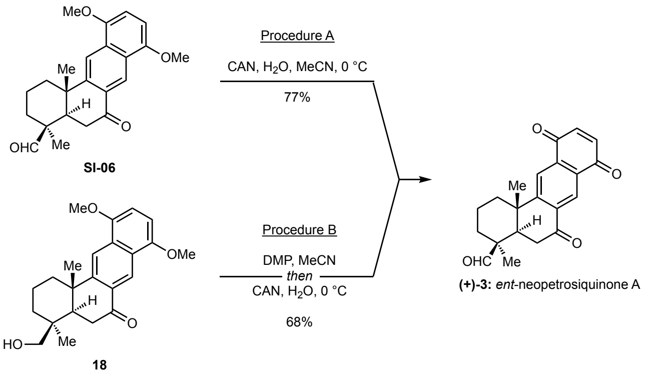

Procedure A

To a 10-mL one-neck round-bottom flask open to air and equipped with a stir bar was added aldehyde **SI-06** (15.0 mg, 0.0409 mmol, 1.00 equiv), acetonitrile (3.0 mL, 0.014 M), and deionized water (1.0 mL, 0.041 M). The flask was submerged in an ice/water bath, and ceric ammonium nitrate (67.3 mg, 0.123 mmol, 3.00 equiv) was added as one portion over approximately 5 seconds. The flask was stoppered, and the reaction was stirred for 30 minutes before being extracted with ethyl acetate (×3), washed with brine (×1), dried over sodium sulfate, filtered, and concentrated under reduced pressure. The residue was dissolved in the minimum volume of dichloromethane and purified via flash chromatography (ratio of silica to crude mass = 10:1, eluted with a gradient of 100% dichloromethane to 99:1 dichloromethane:ethyl acetate).

Yield: 10.6 mg, 0.032 mmol, 77%.

Procedure B

To a 25-mL one-neck round-bottom flask open to air and equipped with a stir bar was added alcohol **18** (19.8 mg, 0.054 mmol, 1.00 equiv), acetonitrile (3.0 mL, 0.018 M), and Dess-Martin periodinane (45.6 mg, 0.107 mmol, 2.00 equiv). The flask was stoppered, and the reaction was stirred for 20 minutes. The flask was submerged in an ice/water bath, and ceric ammonium nitrate (147 mg, 0.269 mmol, 5.00 equiv) in deionized water (0.5 mL, 0.54 M) was added. The reaction was stirred for an additional 20 minutes before being quenched with saturated aqueous sodium bicarbonate, extracted with ethyl acetate (×3), washed with brine (×1), dried over magnesium sulfate, filtered, and concentrated under reduced pressure. The residue was dissolved in the minimum volume of dichloromethane and purified via flash chromatography (ratio of silica to crude mass = 10:1, eluted with a gradient of 100% dichloromethane to 99:1 dichloromethane:ethyl acetate).

Yield: 12.3 mg, 0.037 mmol, 68%;

Appearance: yellow solid;

R*f*: 0.30 (99:1 dichloromethane:ethyl acetate);

^{1}H NMR (500 MHz, CD_2Cl_2): δ 9.88 (s, 1H), 8.63 (s, 1H), 8.15 (s, 1H), 7.01 (app s, 2H), 3.20 – 3.06 (m, 2H), 2.55 (ddd, *J* = 12.9, 3.9, 3.9 Hz, 1H), 2.55 – 2.18 (m, 2H), 1.85 – 1.80 (m, 2H), 1.65 – 1.59 (m, 1H), 1.26 – 1.22 (m, 1H), 1.18 (s, 3H), 1.13 (s, 3H) ppm;

^{13}C NMR (125 MHz, CD_2Cl_2): δ 204.8, 196.3, 184.8, 184.2, 159. 7, 139.7, 139.2, 135.2, 134.7, 130.6, 126.5, 123.7, 49.4, 48.0, 39.4, 37.7, 35.7, 34.7, 23.9, 23.0, 19.3 ppm;

IR: 2926, 2855, 1724, 1690, 1671, 1596, 1456, 1413, 1382, 1363, 1338, 1299, 1291, 1258, 1147, 843 cm^{-1};

HRMS (ESI): calculated for $[C_{21}H_{20}O_4+H]^+$: 337.1434, found: 337.1436;

$[\alpha]_D^{25}$: +39.8° (c = 1.53, $CHCl_3$).

Neopetrosiquinone B

optimized route

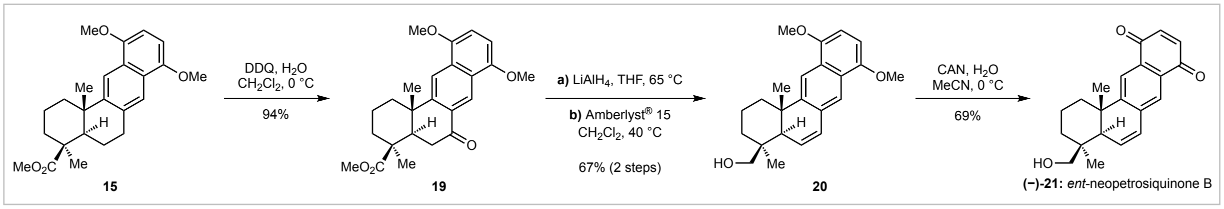

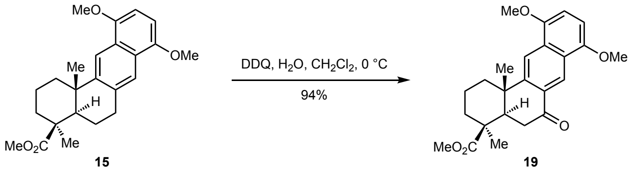

Conducted according to a modification of related procedures reported by Harvey[25] and Shibasaki.[14] To a 1000-mL one-neck round-bottom flask open to air and equipped with a large stir bar was added ester **15** (4.50 g, 11.8 mmol, 1.00 equiv), dichloromethane (450 mL), and deionized water (150 mL). The flask was submerged in an ice/water bath, and 2,3-dichloro-5,6-dicyano-1,4-benzoquinone (8.01 g, 35.3 mmol, 3.00 equiv) was added as one portion over approximately 30 seconds. The flask was stoppered, and the reaction was stirred for 2 hours before being quenched with saturated aqueous sodium bicarbonate, extracted with ethyl acetate (×3), washed with brine (×1), dried over magnesium sulfate, filtered, and concentrated under reduced pressure. The residue was dissolved in the minimum volume of dichloromethane and purified via flash chromatography (ratio of silica to crude mass = 10:1, eluted with 4:1 hexane:ethyl acetate).

Yield: 4.38 g, 11.05 mmol, 94%;

Appearance: yellow solid;

R_f: 0.30 (4:1 hexane:ethyl acetate);

^{1}H NMR (500 MHz, $CDCl_3$): δ 8.98 (s, 1H), 8.20 (s, 1H), 6.75 (d, J = 8.3 Hz, 1H), 6.63 (d, J = 8.3 Hz, 1H), 3.95 (s, 3H), 3.95 (s, 3H), 3.72 (s, 3H), 3.32 (dd, J = 18.0, 13.5 Hz, 1H), 3.08 (dd, J = 18.1, 3.4 Hz, 1H), 2.63 (d, J = 12.7 Hz, 1H), 2.34 (d, J = 13.6 Hz, 1H), 2.14 – 2.04 (m, 2H), 1.76 (app dt, J = 14.2, 3.6 Hz, 1H), 1.66 (app td, J = 13.3, 4.0 Hz, 2H), 1.29 (s, 3H), 1.17 – 1.20 (m, 4H) ppm;

^{13}C NMR (125 MHz, $CDCl_3$): δ 199.2, 177.3, 151.1, 150.1, 149.0, 129.3, 128.7, 124.6, 123.4, 117.8, 106.0, 103.1, 55.8, 55.8, 51.7, 50.1, 44.2, 39.2, 38.9, 38.2, 37.8, 28.2, 22.3, 19.9 ppm;

IR: 2938, 1724, 1682, 1626, 1589, 1460, 1434, 1341, 1333, 1270, 1241, 1224, 1143, 1114, 1091, 1030, 972, 908, 802, 725 cm^{-1};

HRMS (ESI): calculated for $[C_{24}H_{28}O_5+H]^+$: 397.2010, found: 397.2012;

$[\alpha]_D^{25}$: +48.2° (c = 1.00, $CHCl_3$).

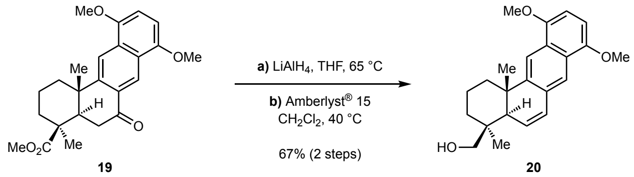

Note: Alcohol **20** was previously prepared in 16 steps from (*nat*)-*trans*-communic acid by Chahboun and Alvarez-Manzaneda[6] (presented earlier in this document within our summary of their synthesis of neopetrosiquinone A and B). Our characterization data agrees with those reported by Chahboun and Alvarez-Manzaneda.

To a flame-dried, 50-mL one-neck round-bottom flask under argon and equipped with a stir bar was added ketone **19** (0.206 g, 0.502 mmol, 1.00 equiv) and anhydrous tetrahydrofuran (6.65 mL, 0.100 M). The flask was submerged in an ice/water bath and lithium aluminum hydride (0.126 g, 3.33 mmol, 5.00 equiv) was added as one portion. The ice/water bath was replaced with a heating mantle, and the flask was equipped with a high-efficiency air condenser and warmed to 66 °C for 2 hours. The flask was submerged in an ice/water bath, quenched with 2 N HCl, extracted with dichloromethane (×2), and transferred into 100-mL one-neck round-bottom flask equipped with a stir bar. Amberlyst® 15 (0.300 g, approximately 1× crude mass) was added, and the flask was equipped with a high-efficiency air condenser and warmed to 40 °C. The reaction was stirred for 2 hours, filtered, and concentrated under reduced pressure. The residue was dissolved in the minimum volume of dichloromethane and purified via flash chromatography (ratio of silica to crude mass = 10:1, eluted with 7:3 hexane:ethyl acetate).

Yield: 0.123 mg, 0.349 mmol, 67%;

Appearance: yellow oil;

R*f*: 0.29 (4:1 hexane:ethyl acetate);

^{1}H NMR (500 MHz, $CDCl_3$): δ 7.96 (s, 1H), 7.86 (s, 1H), 6.75 (dd, *J* = 9.8, 3.6 Hz, 1H), 6.65 (d, *J* = 8.5 Hz, 1H), 6.63 (d, *J* = 8.5 Hz, 1H), 6.21 (dd, *J* = 9.7, 3.4 Hz, 1H), 3.95 (s, 3H), 3.94 (s, 3H), 3.89 (d, *J* = 11.2 Hz, 1H), 3.76 (d, *J* = 11.2 Hz, 1H), 2.44 (d, *J* = 10.3 Hz, 1H), 2.35 (dd, *J* = 3.2, 3.2 Hz, 1H), 1.91 (d, *J* = 13.9 Hz, 1H), 1.87 – 1.75 (m, 3H), 1.41 (br s, 1H), 1.17 – 1.12 (m, 1H), 1.10 (s, 6H) ppm;

^{13}C NMR (125 MHz, $CDCl_3$): δ 149.8, 149.7, 146.9, 131.4, 129.9, 128.4, 126.0, 125.0, 119.2, 114.4, 103.3, 103.1, 66.1, 55.9, 55.8, 51.2, 38.5, 38.4, 36.4, 35.4, 26.2, 21.9, 18.9 ppm;

IR: 3400, 2931, 1596, 1460, 1456, 1436, 1325, 1269, 1249, 1102, 1091, 1025, 968, 904, 799, 723 cm^{-1};

HRMS (ESI): calculated for $[C_{23}H_{28}O_3+H]^+$: 352.2033, found: 352.2045;

$[\alpha]_D^{25}$: −212° (c = 1.10, $CHCl_3$).

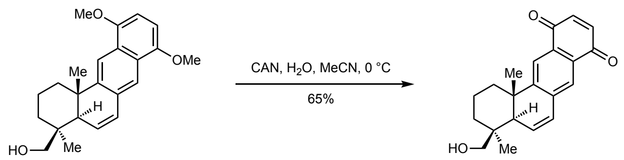

20 **(−)-21:** *ent*-neopetrosiquinone B

To a 10-mL one-neck round-bottom flask equipped with a stir bar was added alcohol **20** (15.0 mg, 0.0426 mmol, 1.00 equiv), (3.00 mL, 0.0142 M), and deionized water (1.00 mL, 0.0426 M). The flask was submerged in an ice/water bath, and ceric ammonium nitrate (70.1 mg, 0.128 mmol, 3.00 equiv) was added as one portion over approximately 5 seconds. The reaction was stirred for 30 minutes before being extracted with ethyl acetate (×3), washed with brine (×1), dried with sodium sulfate, filtered, and concentrated under reduced pressure. The residue was dissolved in the minimum volume of dichloromethane and purified via flash chromatography (ratio of silica to crude mass = 10:1, eluted with 4:1 hexane:ethyl acetate).

Yield: 8.9 mg, 0.028 mmol, 65%;

Appearance: yellow oil;

R_f: 0.26 (3:7 ethyl acetate:petroleum ether);

^{1}H NMR (500 MHz, $CDCl_3$): δ 7.88 (s, 1H), 7.71 (s, 1H), 6.93 (d, *J* = 10.4 Hz, 1H), 6.90 (d, *J* = 10.4 Hz, 1H), 6.68 (dd, *J* = 9.7, 3.2 Hz, 1H), 6.43 (dd, *J* = 9.7, 2.8 Hz, 1H), 3.84 (d, *J* = 11.1 Hz, 1H), 3.76 (d, *J* = 11.1 Hz, 1H), 2.35 – 2.34 (m, 1H), 2.31 (app t, *J* = 3.2, 1H), 1.90 (d, *J* = 11.9 Hz, 1H), 1.79 – 1.74 (m, 3H), 1.16 – 1.12 (m, 1H), 1.10 (s, 3H), 1.07 (s, 3H) ppm;

^{13}C NMR (125 MHz, $CDCl_3$): δ 185.3, 185.1, 153.8, 139.0, 138.6, 138.2, 134.4, 131.1, 130.5, 126.8, 124.3, 120.7, 66.2, 50.5, 38.6, 38.5, 36.0, 35.3, 26.2, 20.7, 18.6 ppm;

IR: 3451, 2929, 1667, 1664, 1590, 1144, 1051, 1030, 974, 920, 843, 730 cm^{-1};

HRMS (ESI): calculated for $[C_{21}H_{22}O_3]^+$: 352.2033, found: 352.2045;

$[\alpha]_D^{25}$: −163° (c = 0.660, $CHCl_3$).

Part 3: Orhalquinone, Xestoquinone, and Halenaquinone

Orhalquinone

optimized route

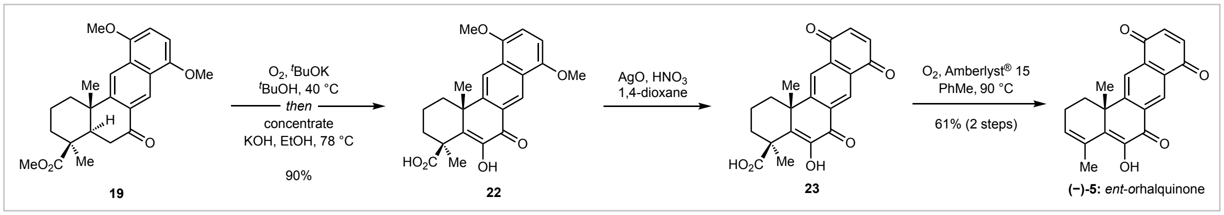

select route scouting

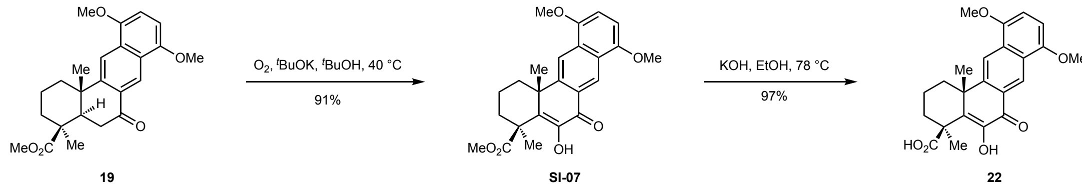

notes

- this two-step route abandoned once conditions to conduct a one-pot alternative were developed
- **(−)-5:** *ent*-orhalquinone was also be made via the deprotection of **23** using AgO and HNO_3 in THF, although yields were low (~20% on ~10 mg scale)

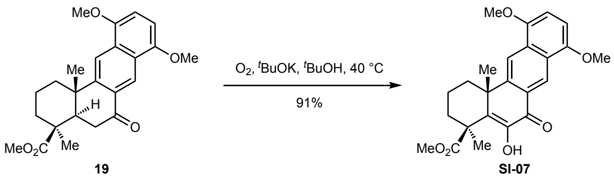

Conducted according to a modification of a related procedure reported by Harada.[13] To a 500-mL one-neck round-bottom flask open to air and equipped with a stir bar was added ketone **19** (3.50 g, 8.83 mmol, 1.00 equiv) and *tert*-butanol (175 mL, 0.0500 M). The flask was warmed on a heating mantle at 40 °C and oxygen (balloon) was bubbled through the solution for approximately 5 minutes. Potassium *tert*-butoxide (5.95 g, 53.0 mmol, 6.00 equiv) was added as one portion over approximately 10 seconds, and the reaction was stirred for 1.5 hours (oxygen balloon still attached). The heating mantle was removed, and the flask allowed to cool to room temperature. The reaction was quenched with 2 N HCl, extracted with ethyl acetate (×3), washed with brine (×1), dried over magnesium sulfate, filtered, and concentrated under reduced pressure. The residue was dissolved in the minimum volume of dichloromethane and purified via flash chromatography (ratio of silica to crude mass = 10:1, eluted with 7:3 hexane:ethyl acetate).

Yield: 3.298 g, 8.035 mmol, 91%;

Appearance: yellow oil;

R*f*: 0.24 (4:1 hexane:ethyl acetate);

^{1}H NMR (500 MHz, $CDCl_3$): 9.12 (s, 1H), 8.37 (s, 1H), 7.00 (s, 1H), 6.79 (d, J = 8.3 Hz, 1H), 6.68 (d, J = 8.3 Hz, 1H), 3.98 (two overlapping s, 6H), 3.72 (s, 3H), 2.71 (ddd, J = 13.6, 8.0, 2.5 Hz, 1H), 2.51 – 2.43 (m, 1H), 2.17 – 2.08 (m, 1H), 1.98 – 1.88 (m, 1H), 1.85 – 1.77 (m, 1H), 1.65 (s, 3H), 1.63 – 1.60 (m, 1H), 1.59 (s, 3H) ppm;

^{13}C NMR (125 MHz, $CDCl_3$): δ 180.9, 177.2, 150.8, 149.0, 148.2, 144.1, 136.6, 128.6, 125.7, 124.8, 123.2, 118.6, 106.1, 103.2, 55.9, 55.8, 52.4, 47.0, 40.3, 33.0, 32.6, 32.4, 24.5, 16.4 ppm;

IR: 3377, 3001, 2949, 2363, 2345, 1731, 1641, 1621, 1593, 1463, 1432, 1393, 1372, 1332, 1298, 1268, 1239, 1216, 1199, 1177, 1124, 1111, 1091, 1077, 1035, 968, 910, 797, 726 cm^{-1};

HRMS (ESI): calculated for $[C_{24}H_{26}O_6+H]^+$: 411.1802, found: 411.1811;

$[\alpha]_D^{25}$: −27.6° (c = 1.08, $CHCl_3$).

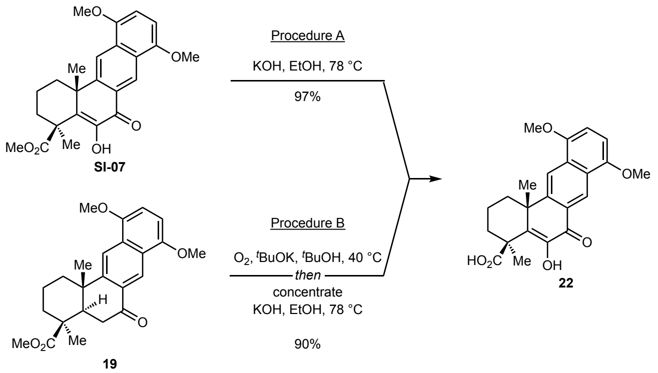

Note: Carboxylic acid **22** is prone to decarboxylation upon standing, which appears to be accelerated by flash column chromatography with untreated silica. Purification of **22** can be achieved provided phosphate buffered silica (pH = 7) was used as stationary phase (in minimal quantities), and compressed nitrogen (rather than air) is used to elute the column. Immediate submission to subsequent reactions is recommended.

<u>Procedure A</u>

To a 500-mL one-neck round-bottom flask open to air and equipped with a stir bar was added ester **SI-07** (1.50 g, 3.65 mmol, 1.00 equiv) and ethanol (75.0 mL, 0.049 M). A solution of potassium hydroxide (8.20 g, 146 mmol, 40.0 equiv) in deionized water (73 mL, 2.00 M) was added as one portion, and a high-efficiency air condenser was attached to the flask. The flask was warmed to 80 °C on a heating mantle, and the reaction was stirred for 8 hours before being cooled to room temperature, submerged in an ice/water bath, quenched with 2 N HCl, extracted with ethyl acetate (×3), washed with brine (×1), dried over magnesium sulfate, filtered, and concentrated under reduced pressure. The residue was dissolved in the minimum volume of dichloromethane and purified via flash column chromatography using phosphate buffered silica (pH = 7, ratio of buffered silica to crude mass = 5:1, eluted with 3:2 hexane:ethyl acetate).

Yield: 1.041 g, 3.544 mmol, 97%.

<u>Procedure B</u>

To a 1000-mL one-neck round-bottom flask open to air and equipped with a stir bar was added ketone **19** (3.630 g, 9.156 mmol, 1.00 equiv) and *tert*-butanol (185.0 mL, 0.049 M). Oxygen (balloon) was bubbled through the solution while the flask was warmed to 40 °C on a heating mantle (approximately 5 minutes). Potassium *tert*-butoxide (6.164 g, 54.94 mmol, 6.00 equiv) was added as one portion over approximately 10 seconds, the needle was lifted into the headspace, and the reaction was stirred for 1.5 hours. The stir bar was removed, and the solvent was removed under reduced pressure. The stir bar was re-added, and the residue was dissolved in ethanol (183 mL, 0.050 M). A solution of potassium hydroxide (20.55 g, 366.2 mmol, 40.0 equiv) in deionized water (122 mL, 3.0 M) was added as one portion, and the flask was equipped with a high-efficiency air condenser. The flask was warmed to 80 °C on a heating mantle, and the reaction was stirred for 8 hours before being cooled to room temperature, submerged in an ice/water bath, quenched with 2 N HCl, extracted with ethyl acetate (×3), washed with brine (×1), dried over magnesium sulfate, filtered, and concentrated under reduced pressure. The residue was dissolved in the minimum volume of dichloromethane and purified via flash column chromatography using phosphate buffered silica (pH = 7, ratio of buffered silica to crude mass = 10:1, eluted with 3:2 hexane:ethyl acetate).

Yield: 3.267 g, 8.240 mmol, 90%;

Appearance: orange oil;

R*f*: 0.22 (3:2 ethyl acetate:hexane);

^{1}H NMR (500 MHz, $CDCl_3$): δ 9.12 (s, 1H), 8.37 (s, 1H), 6.79 (d, *J* = 8.3 Hz, 1H), 6.67 (d, *J* = 8.3 Hz, 1H), 3.97 (s, 4H), 3.97 (s, 3H), 2.72 (ddd, *J* = 13.7, 8.3, 2.7 Hz, 1H), 2.54 (ddd, *J* = 13.7, 9.2, 7.2 Hz, 1H), 2.20 – 2.10 (m, 1H), 1.98 – 1.90 (m, 1H), 1.87 – 1.79 (m, 1H), 1.74 – 1.69 (m, 1H), 1.67 (s, 3H), 1.63 (s, 3H) ppm.

^{13}C NMR (125 MHz, $CDCl_3$): δ 182.1, 180.7, 150.8, 148.9, 148.2, 144.2, 136.2, 128.6, 125.7, 124.8, 123.3, 118.5, 106.1, 103.2, 55.9, 55.8, 46.8, 40.2, 33.5, 32.1, 32.1, 24.0, 16.2 ppm;

IR (neat) 3180, 2949, 2360, 2337, 1701, 1623, 1469, 1395, 1298, 1268, 1178, 1111, 1077, 970, 912, 809, 726 cm^{-1};

HRMS (ESI): calculated for $[C_{23}H_{24}O_6+H]^+$: 397.1646, found 397.1665;

$[\alpha]_D^{25}$: –40.6 (c = 0.84, $CHCl_3$).

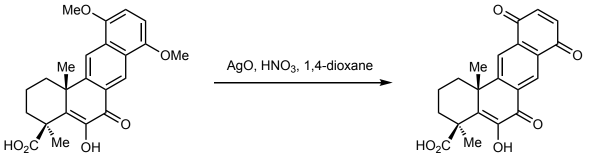

Note: Carboxylic acid **23** is prone to decarboxylation upon standing. It was not uncommon to observe small quantities of (*ent*)-orhalquinone in crude reaction mixtures. Immediate submission to subsequent reactions is recommended. Due to the instability of the crude residue, which was used without further purification, we only report here the R*f* and a crude ^{1}H NMR.

To a 250-mL one-neck round-bottom flask open to air and equipped with a stir bar was added carboxylic acid **22** (27.1 mg, 0.07 mmol, 1.00 equiv) and 1,4-dioxane (1.90 mL, 0.037 M). Silver(II) oxide (39.6 mg, 0.17 mmol, 2.50 equiv) and an aqueous solution of 2 M nitric acid (0.34 mL, 0.7 mmol, 10.0 equiv) were added sequentially each as one portion, and the reaction was stirred for 30 minutes before being diluted with deionized water (approximately 10 mL), extracted with ethyl acetate (×3), washed with brine (×1), dried over sodium sulfate, filtered, and concentrated under reduced pressure. The residue was submitted to subsequent reactions without further purification.

Appearance: orange oil;

R*f*: 0.37 (1:4 hexane:ethyl acetate);

^{1}H NMR (500 MHz, $CDCl_3$): δ 8.90 (s, 1H), 8.33 (s, 1H), 7.07 (s, 1H), 7.07 (s, 1H), 6.99 (br s, 1H), 2.65 (ddd, J = 13.7, 8.3, 2.7 Hz, 1H), 2.53 (ddd, J = 13.9, 7.9, 7.9 Hz, 1H), 2.20 – 2.13 (m, 2H), 1.99 – 1.90 (m, 2H), 1.68 (s, 3H), 1.63 (s, 3H) ppm;

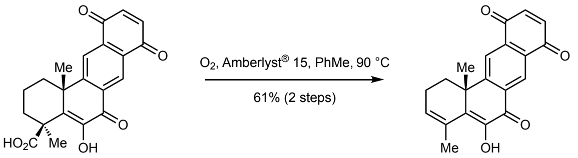

23 **(−)-5:** *ent*-orhalquinone

To a 50-mL one-neck round-bottomed flask open to air and equipped with a stir bar was added crude carboxylic acid **23** (25.0 mg, 0.068 mmol, 1.00 equiv) and toluene (2.5 mL, 0.027 M). An oxygen balloon was bubbled through the solution while the flask was warmed to 90 °C on a heating mantle (approximately 10 minutes). Amberlyst® 15 (25.0 mg, approximately 1× crude mass) was added as one portion, the oxygen needle was lifted into the headspace, the vent needle was removed, and the reaction was stirred for 1 hour. The flask was allowed to cool to room temperature over approximately 15 minutes before the solution was filtered and concentrated under reduced pressure. The residue was dissolved in the minimum volume of dichloromethane and purified via flash chromatography (ratio of buffered silica to crude mass = 10:1, eluted with 4:1 hexane:ethyl acetate).

Yield: 13.1 mg, 0.041 mmol, 61%;

Appearance: orange powder;

R*f*: 0.25 (15:85 ethyl acetate:petroleum ether);

^{1}H NMR (500 MHz, $CDCl_3$): 8.92 (s, 1H), 8.34 (s, 1H), 7.09 (s, 1H), 7.07 (s, 1H), 7.06 (s, 1H), 5.90 (s, 1H), 2.61 – 2.52 (m, 1H), 2.49 (dd, J = 13.0, 6.3 Hz, 1H), 2.45 – 2.39 (m, 1H), 2.29 (br s, 3H), 1.69 (ddd, J = 12.2, 12.1, 6.4 Hz, 1H), 1.48 (s, 3H) ppm;

^{13}C NMR (125 MHz, $CDCl_3$): δ 184.7, 183.8, 179.5, 156.4, 142.6, 139.6, 139.1, 133.9, 133.2, 132.2, 132.0, 131.9, 130.4, 126.1, 125.3, 39.7, 33.7, 27.6, 23.7, 23.5 ppm;

IR: 3363, 2932, 2871, 1676, 1635, 1619, 1456, 1383, 1363, 1286, 1228, 1145, 1059 cm^{-1};

HRMS (ESI): calculated for $[C_{20}H_{16}O_4+H]^+$: 321.1121, found: 321.1117;

$[\alpha]_D^{25}$: −159° (c = 0.96, $CHCl_3$); −150° (c = 0.18, CH_3OH).

Xestoquinone

optimized route

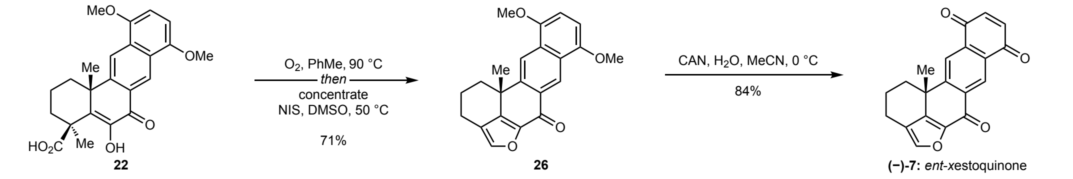

select route scouting

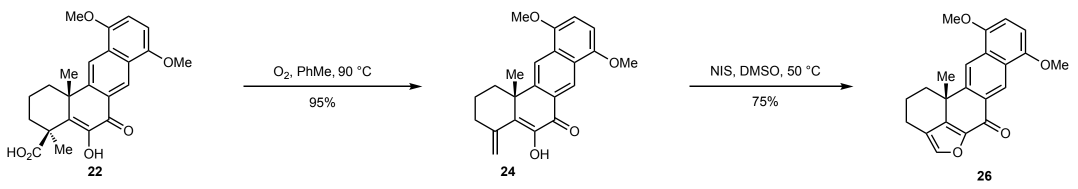

notes

- two-step route abandoned once conditions to conduct a one-pot alternative were developed (avoids isolation of sensitive **24**)

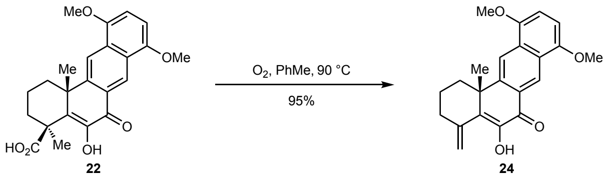

To a 250-mL one-neck round-bottom flask open to air and equipped with a stir bar was added carboxylic acid **22** (0.250 g, 0.631 mmol, 1.00 equiv) and toluene (63 mL, 0.010 M). The flask was warmed on a heating mantle at 90 °C and oxygen (balloon) was bubbled through the solution for approximately 10 minutes. The needle was lifted into the headspace, and the reaction was stirred for 1.5 hours. The stir bar was removed, and the solvent was removed under reduced pressure. **Note**: The crude residue was typically pure enough to use directly in subsequent reactions without further purification. However, if necessary, it can be purified by dissolving in the minimum volume of dichloromethane and then subjected to flash chromatography (ratio of silica to crude mass = 10:1, eluted with 17:3 hexane:ethyl acetate). This compound is prone to isomerization of the exocyclic olefin into the ring (**25**) upon exposure to acid.

Yield: 0.210 g, 0.599 mmol, 95%;

Appearance: orange oil;

R*f*: 0.24 (4:1 hexane:ethyl acetate);

^{1}H NMR (500 MHz, $CDCl_3$): δ 9.18 (s, 1H), 8.38 (s, 1H), 6.96 (br s, 1H), 6.78 (d, *J* = 8.3 Hz, 1H), 6.67 (d, *J* = 8.3 Hz, 1H), 5.49 (dd, *J* = 2.2, 2.2 Hz, 1H), 5.36 (dd, *J* = 2.1, 2.1 Hz, 1H), 3.98 (s, 3H), 3.97 (s, 3H), 2.59 (dddd, *J* = 12.8,

12.8, 7.1, 7.1 Hz, 2H), 2.31 – 2.25 (m, 1H), 2.08 – 1.98 (m, 1H), 1.90 – 1.87 (m, 1H), 1.70 (ddd, J = 13.4, 13.4, 4.2 Hz, 1H), 1.51 (s, 3H) ppm;

^{13}C NMR (125 MHz, $CDCl_3$): δ 181.0, 150.8, 148.9, 147.1, 141.2, 140.9, 135.5, 128.6, 126.0, 124.9, 123.1, 119.4, 116.5, 105.9, 103.1, 55.8, 40.7, 40.0, 35.9, 29.1, 22.6 ppm;

IR: 3584, 2977, 2912, 2360, 1594, 1455, 1248, 1091, 1048, 1018, 897, 811, 724 cm^{-1};

HRMS (ESI): calculated for $[C_{22}H_{22}O_4+H]^+$: 351.1591, found: 351.1605;

$[\alpha]_D^{25}$: –236.7° (c = 5.92, $CHCl_3$).

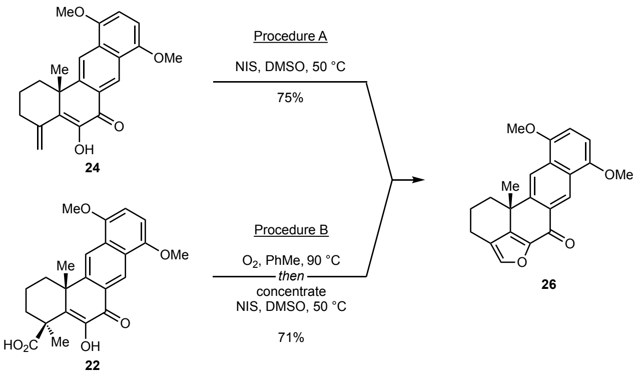

Procedure A

Conducted according to a modification of a related procedure reported by del Moral and Barrero.[26] To a 100-mL one-neck round-bottom flask open to air and equipped with a stir bar was added alkene **24** (0.105 g, 0.300 mmol, 1.00 equiv) and dimethyl sulfoxide (6.0 mL, 0.050 M). *N*-iodosuccinimide (1.17 g, 5.25 mmol, 1.75 equiv) was added as one portion, the flask was warmed to 50 °C on a heating mantle, and the reaction was stirred for four hours before being quenched with saturated aqueous sodium thiosulfate (approximately 3 mL), extracted with ethyl acetate, washed with 5% aqueous lithium chloride (×3), saturated aqueous sodium bicarbonate (×1), and brine (×1), dried over magnesium sulfate, filtered, and concentrated under reduced pressure. The residue was dissolved in the minimum volume of dichloromethane and purified via flash chromatography (ratio of silica to crude mass = 10:1, eluted with 7:3 hexane:ethyl acetate).

Yield: 0.0784 g, 0.225 mmol, 75%;

Procedure B

To a 250-mL one-neck round-bottom flask open to air and equipped with a stir bar was added carboxylic acid **22** (0.125 g, 0.316 mmol, 1.00 equiv) and toluene (32.0 mL, 0.0100 M). Oxygen (balloon) was bubbled through the solution while the flask was warmed to 90 °C on a heating mantle (approximately 10 minutes). The needle was lifted into the headspace, and the reaction was stirred for 1.5 hours. The stir bar was removed, and the reaction was concentrated under reduced pressure. The stir bar was returned to the flask, the residue was dissolved in dimethyl sulfoxide (6.3 mL, 0.050 M), and *N*-iodosuccinimide (1.23 g, 5.50 mmol, 1.75 equiv) was added as one portion. The

flask was warmed to 50 °C on a heating mantle, and the reaction was stirred for four hours before being quenched with saturated aqueous sodium thiosulfate (approximately 3 mL), extracted with ethyl acetate (×3), washed with 5% aqueous lithium chloride (×3), saturated aqueous sodium bicarbonate (×1), and brine (×1), dried over magnesium sulfate, filtered, and concentrated under reduced pressure. The residue was dissolved in the minimum volume of dichloromethane and purified via flash chromatography (ratio of silica to crude mass = 10:1, eluted with 7:3 hexane:ethyl acetate).

Yield: 0.0780 g, 0.224 mmol, 71%;

Appearance: orange oil;

R*f*: 0.20 (4:1 hexane:ethyl acetate);

^{1}H NMR (500 MHz, $CDCl_3$): δ 9.28 (s, 1H), 8.28 (s, 1H), 7.47 (s, 1H), 6.81 (d, *J* = 9.3 Hz, 1H), 6.70 (d, *J* = 9.4 Hz, 1H), 3.98 (s, 3H), 3.98 (s, 3H), 2.87 (dd, *J* = 16.9, 7.7 Hz, 1H), 2.66 – 2.59 (m, 2H), 2.32 – 2.22 (m, 1H), 2.17 – 2.13 (m, 1H), 1.83 (ddd, *J* = 13.3, 13.3, 4.3 Hz, 1H), 1.54 (s, 3H) ppm;

^{13}C NMR (125 MHz, $CDCl_3$): δ 173.0, 151.1, 149.0, 146.9, 146.9, 145.0, 143.9, 131.6, 127.5, 124.9, 124.3, 121.4, 117.7, 106.2, 103.5, 77.2, 55.9, 55.9, 36.5, 33.9, 32.2, 18.9, 17.4 ppm;

IR: 2939, 2837, 1669, 1629, 1614, 1465, 1443, 1435, 1425, 1355, 1341, 1268, 1245, 1229, 1145, 1112, 1091, 1045, 804, 726 cm^{-1};

HRMS (ESI): calculated for $[C_{22}H_{20}O_4+H]^+$: 349.1435, found: 349.1437;

$[\alpha]_D^{25}$: –86.7° (c = 1.66, $CHCl_3$).

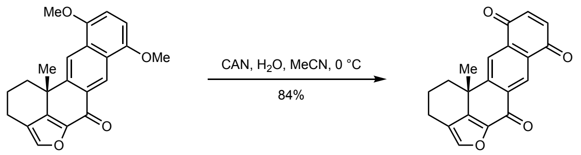

To a 100-mL one-neck round-bottom flask equipped with a stir bar was added **26** (80.0 mg, 0.236 mmol, 1.00 equiv), acetonitrile (15.0 mL, 0.016 M), and deionized water (5.00 mL, 0.047 M). The flask was submerged in an ice/water bath, and ceric ammonium nitrate (0.388 mg, 0.708 mmol, 3.00 equiv.) was added as one portion over approximately 5 seconds. The flask was stoppered, and the reaction was stirred for 30 minutes before being extracted with ethyl acetate (×3), washed with brine (×1), dried with magnesium sulfate, filtered, and concentrated under reduced pressure. The residue was dissolved in the minimum volume of hexane and purified via flash chromatography (ratio of silica to crude mass = 10:1, eluted with 9:1 hexane:ethyl acetate). **Notes**: The yield of this reaction was sensitive toward the source of ceric ammonium nitrate. Under otherwise identical conditions, newer bottles of ceric ammonium nitrate led to significant quantities of an unidentified compound that we tentatively assign as a product of over oxidation. In addition, in our hands xestoquinone appears to be somewhat sensitive to silica, as evidenced by two-dimensional thin-layer chromatographic analysis.

Yield: 63.1 mg, 0.199 mmol, 84%;

Appearance: yellow-orange solid;

R*f*: 0.28 (7:3 hexane:ethyl acetate);

^{1}H NMR (600 MHz, $CDCl_3$): δ 9.06 (s,1H), 8.25 (s, 1H), 7.54 (s, 1H), 7.06 (d, *J* = 10.3 Hz, 1H), 7.04 (d, *J* = 10.3 Hz, 1H), 2.89 (dd, *J* = 16.7, 7.9, Hz, 1H), 2.65 (ddd, *J* = 16.9, 10.1, 10.1 Hz, 1H), 2.58 (ddd, *J* = 12.9, 3.4, 3.4 Hz, 1H), 2.33 – 2.24 (m, 1H), 2.20 – 2.16 (m, 1H), 1.76 (ddd, *J* = 13.0, 13.0, 4.6 Hz, 1H), 1.54 (s, 3H) ppm;

^{13}C NMR (150 MHz, $CDCl_3$): δ 184.9, 184.0, 170.4, 156.4, 147.4, 145.1, 144.3, 139.6, 138.8, 138.2, 133.4, 130.6, 127.3, 123.4, 121.6, 37.5, 32.7, 31.4, 18.6, 17.1 ppm;

IR (solid): 2925, 2856, 1694, 1596, 1465, 1404, 1261, 1200, 1084, 1038, 975, 887, 747 cm^{-1};

HRMS (ESI): calculated for $[C_{20}H_{15}O_4+H]^+$: 319.0965, found: 319.0969;

$[\alpha]_D^{25}$: –21.1° (c = 1.00, CH_2Cl_2).

Halenaquinone

optimized route

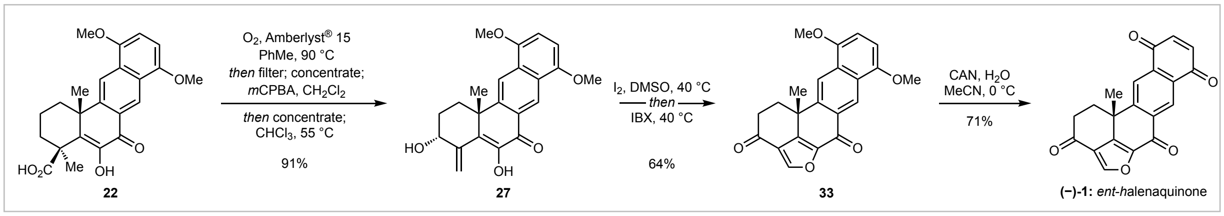

select route scouting

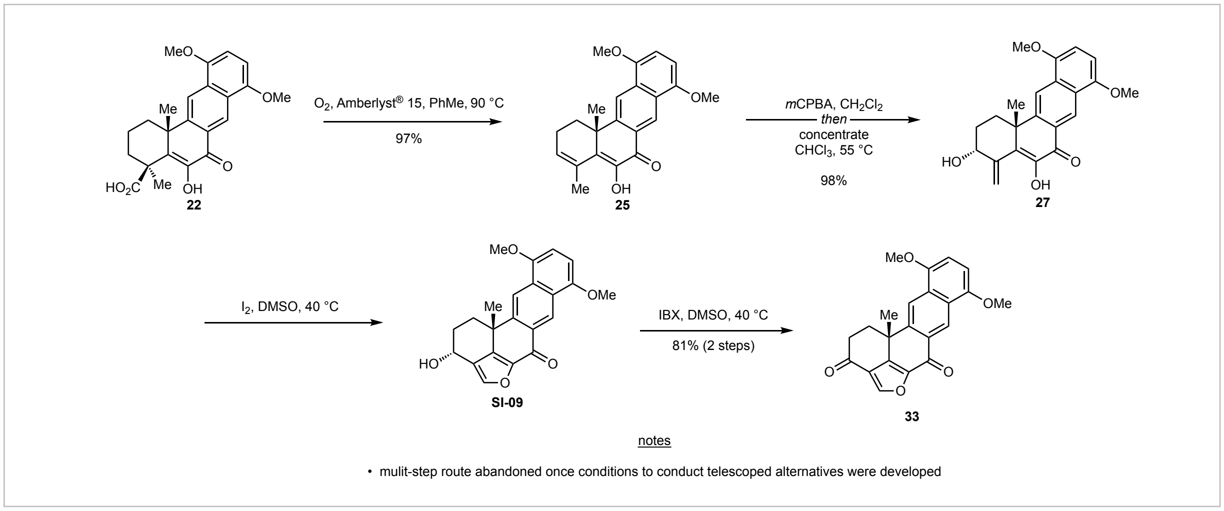

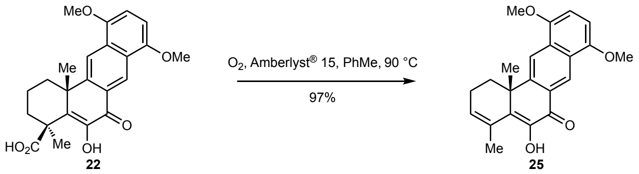

To a 250-mL one-neck round-bottomed flask open to air and equipped with a stir bar was added carboxylic acid **22** (1.401 g, 3.534 mmol, 1.00 equiv) and toluene (40.0 mL, 0.088 M). An oxygen balloon was bubbled through the solution while the flask was warmed to 90 °C on a heating mantle (approximately 10 minutes). Amberlyst® 15 (1.40 g, approximately 1× mass) was added as one portion, the oxygen needle was lifted into the headspace, the vent needle was removed, and the reaction was stirred for 1 hour. The flask was allowed to cool to room temperature over approximately 15 minutes before the solution was filtered and concentrated under reduced pressure. The residue was dissolved in the minimum volume of dichloromethane and purified via flash chromatography (ratio of buffered silica to crude mass = 10:1, eluted with 4:1 hexane:ethyl acetate).

Yield: 1.201 g, 3.428 mmol, 97%;

Appearance: reddish-brown solid

R_f: 0.30 (9:1 hexane:ethyl acetate);

^{1}H NMR (500 MHz, $CDCl_3$): 9.15 (s, 1H), 8.41 (s, 1H), 7.28 (s, 1H), 6.79 (d, J = 8.3 Hz, 1H), 6.68 (d, J = 8.3 Hz, 1H), 5.85 (br s, 1H), 3.99 (s, 3H), 3.99 (s, 3H), 2.60 – 2.52 (m, 2H), 2.42 – 2.35 (m, 1H), 2.31 (s, 3H), 1.82 - 1.72 (m, 1H), 1.49 (s, 3H) ppm;

^{13}C NMR (150 MHz, $CDCl_3$): δ 181.3, 150.8, 148.9, 146.8, 142.4, 132.1, 132.0, 131.2, 128.6, 125.9, 125.0, 122.7, 119.4, 105.8, 103.0, 55.8, 55.8, 38.7, 34.8, 29.0, 24.0, 23.7 ppm;

IR: 3354, 2961, 2931, 1614, 1463, 1390, 1332, 1301, 1268, 1241, 1218, 1197, 1114, 1085, 980, 906, 794, 726 cm^{-1};

HRMS (ESI): calculated for $[C_{22}H_{22}O_4+H]^+$: 351.1591, found 351.1593;

$[\alpha]_D^{25}$: –197.5° (c = 1.16, $CHCl_3$).

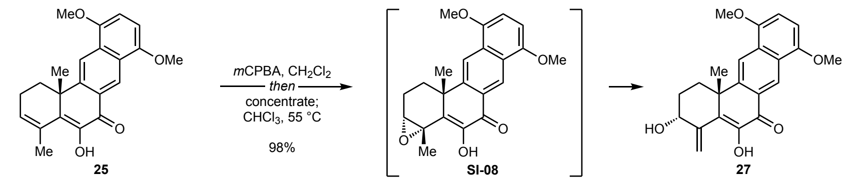

Note: Epoxide **SI-08** is unstable to a variety of acidic conditions (e.g., $MgSO_4$, SiO_2, BF_3OEt_2, $CHCl_3$, 2 N HCl, Amberlyst® 15, *para*-toluenesulfonic acid) as well as phosphate-buffered silica (pH = 7), hence it was immediately submitted to thermal ring-opening conditions.

To a 250-mL, one-neck round-bottom flask open to air and equipped with a stir bar was added **25** (0.250 g, 0.713 mmol, 1.00 equiv) and dichloromethane (35 mL, 0.020 M). *meta*-Chloroperoxybenzoic acid (0.264 g, 70% wt, 1.070 mmol, 1.50 equiv) was added as one portion, and the reaction was stirred at room temperature for 30 minutes before being quenched with a saturated solution of aqueous sodium thiosulfate (approximately 5 mL) and extracted with dichloromethane. The organics were washed with a saturated solution of aqueous sodium bicarbonate (×2), 10% aqueous sodium sulfate (×1), and brine (×1), dried with sodium sulfate, filtered, and concentrated under reduced pressure at 30 °C. The crude residue (approximately a 4:1 mixture of epoxide **SI-09**:allylic alcohol **27** by ^{1}H NMR) was transferred to a 100-mL, one-neck round-bottom flask open to air and equipped with a stir bar and high-efficiency air condenser, dissolved in chloroform (35 mL, 0.020 M) and warmed to 55 °C on a heating mantle. The reaction was stirred for 15 hours before being concentrated under reduced pressure, dissolved in the minimum volume of dichloromethane, and purified via flash chromatography (ratio of buffered silica to crude mass = 10:1, eluted with 7:3 hexane:ethyl acetate).

Yield: 0.256 g, 0.699 mmol, 98%;

Appearance: Reddish-brown solid;

R*f*: 0.37 (1:1 hexane:ethyl acetate);

^{1}H NMR (500 MHz, $CDCl_3$): δ 9.18 (s, 1H), 8.39 (s, 1H), 7.01 (s, 1H), 6.80 (d, J = 8.3 Hz, 1H), 6.69 (d, J = 8.3 Hz, 1H), 5.67 (br s, 1H), 5.55 (br s, 1H), 4.49 (s, 1H), 3.99 (s, 3H), 3.98 (s, 3H), 2.41 – 2.37 (m, 1H), 2.21 – 2.17 (m, 2H), 2.10 – 2.04 (m, 1H), 1.87 (br s, 1H), 1.50 (s, 3H) ppm;

^{13}C NMR (125 MHz, $CDCl_3$): δ 180.6, 150.8, 148.9, 146.8, 143.3, 142.8, 132.4, 128.7, 125.9, 125.0, 123.3, 118.7, 119.5, 106.0, 103.2, 73.0, 55.9, 55.9, 40.4, 34.0, 29.0, 28.9 ppm;

IR (neat): 2935, 1640, 1620, 1593, 1432, 1435, 1382, 1333, 1294, 1268, 1239, 1216, 1191, 1169, 1155, 1112, 1091, 1081, 1060, 1044, 1023, 994, 967, 910, 806 cm^{-1};

HRMS (ESI): calculated for $[C_{22}H_{22}O_5+H]^+$: 367.1540, found: 367.1541;

$[\alpha]_D^{25}$: –334.8° (c = 1.14, $CHCl_3$).

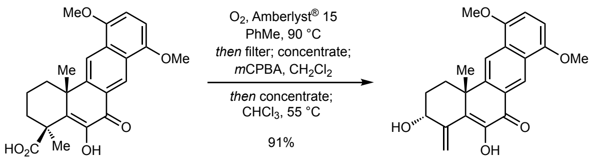

22 **27**

To a 250-mL one-neck round-bottomed flask open to air and equipped with a stir bar was added carboxylic acid **22** (0.750 g, 1.89 mmol, 1.00 equiv) and toluene (21 mL, 0.088 M). An oxygen balloon was bubbled through the solution while the flask was warmed to 90 °C on a heating mantle (approximately 10 minutes). Amberlyst® 15 (0.750 g, approximately 1× mass) was added as one portion, the oxygen needle was lifted into the headspace, the vent needle was removed, and the reaction was stirred for 1 hour. The flask was allowed to cool to room temperature over approximately 15 minutes before the solution was filtered and concentrated under reduced pressure, and the residue was dissolved in dichloromethane (38 mL, 0.020 M). A stir bar was added to the flask, and *meta*-chloroperoxybenzoic acid (0.699 g, 2.84 mmol, 1.50 equiv, contains approximately 30% water) was added as one portion, and the reaction was stirred at room temperature for 45 minutes. The stir bar was removed, the solution was concentrated under reduced pressure, and the residue was dissolved in chloroform (38 mL, 0.020 M). A stir bar was added, a high-efficiency air condenser was attached to the flask, and the solution was warmed to 55 °C on a heating mantle. The reaction was stirred for 15 hours before being concentrated under reduced pressure, dissolved in the minimum volume of dichloromethane, and purified via flash chromatography (ratio of buffered silica to crude mass = 10:1, eluted with 7:3 hexane:ethyl acetate).

Yield: 0.630 g, 1.72 mmol, 91%;

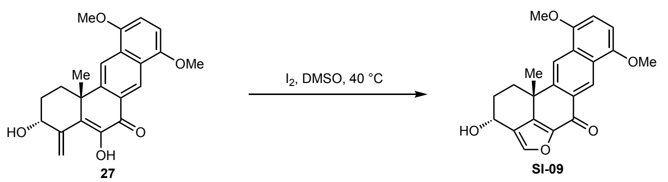

Conducted according to a modification of a related procedure reported by del Moral and Barrero.[26] To a 50-mL, one-neck round-bottom flask open to air and equipped with a stir bar was added allylic alcohol **27** (15.1 mg, 0.041 mmol, 1.00 equiv) and dimethyl sulfoxide (1.0 mL, 0.04 M). Iodine (15.6 mg, 0.062 mmol, 1.50 equiv) was added as one portion, and the reaction was stirred for 10 minutes, warmed to 40 °C on a heating mantle, and stirred for an additional 16 hours. The reaction was cooled to room temperature and quenched with a saturated solution of aqueous sodium thiosulfate (approximately 1 mL), extracted with ethyl acetate (×3), washed with 5% aqueous lithium chloride (×3), saturated aqueous sodium bicarbonate (×1), and brine (×1), dried with sodium sulfate, filtered, and concentrated under reduced pressure. Due to the instability of the crude residue, which was used without further purification, we only report here the R_f and a crude ^{1}H NMR.

Appearance: reddish-brown solid;

$\mathbf{R_f}$: 0.21 (3:2 hexane:ethyl acetate);

^{1}H NMR (500 MHz, $CDCl_3$): δ 9.18 (s, 1H), 8.39 (s, 1H), 7.01 (s, 1H), 6.80 (d, J = 8.3 Hz, 1H), 6.69 (d, J = 8.3 Hz, 1H), 5.67 (br s, 1H), 5.55 (br s, 1H), 4.49 (s, 1H), 3.99 (s, 3H), 3.98 (s, 3H), 2.41 – 2.37 (m, 1H), 2.21 – 2.17 (m, 2H), 2.10 – 2.04 (m, 1H), 1.50 (s, 3H) ppm;

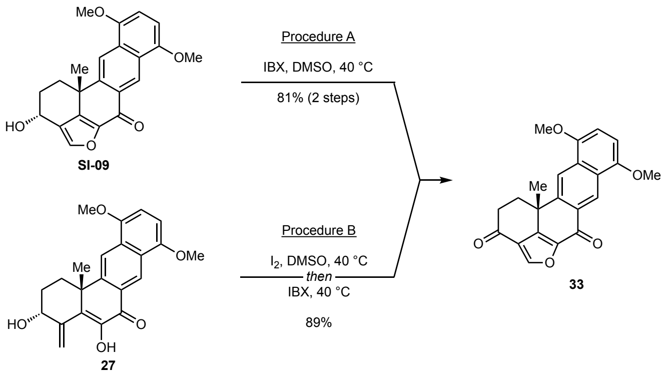

Procedure A

To a 25-mL one-neck round-bottomed flask open to air and equipped with a stir bar was added crude **SI-09** (15.0 mg, 0.041 mmol, 1.00 equiv) and dimethyl sulfoxide (1.5 mL, 0.027 M). 2-iodoxybenzoic acid (20.2 mg, 0.072 mmol, 1.75 equiv) was added as one portion over approximately 5 seconds, the flask was warmed to 40 °C on a heating mantle, and the reaction was stirred for four hours before being allowed to cool to room temperature over 10 minutes. The reaction was quenched with a saturated solution of aqueous sodium thiosulfate (approximately 2 mL), extracted with ethyl acetate (×3), washed with 5% aqueous lithium chloride (×3), a saturated solution of sodium bicarbonate (×2), and brine (×1), dried with sodium sulfate, filtered, and concentrated under reduced pressure. The residue was

dissolved in the minimum volume of dichloromethane and purified via flash chromatography (ratio of buffered silica to crude mass = 10:1, eluted with 3:2 hexane:ethyl acetate).

Yield: 12.0 mg, 0.033 mmol, 81% (2 steps);

Procedure B

Conducted according to a modification of a related procedure reported by del Moral and Barrero.[26] To a 25-mL one-neck round-bottomed flask open to air and equipped with a stir bar was added allylic alcohol **27** (5.5 mg, 0.015 mmol, 1.00 equiv) and dimethyl sulfoxide (1.0 mL, 0.015 M). Iodine (5.8 mg, 0.023 mmol, 1.50 equiv) was added as one portion, the flask was warmed to 40 °C on a heating mantle, and the reaction was stirred for 4 hours before being allowed to cool to room temperature over 10 minutes. 2-iodoxybenzoic acid (8.4 mg, 0.030 mmol, 2.00 equiv) was added as one portion, the flask was warmed to 40 °C on a heating mantle, and the reaction was stirred for 4 hours before being allowed to cool to room temperature over 10 minutes. The reaction was quenched with a saturated solution of aqueous sodium thiosulfate (approximately 2 mL), extracted with ethyl acetate (×3), washed with 5% aqueous lithium chloride (×3), a saturated solution of sodium bicarbonate (×2), and brine (×1), dried with sodium sulfate, filtered, and concentrated under reduced pressure. The residue was dissolved in the minimum volume of dichloromethane and purified via flash chromatography (ratio of buffered silica to crude mass = 10:1, eluted with 3:2 hexane:ethyl acetate).

Yield: 4.7 mg, 0.013 mmol, 89%;

Appearance: orange oil;

R*f*: 0.30 (3:2 hexane:ethyl acetate);

^{1}H NMR (500 MHz, $CDCl_3$): δ 9.30 (s, 1H), 8.31 (s, 1H), 8.22 (s, 1H), 6.85 (d, *J* = 8.3 Hz, 1H), 6.74 (d, *J* = 8.3 Hz, 1H), 3.99 (s, 3H), 3.99 (s, 3H), 3.02 (ddd, *J* = 18.7, 13.6, 5.4 Hz, 1H), 2.92 (ddd, *J* = 13.3, 5.4, 2.0 Hz, 1H), 2.82 (ddd, *J* = 18.6, 4.9, 2.0 Hz, 1H), 2.34 (ddd, *J* = 13.4, 13.4, 4.8 Hz, 1H), 1.68 (s, 3H) ppm;

^{13}C NMR (125 MHz, $CDCl_3$): δ 192.3, 172.8, 150.9, 148.8, 148.4, 147.1, 145.8, 144.6, 130.6, 127.7, 124.9, 124.8, 122.7, 118.6, 106.7, 103.9, 77.1, 55.9, 55.9, 36.9, 35.9, 34.3, 31.9 ppm;

IR: 2935, 2362, 1697, 1671, 1629, 1617, 1594, 1526, 1469, 1463, 1436, 1395, 1349, 1339, 1266, 1254, 1241, 1216, 1164, 1149, 1117, 1098, 1044, 967, 924, 807, 733, 726 cm^{-1};

HRMS (ESI): calculated for $[C_{22}H_{18}O_5+H]^+$: 363.1227, found: 363.1228;

$[\alpha]_D^{25}$: −146.3° (c = 0.324, $CHCl_3$).

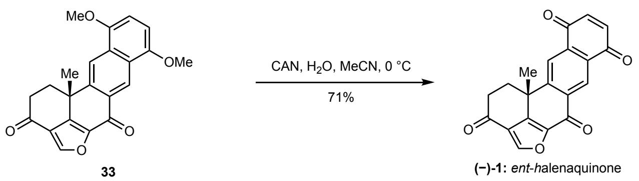

To a 20-mL scintillation vial open to air and equipped with a stir bar was added **33** (6.5 mg, 0.018 mmol, 1.00 equiv), acetonitrile (1.5 mL, 0.012 M), and deionized water (0.5 mL, 0.036 M). The vial was submerged in an ice/water bath, and ceric ammonium nitrate (29.5 mg, 0.054 mmol, 3.00 equiv) was added as one portion over approximately 5 seconds. The reaction was stirred for 10 minutes before being extracted with diethyl ether (×3), washed with brine (×1), dried with sodium sulfate, filtered, and concentrated under reduced pressure. The residue was dissolved in the minimum volume of dichloromethane and purified via flash chromatography (ratio of buffered silica to crude mass = 10:1, eluted with 2:3 hexane:diethyl ether). **Notes**: In our hands, halenaquinone appears to be somewhat sensitive to silica, as evidenced by two-dimensional thin-layer chromatographic analysis.

Yield: 5.2 mg, 0.016 mmol, 87%;

Appearance: yellow solid;

R*f*: 0.24 (3:2 hexane:ethyl acetate);

^{1}H NMR (400 MHz, $CDCl_3$): δ 8.90 (s, 1H), 8.72 (s, 1H), 8.34 (s, 1H), 7.19 (s, 2H), 3.10 (ddd, *J* = 18.1, 13.3, 5.4 Hz, 1H), 2.94 (dd, *J* = 12.7, 4.6 Hz, 1H), 2.67 (dd, *J* = 17.9, 3.5 Hz, 1H), 2.21 (ddd, *J* = 13.1, 12.9, 4.4 Hz, 1H), 1.66 (s, 3H) ppm;

^{13}C NMR (225 MHz, $CDCl_3$): δ 191.6, 184.3, 183.9, 170.0, 154.6, 151.0, 148.6, 144.0, 139.2, 139.2, 136.4, 133.7, 130.4, 125.1, 123.8, 122.5, 36.7, 36.3, 32.3, 29.8 ppm;

IR: 2921, 2857, 2517, 2343, 2154, 1984, 1689, 1614, 1528, 1457, 1373, 1310, 1259, 1100, 1031, 804, 755, 704 cm^{-1};

HRMS (ESI): calculated for $[C_{20}H_{12}O_5+H]^+$: 333.0758, found: 333.0741;

$[\alpha]_D^{25}$: –18.9° (c = 0.12, CH_2Cl_2).

Part 4: Xestoquinolides B and C

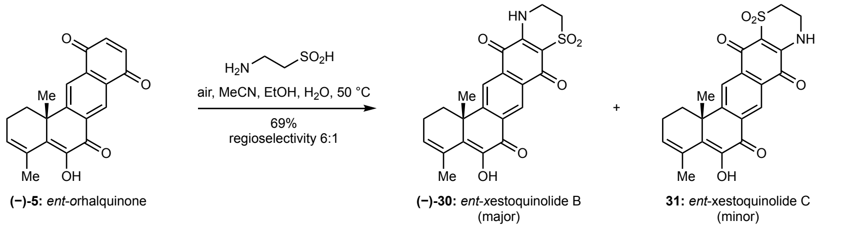

Conducted according to a modification of a related procedure reported by Gao.[12] To a 20-mL glass scintillation vial open to air and equipped with a stir bar was added (−)-orhalquinone **5** (42.0 mg, 0.131 mmol, 1.00 equiv), ethanol (2.9 mL, 0.045 M), acetonitrile (2.9 mL, 0.045 M), and deionized water (1.4 mL, 0.094 M). Hypotaurine (21.4 mg, 0.197 mmol, 1.50 equiv) was added as one portion, the vial was sealed and warmed to 50 °C on a heating mantle, and the reaction was stirred for 4 hours before being allowed to cool to room temperature. The reaction was concentrated under reduced pressure, and the crude residue was dissolved in the minimum volume of dichloromethane and purified via flash chromatography (ratio of silica to crude mass = 10:1, eluted with 4:1 CH_2Cl_2:CH_3OH). For characterization purposes, a small amount of regioisomerically pure (−)-xestoquinolide B **30** was obtained via repeating the flash chromatography procedure.

Yield: 38.5 mg, 0.0905 mmol, 69%, regioselectivity = 6:1 (xestoquinolide B:C);

Appearance: orange solid

R*f*: 0.13 (major, xestoquinolide B) and 0.18 (minor, xestoquinolide C) (19:1 $CHCl_3$:CH_3OH)

For diagnostic peaks tentatively assigned to xestoquinolide C, see the NMR screenshots later in this document.

The following data was collected using regioisomerically pure xestoquinolide B:

^{1}H NMR (600 MHz, $CDCl_3$): δ 8.98 (s, 1H), 8.33 (s, 1H), 7.08 (s, 1H), 6.82 (s, 1H), 5.90 (s, 1H), 4.17 – 4.14 (m, 2H), 3.38 (app dd, *J* = 6.4, 6.4 Hz, 2H), 2.56 – 2.52 (m, 1H), 2.47 – 2.41 (m, 2H), 2.28 (s, 3H), 1.71 (ddd, *J* = 12.7, 12.3, 6.4 Hz, 1H), 1.47 (s, 3H) ppm;

IR: 3494, 3261, 2959, 2927, 2864, 2212, 1768, 1609, 1445, 1273, 1106 cm^{-1};

HRMS (ESI): calculated for $[C_{22}H_{19}NO_6S+H]^+$: 426.1006, found: 426.0989;

$[\alpha]_D^{25}$: −0.86° (c = 0.004, $CHCl_3$).

Note: Attempts to obtain ^{13}C NMR data in $CDCl_3$ were unsuccessful (low solubility). While improved solubility was observed in CD_3CN, the requisite long-range HMBC couplings necessary to assign regioselectivity were not observed. Satisfactory data was obtained in $(CD_3)_2SO$:

^{1}H NMR [600 MHz, $(CD_3)_2SO$]: δ 9.24 (s, 1H), 8.97 (s, 1H), 8.60 (s, 1H), 8.37 (s, 1H), 5.88 (s, 1H), 3.87 (s, 2H), 3.39 (app t, *J* = 5.1 Hz, 2H), 2.53 (app d, *J* = 6.9 Hz, 2H), 2.33 (d, *J* = 18.4 Hz, 1H), 2.20 (s, 3H), 1.55 (app d, *J* = 6.9 Hz, 1H), 1.41 (s, 3H) ppm;

^{13}C NMR [150 MHz, $(CD_3)_2SO$]: δ 178.9, 178.4, 173.7, 154.2, 147.3, 143.2, 133.3, 132.9, 132.3, 131.6, 131.2, 130.9, 125.3, 123.7, 111.5, 48.3, 39.7 [hidden beneath $(CD_3)_2SO$, assigned by HSQC], 39.2 [hidden beneath $(CD_3)_2SO$, assigned by HSQC], 33.2, 26.9, 23.6, 23.1 ppm;

NMR assignments and key HMBC correlations for the assignment of regioselectivity:

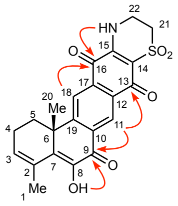

(−)-30: *ent*-xestoquinolide B

#	^{1}H NMR	^{13}C NMR
**1**	2.20 (s, 3H)	23.6
**2**	–	131.6
**3**	5.88 (s, 1H)	131.2
**4a**	2.53 (app d, *J* = 6.9 Hz, 2H)	23.1
**4b**	2.33 (d, *J* = 18.4 Hz, 1H)	
**5a**	included within **4a**	33.2
**5b**	1.55 (app d, *J* = 6.9 Hz, 1H)	
**6**	–	39.2
**7**	–	133.3
**8**	–	143.2
**9**	–	178.9
**10**	–	154.2
**11**	8.60 (s, 1H)	123.7
**12**	–	132.3
**13**	–	173.7
**14**	–	147.3
**15**	–	111.5
**16**	–	178.4
**17**[a]	–	132.9
**18**	8.37 (s, 1H)	125.3
**19**[a]	–	130.9
**20**	1.41 (s, 3H)	26.9
**21a**	3.87 (s, 2H)	39.7
**21b**		
**22a**	3.39 (app t, *J* = 5.1 Hz, 2H)	48.3
**22b**		
**OH**	8.97 (s, 1H)	–
**NH**	9.24 (s, 1H)	–

[a]Assignments are interchangeable.

Comparison of Isolated Natural Products with Synthetic Samples

Cyclozonarone

Comparison of data for isolated cyclozonarone[27] and synthetic *ent*-cyclozonarone.

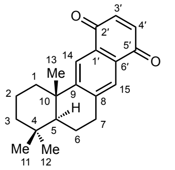

(+)-2: *ent*-cyclozonarone

**isolated cyclozonarone**	**synthetic *ent*-cyclozonarone**
**appearance**	**appearance**
oil	yellow oil
**specific rotation** ($[\alpha]^{19}_{D}$, c = 0.330, $CHCl_3$)	**specific rotation** ($[\alpha]^{25}_{D}$, c = 0.238, $CHCl_3$)
−89.1°	+81.1°

	^{1}H NMR			^{13}C NMR		
**#**	**isolated**[a] CDCl$_3$, 400 MHz	**synthetic**[b] CDCl$_3$, 500 MHz	**Δ**	**isolated**[c] CDCl$_3$, 100 MHz	**synthetic**[d] CDCl$_3$, 125 MHz	**Δ**
**1a**	1.43 (m, 1H)	1.42 (app td, *J* = 13.1, 3.9 Hz, 1H)	0.01	38.6	38.7	0.1
**1b**	2.44 (br dd, *J* = 11.7 Hz, 1H)	2.43 (d, *J* = 12.8 Hz, 1H)	0.01			
**2a**	1.67 (m, 1H)	1.69 – 1.64 (m, 1H)	–	41.5	41.6	0.1
**2b**	1.76 (m, 1H)	1.83 – 1.70 (m, 2H)	–			
**3a**	1.22 (m, 1H)	1.24 (dd, *J* = 13.6, 4.0 Hz, 1H)	0.02	19.1	19.2	0.1
**3b**	1.52 (m, 1H)	1.51 (d, *J* = 14.8 Hz, 1H)	0.01			
**4**	–	–	–	33.6	33.7	0.1
**5**	1.31 (dd, *J* = 12.7, 2.4 Hz, 1H)	1.31 (dd, *J* = 12.6, 2.6 Hz, 1H)	0	49.8	49.9	0.1
**6a**	1.78 (m, 1H)	included within **2b**		16.6	18.7	2.1
**6b**	1.95 (br dd, *J* = 13.7, 7.8 Hz, 1H)	1.95 (dd, *J* = 13.5, 7.9 Hz, 1H)	0			
**7a**	2.97 (ddd, *J* = 18.0, 10.7, 7.8 Hz, 1H)	2.96 (ddd, *J* = 18.7, 11.1, 7.6 Hz, 1H)	0.01	30.7	30.8	0.1
**7b**	3.10 (dd, *J* = 18.0, 6.9 Hz, 1H)	3.09 (dd, *J* = 18.3, 7.0 Hz, 1H)	0.01			
**8**	–	–	–	142.9	143.0	0.1
**9**	–	–	–	157.0	157.1	0.1
**10**	–	–	–	38.7	39.8	1.1
**11**[e]	0.97 (s, 3H)	0.95 (s, 3H)	0.02	21.7	21.8	0.1
**12**[e]	0.99 (s, 3H)	0.97 (s, 3H)	0.02	33.2	33.3	0.1
**13**	1.21 (s, 3H)	1.20 (s, 3H)	0.01	24.5	24.6	0.1
**14**	7.98 (s, 1H)	7.98 (s, 1H)	0	127.4	127.5	0.1
**15**	7.74 (s, 1H)	7.70 (s, 1H)	0.01	123.1	123.2	0.1
**1′**	–	–	–	129.1	129.2	0.1
**2′**	–	–	–	185.3	185.4	0.1
**3′**	6.90 (d, *J* = 10.3 Hz, 1H)	6.90 (d, *J* = 10.4 Hz, 1H),	0	138.6	138.7	0.1
**4′**	6.89 (d, *J* = 10.3 Hz, 1H)	6.88 (d, *J* = 10.4 Hz, 1H)	0.01	138.9	139.0	0.1
**5′**	–	–	–	185.3	185.4	0.1
**6′**	–	–	–	129.8	129.9	0.1

[a]Referenced to TMS. [b]Referenced to 7.26 ppm. [c]Referenced to TMS. [d]Referenced to 77.16 ppm. [e]Assignments are interchangeable.

Note: NMR screenshots of isolated cyclozonarone were not published.

Neopetrosiquinone A

Comparison of data for isolated neopetrosiquinone A[28] and synthetic *ent*-neopetrosiquinone A.

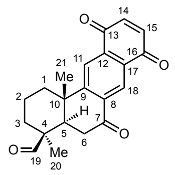

(+)-3: *ent*-neopetrosiquinone A

isolated neopetrosiquinone A	synthetic *ent*-neopetrosiquinone A
**appearance**	**appearance**
yellow oil	yellow solid
**specific rotation** ($[\alpha]^{25}_{D}$, c = 0.006, $CHCl_3$)[a]	**specific rotation** ($[\alpha]^{25}_{D}$, c = 0.015, $CHCl_3$)
−40.0°	+39.8°

[a]Measured by us using an authentic sample of isolated neopetrosiquinone A provided by the original isolation chemists (Amy E. Wright, Florida Atlantic University).

	^{1}H NMR			^{13}C NMR		
**#**	**isolated**[a] CD_2Cl_2, 600 MHz	**synthetic**[a] (CD_2Cl_2, 500 MHz	**Δ**	**isolated**[b] CD_2Cl_2, 200 MHz	**synthetic**[c] CD_2Cl_2, 125 MHz	**Δ**
**1a**	2.55 (br dt, *J* = 13.1, 3.4, Hz, 1H)	2.55 (app dt, *J* = 12.9, 3.9 Hz, 1H)	0	37.1	37.7	0.6
**1b**	1.62 (m, 1H)	1.65 – 1.59 (m, 1H)	–			
**2a**	1.82 (m, 2H)	1.85 – 1.80 (m, 2H)	–	18.6	19.3	0.7
**2b**						
**3a**	2.23 (dt, 1H)	2.55 – 2.18 (m, 2H)	–	34.1	34.7	0.6
**3b**	1.23 (m, 1H)	1.26 – 1.22 (m, 1H)	–			
**4**	–	–	–	48.7	48.0	0.7
**5**	2.19 (dd, 1H)	included within **3a**	–	49.0	49.4	0.4
**6a (eq)**	3.14 (dd, 1H)	3.20 – 3.06 (m, 2H)	–	35.2	35.7	0.5
**6b (ax)**	3.05 (dd, 1H)		–			
**7**	–	–	–	194.8	196.3	1.5
**8**	–	–	–	134.2	134.7	0.5
**9**	–	–	–	159.1	159.7	0.6
**10**	–	–	–	38.8	39.4	0.6
**11**	8.16 (s, 1H)	8.15 (s, 1H)	0.1	123.5	123.7	0.2
**12**[e]	–	–	–	134.7	135.2	0.5
**13**	–	–	–	184.3	184.8	0.5
**14**[d]	6.99 (d, 1H)	7.01 (app s, 2H)	–	138.6	139.2	0.8
**15**[d]	6.98 (d, 1H)			139.1	139.7	0.6
**16**	–	–	–	183.7	184.2	0.5
**17**[e]	–	–	–	130.0	130.6	0.6
**18**	8.64 (s, 1H)	8.63 (s, 1H)	0.1	126.5	126.5	0
**19**	9.88 (s, 1H)	9.88 (s, 1 H)	0	204.3	204.8	0.5
**20**	1.12 (s, 3H)	1.13 (s, 3H)	0.1	22.3	23.0	0.7
**21**	1.18 (s, 3H)	1.18 (s, 3H),	0	23.3	23.9	0.6

[a]Referenced to 5.32 ppm. [b]Originally referenced to 54 ppm, shifts adjusted herein to match our reference of 53.84 ppm. [c]Referenced to 53.84 ppm. [d]Assignments are interchangeable. [e]Assignments are interchangeable.

NMR Screenshots

Isolated Neopetrosiquinone A, ^{1}H NMR:

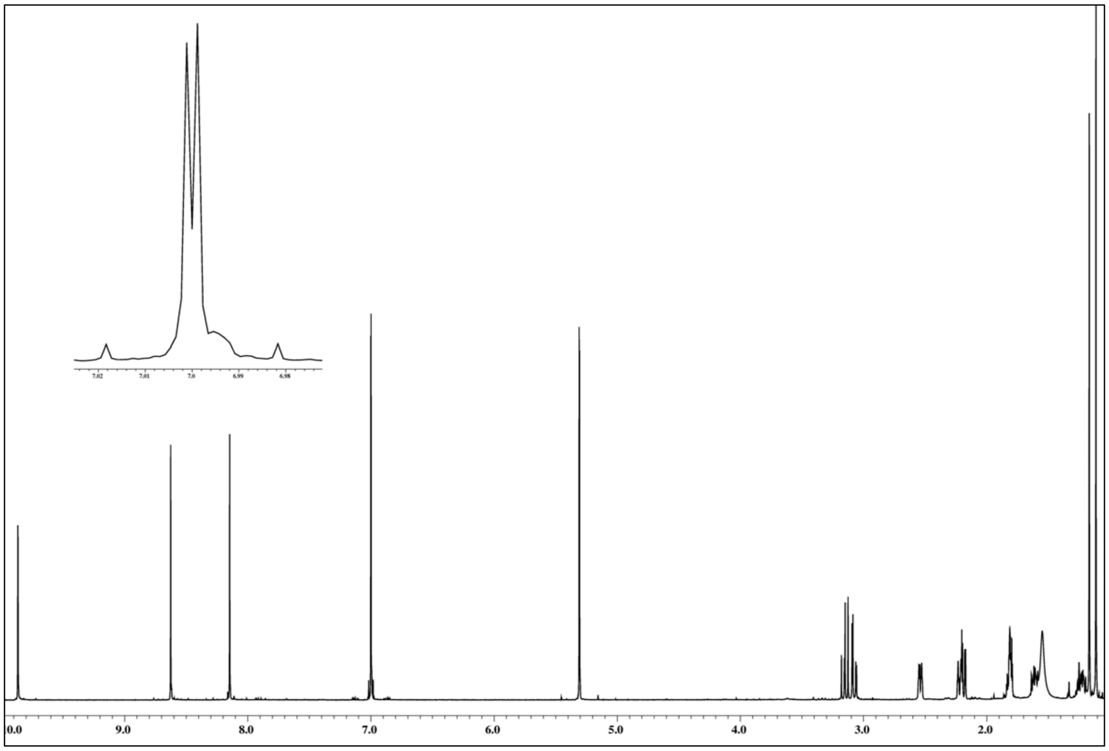

Synthetic *ent*-Neopetrosiquinone A, ^{1}H NMR:

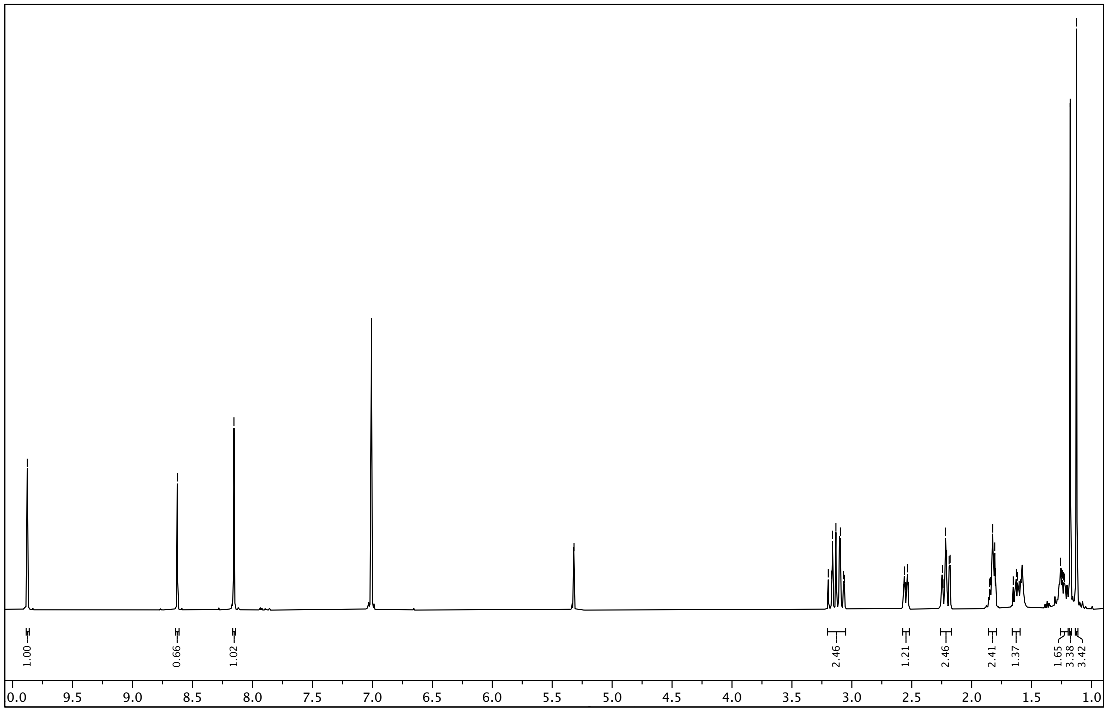

Isolated Neopetrosiquinone A, ^{13}C NMR:

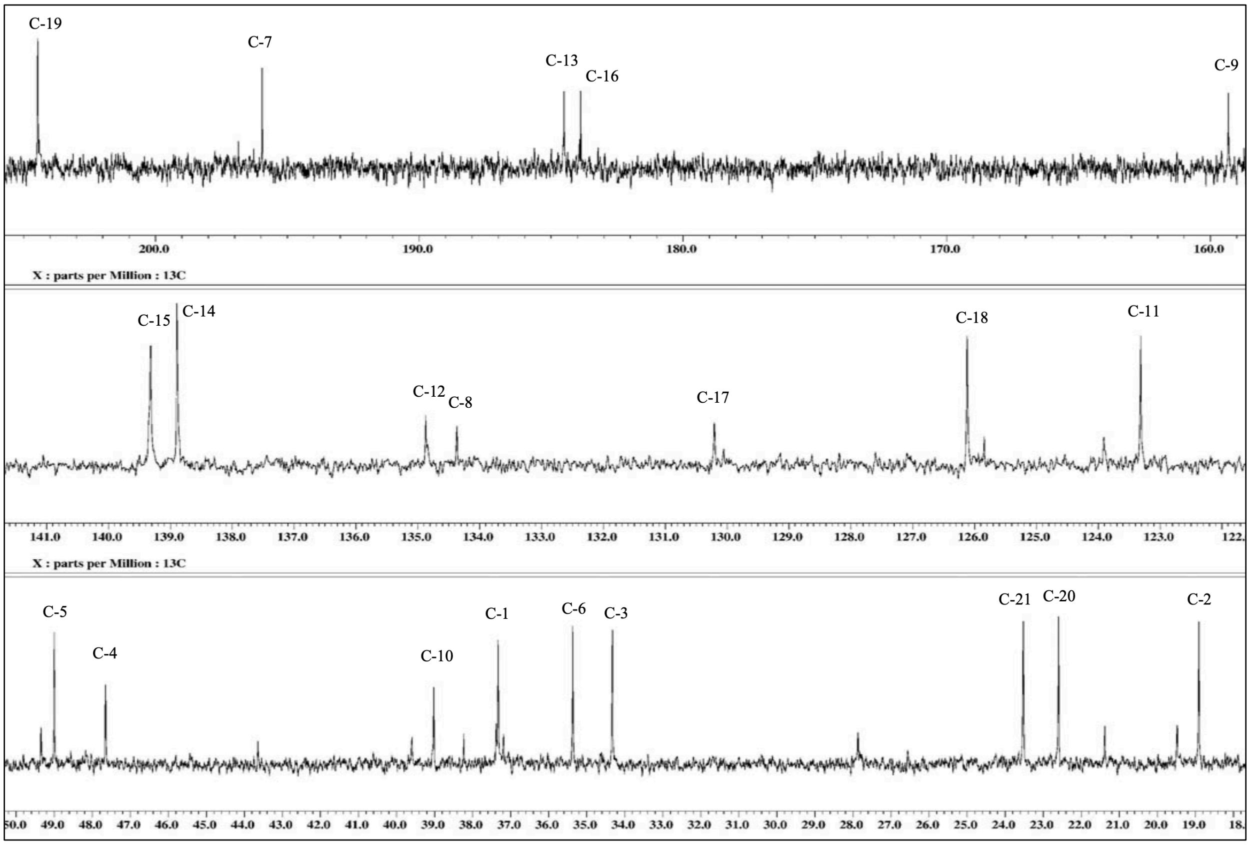

Synthetic *ent*-Neopetrosiquinone A, ^{13}C NMR:

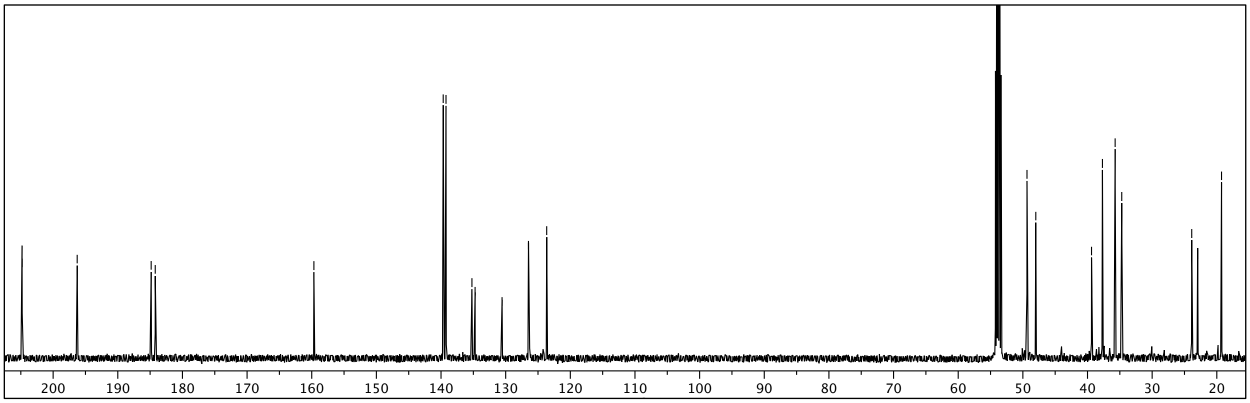

Neopetrosiquinone B

Comparison of data for isolated neopetrosiquinone B[28] and synthetic *ent*-neopetrosiquinone B.

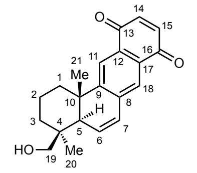

(−)-21: *ent*-neopetrosiquinone B

isolated neopetrosiquinone B	synthetic *ent*-neopetrosiquinone B
**appearance**	**appearance**
yellow oil	yellow oil
**specific rotation**[a]	**specific rotation** ($[\alpha]^{25}_{D}$, c = 0.66, $CHCl_3$)
not reported	−163°

[a]An authentic sample of isolated neopetrosiquinone B could not be obtained from the original isolation chemists (decomposition).

#	^{1}H NMR			^{13}C NMR		
	**isolated**[a] $CDCl_3$, 600 MHz	**synthetic**[b] $CDCl_3$, 500 MHz	**Δ**	**isolated**[c] $CDCl_3$, 150 MHz	**synthetic**[d] $CDCl_3$, 125 MHz	**Δ**
**1a**	2.35 (br m, 1H)	2.35 – 2.34 (m, 1H)	–	36.1	36.0	0.1
**1b**	1.77 (br m, 1H)	1.79 – 1.74 (m, 3H)	–			
**2a**	1.77 (br m, 2H)		–	18.7	18.6	0.1
**2b**			–			
**3a**	1.90 (br d, *J* = 15.1 Hz, 1H)	1.90 (d, *J* = 11.9 Hz, 1H)	0	35.3	35.3	0
**3b**	1.13 (br m, 1H)	1.16 – 1.12 (m, 1H)	–			
**4**	–	–	–	38.6	38.6	0
**5**	2.31 (t, *J* = 3.4, 1H)	2.31 (app t, *J* = 3.2, 1H)	0	50.6	50.5	0.1
**6**	6.44 (dd, *J* = 9.6, 2.8 Hz, 1H)	6.43 (dd, *J* = 9.7, 2.8 Hz, 1H)	0.1	134.5	134.4	0.1
**7**	6.69 (dd, *J* = 9.6, 3.4 Hz, 1H)	6.68 (dd, *J* = 9.7, 3.2 Hz, 1H)	0.1	126.9	126.8	0.1
**8**	–	–	–	138.7	138.6	0.1
**9**	–	–	–	153.8	153.8	0
**10**	–	–	–	38.5	38.5	0
**11**	7.89 (s, 1H)	7.88 (s, 1H)	0.1	120.8	120.7	0.1
**12**[e]	–	–	–	131.2	131.1	0.1
**13**	–	–	–	185.2	185.3	0.1
**14**[f]	6.93 (d, *J* = 10.3 Hz, 1H)	6.93 (d, *J* = 10.4 Hz, 1H)	0	139.1	139.0	0.1
**15**[f]	6.91 (d, *J* = 10.3 Hz, 1H)	6.90 (d, *J* = 10.4 Hz, 1H)	0	138.6	138.2	0.4
**16**	–	–	–	185.2	185.1	0.1
**17**[e]	–	–	–	130.5	130.5	0
**18**	7.72 (s, 1H)	7.71 (s, 1H)	0.1	124.3	124.3	0
**19a**	3.83 (d, *J* = 11.7 Hz, 1H)	3.84 (d, *J* = 11.1 Hz, 1H)	0.1	66.3	66.2	0.1
**19b**	3.76 (d, *J* = 11.0 Hz, 1H)	3.76 (d, *J* = 11.1 Hz, 1H)	0			
**20**	1.11 (s, 3H)	1.10 (s, 3H)	0.1	26.3	26.2	0.1
**21**	1.08 (s, 3H)	1.07 (s, 3H)	0.1	20.8	20.7	0.1
**OH**	not reported	not observed	–	–	–	–

[a]Originally referenced to 7.24 ppm, shifts adjusted herein to match our reference of 7.26 ppm. [b]Referenced to 7.26 ppm. [c]Originally referenced to 77.2 ppm, shifts adjusted herein to match our reference of 77.16 ppm. [d]Referenced to 77.16 ppm. [e]Assignments are interchangeable. [f]Assignments are interchangeable.

NMR Screenshots

Isolated Neopetrosiquinone B, ^{1}H NMR:

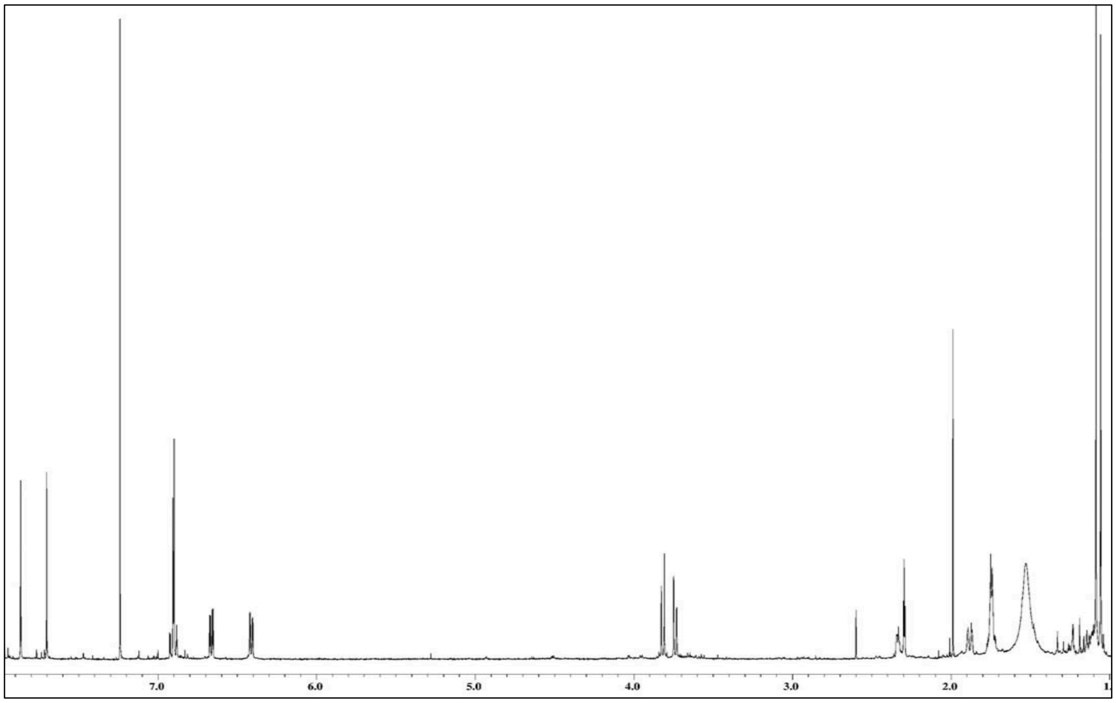

Synthetic *ent*-Neopetrosiquinone B, ^{1}H NMR:

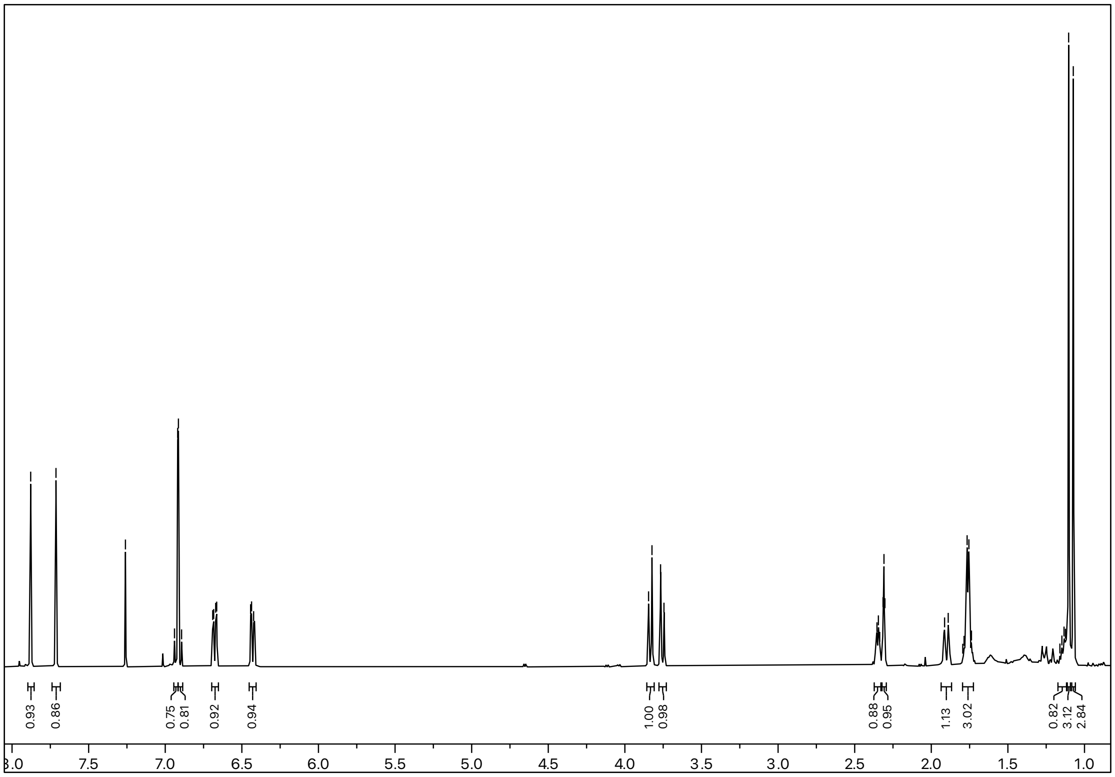

Isolated Neopetrosiquinone B, ^{13}C NMR:

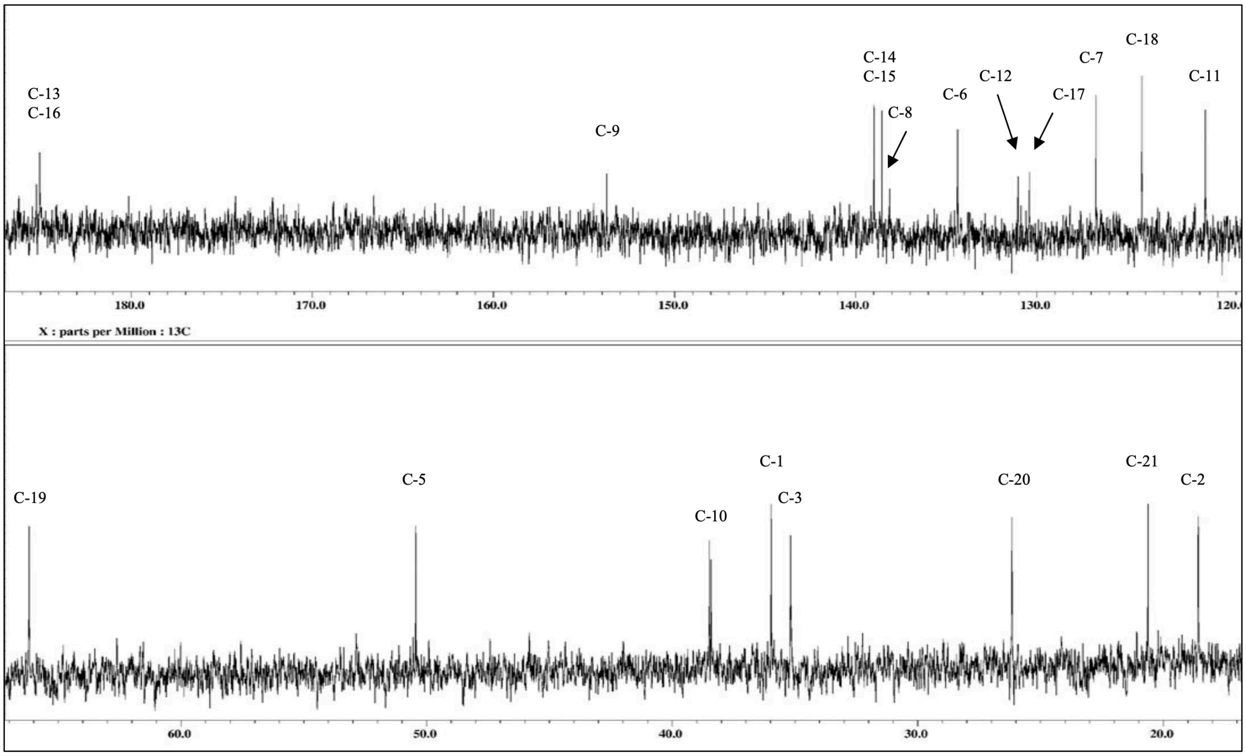

Synthetic *ent*-Neopetrosiquinone B, ^{13}C NMR:

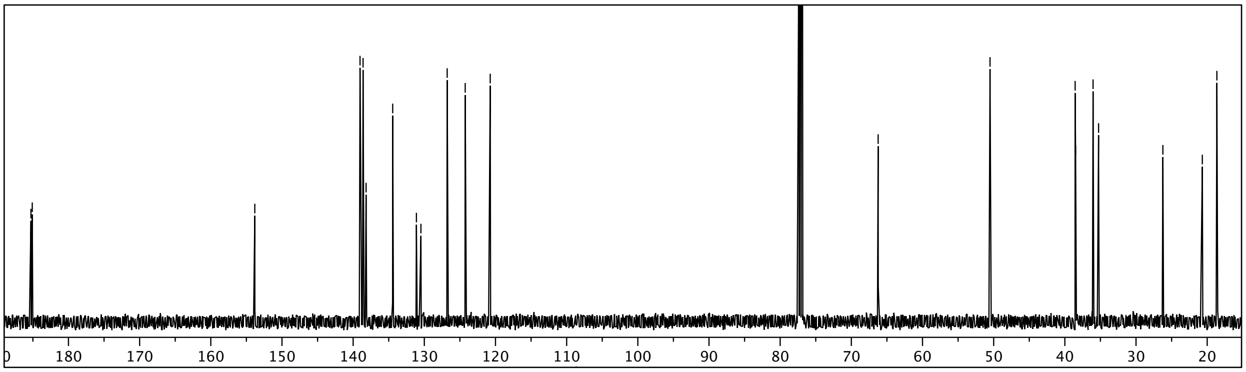

Orhalquinone

Comparison of data for isolated orhalquinone[29] and synthetic *ent*-orhalquinone.

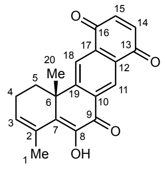

(−)-5: *ent*-orhalquinone

isolated (orhalquinone	synthetic *ent*-orhalquinone
**appearance**	**appearance**
orange powder	orange powder
**specific rotation** ($[\alpha]^{20}_{D}$, c = 0.01, CH_3OH)	**specific rotation** ($[\alpha]^{25}_{D}$, c = 0.96, $CHCl_3$)
+122°	−159°

	^{1}H NMR			^{13}C NMR		
**#**	**isolated**[a] CDCl₃, 600 MHz	**synthetic**[b] CDCl₃, 500 MHz	**Δ**	**isolated**[c] CDCl₃, 150 MHz	**synthetic**[d] CDCl₃, 125 MHz	**Δ**
**1**	2.29 (br s, 3H)	2.29 (br s, 3H)	0	23.3	23.5	0.2
**2**[e]	–	–		131.9	132.0	0.1
**3**	5.90 (br s, 1H)	5.90 (s, 1H)	0	131.8	131.9	0.1
**4a**	2.55 (m, 1H)	2.61 – 2.52 (m, 1H)	–	23.5	23.7	0.2
**4b**	2.42 (br dm, *J* = 19.2, 1H)	2.45 – 2.39 (m, 1H)	–			
**5a**	2.49 (dd, *J* = 13.0, 6.3, 1H)	2.49 (dd, *J* = 13.0, 6.3 Hz, 1H)	0	33.6	33.7	0.1
**5b**	1.70 (ddd *J* = 12.9, 11.8, 6.3, 6.3, 1H)	1.69 (ddd, *J* = 12.2, 12.1, 6.4 Hz, 1H)	0.01			
**6**	–	–	–	39.5	39.7	0.2
**7**	–	–	–	133.1	133.2	0.1
**8**	–	–	–	142.5	142.6	0.1
**9**	–	–	–	179.3	179.5	0.2
**10**[e]	–	–	–	132.0	132.2	0.2
**11**	8.92 (s, 1H)	8.92 (s, 1H)	0	126.0	126.1	0.1
**12**	–	–	–	130.2	130.4	0.2
**13**	–	–	–	183.7	183.8	0.1
**14**	7.05 (ABq, *J* = 10.4, 1H)	7.06 (s, 1H),	0.01	138.9	139.1	0.1
**15**	7.07 (ABq, *J* = 10.4, 1H)	7.07 (s, 1H),	0	139.5	139.6	0.1
**16**	–	–	–	184.5	184.7	0.2
**17**	–	–	–	133.7	133.9	0.2
**18**	8.34 (s, 1H)	8.34 (s, 1H)	–	125.1	125.3	0.2
**19**	–		–	156.3	156.4	0.1
**20**	1.48 (s, 3H)	1.48 (s, 3H)	0	27.4	27.6	0.2
**OH**	7.09 (s, 1H)	7.09 (s, 1H)	0	–	–	–

[a]No reference reported. [b]Referenced to 7.26 ppm. [c]Originally referenced to 77.2 ppm, shifts adjusted herein to match our reference of 77.16 ppm. [d]Referenced to 77.16 ppm. [e]Assignments are interchangeable.

NMR Screenshots

Isolated Orhalquinone, ^{1}H NMR:

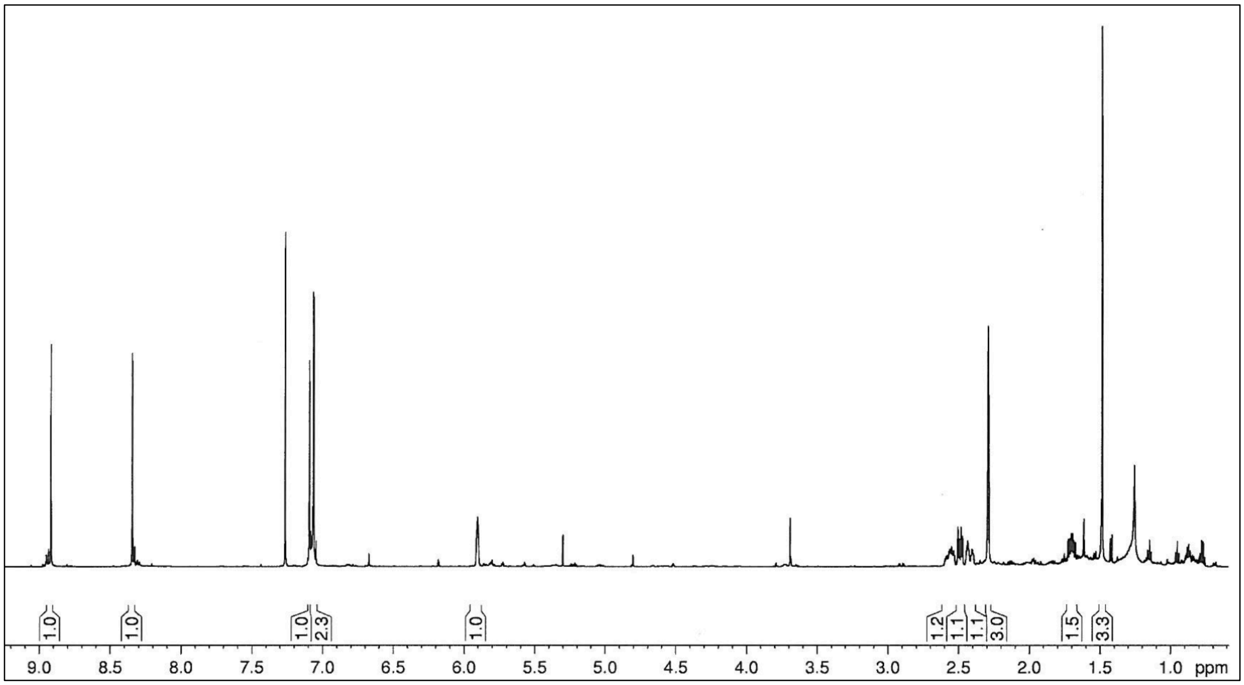

Synthetic *ent*-Orhalquinone, ^{1}H NMR:

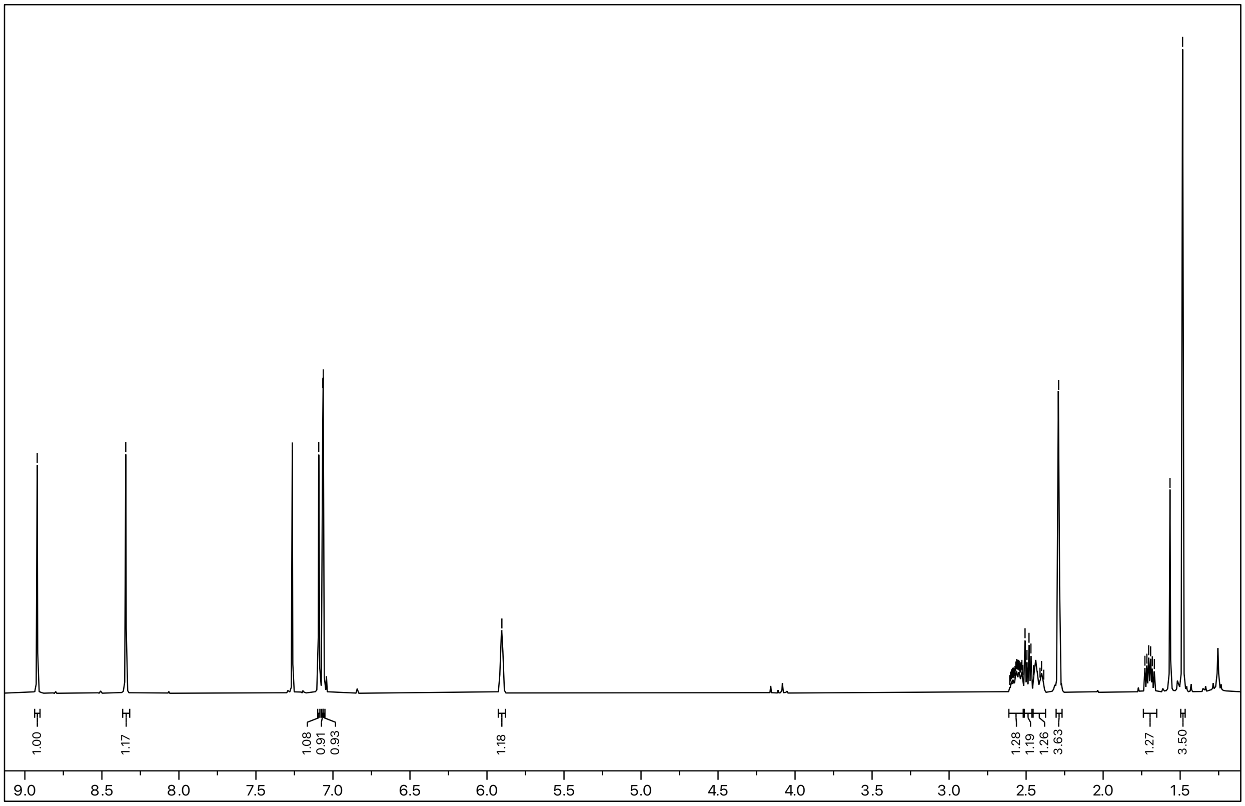

Isolated Orhalquinone, ^{13}C NMR:

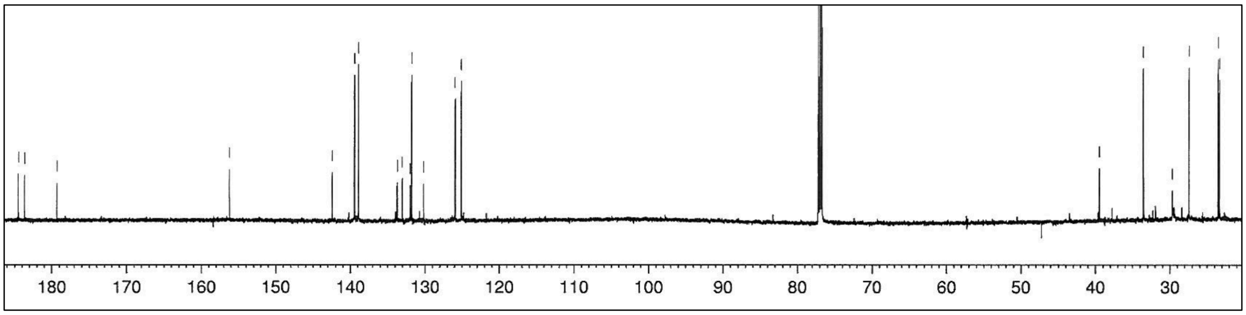

Synthetic *ent*-Orhalquinone, ^{13}C NMR:

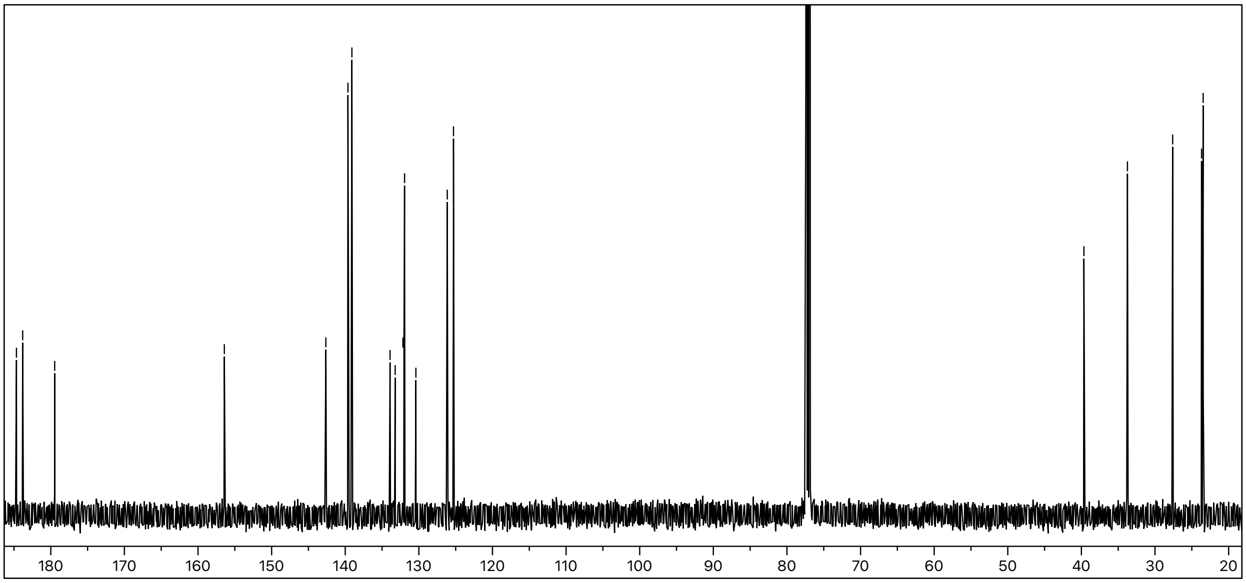

Xestoquinone

Comparison of data for isolated xestoquinone[30] and synthetic *ent*-xestoquinone.

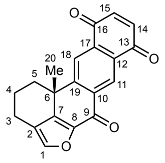

(−)-7: *ent*-xestoquinone

isolated xestoquinone	synthetic *ent*-xestoquinone
**appearance**	**appearance**
yellow powder	yellow-orange solid
**specific rotation** ($[\alpha]^{25}_{D}$, c = 1.16, CH_2Cl_2)	**specific rotation** ($[\alpha]^{25}_{D}$, c = 1.00, CH_2Cl_2)
+17.2°	−21.1°

	**^{1}H NMR**			**^{13}C NMR**		
**#**	**isolated**[a] CDCl$_3$, 270 MHz	**synthetic**[b] CDCl$_3$, 600 MHz	**Δ**	**synthetic (Gao)**[c] CDCl$_3$, 125 MHz	**synthetic (Us)**[d] CDCl$_3$, 150 MHz	**Δ**
**1**	7.62 (t, *J* = 2 Hz, 1H)	7.54 (s, 1H)	0.08	145.2	145.1	0.1
**2**	–	–	–	121.7	121.6	0.1
**3a**	2.68 (dddd, *J* = 17, 10, 9, 2, Hz, 1H)	2.65 (ddd, *J* = 16.9, 10.1, 10.1 Hz, 1H)	0.03	18.6	18.6	0
**3b**	2.92 (dddd, *J* = 17, 8, 3, 2, Hz, 1H)	2.89 (dd, *J* = 16.7, 7.9, Hz, 1H)	0.03			
**4a**	2.1 – 2.4 (m, 2H)	2.33 – 2.24 (m, 1H)	–	17.1	17.1	0
**4b**		2.20 – 2.16 (m, 1H)	–			
**5a**	1.78 (ddd, *J* = 13, 13, 5, Hz, 1H)	1.76 (ddd, *J* = 13.0, 13.0, 4.6 Hz, 1H),	0.02	31.3	31.4	0.1
**5b**	2.59 (ddd, *J* = 13, 4, 4 Hz, 1H)	2.58 (ddd, *J* = 12.9, 3.4, 3.4 Hz, 1H)	0.01			
**6**	–	–	–	37.5	37.5	0
**7**	–	–	–	144.1	144.3	0.2
**8**	–	–	–	147.5	147.4	0.1
**9**	–	–	–	170.3	170.4	0.1
**10**	–	–	–	156.4	156.4	0
**11**	9.14 (s, 1H)	9.06 (s, 1H)	0.08	127.0	127.3	0.3
**13**	–	–	–	130.4	130.6	0.2
**13**	–	–	–	184.8	184.9	0.1
**14**	7.12 (s, 2H)	7.06 (d, J = 10.3 Hz, 1H)	0.06	139.5	139.6	0.1
**15**		7.04 (d, J = 10.3 Hz, 1H)	0.08	138.8	138.8	0
**16**	–	–	–	184.0	184.0	0
**17**	–	–	–	133.3	133.4	0.1
**18**	8.32 (s, 1H)	8.25 (s, 1H)	0.07	123.3	123.4	0.1
**19**	–	–	–	138.0	138.2	0.2
**20**	1.52 (s, 3H)	1.54 (s, 3H)	0.02	32.8	32.7	0.1

[a]No reference reported. [b]Referenced to 7.26 ppm. [c]Originally referenced to 77.0 ppm, shifts adjusted herein to match our reference of 77.16 ppm. [d]Referenced to 77.16 ppm.

Note: Carbon NMR shifts compared with Gao's synthetic sample.[12]

Note: NMR screenshots of isolated xestoquinone were not published.

Halenaquinone

Comparison of data for isolated halenaquinone[31] and synthetic *ent*-halenaquinone.

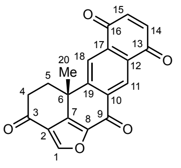

(−)-1: *ent-h*alenaquinone

isolated halenaquinone	synthetic *ent*-halenaquinone
**appearance**	**appearance**
yellow powder	Yellow-orange solid
**specific rotation** ($[\alpha]^{25}_{D}$, c = 0.124, CH_2Cl_2)	**specific rotation** ($[\alpha]^{25}_{D}$, c = 0.12, CH_2Cl_2)
+22.2°	−18.9°

	^{1}**H NMR**			^{13}**C NMR**		
**#**	**isolated**[a] $(CD_3)_2SO$, 300 MHz	**synthetic**[b] $(CD_3)_2SO$, 400 MHz	**Δ**	**isolated**[a] $(CD_3)_2SO$, 75.6 MHz	**synthetic**[c] $(CD_3)_2SO$, 225 MHz	**Δ**
**1**	8.76 (s, 1H)	8.90 (s, 1H)	0.14	150.4	151.0	0.6
**2**	–			122.1	122.5	0.4
**3**	–			190.9	191.6	0.7
**4a (α)**	2.22 (ddd, 1H)	2.21 (ddd, 1H)	−0.01	32.3	32.3	0
**4b (β)**	2.94 (dd, 1H)	2.94 (dd, 1H)				
**5a (α)**	2.74 (dd, 1H)	2.67 (dd, 1H)	−0.07	36.1	36.3	0.2
**5b (β)**	3.11 (ddd, 1H)	3.10 (ddd, 1H)	−0.01			
**6**	–			36.4	36.7	0.3
**7**	–			143.9	144.0	0.1
**8**	–			147.9	148.6	0.7
**9**	–			169.5	170.0	0.5
**10**	–			154.1	154.6	0.5
**11**	8.66 (s, 1H)	8.72 (s, 1H)	0.06	125.2	125.1	0.1
**12**	–			129.9	130.4	0.5
**13**	–			183.3	183.9	0.6
**14**	7.13 (s, 2H)	7.19 (s, 2H)	−0.06	138.7	139.2	0.5
**15**			−0.06	138.8	139.2	0.4
**16**	–			183.8	184.3	0.5
**17**	–			133.3	133.7	0.4
**18**	8.28 (s, 1H)	8.34 (s, 1H)	0.06	123.5	123.8	0.3
**19**	–			136.3	136.4	0.1
**20**	1.68 (s, 3H)	1.66 (s, 3H)	−0.02	29.7	29.8	0.1

[a]No reference reported. [b]Referenced to 2.50 ppm. [c]Referenced to 39.52 ppm. Excellent agreement is observed with more recent isolation reports.[29, 32]

Note: NMR screenshots of isolated halenaquinone were not published.

Xestoquinolide B

Comparison of data for isolated xestoquinolide B[33] and synthetic *ent*-xestoquinolide B.

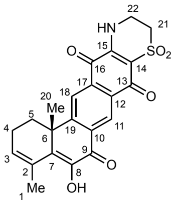

(−)-30: *ent*-xestoquinolide B

**isolated xestoquinolide B**	**synthetic *ent*-xestoquinolide B**
**appearance**	**appearance**
yellow powder	yellow powder
**specific rotation**	**specific rotation** ($[\alpha]^{25}_{D}$, c = 0.004, $CHCl_3$)
not reported	+0.86°

	^{1}**H NMR**		
**#**	**isolated**[a] $CDCl_3$, 250 MHz	**synthetic**[b] $CDCl_3$, 250 MHz	**Δ**
**1**	2.28 (br s, 3H)	2.28 (s, 3H)	0
**2**	–	–	–
**3**	5.90 (br s, 1H)	5.90 (s, 1H)	0
**4a** / **4b**	2.49 (m, 2H)	2.56 – 2.52 (m, 1H)	–
**5a**	2.45 (m, 1H)	2.47 – 2.41 (m, 2H)	–
**5b**	1.70 (m, 1H)	1.71 (ddd, *J* = 12.7, 12.3, 6.4 Hz, 1H)	–
**6**	–	–	–
**7**	–	–	–
**8**	–	–	–
**9**	–	–	–
**10**	–	–	–
**11**	8.99 (s, 1H)	8.98 (s, 1H)	0.01
**12**	–	–	–
**13**	–	–	–
**14**	–	–	–
**15**	–	–	–
**16**	–	–	–
**17**	–	–	–
**18**	8.33 (s, 1H)	8.33 (s, 1H)	0
**19**	–	–	–
**20**	1.47 (s, 3H)	1.47 (s, 3H)	0
**21a** / **21b**	4.18 (m, 2H)	4.17 – 4.14 (m, 2H)	–
**22a** / **22b**	3.40 (m, 2H)	3.38 (dd, *J* = 6.2 Hz, 2H)	–
**OH**	not reported	7.08 (s, 1H)	–
**NH**	not reported	6.82 (s, 1H)	–

[a]No reference reported. [b]Referenced to 7.26 ppm. [c]Assignments are interchangeable.

Note: ^{13}C NMR data of isolated xestoquinolide B was not published.

NMR Screenshots

Isolated Xestoquinolide B, ^{1}H NMR:

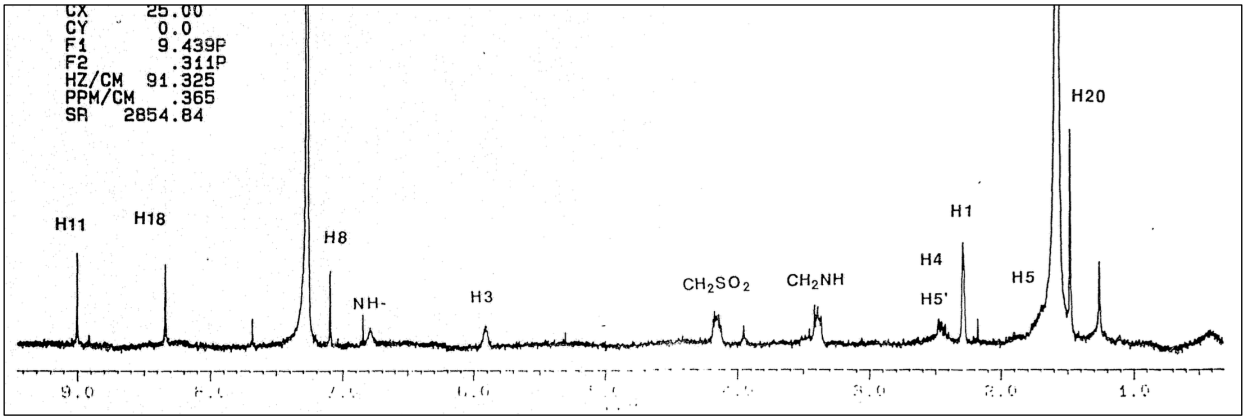

Synthetic *ent*-Xestoquinolide B, ^{1}H NMR:

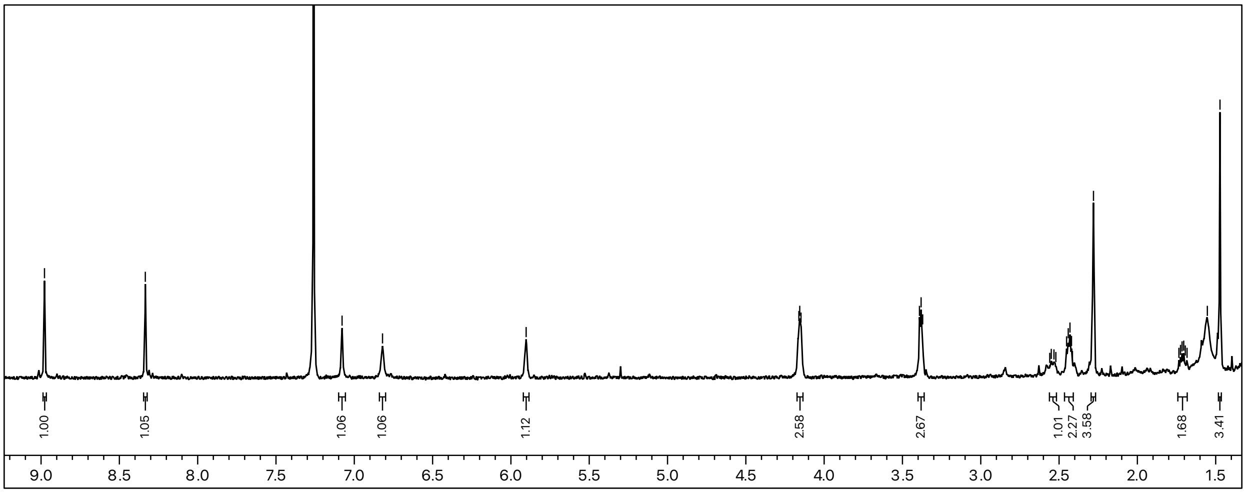

Natural Product Structural Revisions

Xestoquinolide A is Orhalquinone

Comparison of data for isolated xestoquinolide A[33] and isolated orhalquinone.[29]

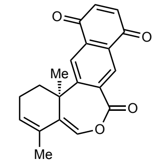

xestoquinolide A
originally reported structure

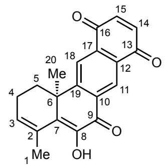

orhalquinone
revised structure

**isolated xestoquinolide A**	**isolated orhalquinone**
**appearance**	**appearance**
orange powder	orange powder
**specific rotation** ($[\alpha]_D$, conc. and temp. not reported, MeOH)	**specific rotation** ($[\alpha]^{20}_D$, c = 0.01, MeOH)
+32°	+122°

	^{1}H NMR			^{13}C NMR		
**#**	**(*nat*)-xestoquinolide A**[a] $CDCl_3$, 250 MHz	**(*nat*)-orhalquinone**[a] $CDCl_3$, 600 MHz	**Δ**	**(*nat*)-xestoquinolide A**[a] $CDCl_3$, 75 MHz	**(*nat*)-orhalquinone**[b] $CDCl_3$, 150 MHz	**Δ**
**1**	2.28 (br s, 3H)	2.29 (br s, 3H)	0.1	23.3	23.3	0
**2**	–	–	–	131.8[d]	131.9[c]	0.1
**3**	5.90 (br s, 1H)	5.90 (br s, 1H)	0	131.8	131.8	0
**4a**	2.49 (m, 2H)	2.55 (m, 1H)	–	23.6	23.5	0.1
**4b**	2.45 (m, 1H)	2.42 (br dm, *J* = 19.2, 1H)	0.03			
**5a**	included within **4a**	2.49 (dd, *J* = 13.0, 6.3, 1H)	–	33.6	33.6	0
**5b**	1.70 (m, 1H	1.70 (ddd *J* = 12.9, 11.8, 6.3, 6.3, 1H)	0			
**6**	–	–	–	39.5	39.5	0
**7**	–	–	–	133.1[e]	133.1	0
**8**	–	–	–	142.5[f]	142.5	0
**9**	–	–	–	179.3	179.3	0
**10**	–	–	–	131.8[g]	132.0[c]	0.2
**11**	8.92 (s, 1H)	8.92 (s, 1H)	0	126.0	126.0	0
**12**	–	–	–	130.3[h]	130.2	0.1
**13**	–	–	–	183.7	183.7	0
**14**	7.06 (s, 2H)	7.05 (ABq, *J* = 10.4, 1H)	–	139.1	138.9	0.2
**15**	included within **14**	7.07 (ABq, *J* = 10.4, 1H)	–	139.5[i]	139.5	0
**16**	–	–	–	184.5	184.5	0
**17**	–	–	–	133.8[j]	133.7	0.1
**18**	8.34 (s, 1H)	8.34 (s, 1H)	0	125.2	125.1	0.1
**19**	–	–	–	156.3	156.3	0
**20**	1.48 (s, 3H)	1.48 (s, 3H)	0	27.5	27.4	0.1
**OH**	7.09 (s, 1H)	7.09 (s, 1H)	0	–	–	–

[a]No reference reported. [b]Referenced to 77.0 ppm. [c]Assignments may be interchanged. [d]C2 was misassigned to signal at 133.1 ppm. [e]C7 was misassigned to 142.5 ppm. [f]C8 was misassigned to 139.5 ppm. [g]C10 was misassigned to 130.3 ppm. [h]C12 was misassigned to 133.8 ppm. [i]C15 was misassigned to 139.1 ppm. [j]C17 was misassigned to 133.1 ppm.

Note: ^{13}C NMR screenshots of isolated xestoquinolide A and isolated orhalquinone were not published.

NMR Screenshots

Isolated Xestoquinolide A, ^{1}H NMR:

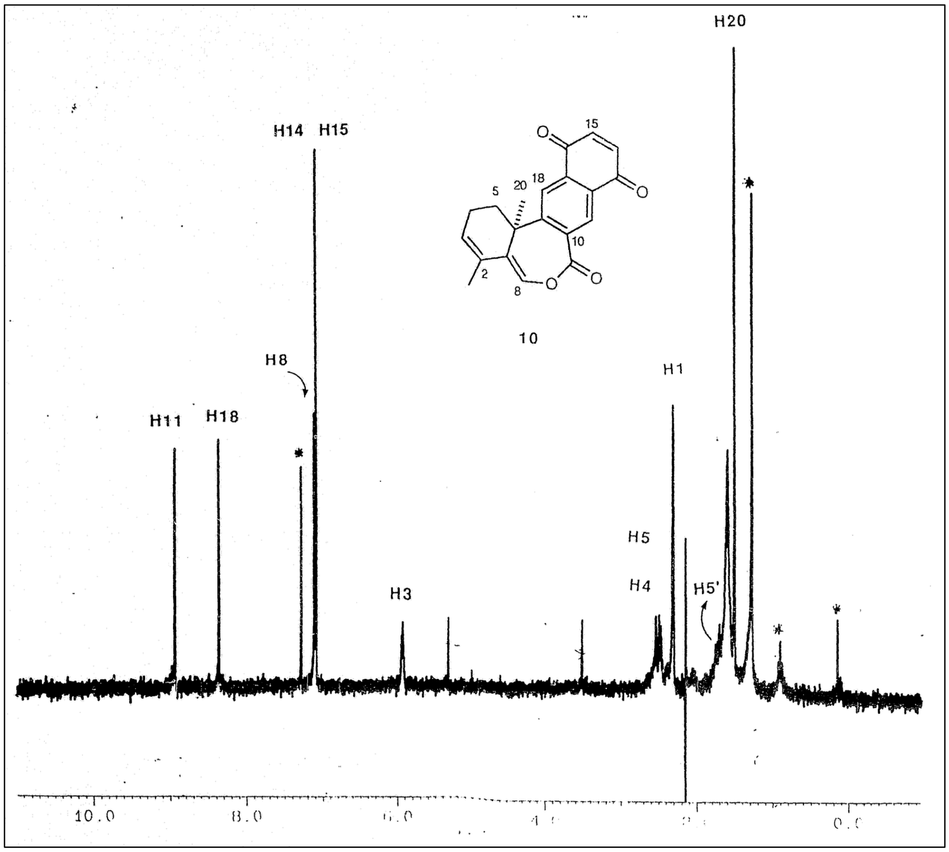

Isolated Orhalquinone, ^{1}H NMR:

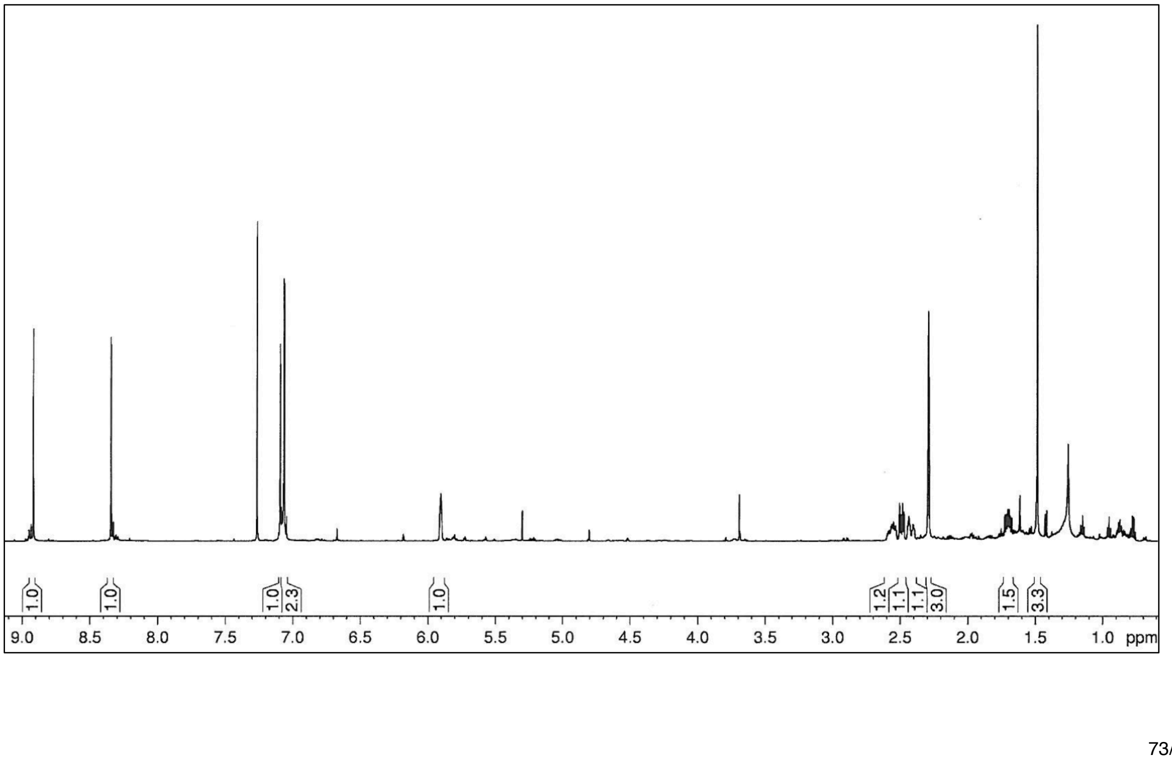

Noelaquinone is 3-Ketoadociaquinone A

Comparison of data for isolated noelaquinone[34] and isolated 3-ketoadociaquinone B.[35]

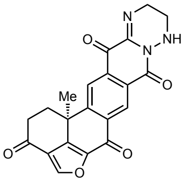

noelaquinone
originally reported structure

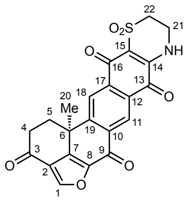

3-ketoadociaquinone B
revised structure

**isolated (*nat*)-noelaquinone**	**isolated (*nat*)-3-ketoadociaquinone B**
**appearance**	**appearance**
yellow solid	yellowish powder
**specific rotation** ($[\alpha]^{23}_{D}$, c = 0.25, MeOH)	**specific rotation** ($[\alpha]^{23}_{D}$, c = 0.12, MeOH)
+53.6°	+13°

	**^{1}H NMR**			**^{13}C NMR**		
**#**	**(*nat*)-noelaquinone**[a] ($(CD_3)_2SO$, 500 MHz)	**(*nat*)-3-ketoadociaquinone B**[a] ($(CD_3)_2SO$, 500 or 400 MHz)	**Δ**	**(*nat*)-noelaquinone**[a] ($(CD_3)_2SO$, 125 MHz)	**(*nat*)-3-ketoadociaquinone B**[a] ($(CD_3)_2SO$, 125 or 100 MHz)	**Δ**
**1**	8.89 (s, 1H)	8.89 (s, H)	0	150.9	151.0	0.2
**2**	–	–	–	122.4	122.5	0.1
**3**	–	–	–	191.5	191.6	0.1
**4a**	3.08 (ddd, 1H)	3.08 (ddd, 1H)	0	32.3	32.3	0
**4b**	2.66 (dd, 1H)	2.65 (dd, 1H)	0.1			
**5a**	2.92 (dd, 1H)	2.93 (ddd, 1H)	0.1	36.5	36.5	0
**5b**	2.19 (ddd, 1H)	2.19 (ddd, 1H)	0			
**6**	–	–	–	36.2	36.3	0.1
**7**	–	–	–	147.2	147.3	0.1
**8**	–	–	–	144.1	144.1	0
**9**	–	–	–	170.0	170.1	0.1
**10**	–	–	–	137.3	137.4	0.1
**11**	8.74 (s, 1H)	8.73 (s, 1H)	0.1	124.9	124.9	0
**12**	–	–	–	132.3	132.3	0
**13**	–	–	–	178.2	178.2	0
**14**	–	–	–	not reported	111.4	–
**15**	–	–	–	148.5	148.6	0.1
**16**	–	–	–	173.6	173.6	0
**17**	–	–	–	131.1	131.2	0.1
**18**	8.34 (s, 1H)	8.34 (s, 1H)	0	124.1	124.2	0.1
**19**	–	–	–	153.3	153.3	0
**20**	1.64 (s, 3H)	1.64 (s, 3H)	0	29.7	29.7	0
**21**	3.87 (m, 1H)	3.88 (m, 1H)	0.1	39.0	40.4	1.4
**22**	3.36 (t, 1H)	3.40 (m, 1H)	0.4	48.2	48.3	0.1
**NH**	9.26 (br s, 1H)	9.27 (t, 1H)	0.1	–	–	–

[a]No reference reported.

Note: NMR screenshots of isolated noelaquinone and isolated 3-ketoadociaquinone were not published.

X-Ray Crystallographic Data

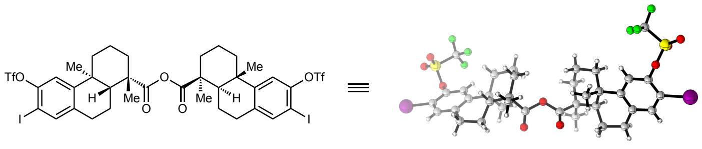

SI-02 (X-ray, CCDC = 2534780)

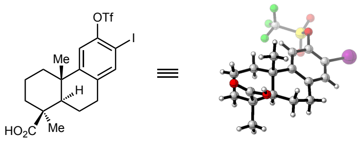

14 (X-ray, CCDC = 2534781)

Single crystals were mounted in Paratone-N oil on a MiTeGen MicroMount. X-ray diffraction data were collected at 150(2) K on an Oxford Xcalibur single crystal diffractometer using Mo Kα radiation.[36] Data sets were corrected for absorption using a multi-scan method, and structures were solved by direct methods (SHELXS)[37] or intrinsic phasing (SHELXT)[37] and refined by full-matrix least squares on F2 by SHELXL,[38] interfaced through the programs X-Seed (version 4)[39] and OLEX2.[40] In general, all non-hydrogen atoms were refined anisotropically and hydrogen atoms were included as invariants at geometrically estimated positions, unless specified otherwise. **Table S1** lists the X-ray experimental data and refinement parameters for the crystal structures. Compounds **SI-02** and **14** are obtained from a chiral starting material with the absolute stereochemistry set by this starting material and confirmed by anomalous dispersion effects in the data. Perspective views of the labelled asymmetric unit and the complete structure of **SI-02** is shown in **Figure S1**, while a perspective view of the asymmetric unit of **14** and one of the hydrogen bonded dimers is shown in **Figure S2**.

Full details of the structure determinations have been deposited with the Cambridge Crystallographic Data Centre as CCDC 2534780 and 2534781 (**SI-02** and **14**, respectively). Copies of this information may be obtained free of charge from The Director, CCDC, 12 Union Street, Cambridge CB2 1EZ, U.K. (fax, +44-1223-336-033; e-mail, deposit@ccdc.cam.ac.uk).

Table S1. X-ray experimental data for **SI-02** and **14**.

Compound	**SI-02**	**14**
CCDC number	2534780	2534781
cif	**CN-R-X1**	**CN-R-X2b**
Empirical formula	$C_{36}H_{38}F_6I_2O_9S_2$	$C_{18}H_{20}O_5F_3SI$
Formula weight	1046.58	532.30
Crystal system	monoclinic	monoclinic
Space group	*C*2	$P2_1$
a (Å)	22.9057(6)	12.1939(2)
b (Å)	7.2954(2)	13.4650(3)
c (Å)	12.0917(3)	12.6256(2)
a (°)	90	90
b (°)	104.598(3)	92.345(2)
g (°)	90	90
Volume ($Å^3$)	1955.37(9)	2071.27(7)
Z	2	4
Density (calc.) (Mg/m^3)	1.778	1.707
Absorption coefficient (mm^{-1})	1.796	1.699
F(000)	1036.0	1056.0
Crystal size (mm^3)	0.46 × 0.28 × 0.13	0.34 × 0.21 × 0.17
2q range for data collection (º)	6.594 to 58.558	6.688 to 58.818
Reflections collected	18826	36873
Observed reflections [*R*(*int*)]	4747 [R_{int} = 0.0453]	10035 [R_{int} = 0.0440]
Data/restraints/parameters	4747/1/251	10035/1/511
Goodness-of-fit on F2	1.046	1.041
R_1 [I>2s(I)]	0.0319	0.0345
wR_2 (all data)	0.0691	0.0733
Largest diff. peak and hole (e.$Å^{-3}$)	0.62/-0.43	0.81/-0.45
Flack parameter	-0.036(12)	-0.035(8)

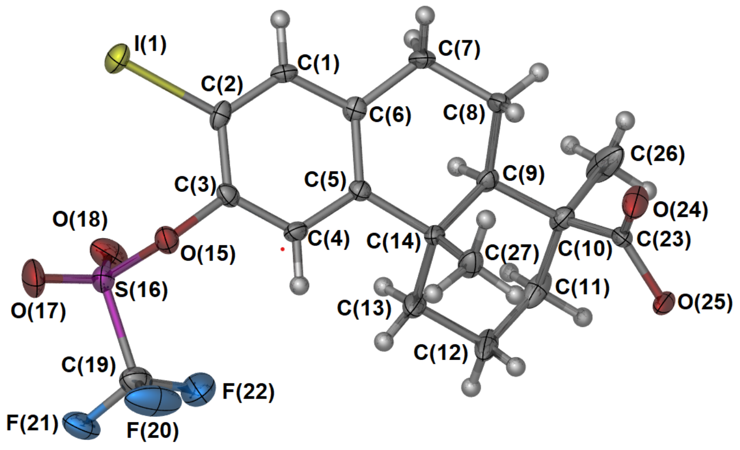

(a)

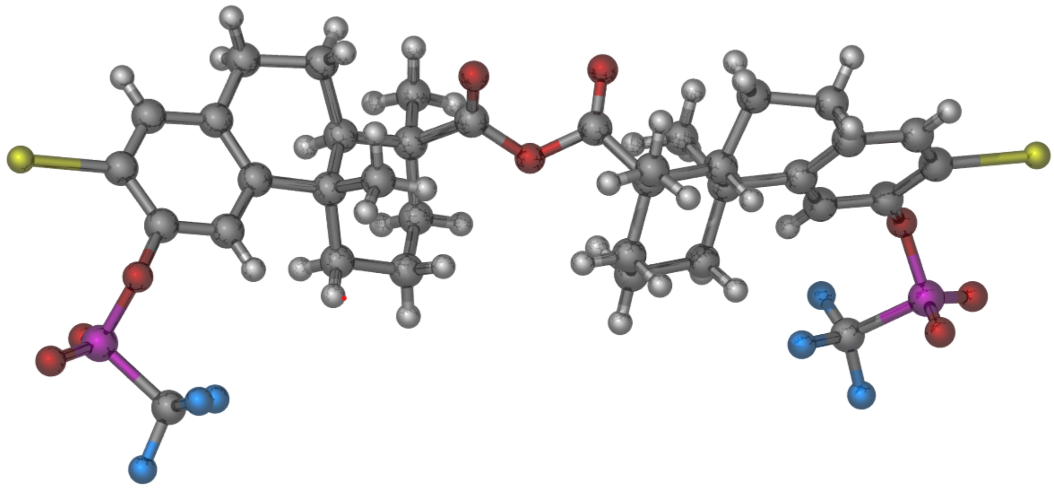

(b)

Figure S1. Perspective views of **(a)** the labelled asymmetric unit and **(b)** the complete structure of **SI-02** (C23-O24 = 1.182(6) Å; C23-O25 (anhydride O) 1.390(6) Å). Carbon – grey, hydrogen – white, oxygen – red, fluorine – blue, sulfur – pink, and iodine – yellow.

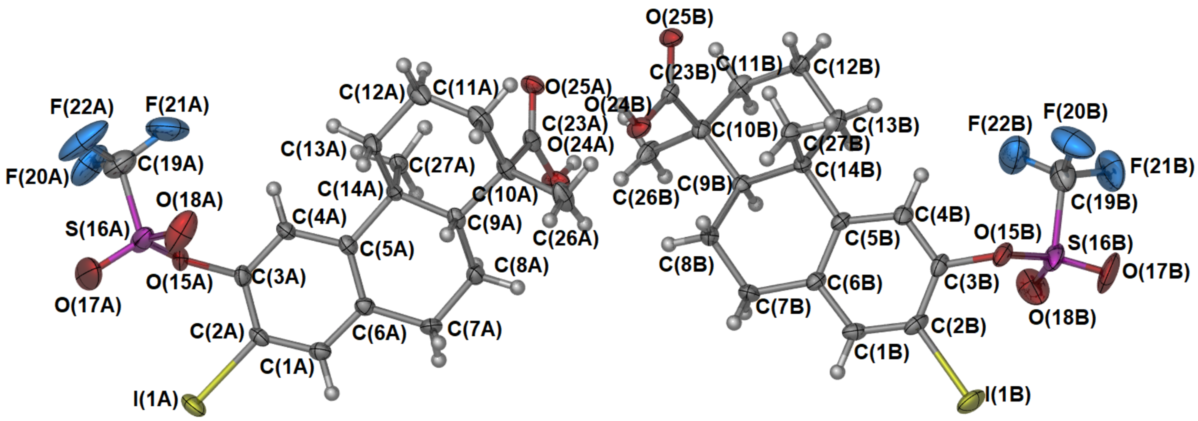

(a)

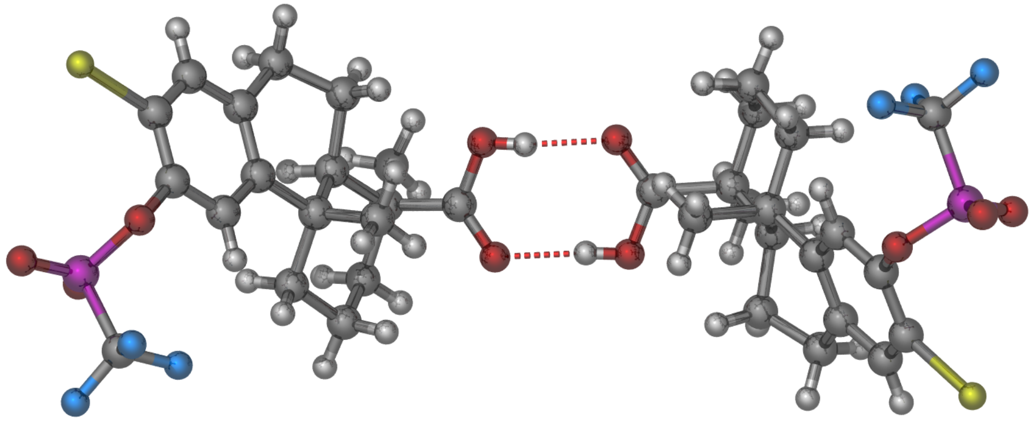

(b)

Figure S2. Perspective views of **(a)** the labelled asymmetric unit (Z' = 2) and **(b)** one of the solid-state hydrogen bonded dimers of **14**. $D_{O\text{-}H\cdots O}$ = 2.63 Å; angle $_{O\text{-}H\cdots O}$ = 164.9°. Carbon – grey, hydrogen – white, oxygen – red, fluorine – blue, sulfur – pink, and iodine – yellow.

Anticancer Assessment

Procedure

Cell growth inhibition was evaluated using the CellTiter-Glo® Luminescent Cell Viability Assay (Promega, Madison, WI, USA), which measures viable cells based on intracellular ATP levels. Briefly, human colon cancer cell lines (HCT116, SW620, RKO, and HT29) and normal human colon epithelial cells (CRL-1790) were seeded in 96-well plates at a density of 2×10^3 cells per well. After attachment, cells were treated with serial dilutions (0.125–128 μM; 0.125, 0.25, 0.5, 1, 2, 4, 8, 16, 32, 64, and 128 μM) of the following compounds:

- **(+)-2**: *ent*-cyclozonarone (CZ)
- quinone protected *ent*-cyclozonarone **17** (CZ_DME)
- **(+)-3**: *ent*-neopetrosiquinone A (NPQ_A)
- quinone protected *ent*-neopetrosiquinone A 1**8** (protected NPQ_A)
- **(−)-21**: *ent*-neopetrosiquinone B (NPQ_B)
- quinone protected *ent*-neopetrosiquinone B **20** (protected NPQ_B)
- **(−)-5**: *ent*-orhalquinone (OQ)
- quinone protected *ent*-orhalquinone **25** (OQ_DME)
- **(−)-7**: *ent*-xestoquinone (XQ)
- quinone protected *ent*-xestoquinone **26** (XQ_DME)
- **(−)-30/31**: *ent*-xestoquinolides B/C (XQL B/C)
- **(−)-1**: *ent*-halenaquinone (HQ)
- quinone protected *ent*-halenaquinone **33** (HQ_DME)
- **(+)-13**: podocarpic acid (PA)
- **(+)-34**: wortmannin
- 5-Fluorouracil (5-FU)

Cells were incubated with compounds for 72 hours. At the end of treatment, luminescence was measured according to the manufacturer's instructions. Relative cell viability was calculated, dose–response curves were generated, and IC_{50} values were determined using GraphPad Prism 10 (GraphPad Software, CA, USA).

Summary

IC_{50} values of halenaquinone family members and related natural products. IC_{50} values are presented with their 95% confidence intervals in parentheses.

<1 µM	1 – 5 µM	5 – 50 µM	>50 µM	

Compound	HCT116 (µM)	SW620 (µM)	RKO (µM)	HT-29 (µM)	CRL-1790 (µM)
**(+)-2**: *ent*-cyclozonarone	0.49 (0.46–0.53)	0.02 (0.016–0.038)	0.38 (0.34–0.41)	1.15 (1.01–1.30)	0.064 (0.04–0.10)
**(+)-3**: *ent*-neopetrosiquinone A	15.59 (13.61–17.86)	6.14 (5.72–6.60)	49.09 (44.51–54.15)	77.52 (63.58–94.51)	77.26 (64.28–92.88)
**(−)-21**: *ent*-neopetrosiquinone B	1.52 (1.46–1.58)	0.74 (0.69–0.78)	1.16 (1.09–1.22)	1.31 (1.22–1.41)	0.55 (0.47–0.65)
**(−)-5**: *ent*-orhalquinone	0.58 (0.54–0.63)	0.39 (0.36–0.42)	0.58 (0.55–0.62)	1.57 (1.32–1.87)	0.062 (0.05–0.08)
**(−)-7**: *ent*-xestoquinone	0.56 (0.53–0.59)	0.33 (0.31–0.37)	0.49 (0.44–0.55)	0.15 (0.13–0.18)	1.63 (1.52–1.75)
**(−)-30/31**: *ent*-xestoquinolides B/C	24.16 (22.95–25.43)	17.91 (15.54–20.64)	16.90 (15.29–18.68)	12.75 (10.82–15.03)	40.70 (35.56 to 46.58)
**(−)-1**: *ent*-halenaquinone	148.00 (119.0–184.1)	40.12 (34.71–46.39)	59.33 (54.18–64.98)	166.70 (112.1–248.0)	119.4 (91.05–156.5)
**17**: protected *ent*-cyclozonarone	30.95 (28.21–33.95)	10.23 (9.680–10.80)	16.44 (15.33–17.62)	9.24 (8.55–9.98)	–
**18**: protected *ent*-neopetrosiquinone A	ND (but >50 µM)	ND (but >50 µM)	51.98 (45.91–58.86)	75.05 (61.15–92.12)	–
**20**: protected *ent*-neopetrosiquinone B	82.54 (72.40–94.10)	32.99 (29.74–36.60)	53.27 (46.99–60.38)	10.95 (7.847–15.29)	–
**25**: protected *ent*-orhalquinone	12.42 (10.90–14.15)	10.83 (9.627–12.18)	11.46 (10.28–12.78)	10.59 (9.78–11.47)	–
**26**: protected *ent*-xestoquinone	15.42 (13.09–18.17)	32.27 (26.83–38.83)	7.10 (6.03–8.36)	6.34 (5.89–6.83)	–
**33**: protected *ent*- halenaquinone	2.90 (2.74–3.06)	2.67 (2.52–2.83)	2.66 (2.53–2.79)	2.73 (2.64–2.84)	–
**(+)-13**: podocarpic acid	202.30 (147.4–277.7)	214.50 (57.89–794.6)	ND (but >50 µM)	158.1 (123.5–202.3)	234.7 (181.4–303.7)
**(+)-34**: wortmannin	12.75 (11.88–13.68)	22.73 (18.71–27.62)	171.20 (136.0–215.4)	34.90 (29.54–41.23)	59.08 (45.15–77.30)
5-fluorouracil	12.88 (10.87–15.25)	134.30 (83.39–216.4)	6.11 (5.63–6.63)	14.62 (11.46–18.65)	–

ND = not determined due to ambiguous nonlinear fit

Response Curves

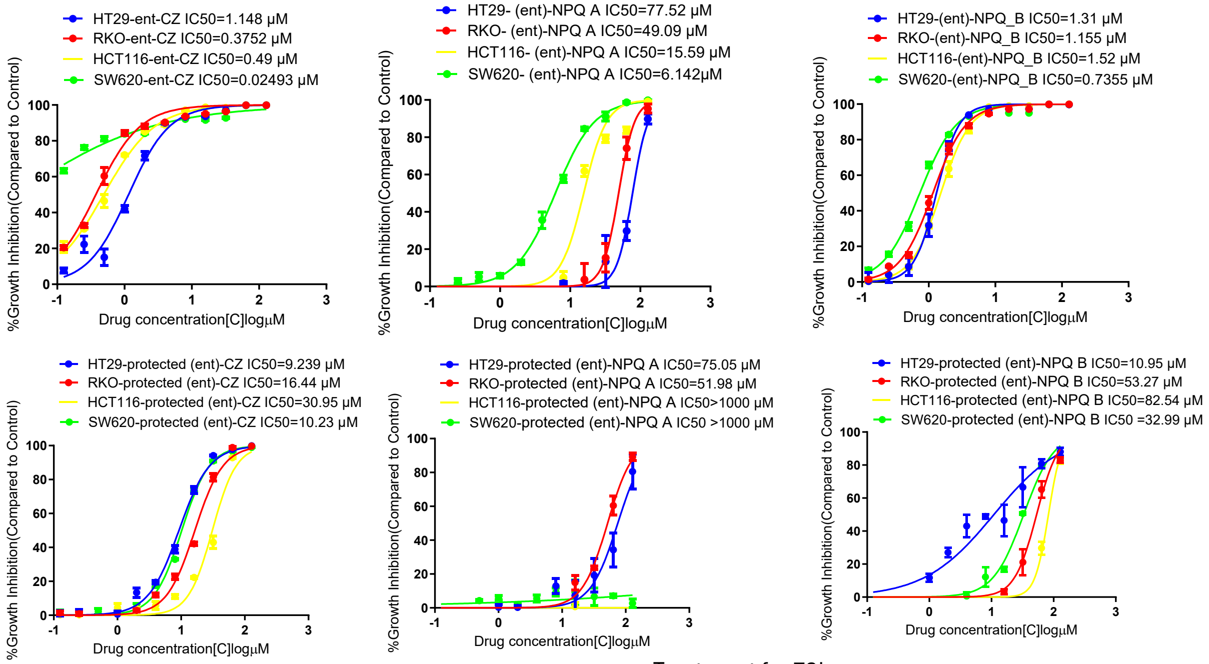

Treatment for 72h

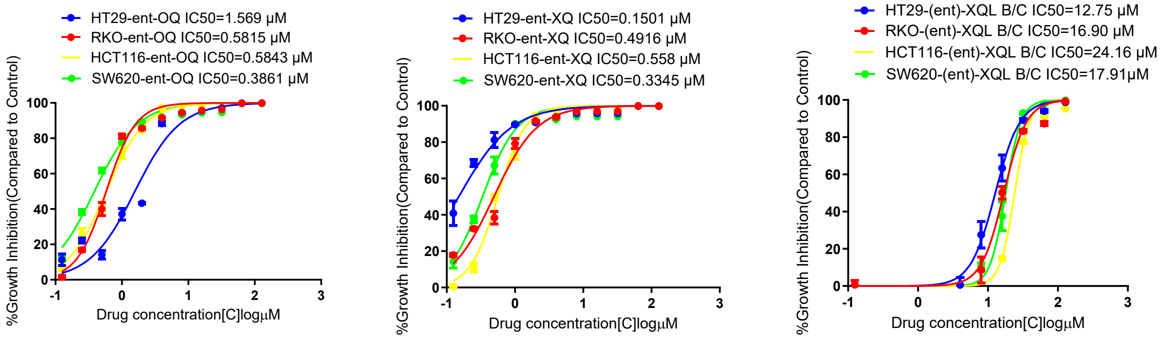

Treatment for 72h

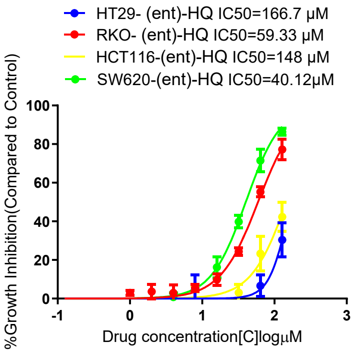

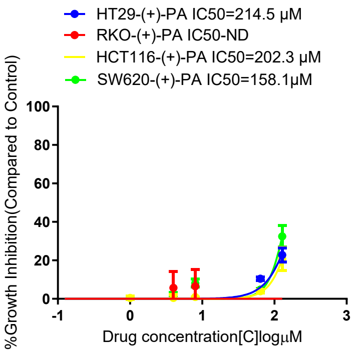

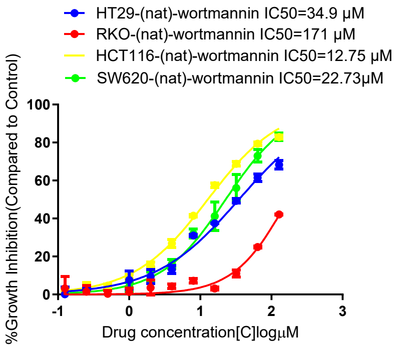

Treatment for 72h

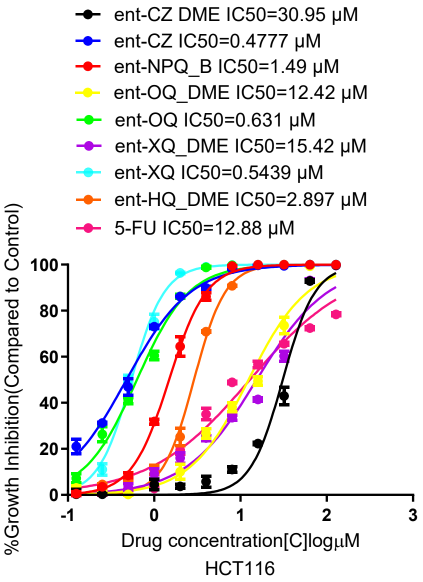

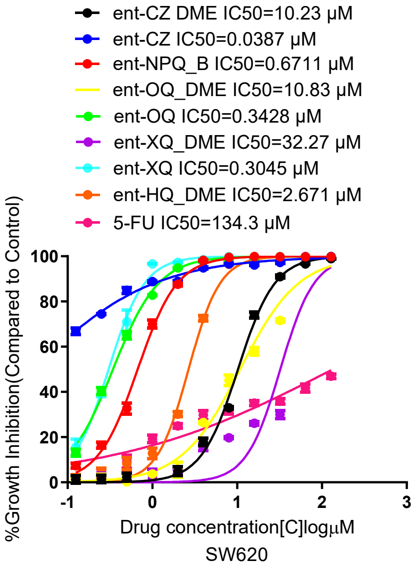

Treatment for 72h

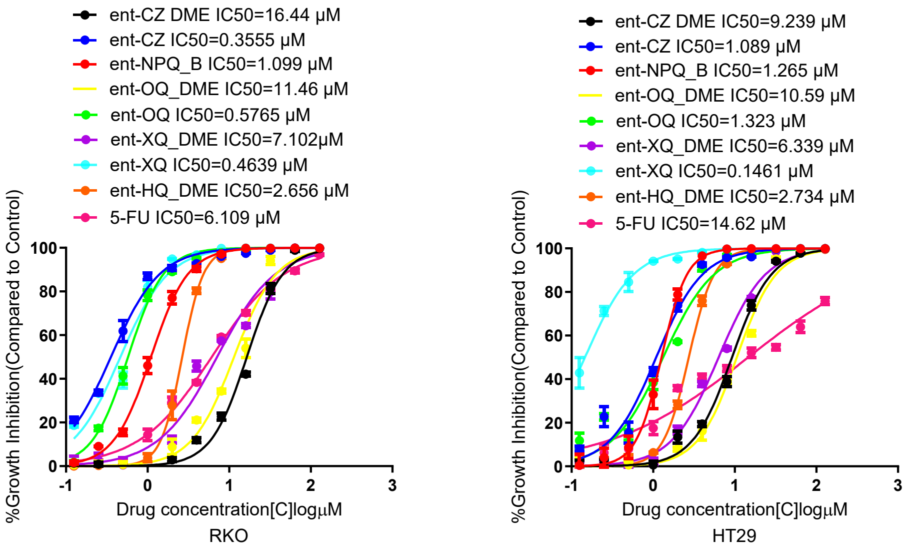

Treatment for 72h

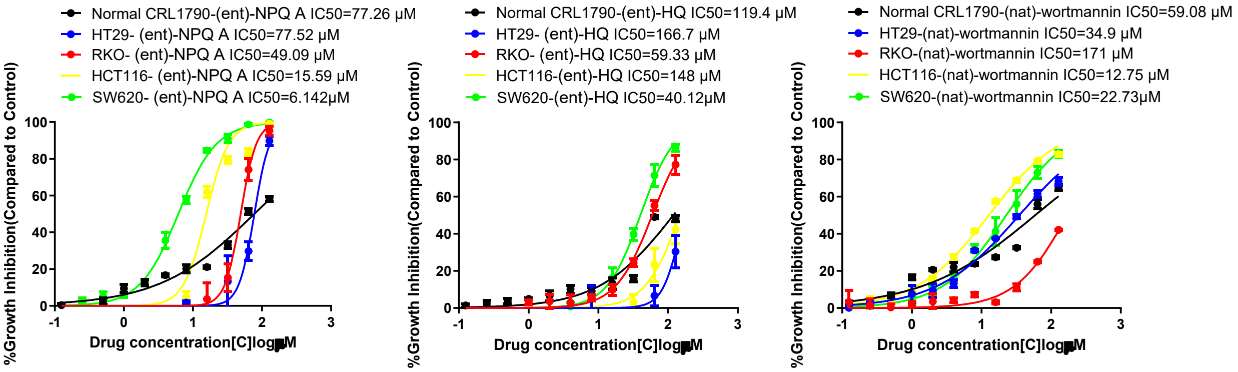

Treatment for 72 h

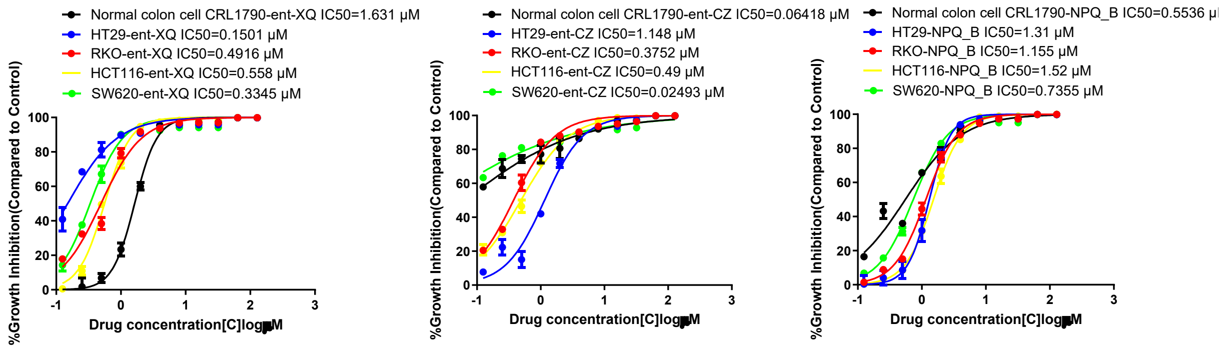

Treatment for 72 h

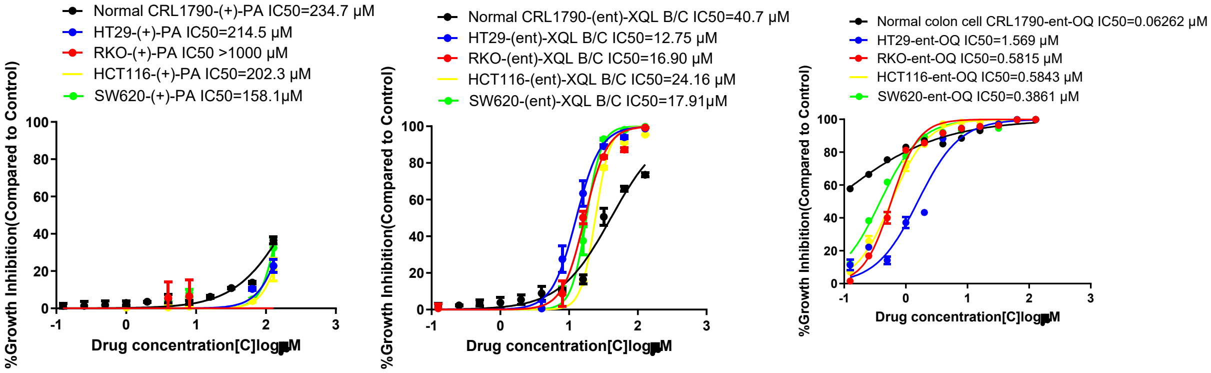

Treatment for 72 h

NMR Spectra

Isolation and Characterization of Podocarpic Acid

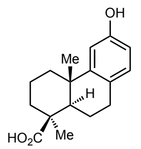

(+)-13: podocarpic acid
^{1}H NMR
$(CD_3)_2CO$, 600 MHz

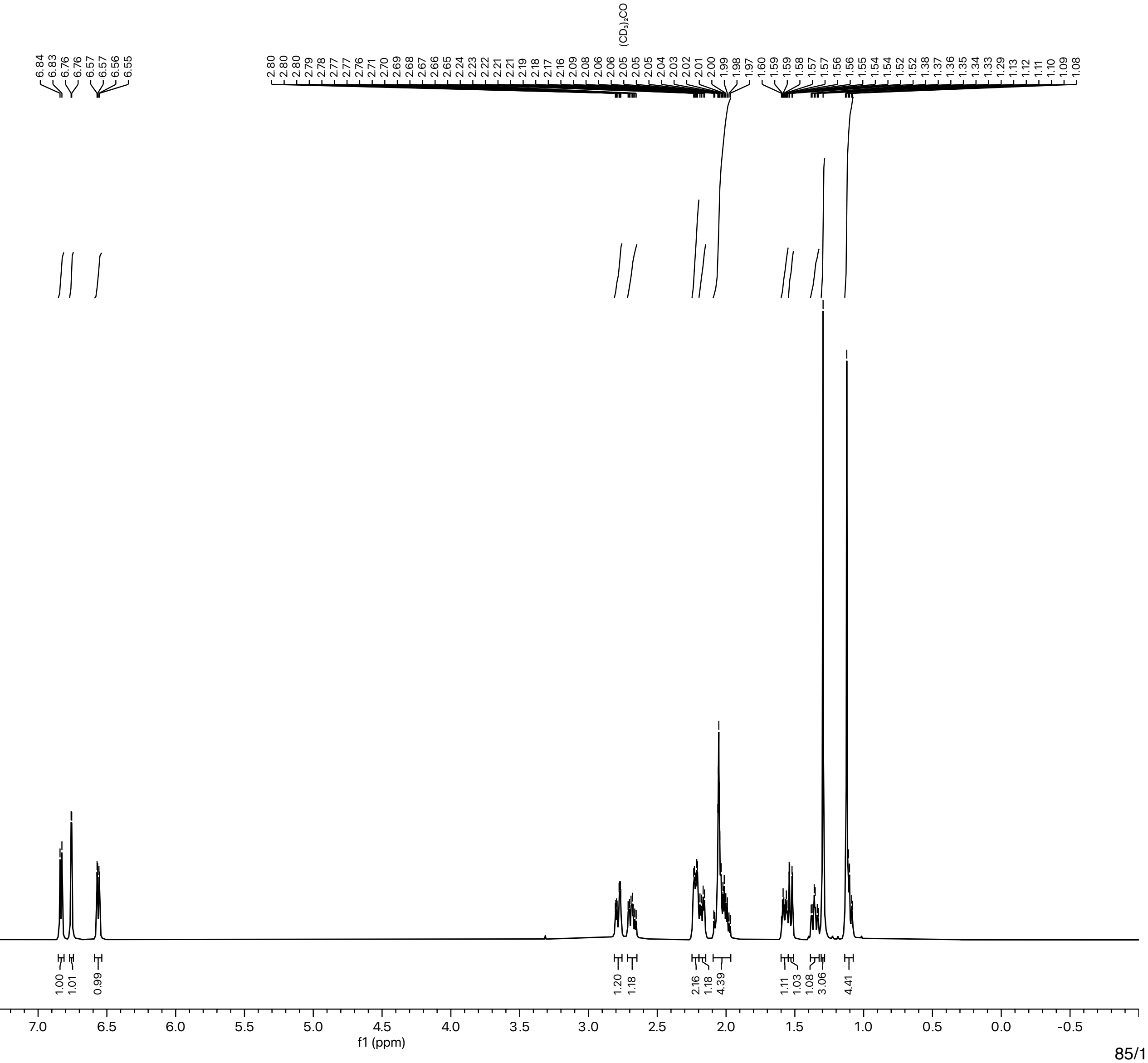

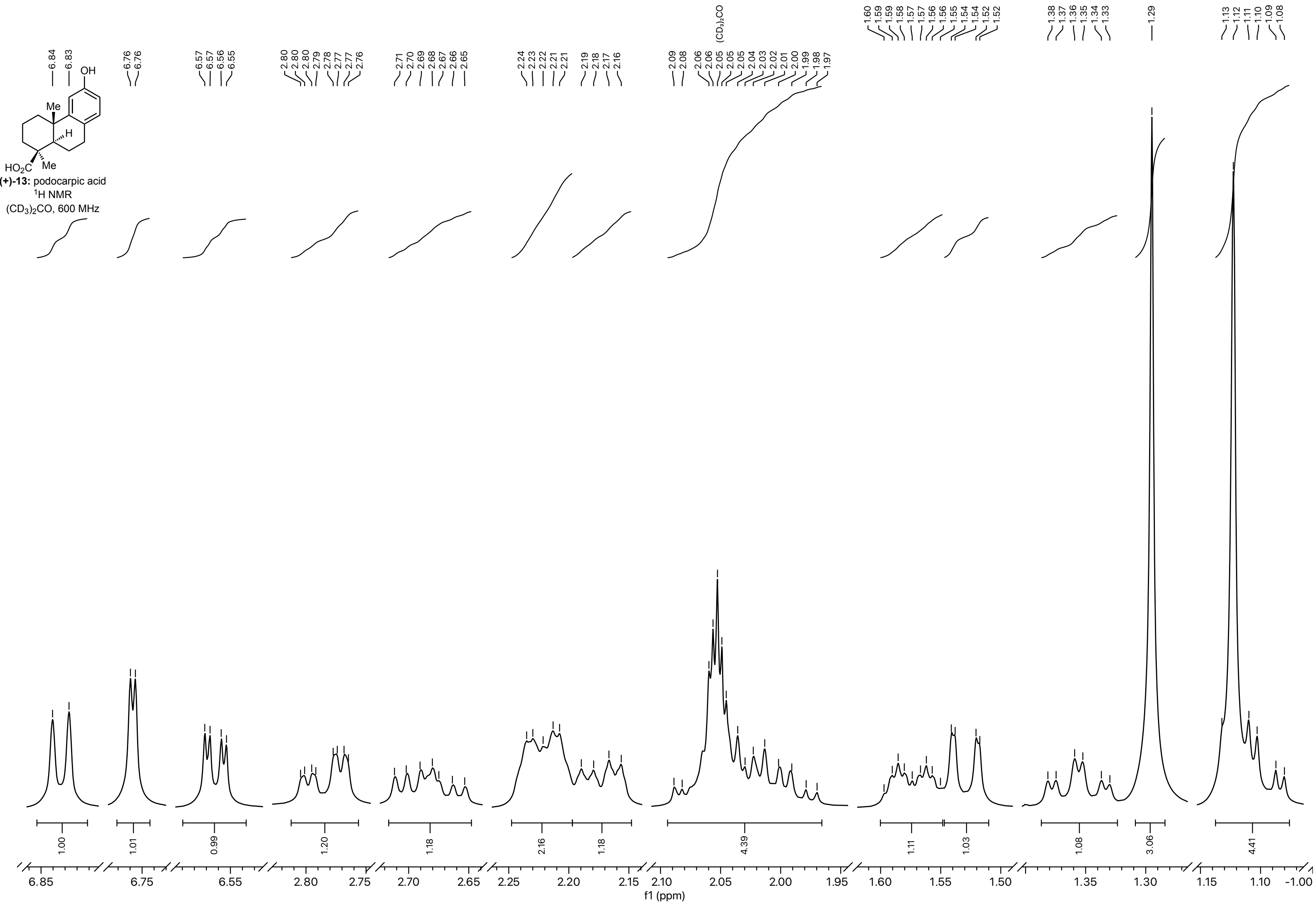

(+)-13: podocarpic acid
^1H NMR
$(CD_3)_2CO$, 600 MHz

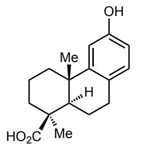

(+)-13: podocarpic acid
^1H NMR
CD_3OD, 500 MHz

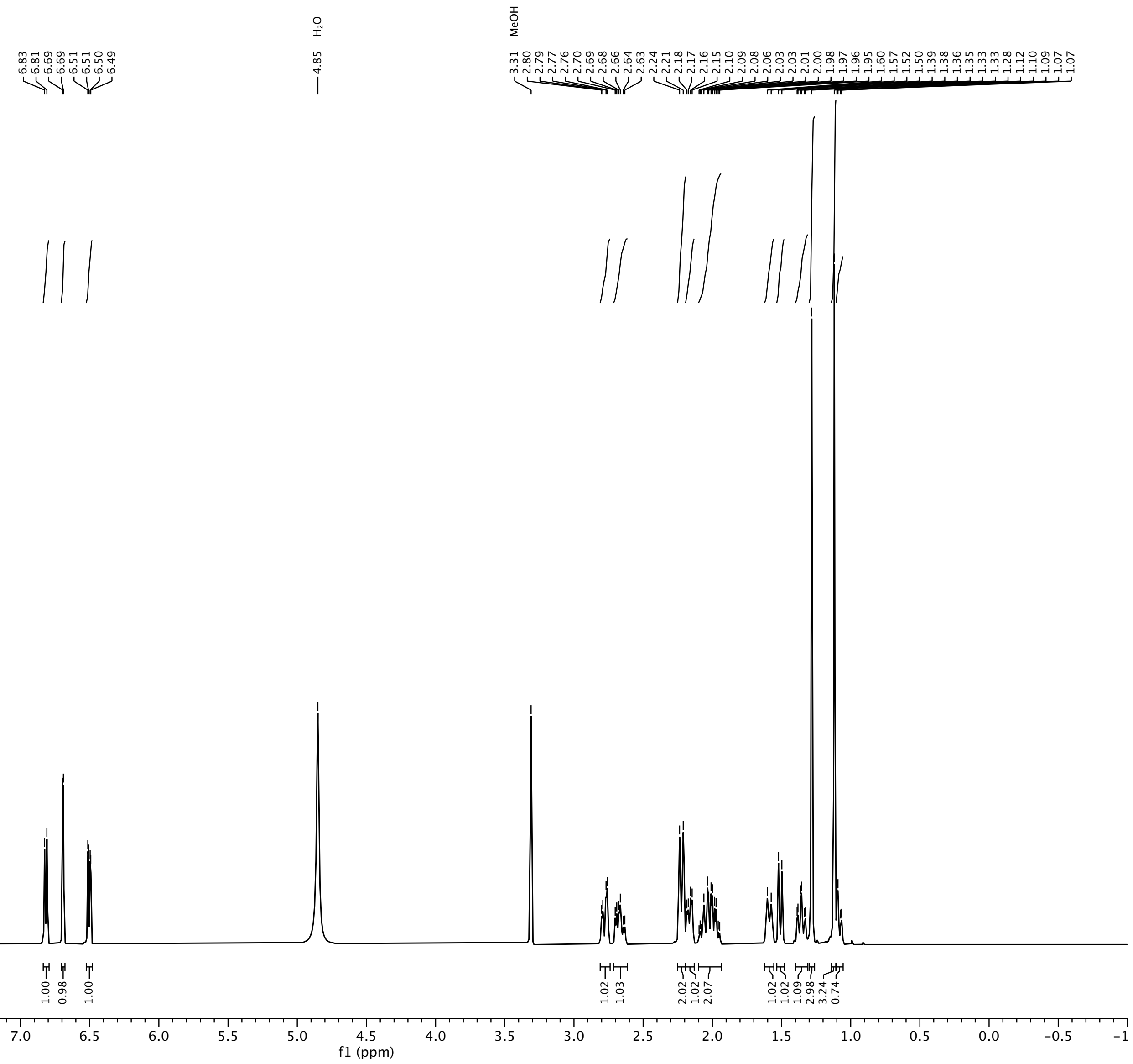

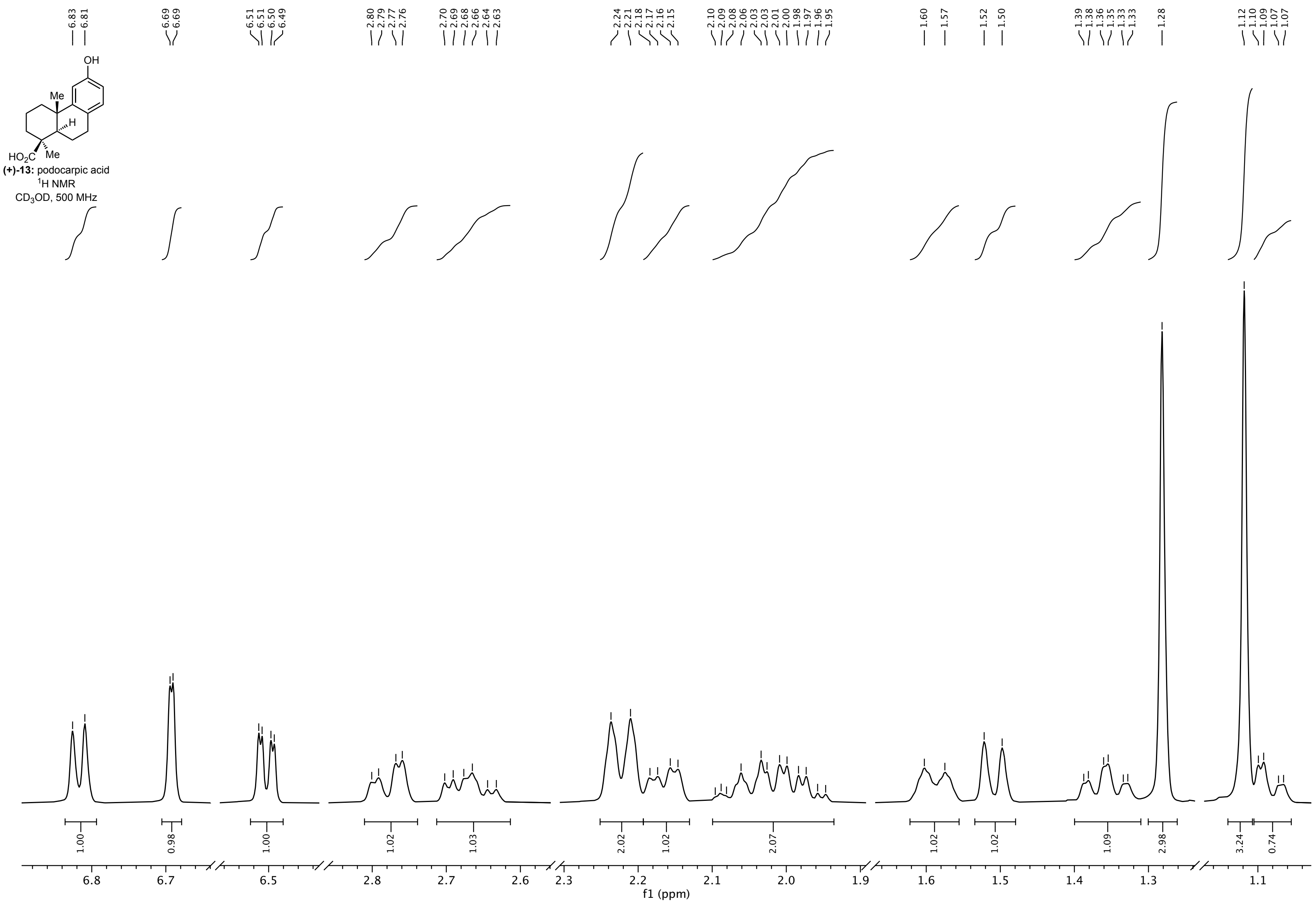
OH
Me
H
HO2C
Me
(+)-13: podocarpic acid
1H NMR
CD3OD, 500 MHz
f1 (ppm)

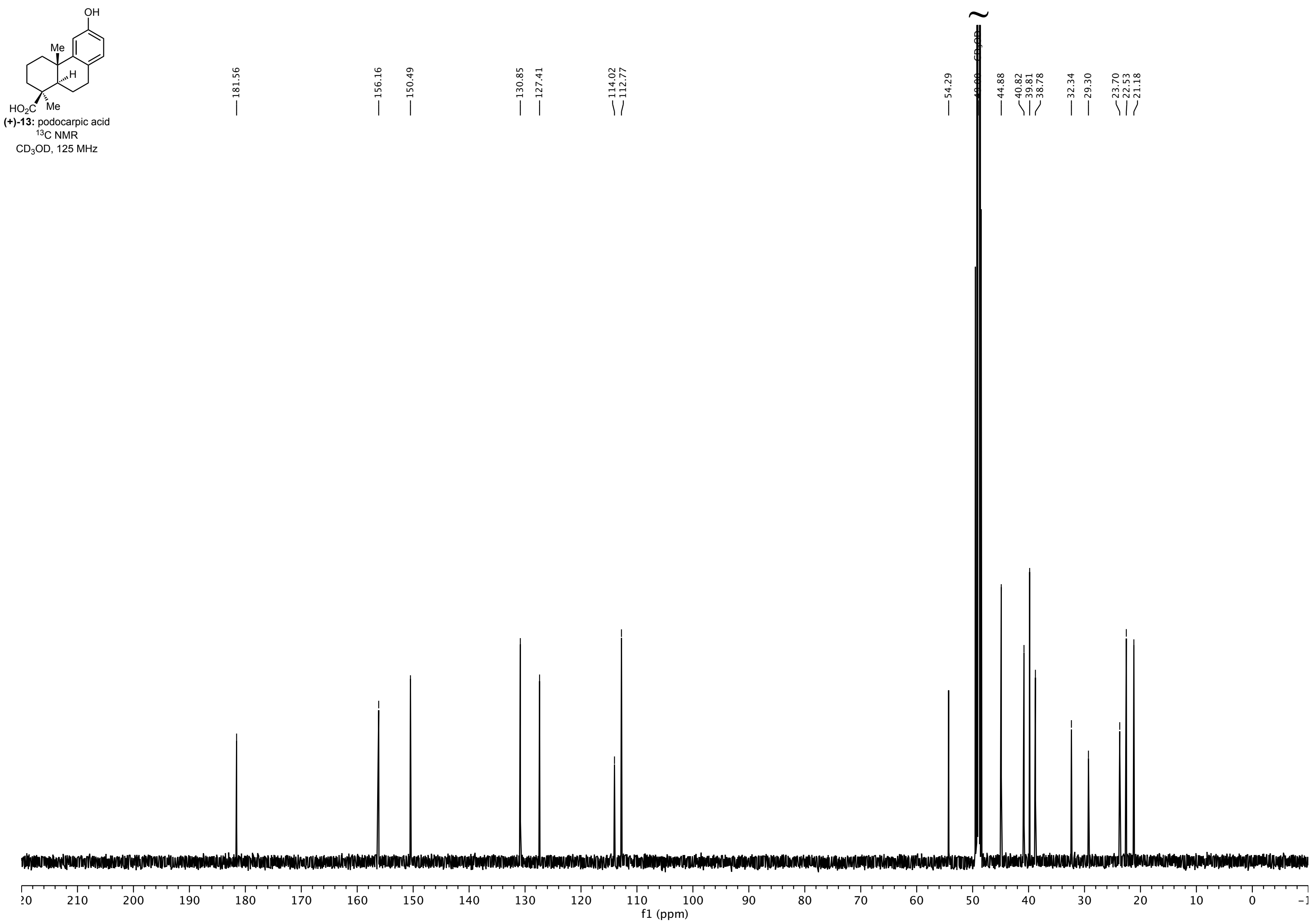
OH
Me
H
HO_2C
Me
(+)-13: podocarpic acid
^{13}C NMR
CD_3OD, 125 MHz
181.56
156.16
150.49
130.85
127.41
114.02
112.77
54.29
44.88
40.82
39.81
38.78
32.34
29.30
23.70
22.53
21.18
210
200
190
180
170
160
150
140
130
120
110
100
90
80
70
60
50
40
30
20
10
0
f1 (ppm)

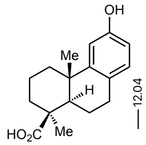

(+)-13: podocarpic acid
^{1}H NMR
$(CD_3)_2SO$, 500 MHz

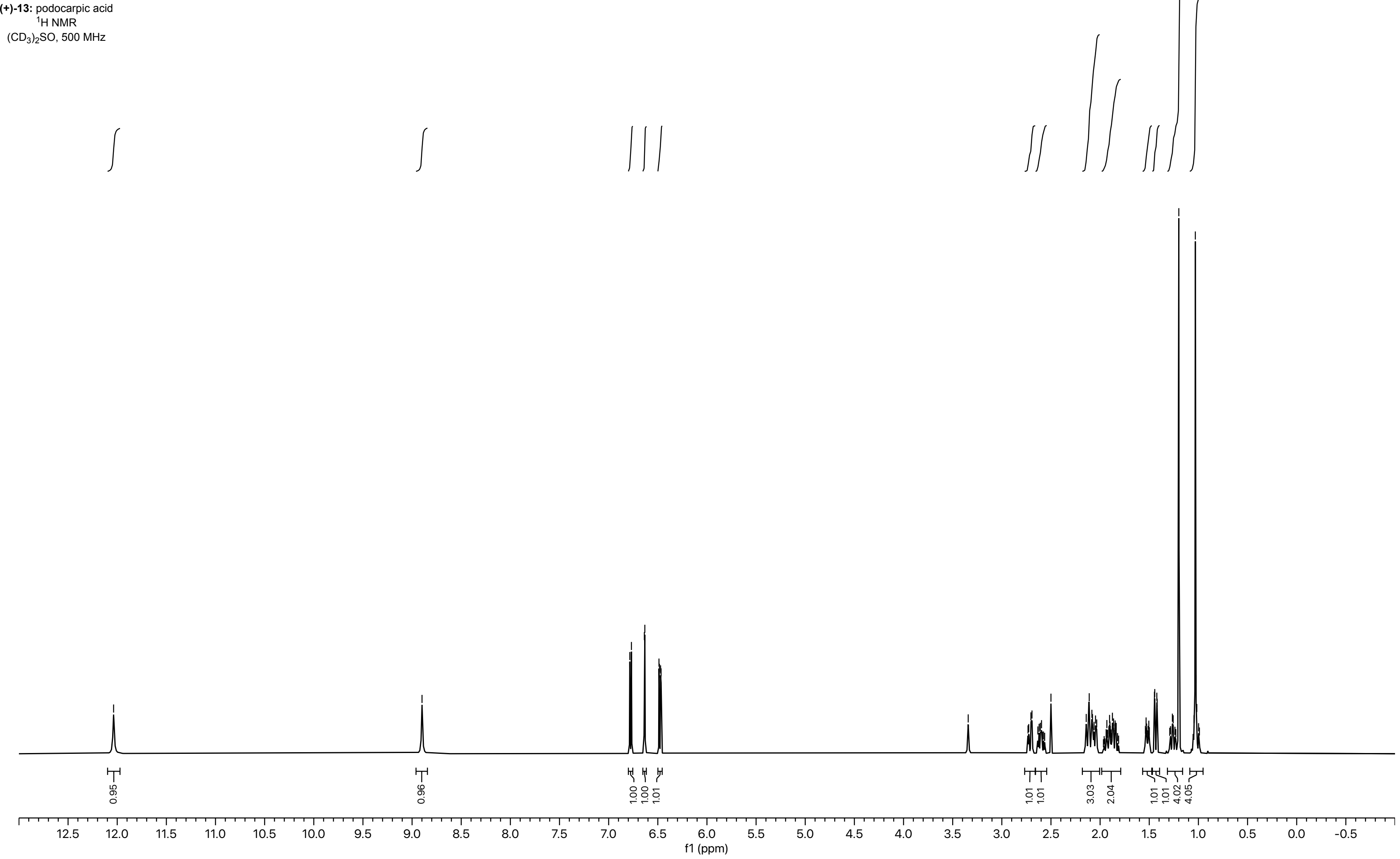

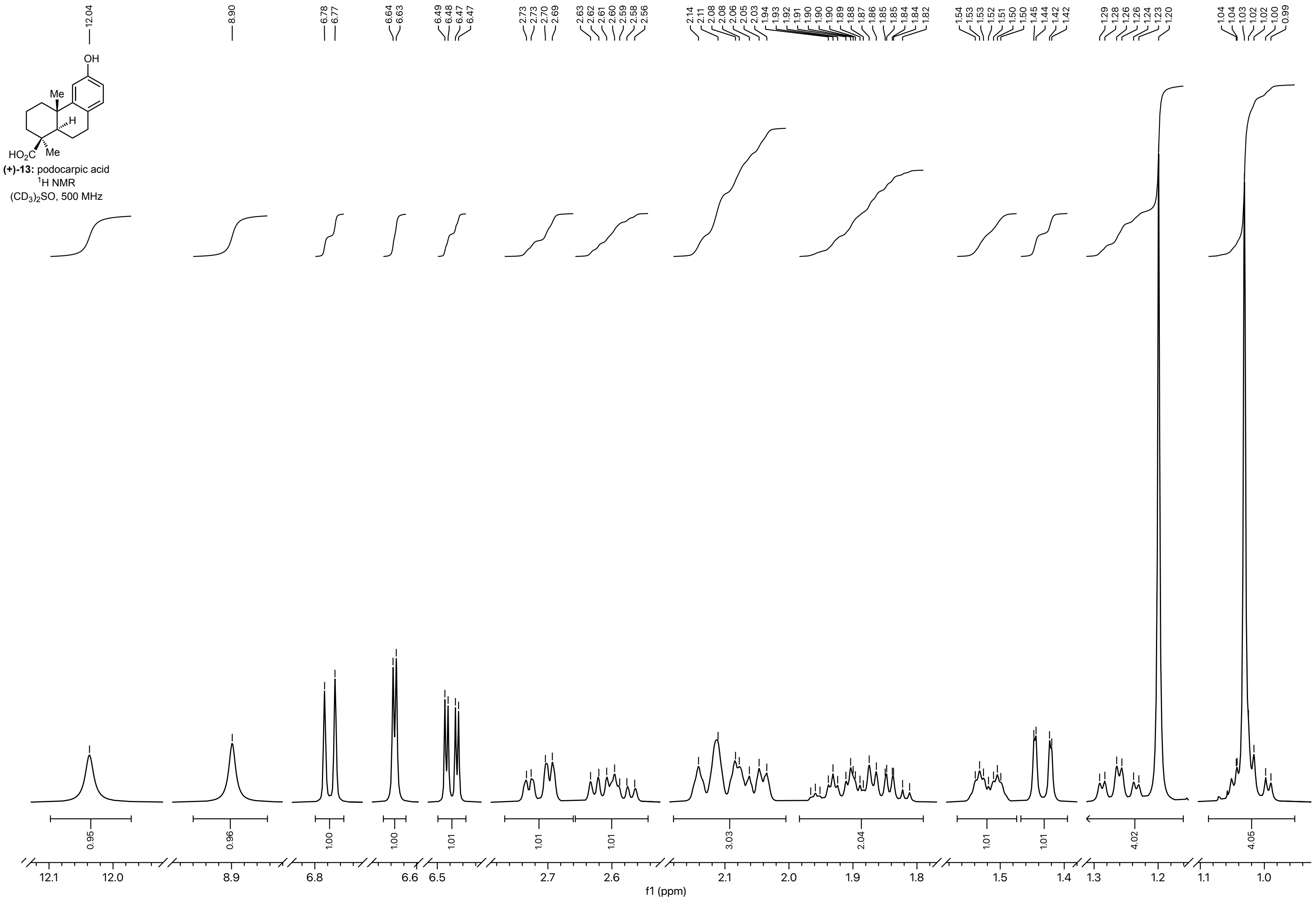
OH
Me
H
HO2C Me
(+)-13: podocarpic acid
1H NMR
(CD3)2SO, 500 MHz
f1 (ppm)

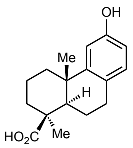

(+)-13: podocarpic acid
^{13}C NMR
$(CD_3)_2SO$, 125 MHz

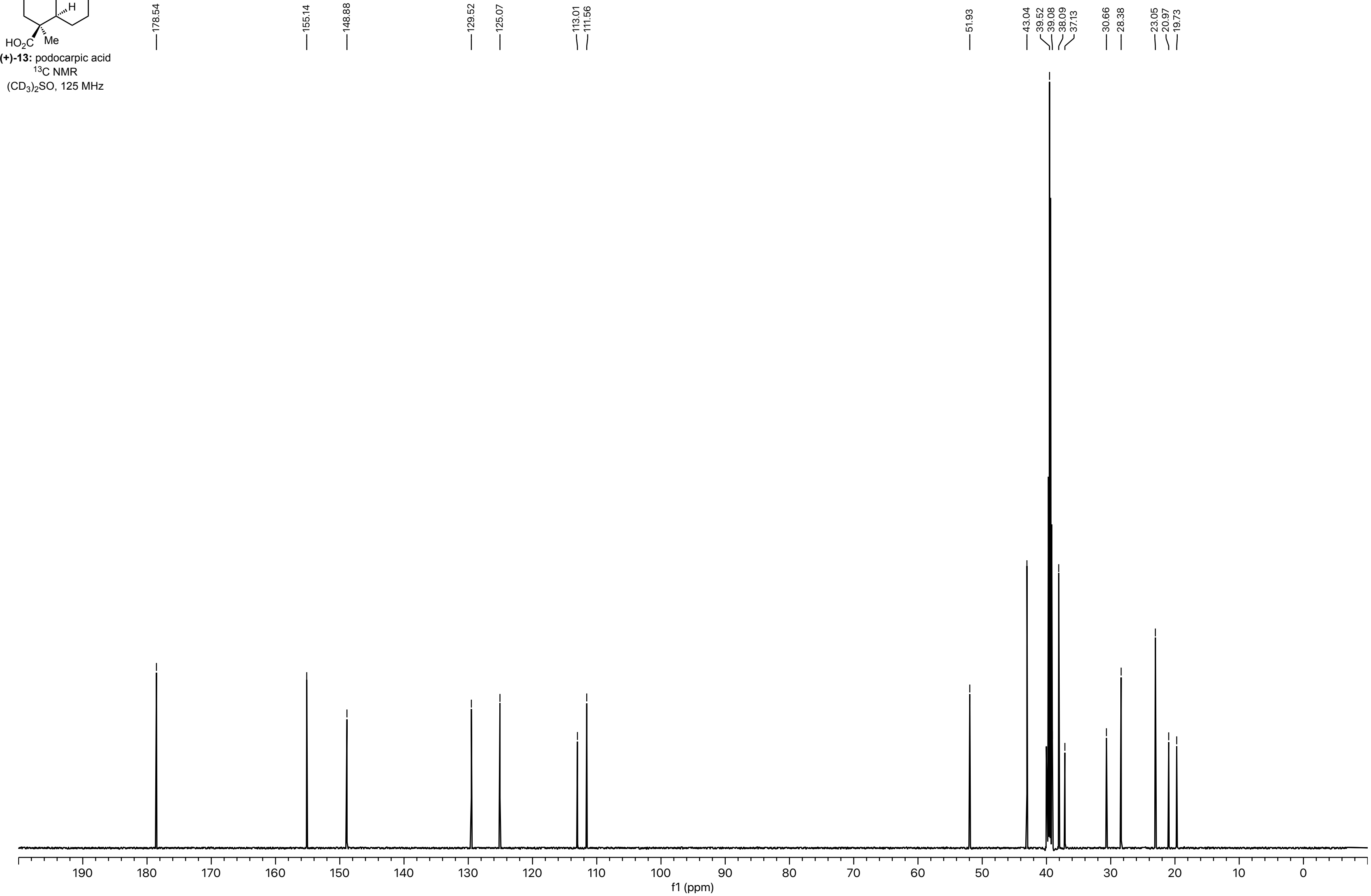

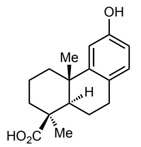

(+)-13: podocarpic acid
COSY
$(CD_3)_2SO$, 500 MHz

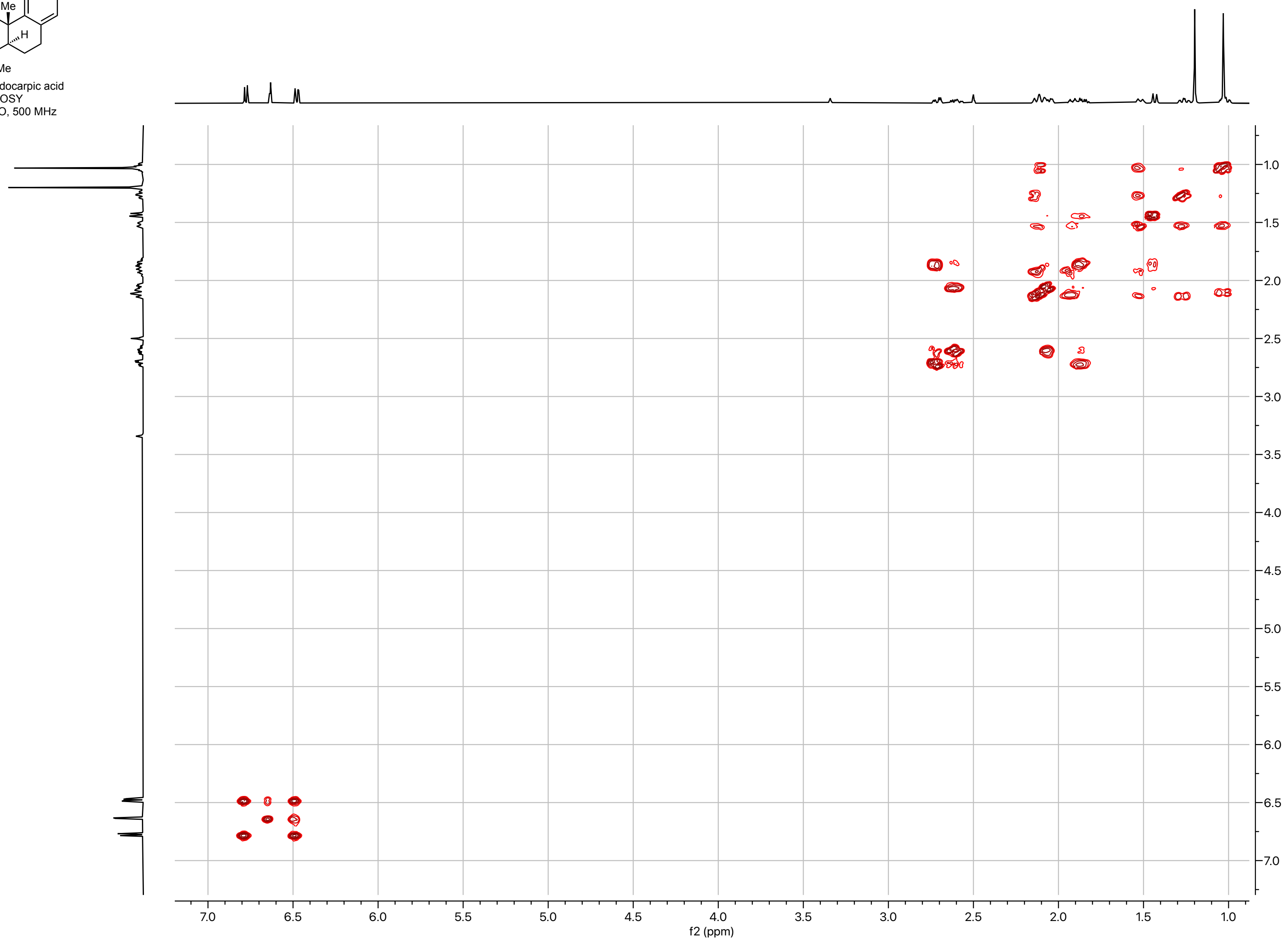

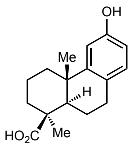

(+)-13: podocarpic acid
HSQC
$(CD_3)_2SO$, 500 MHz

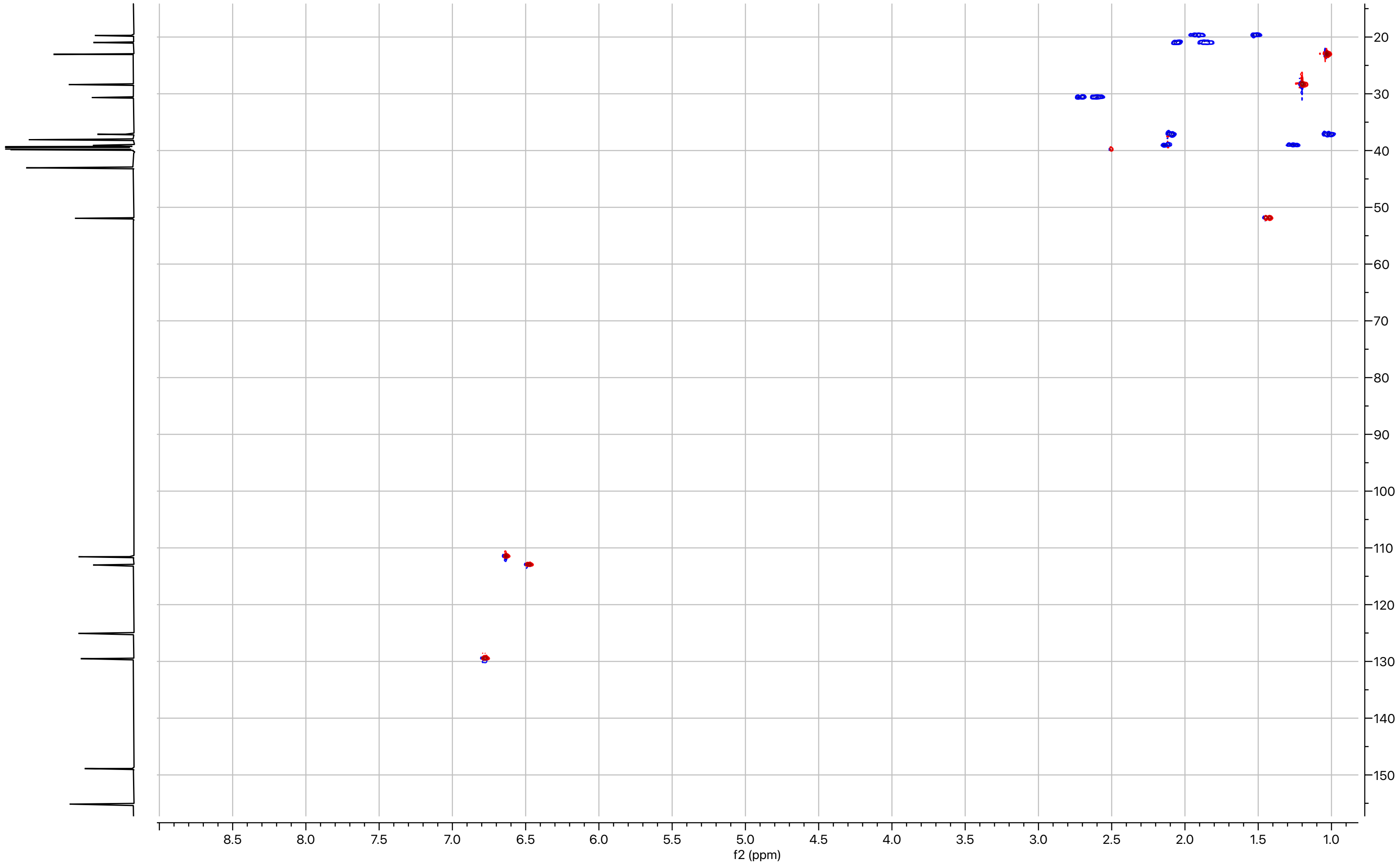

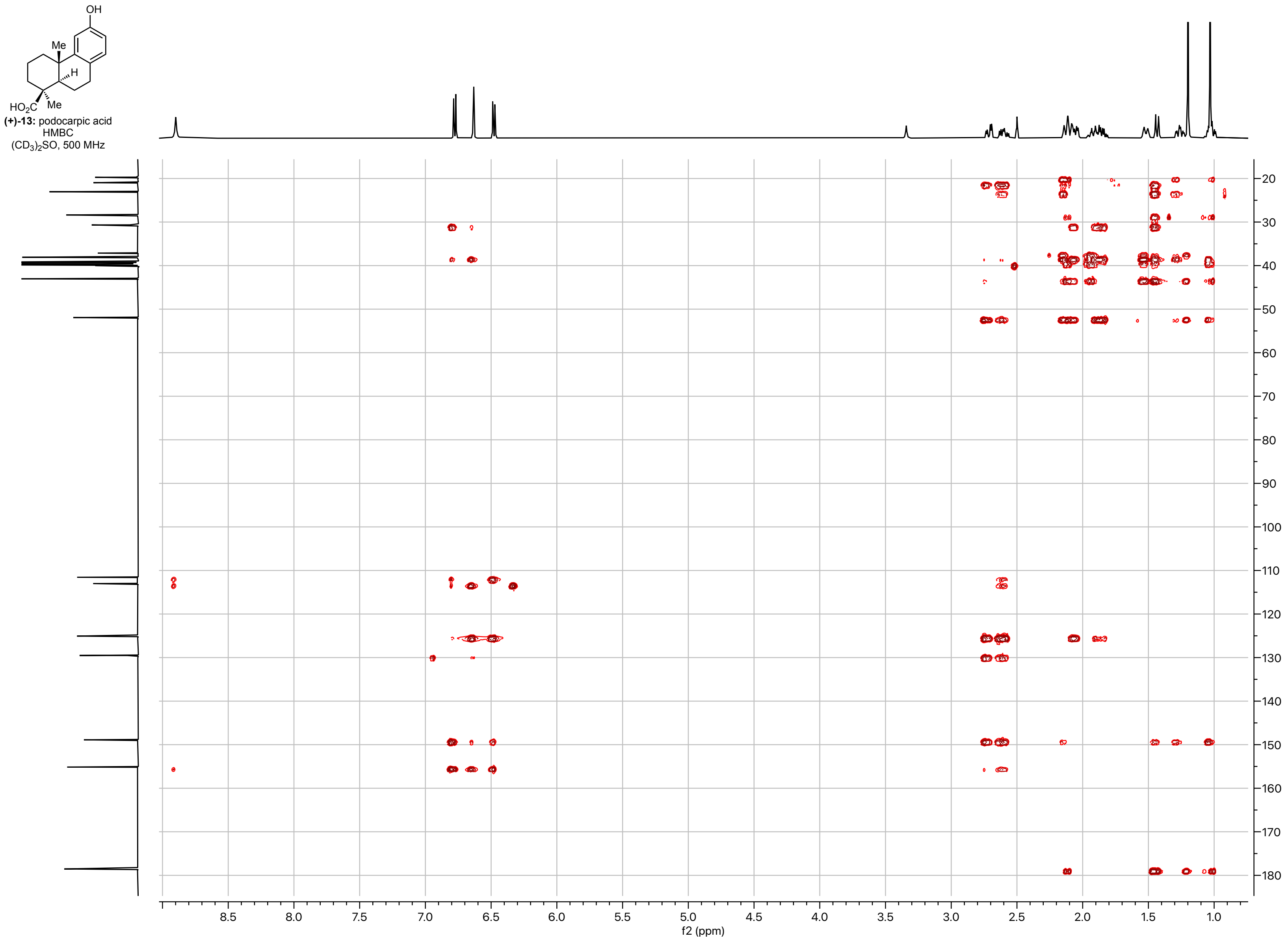

(+)-13: podocarpic acid
HMBC
$(CD_3)_2SO$, 500 MHz

Part 1: A Diels–Alder Disconnection

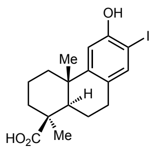

SI-01
^{1}H NMR
CD_3OD, 500 MHz

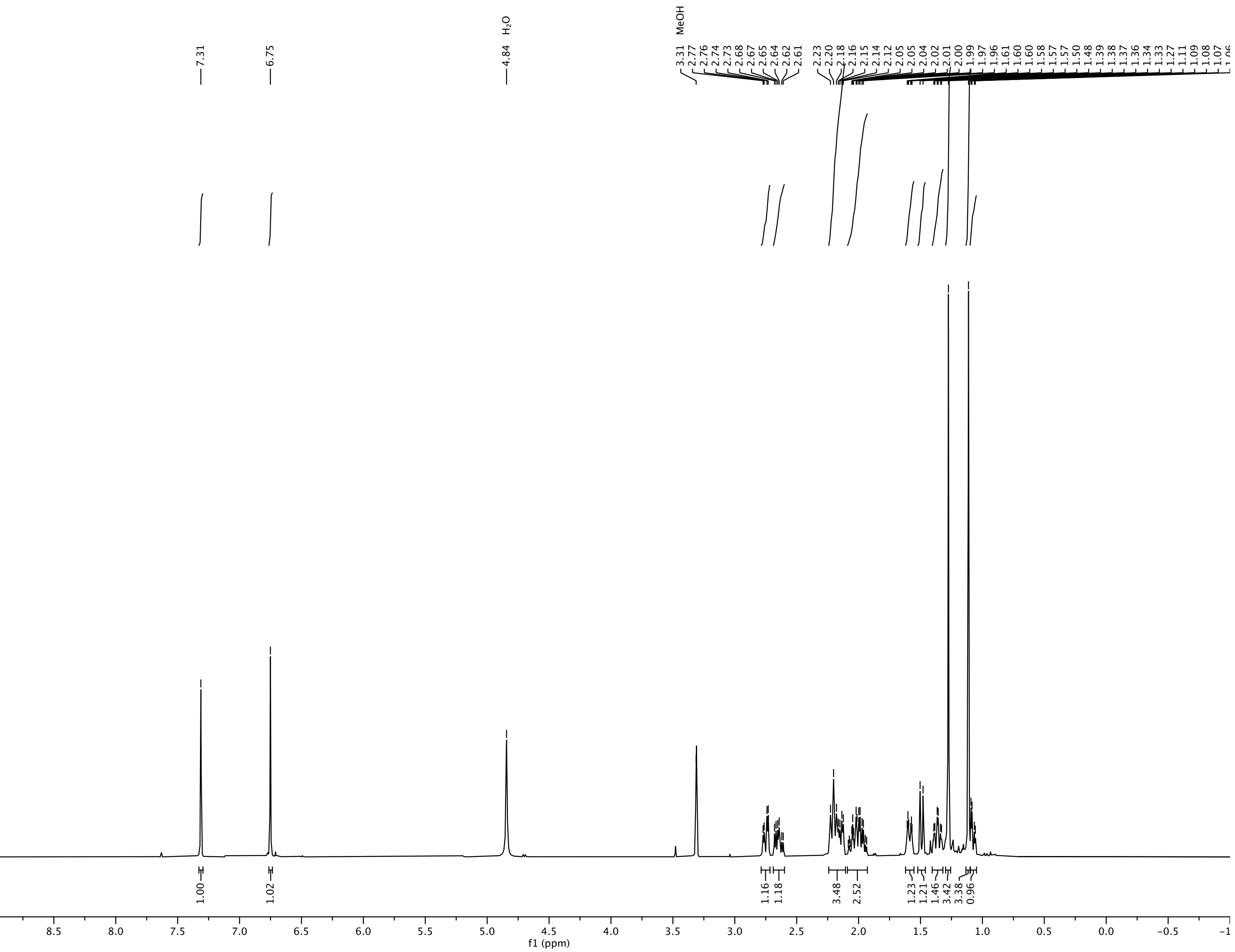

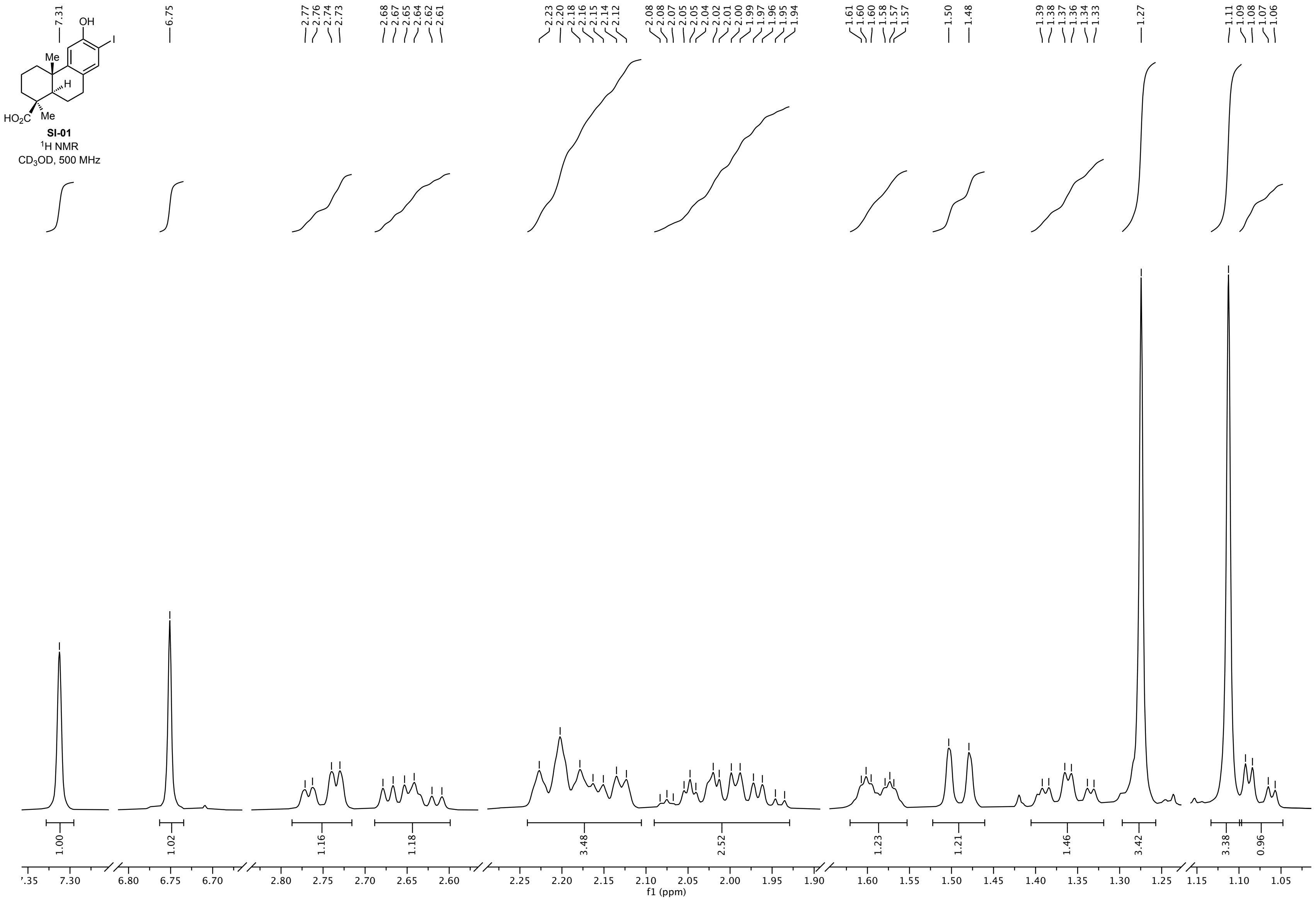

SI-01
^{1}H NMR
CD_3OD, 500 MHz

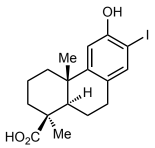

SI-01

^{13}C NMR

CD_3OD, 125 MHz

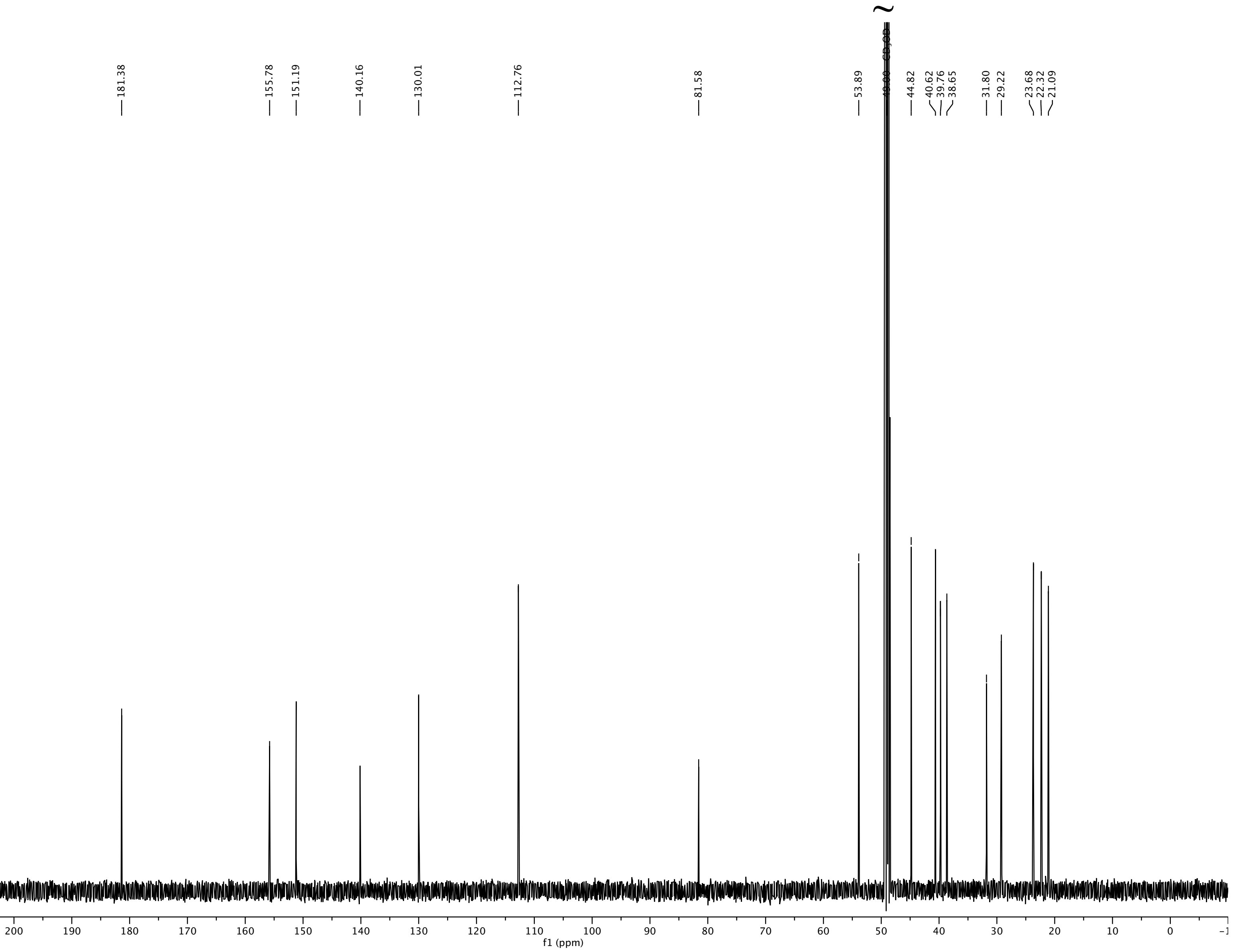

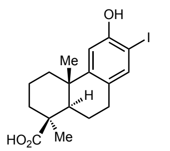

SI-01
^{1}H NMR
$CDCl_3$, 500 MHz

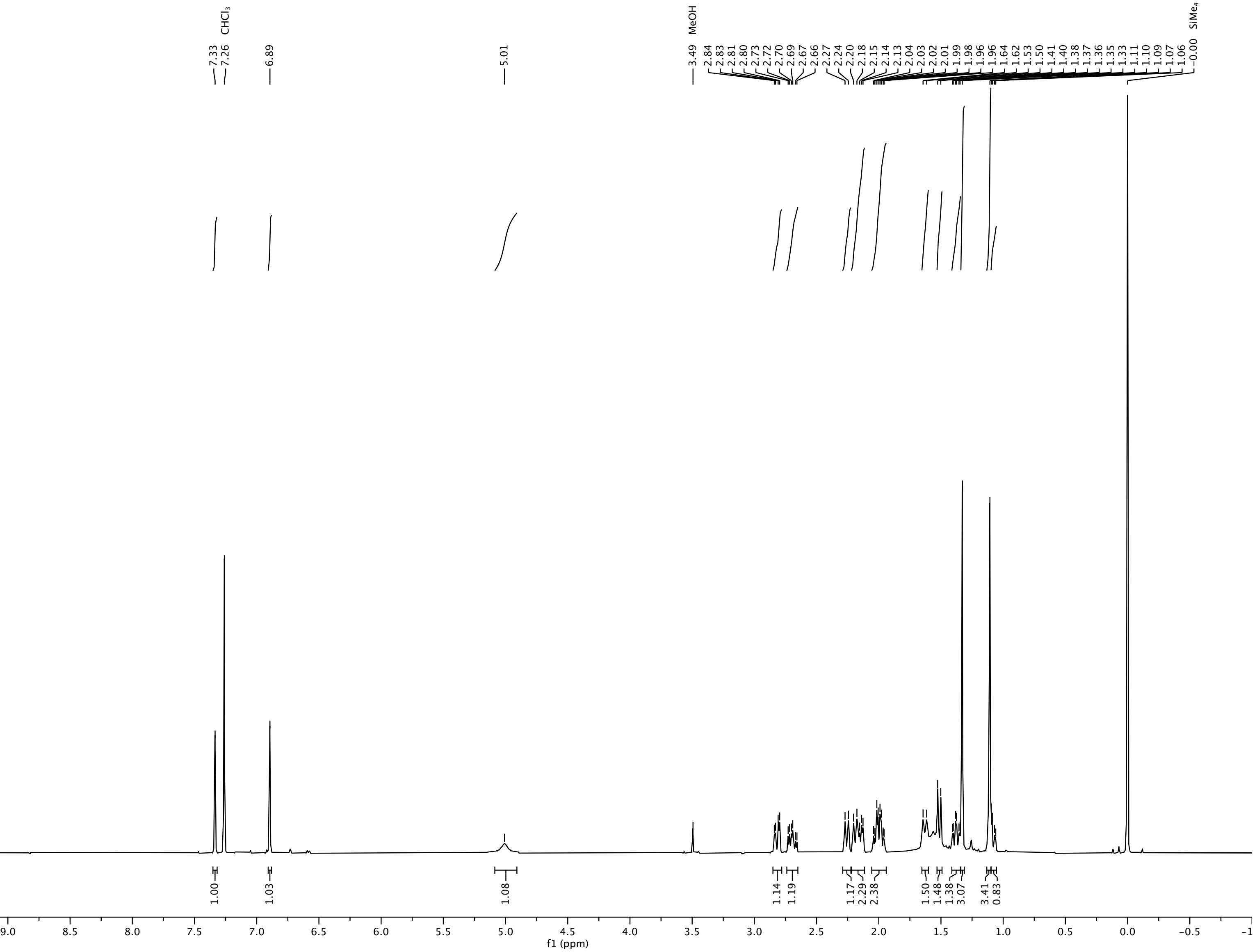

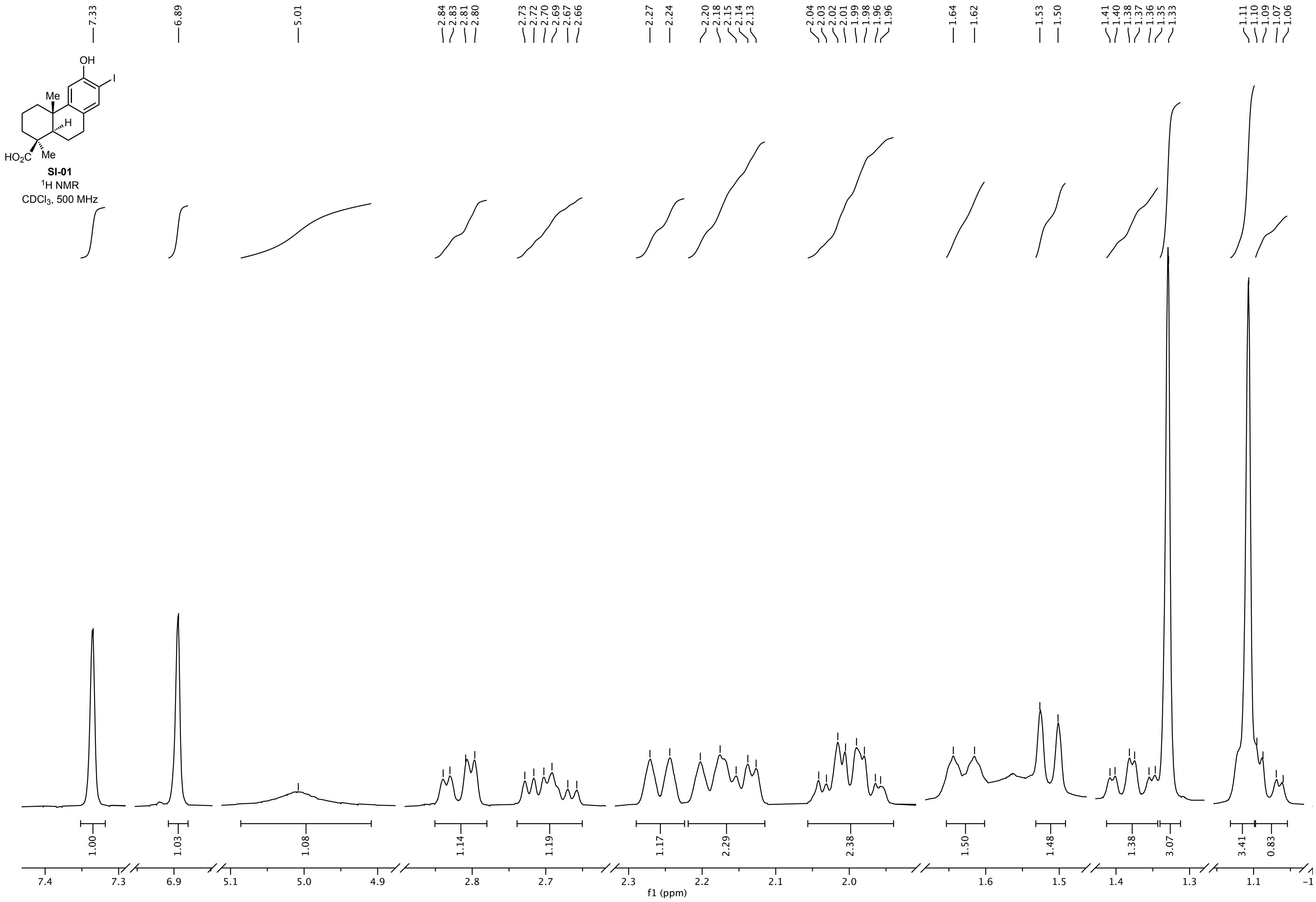

SI-01

^{1}H NMR

$CDCl_3$, 500 MHz

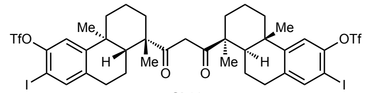

SI-02
^{1}H NMR
$CDCl_3$, 500 MHz

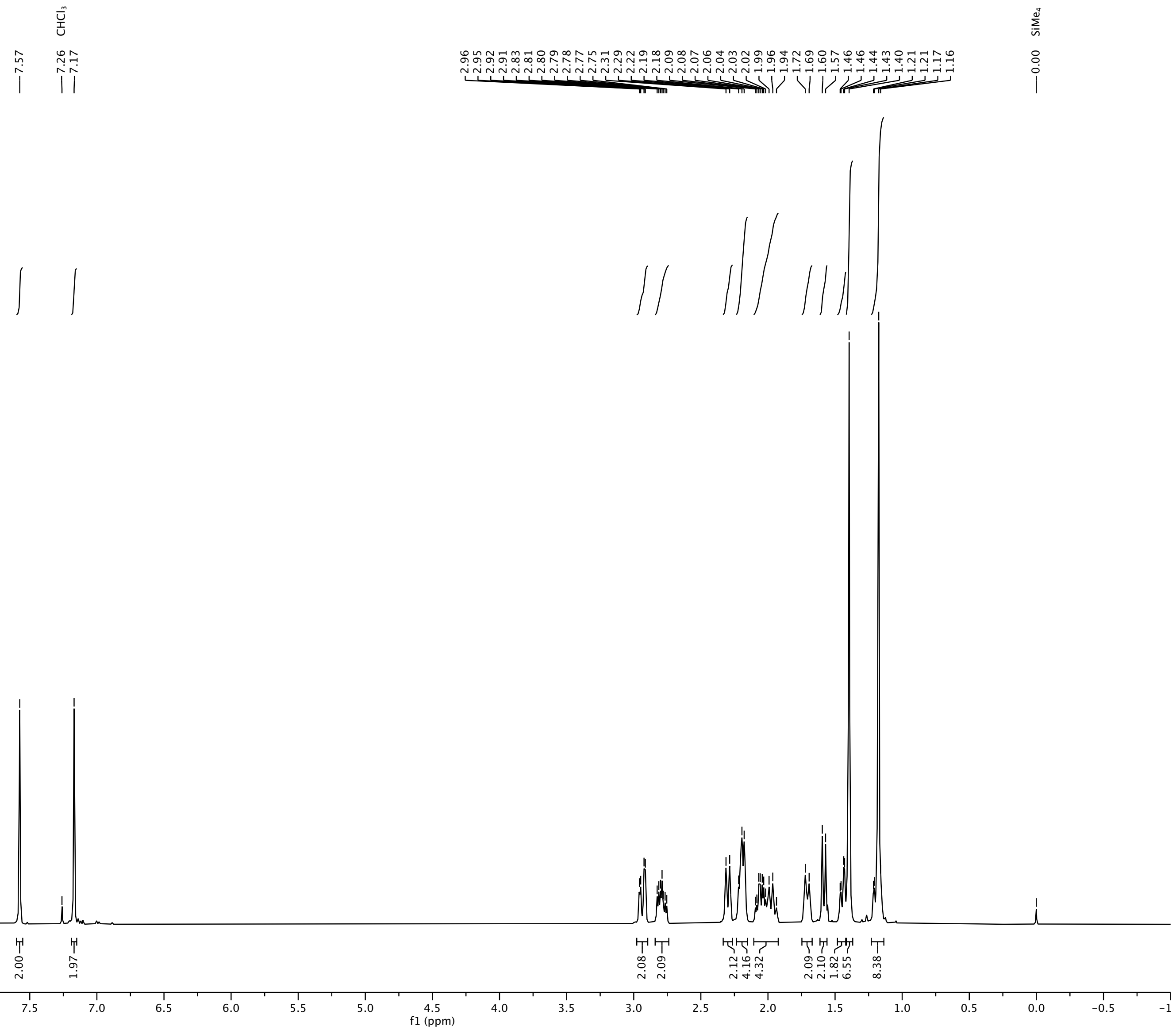

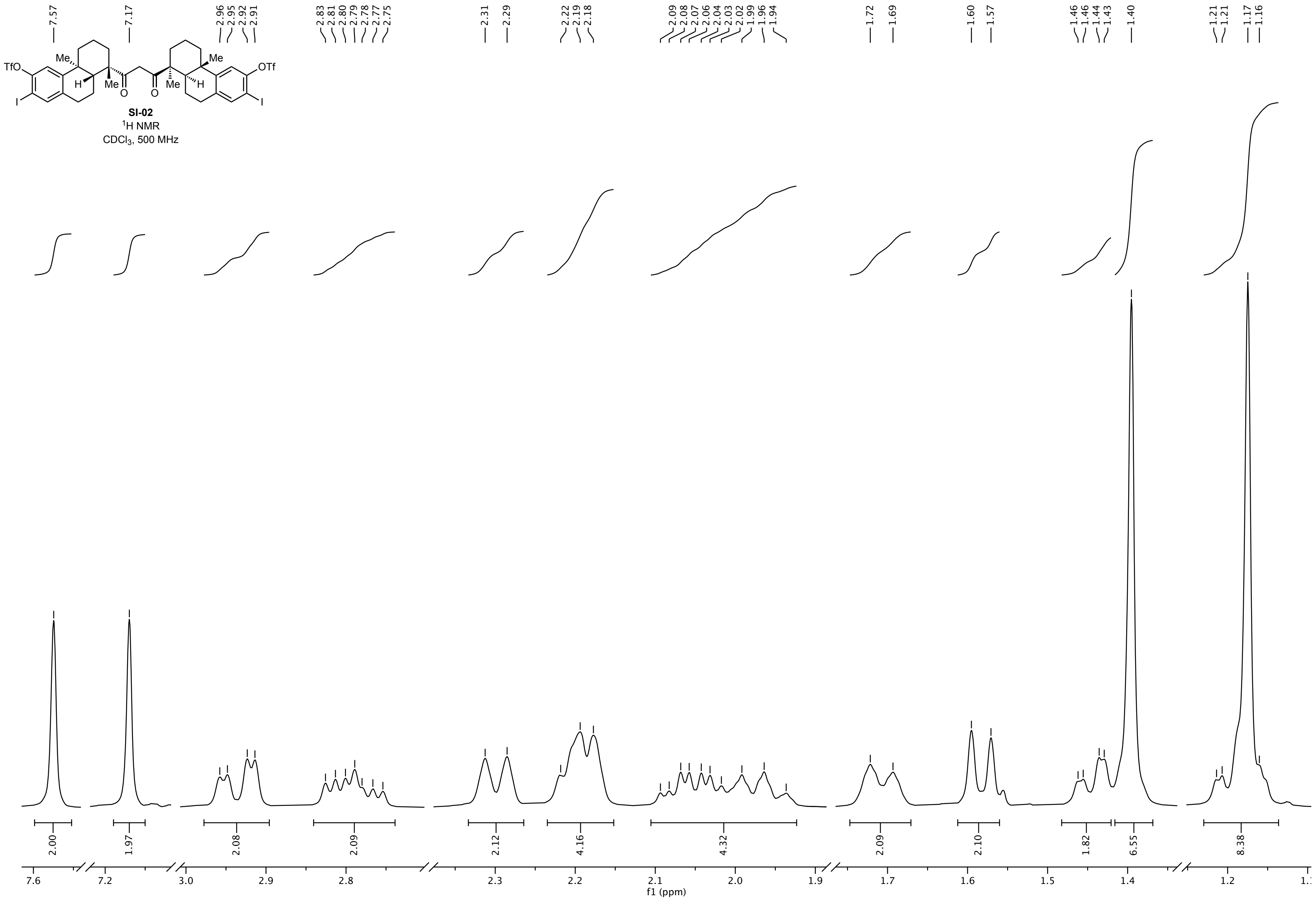
SI-02
^{1}H NMR
$CDCl_3$, 500 MHz
f1 (ppm)

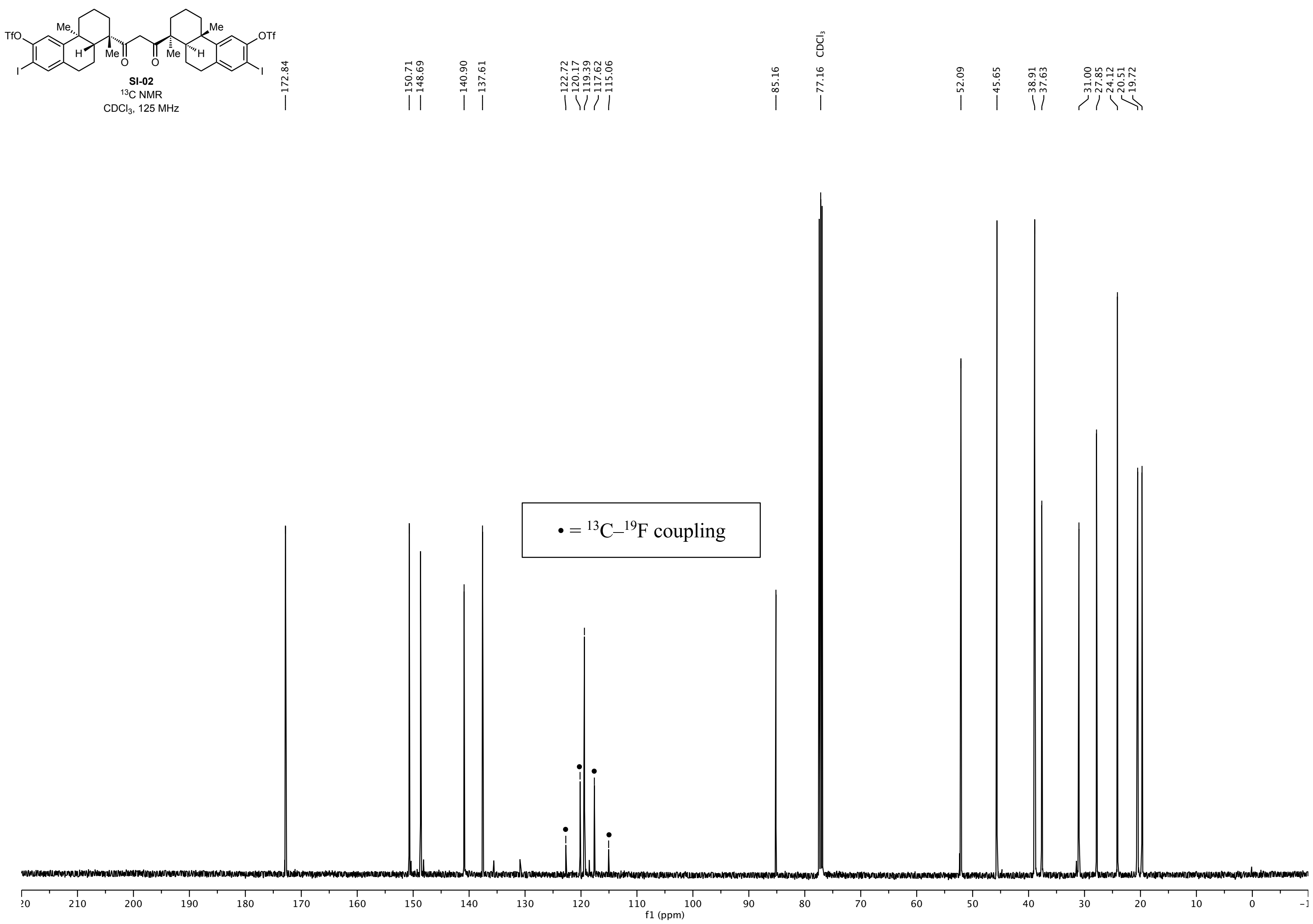
SI-02
^{13}C NMR
CDCl$_3$, 125 MHz
172.84
150.71
148.69
140.90
137.61
122.72
120.17
119.39
117.62
115.06
85.16
77.16 CDCl$_3$
52.09
45.65
38.91
37.63
31.00
27.85
24.12
20.51
19.72
• = ^{13}C–^{19}F coupling
f1 (ppm)

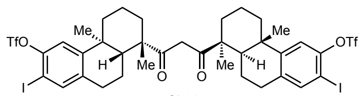

SI-02
^{19}F NMR
$CDCl_3$, 470 MHz

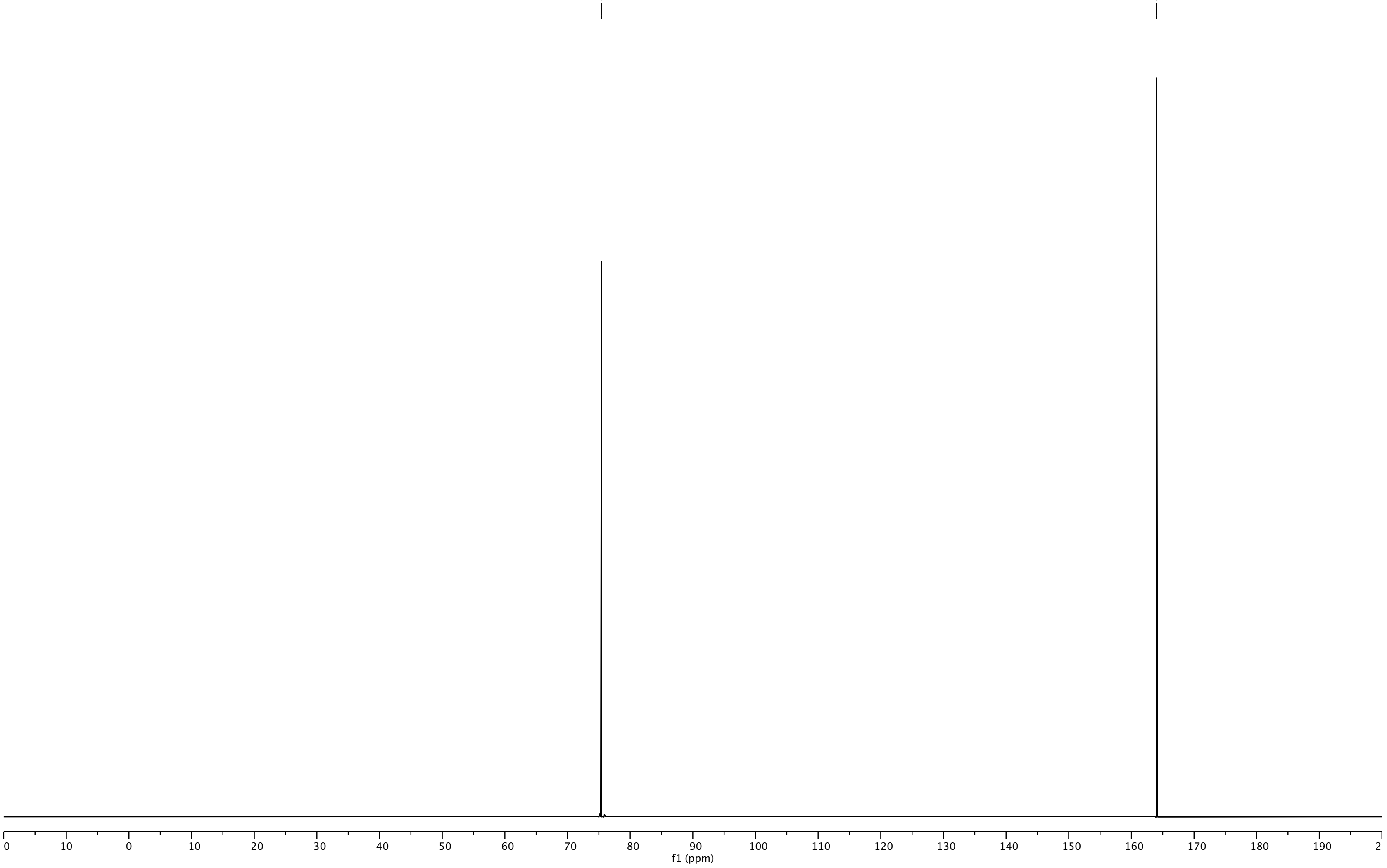

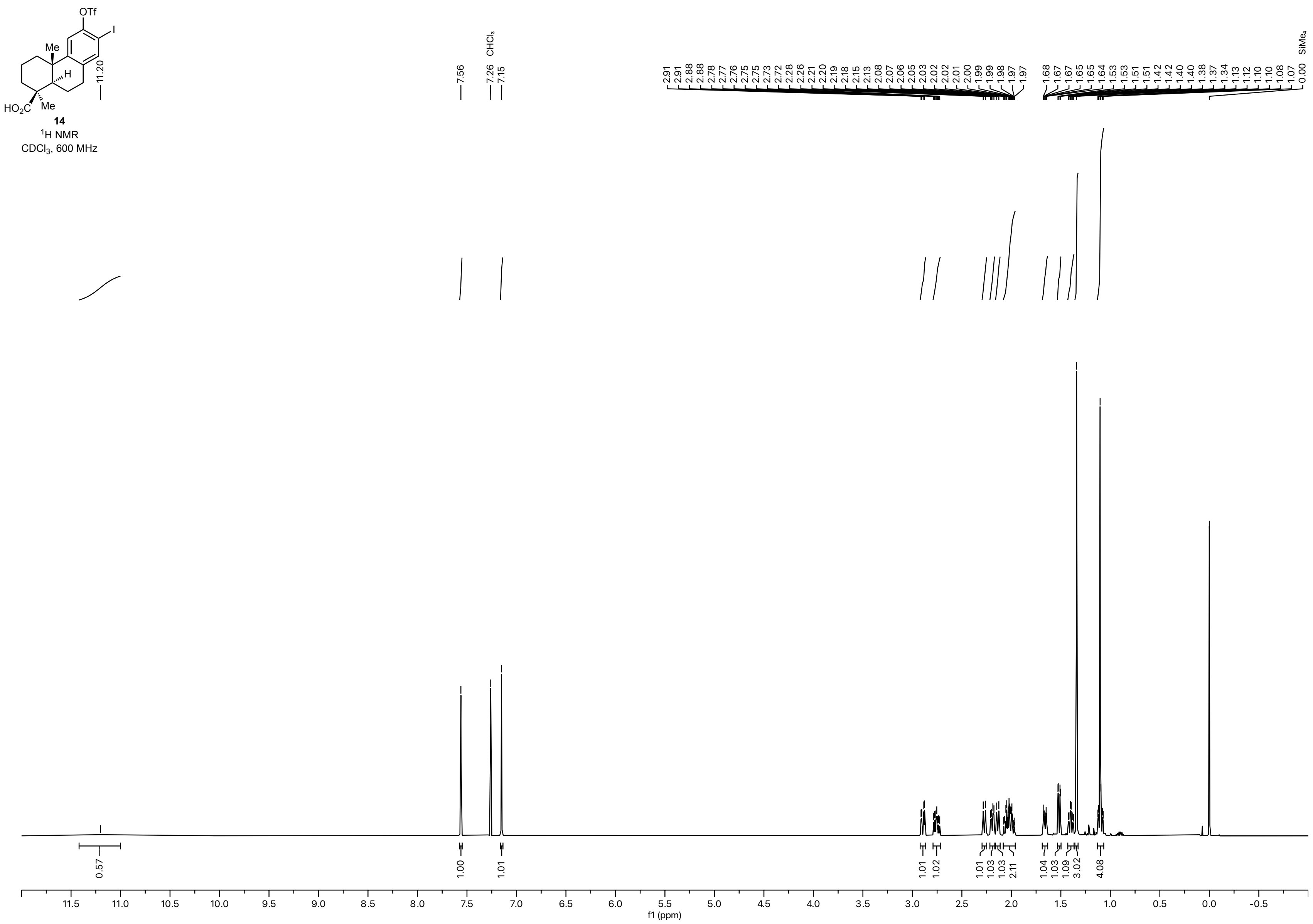
OTf
I
Me
H
HO2C
Me
14
1H NMR
CDCl3, 600 MHz
f1 (ppm)

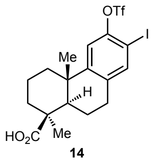

14

^{1}H NMR

$CDCl_3$, 600 MHz

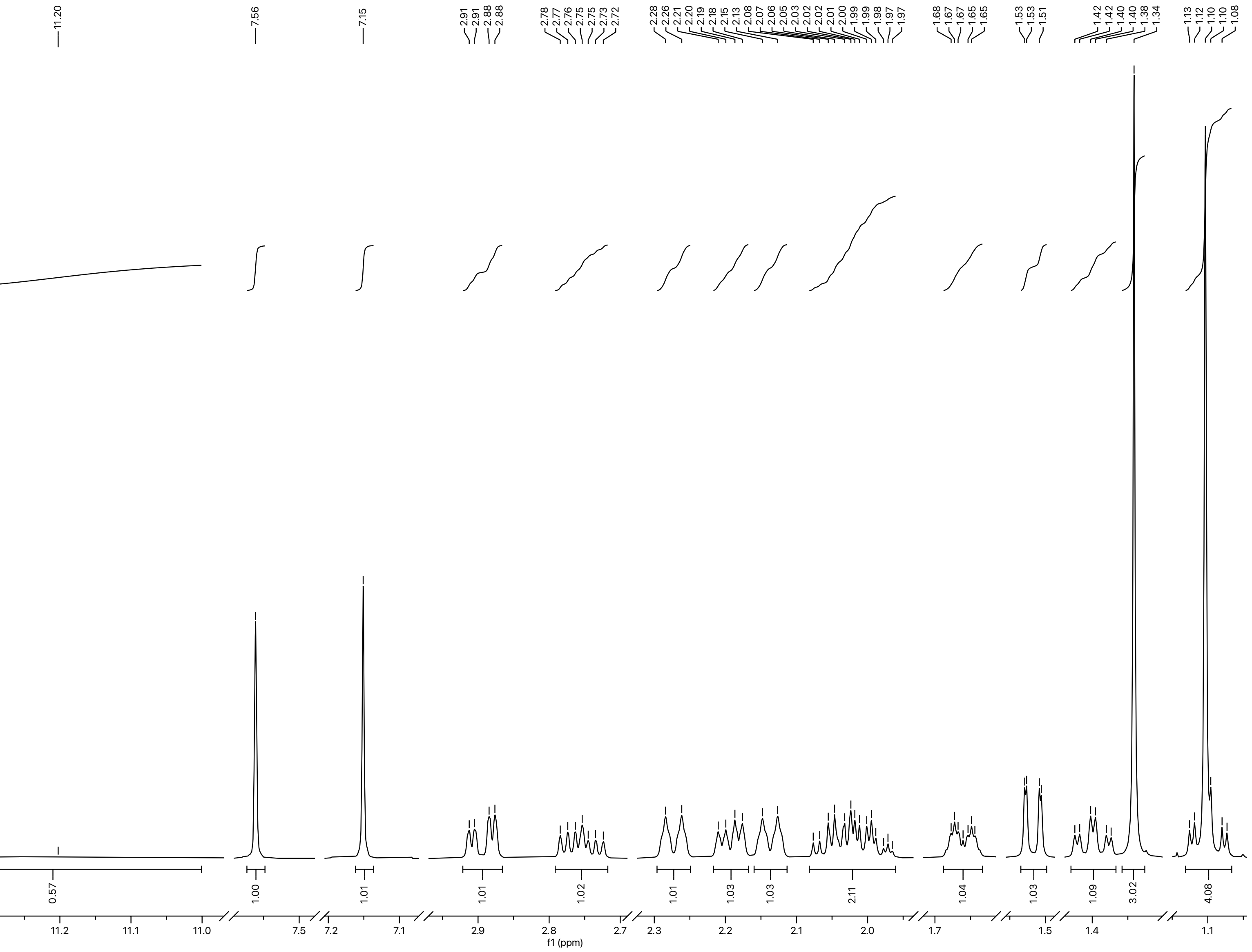

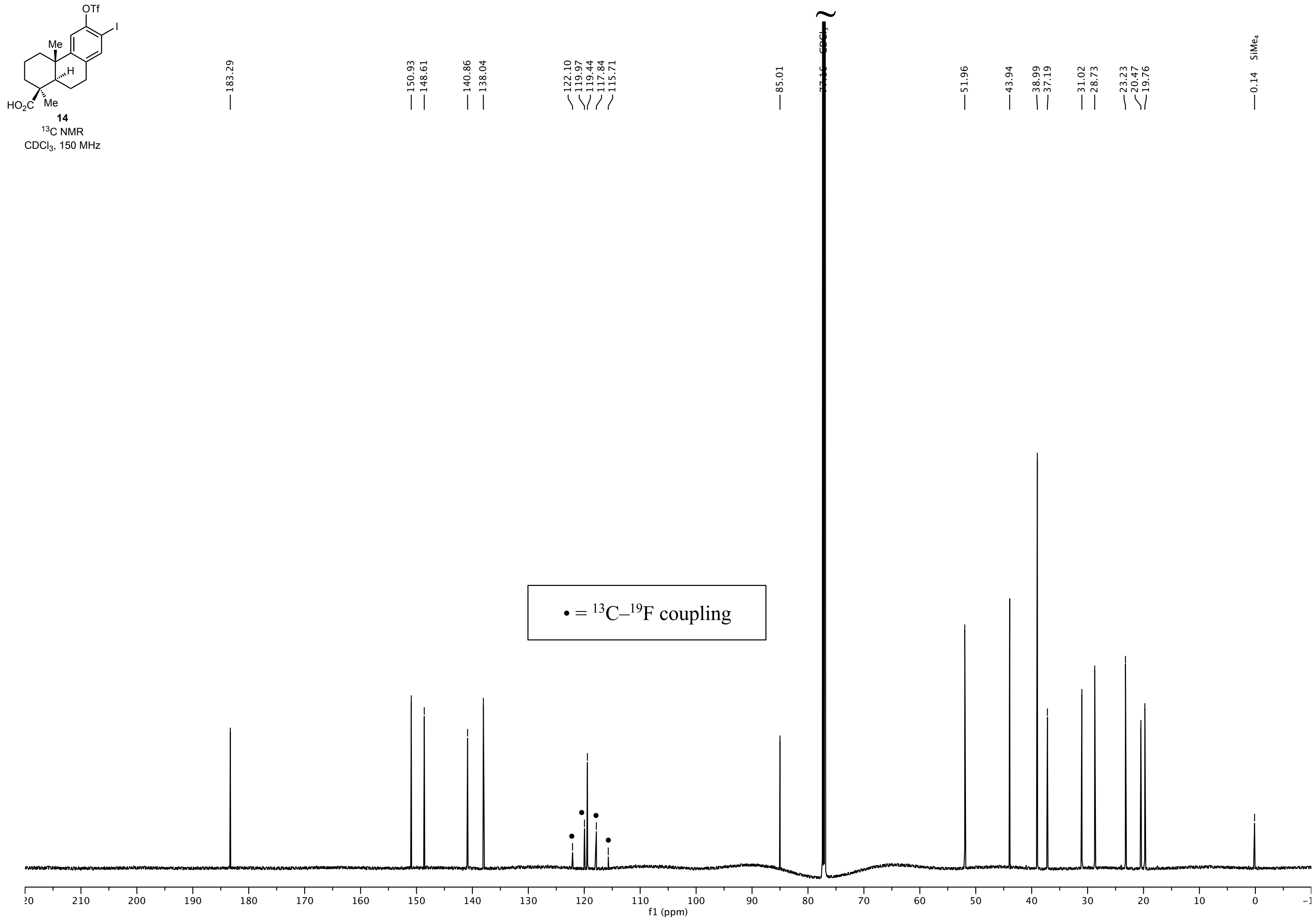

OTf
I
Me
H
HO2C
Me
14
13C NMR
CDCl3, 150 MHz
183.29
150.93
148.61
140.86
138.04
122.10
119.97
119.44
117.84
115.71
85.01
77.16 CDCl3
51.96
43.94
38.99
37.19
31.02
28.73
23.23
20.47
19.76
0.14 SiMe4
• = 13C–19F coupling
f1 (ppm)

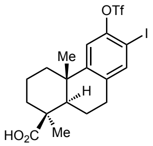

14

^{19}F NMR

$CDCl_3$, 470 MHz

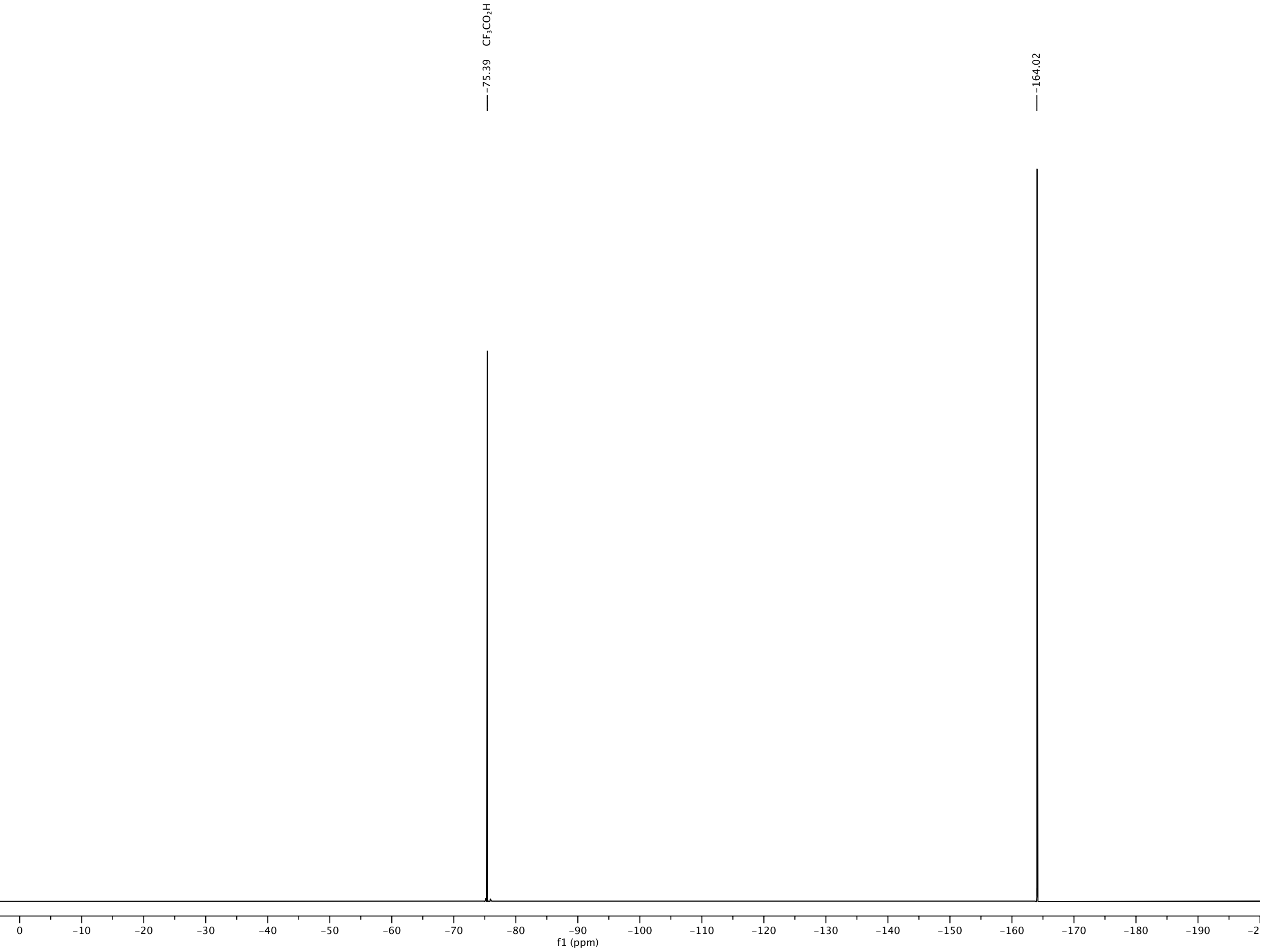

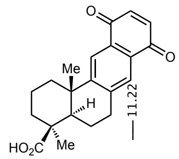

SI-03
^{1}H NMR
$CDCl_3$, 500 MHz

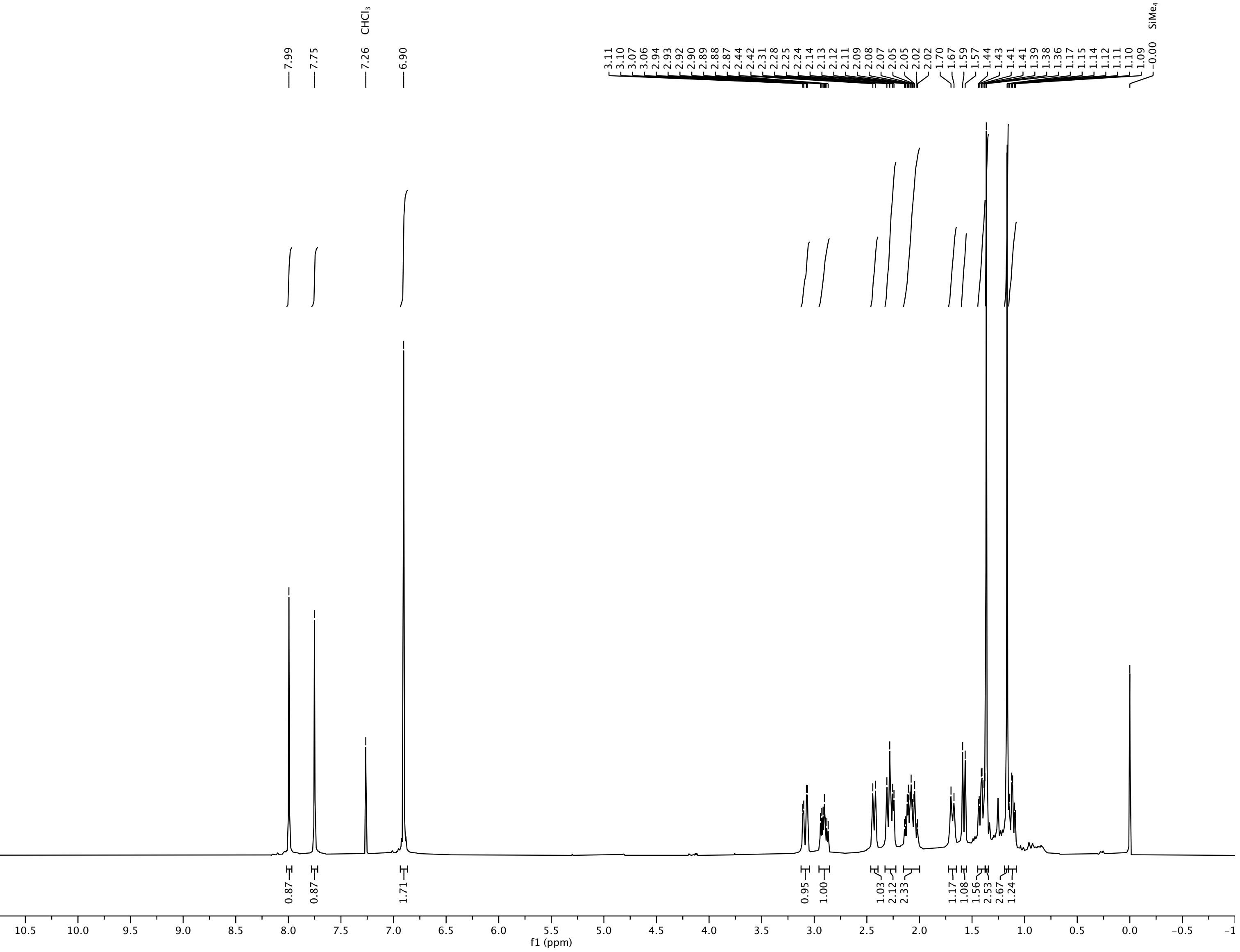

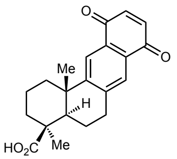

SI-03
^{1}H NMR
$CDCl_3$, 500 MHz

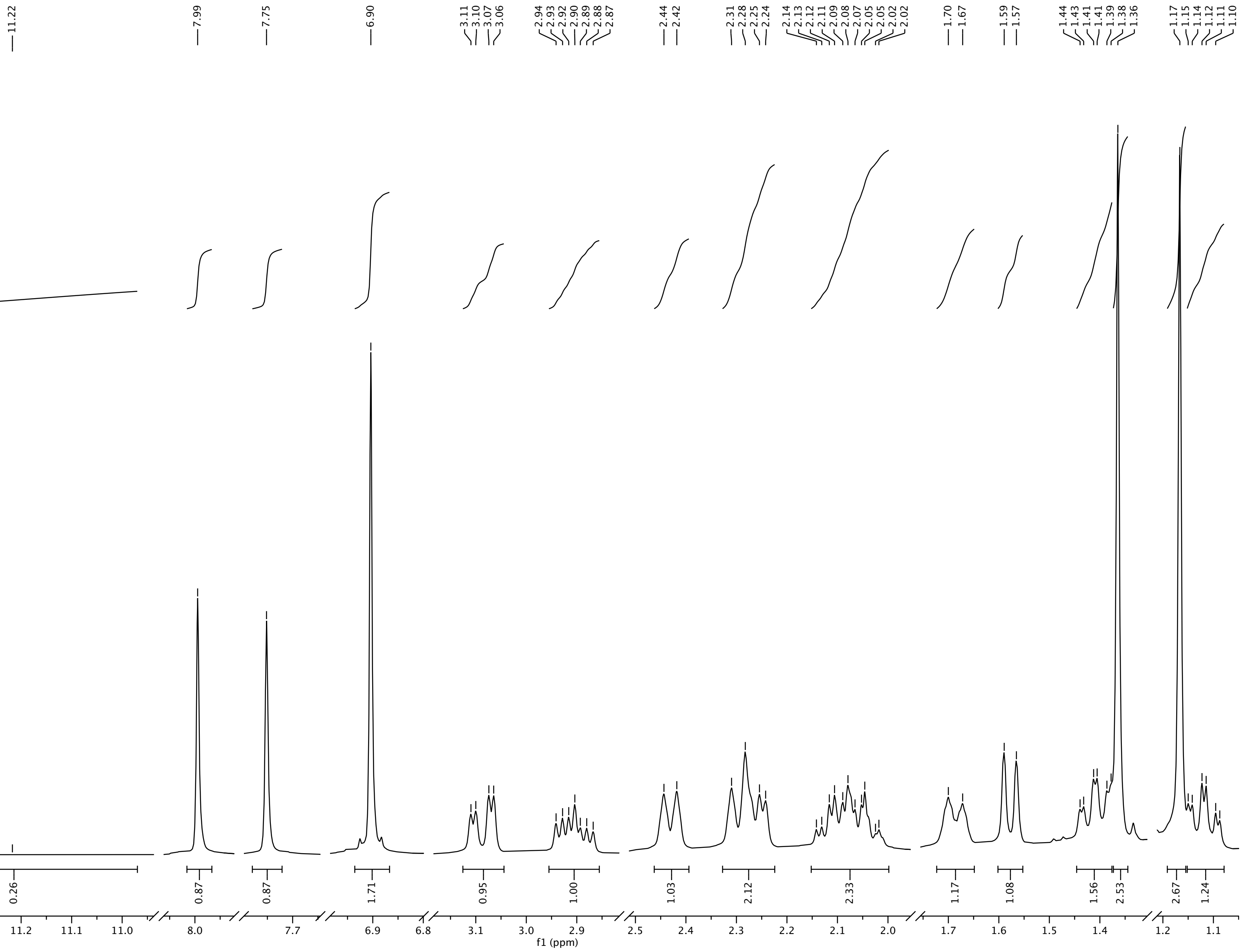

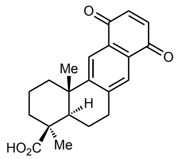

SI-03
^{13}C NMR
$CDCl_3$, 125 MHz

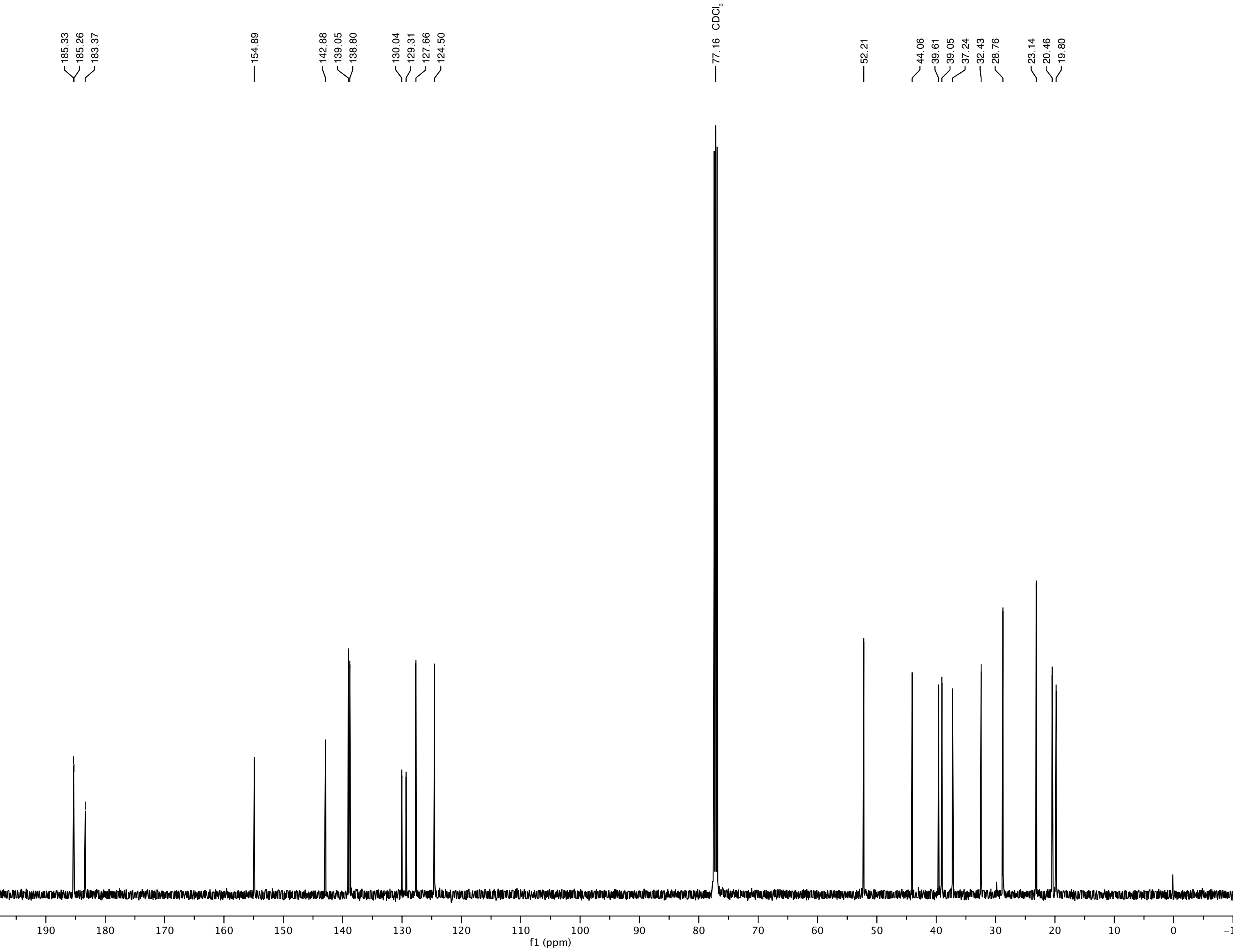

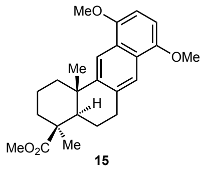

15
^{1}H NMR
$CDCl_3$, 500 MHz

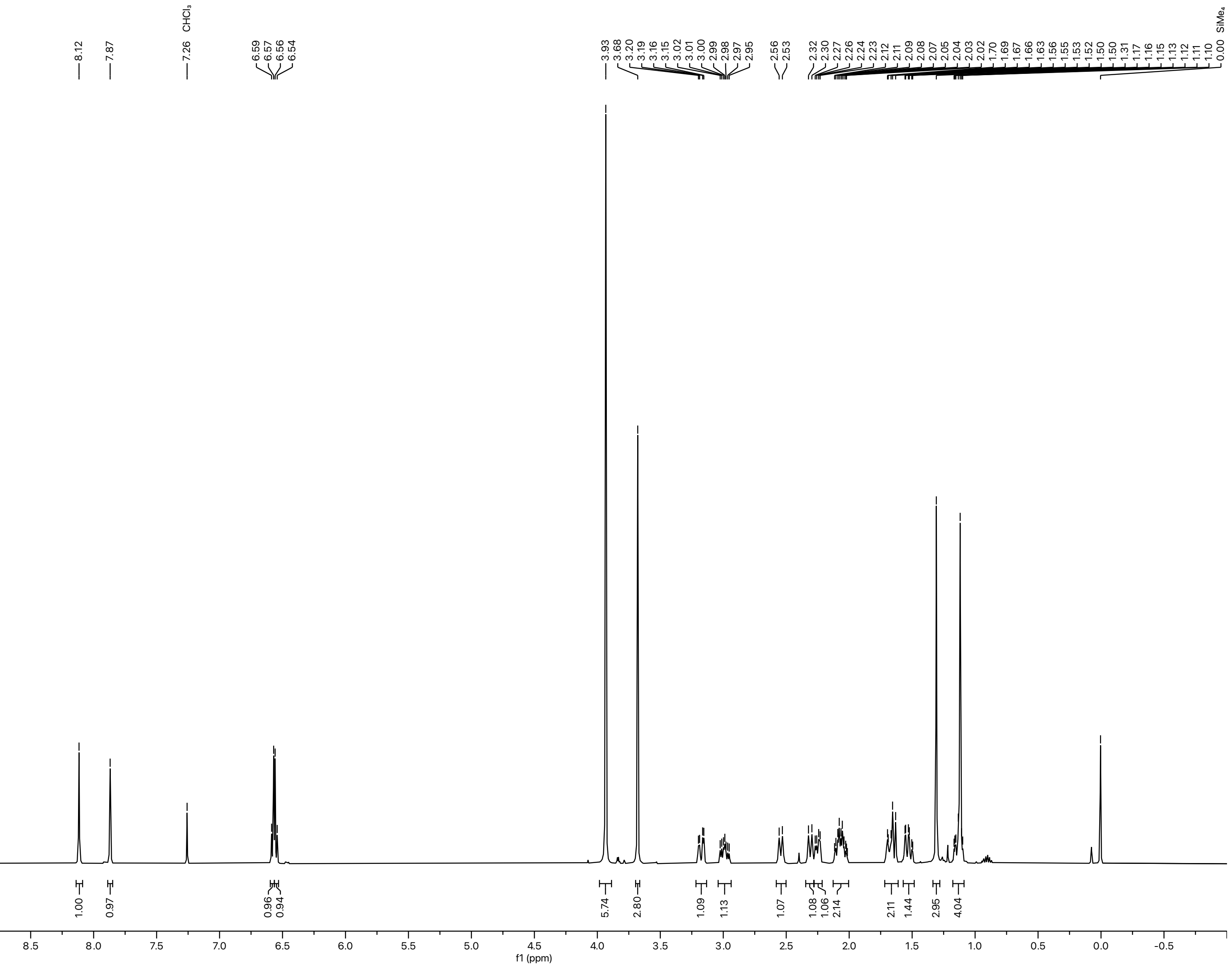

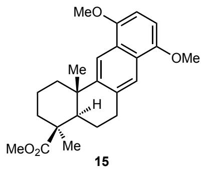

15

^1H NMR

$CDCl_3$, 500 MHz

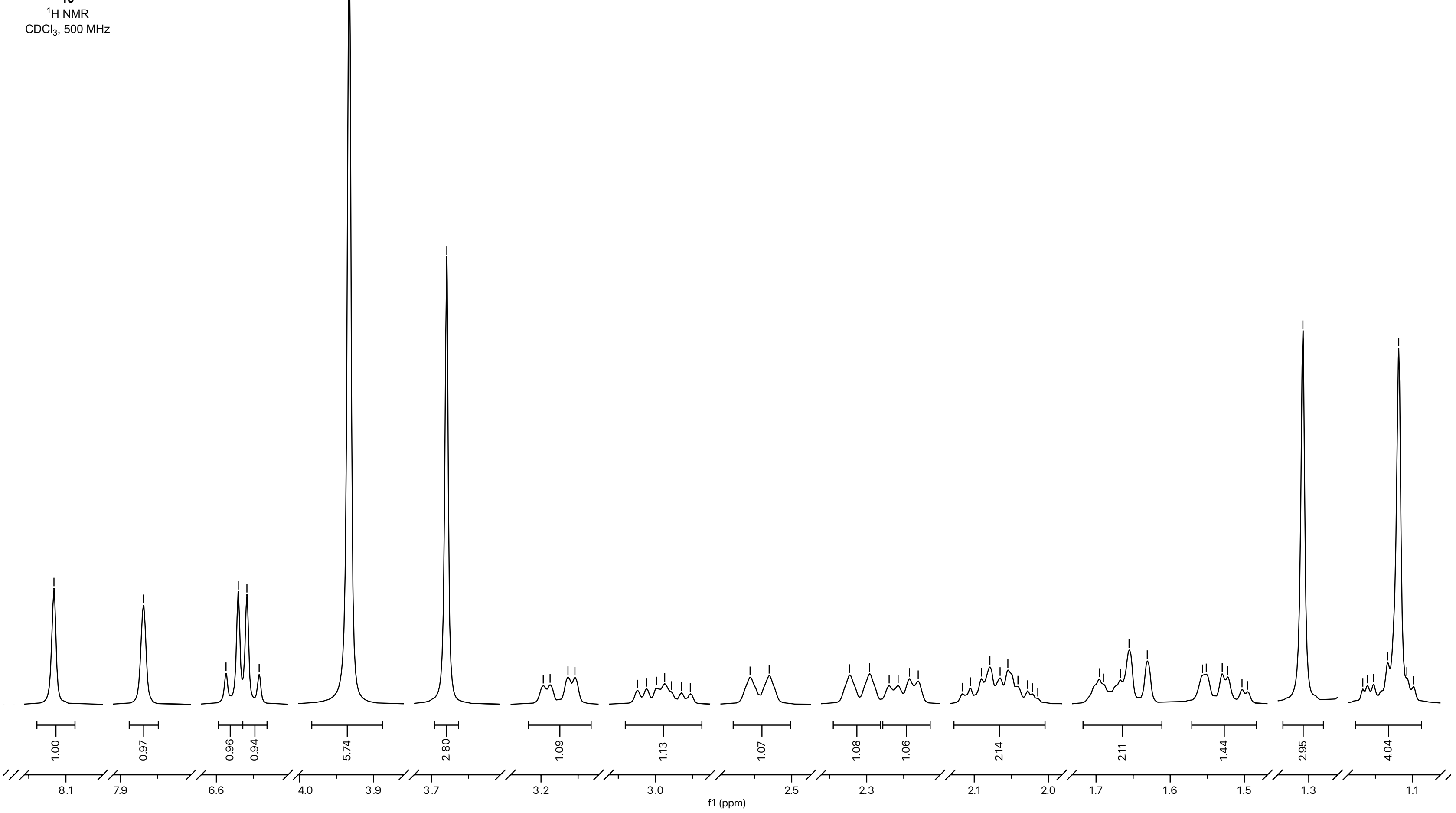

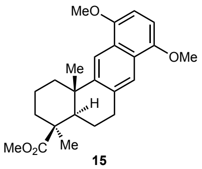

15

^{13}C NMR

$CDCl_3$, 125 MHz

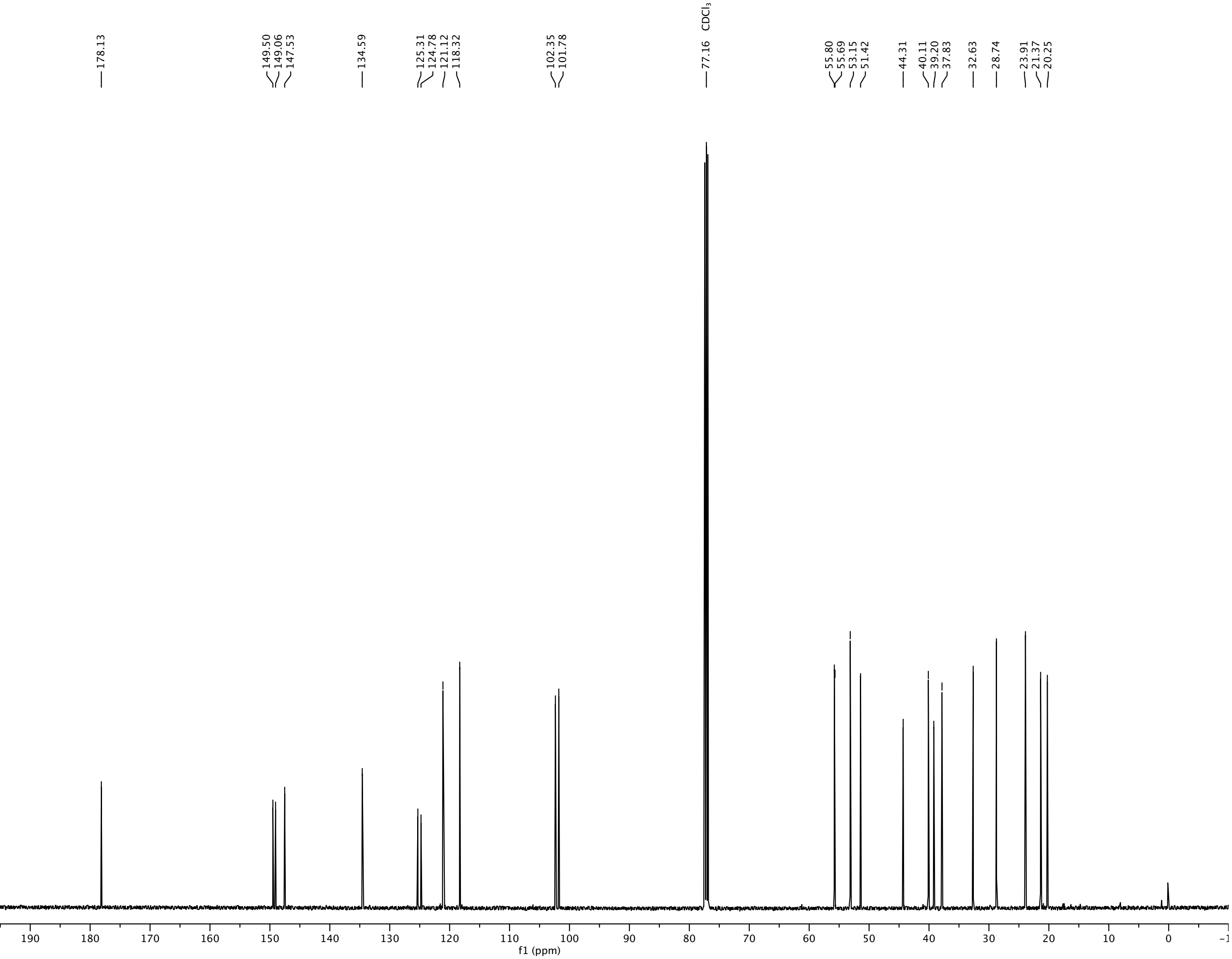

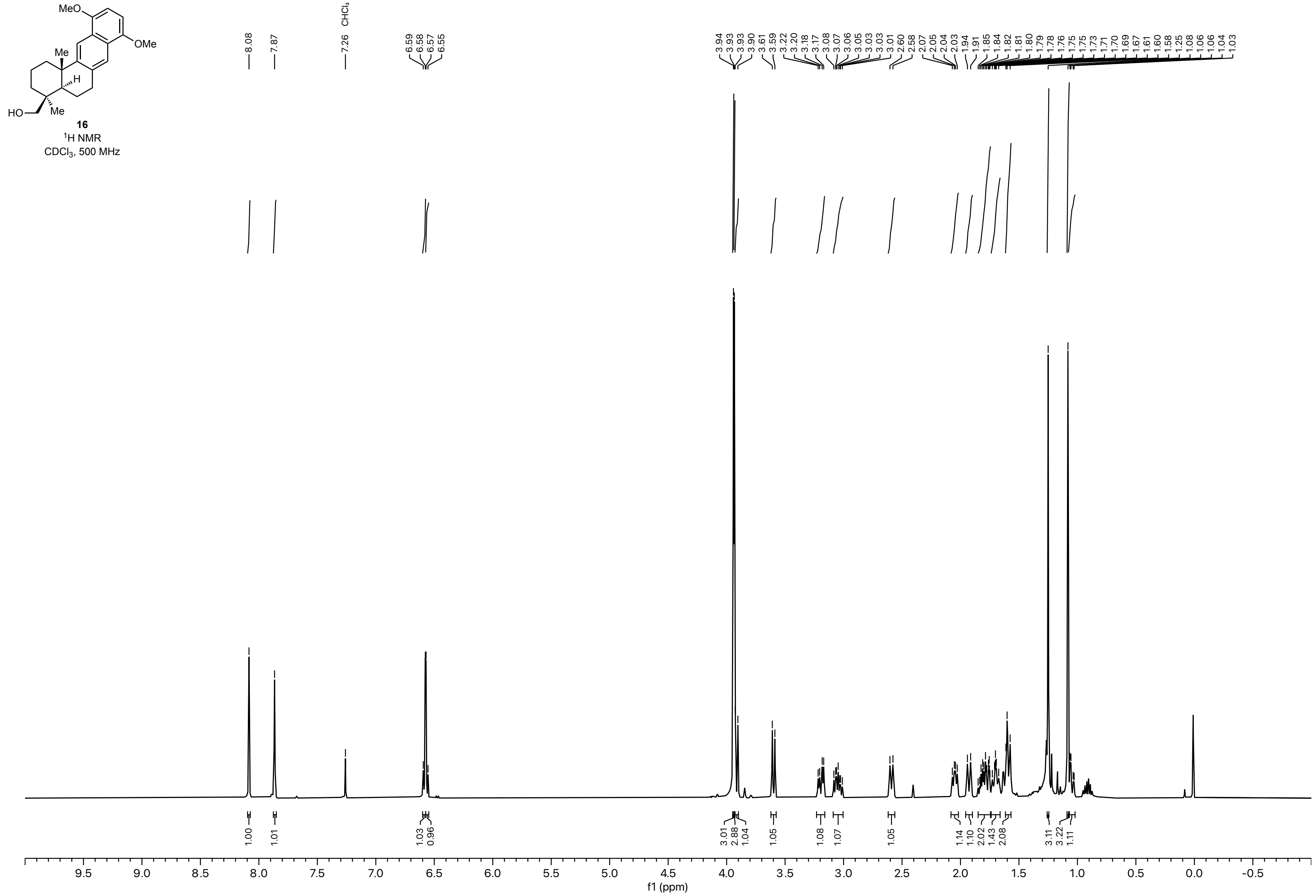
MeO
OMe
Me
H
HO
Me
16
^{1}H NMR
CDCl$_3$, 500 MHz
8.08
7.87
7.26 CHCl$_3$
6.59
6.58
6.57
6.55
3.94
3.93
3.93
3.90
3.61
3.59
3.22
3.20
3.18
3.17
3.08
3.07
3.06
3.05
3.03
3.03
3.01
2.60
2.58
2.07
2.05
2.04
2.03
1.94
1.91
1.85
1.84
1.82
1.81
1.80
1.79
1.78
1.76
1.75
1.75
1.73
1.71
1.70
1.69
1.67
1.61
1.60
1.58
1.25
1.08
1.06
1.06
1.04
1.03
1.00
1.01
1.03
0.96
3.01
2.88
1.04
1.05
1.08
1.07
1.05
1.14
1.10
2.02
1.43
2.08
3.11
3.22
1.11
9.5
9.0
8.5
8.0
7.5
7.0
6.5
6.0
5.5
5.0
4.5
4.0
3.5
3.0
2.5
2.0
1.5
1.0
0.5
0.0
-0.5
f1 (ppm)

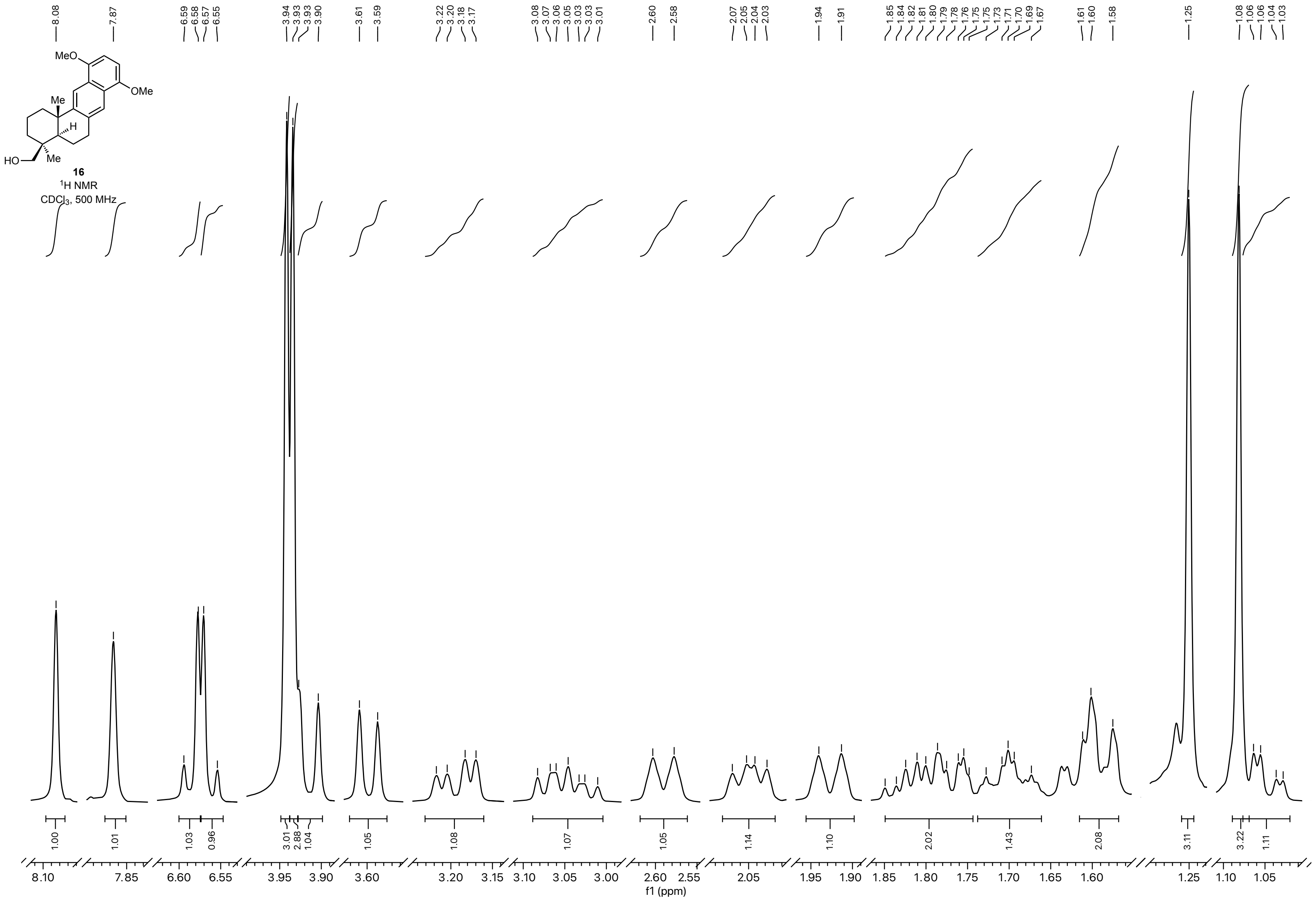
MeO
OMe
Me
H
HO
Me
16
^{1}H NMR
$CDCl_3$, 500 MHz
f1 (ppm)

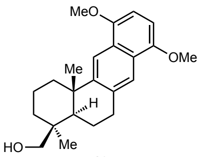

16
^{13}C NMR
$CDCl_3$, 125 MHz

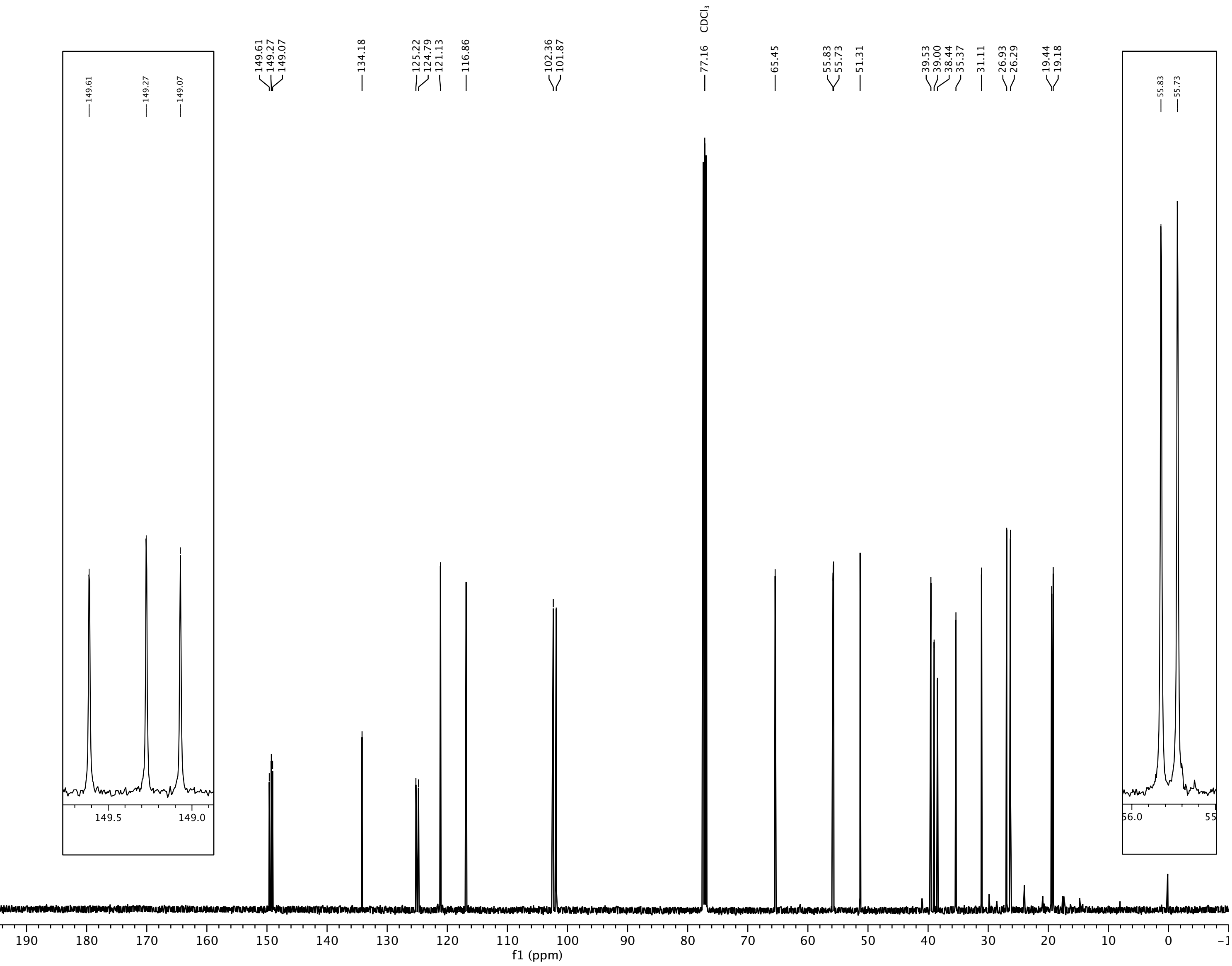

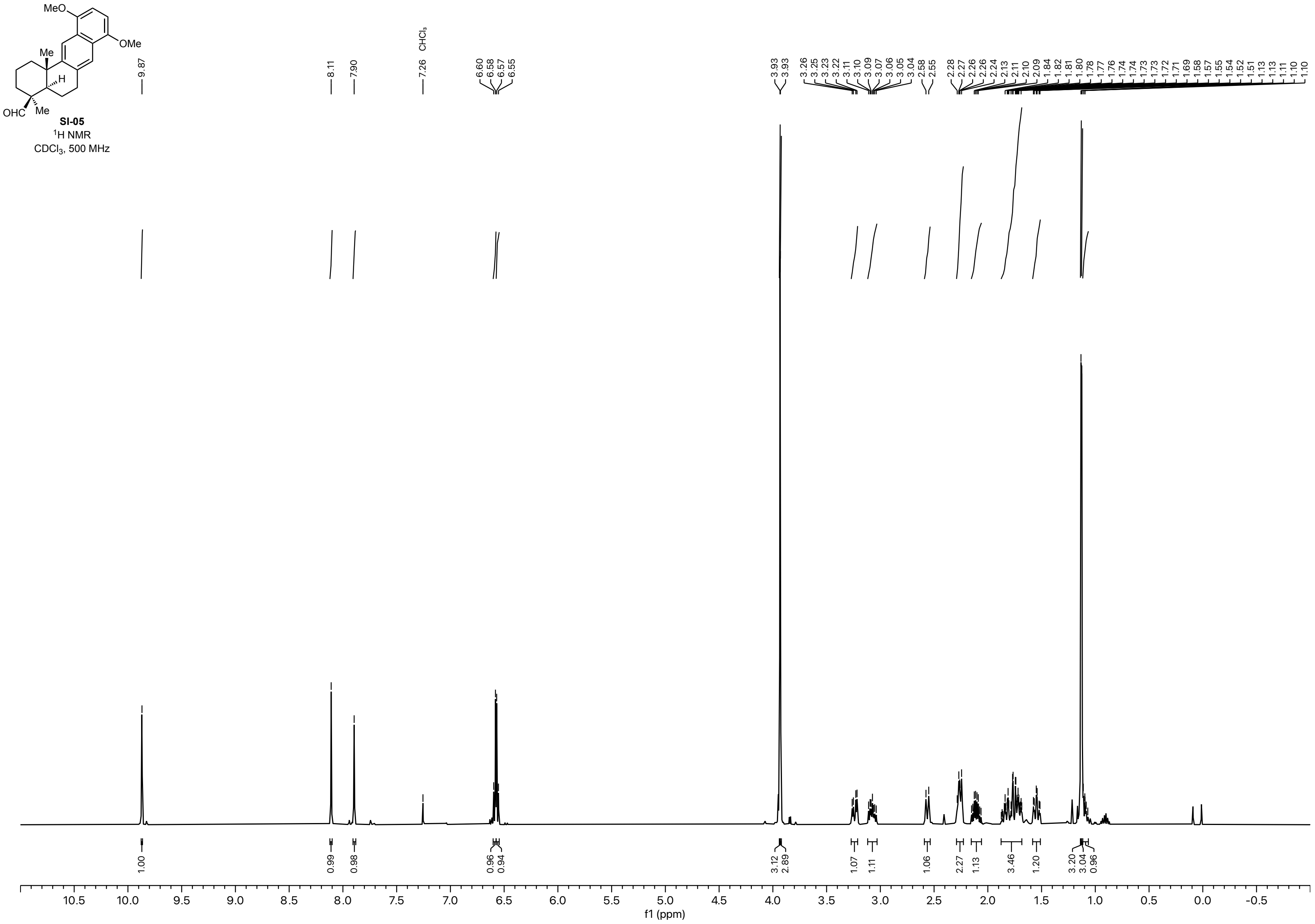
MeO
OMe
Me
H
OHC
Me
SI-05
1H NMR
CDCl3, 500 MHz
9.87
8.11
7.90
7.26 CHCl3
6.60 6.58 6.57 6.55
3.93 3.93
3.26 3.25 3.23 3.22 3.11 3.10 3.09 3.07 3.06 3.05 3.04
2.58 2.55
2.28 2.27 2.26 2.26 2.24 2.13 2.11 2.10 2.09
1.84 1.82 1.81 1.80 1.78 1.77 1.76 1.74 1.74 1.73 1.73 1.72 1.71 1.69 1.58 1.57 1.55 1.54 1.52 1.51
1.13 1.13 1.11 1.10 1.10
1.00
0.99
0.98
0.96 0.94
3.12 2.89
1.07
1.11
1.06
2.27
1.13
3.46
1.20
3.20 3.04 0.96
10.5 10.0 9.5 9.0 8.5 8.0 7.5 7.0 6.5 6.0 5.5 5.0 4.5 4.0 3.5 3.0 2.5 2.0 1.5 1.0 0.5 0.0 -0.5
f1 (ppm)

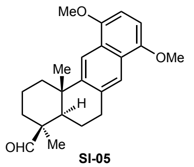

SI-05
^{1}H NMR
$CDCl_3$, 500 MHz

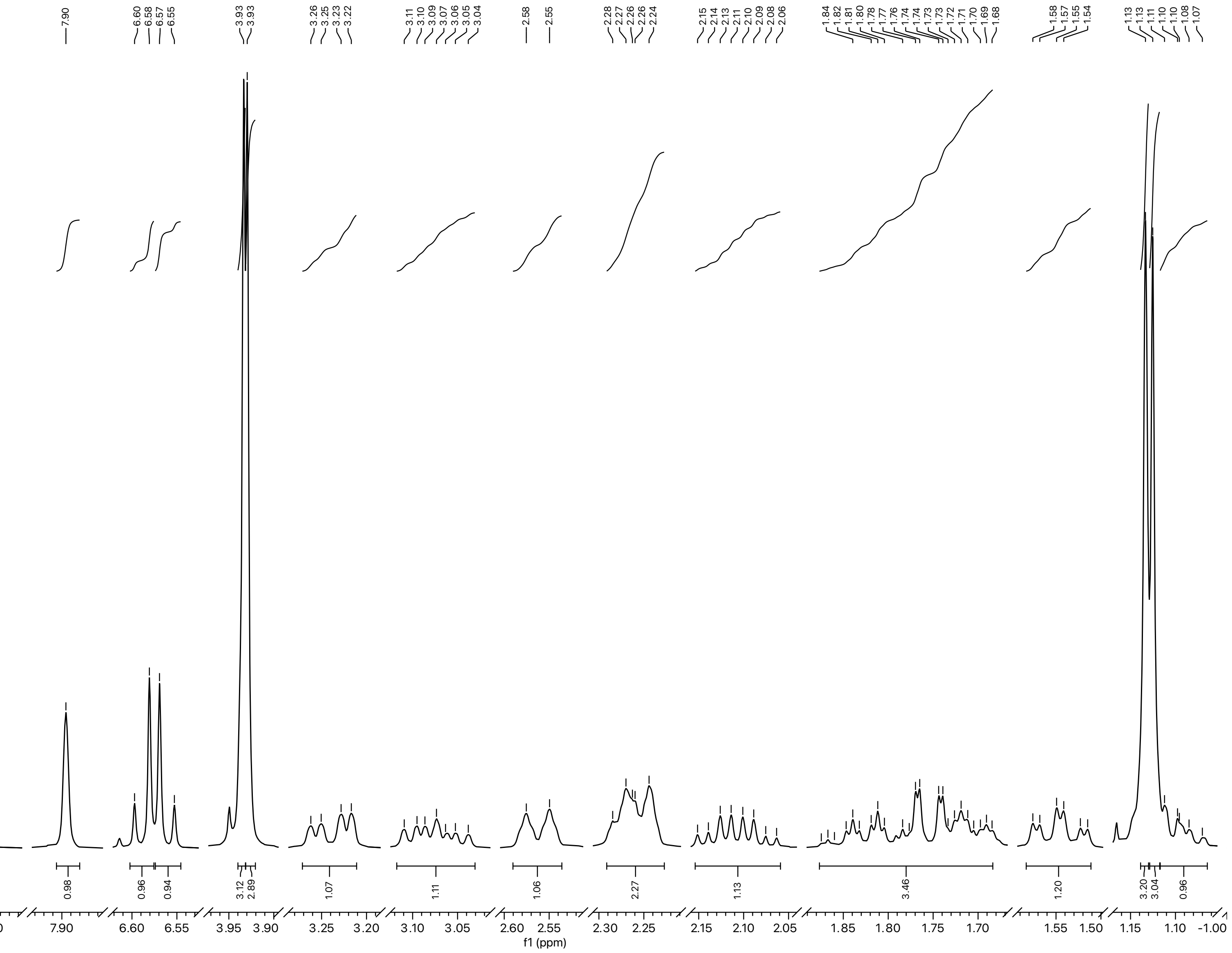

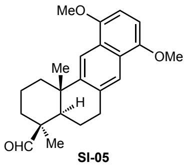

SI-05
^{13}C NMR
$CDCl_3$, 125 MHz

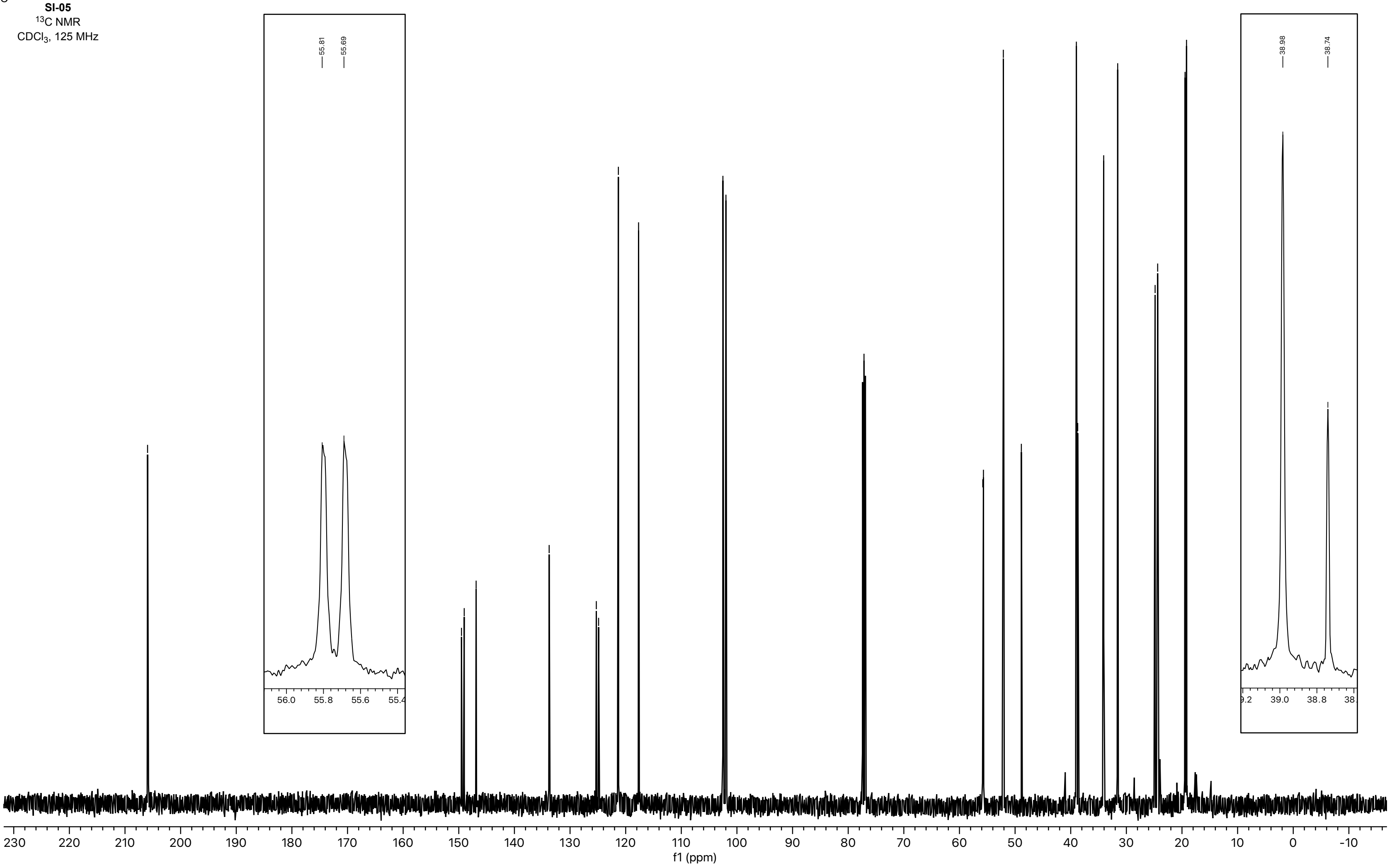

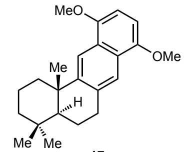

17
^{1}H NMR
$CDCl_3$, 500 MHz

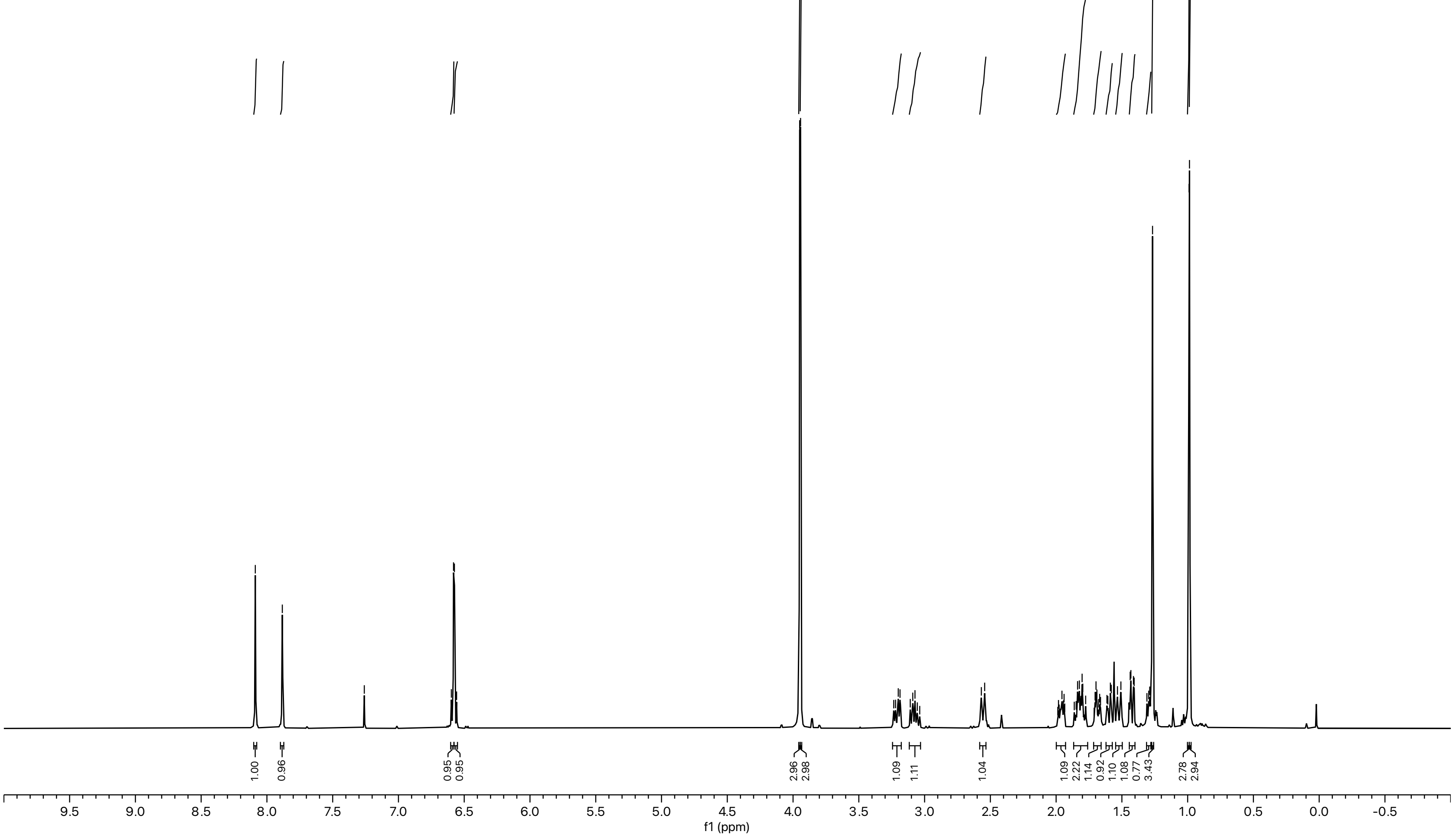

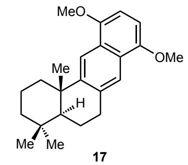

17
^{1}H NMR
$CDCl_3$, 500 MHz

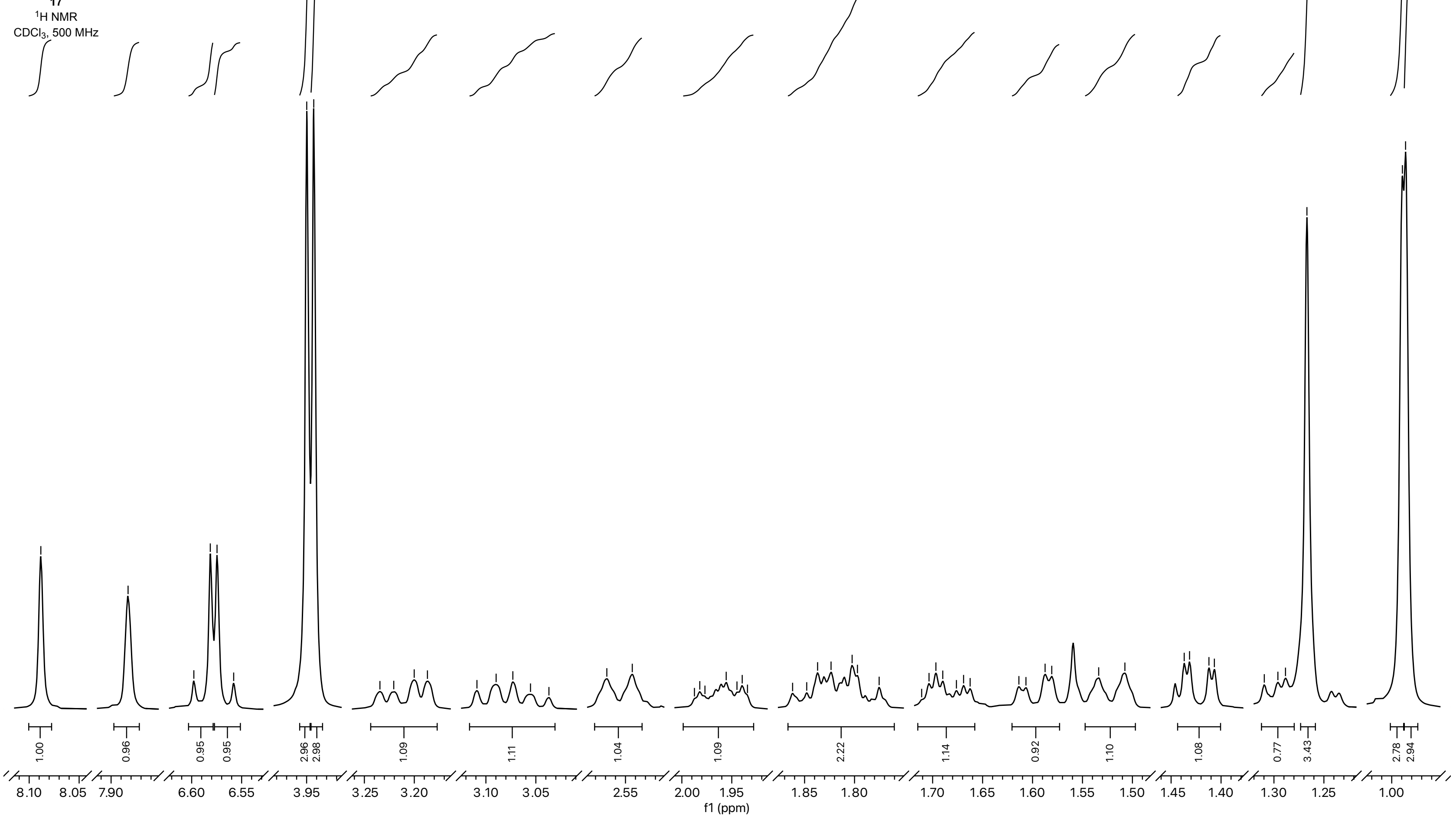

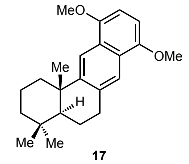

17
^{13}C NMR
$CDCl_3$, 125 MHz

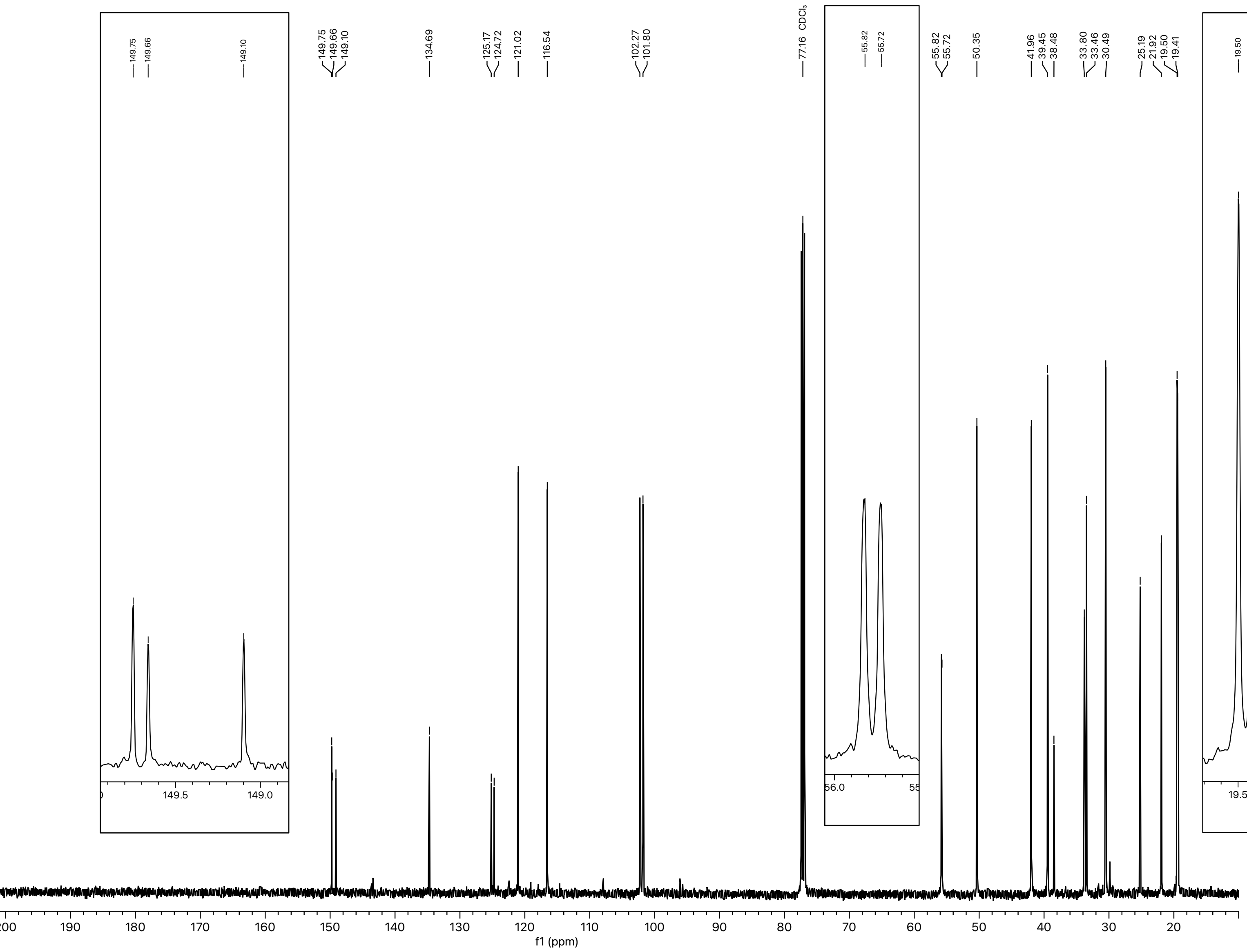

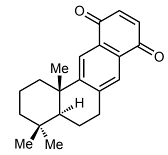

(+)-2: *ent*-cyclozonarone
^{1}H NMR
$CDCl_3$, 500 MHz

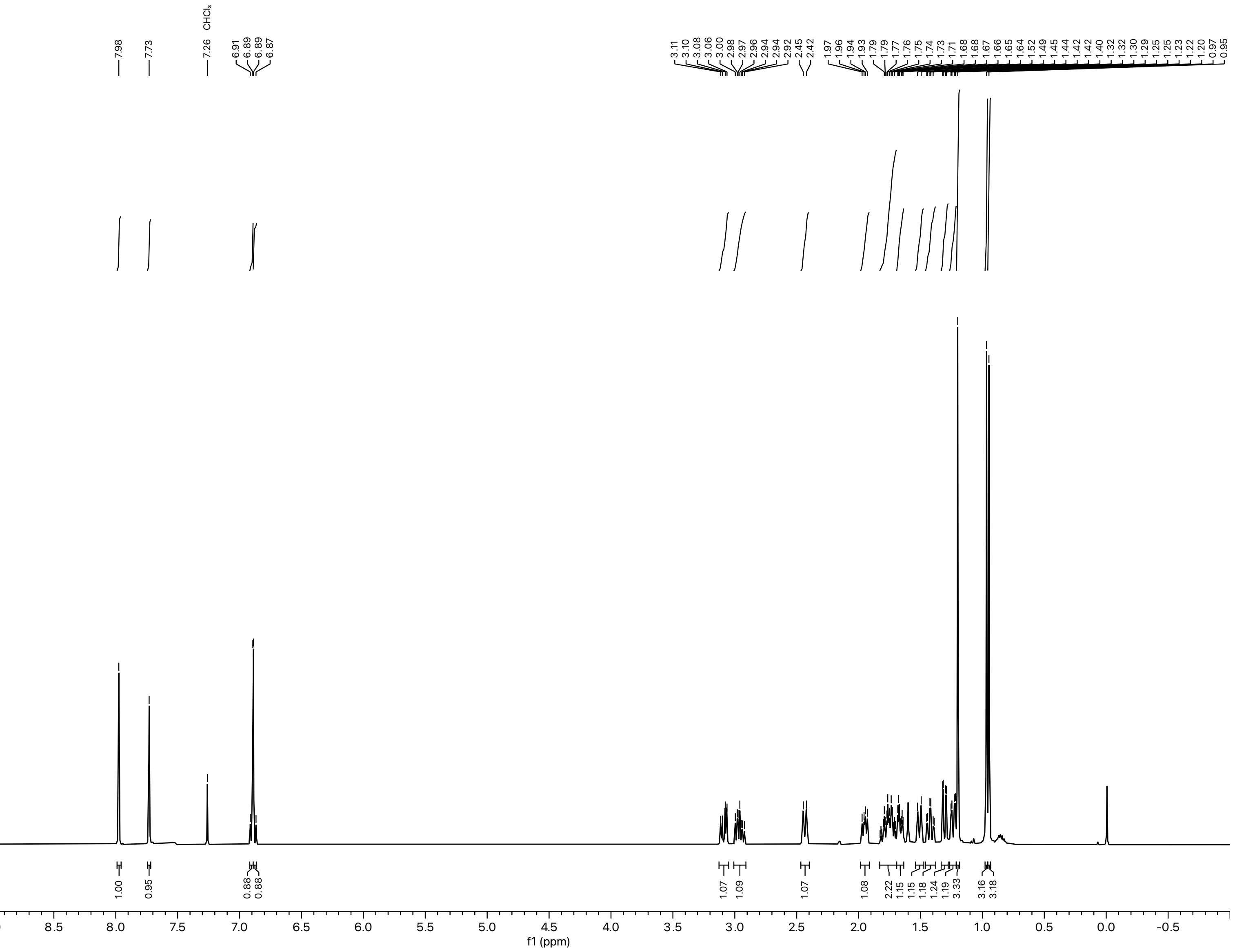

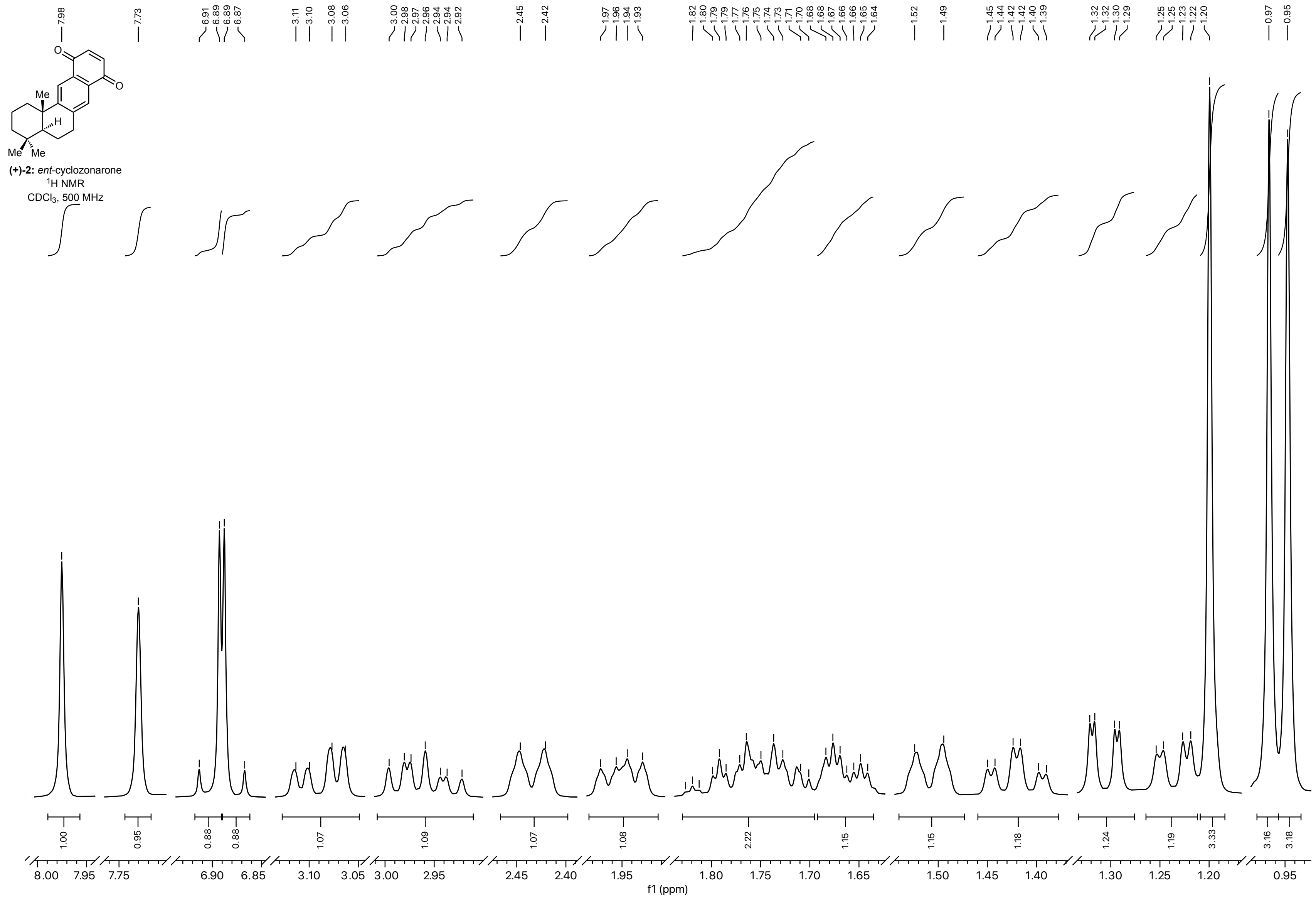
(+)-2: ent-cyclozonarone
1H NMR
CDCl3, 500 MHz
f1 (ppm)

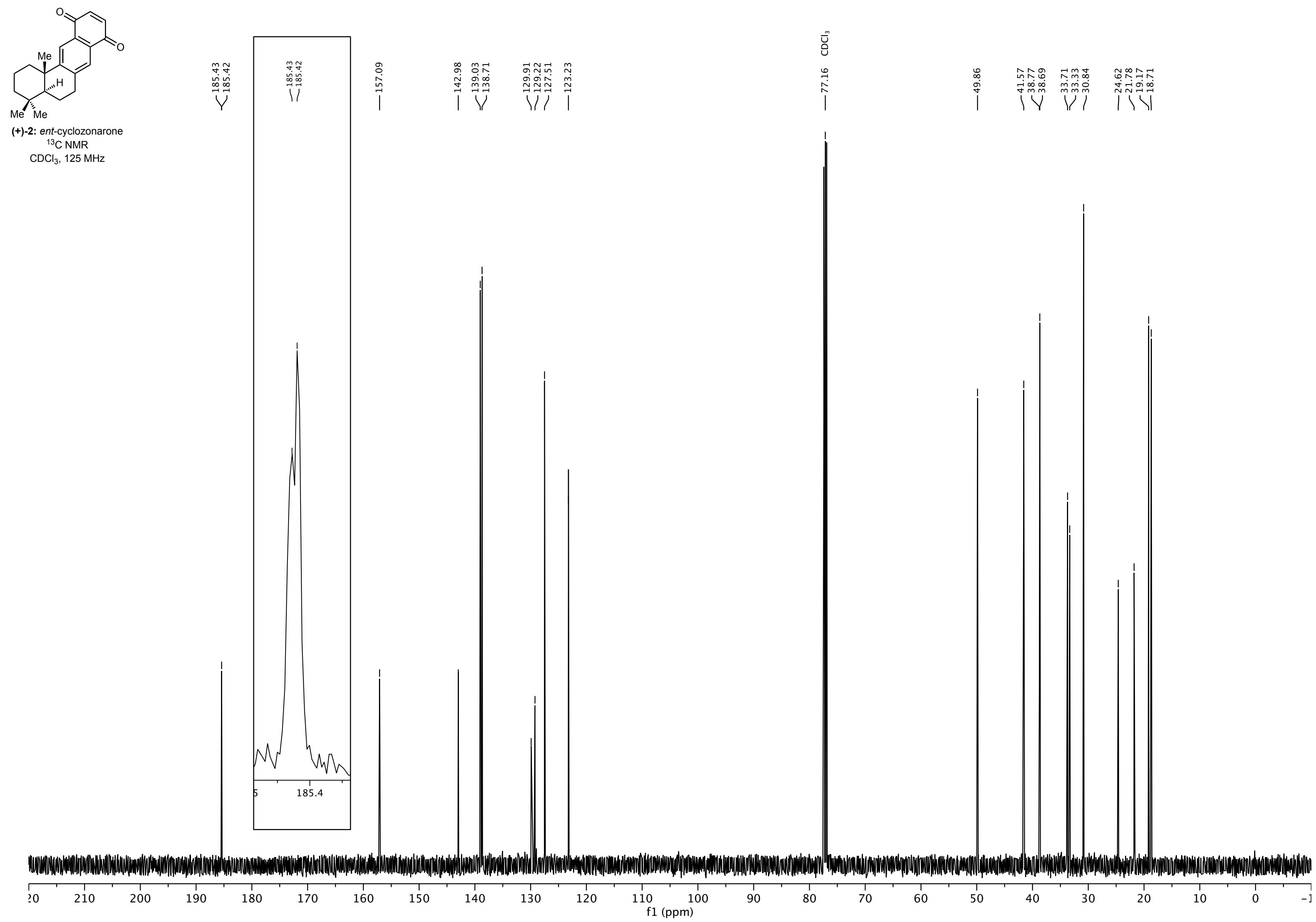
(+)-2: ent-cyclozonarone
13C NMR
CDCl3, 125 MHz
185.43
185.42
157.09
142.98
139.03
138.71
129.91
129.22
127.51
123.23
CDCl3
77.16
49.86
41.57
38.77
38.69
33.71
33.33
30.84
24.62
21.78
19.17
18.71
f1 (ppm)

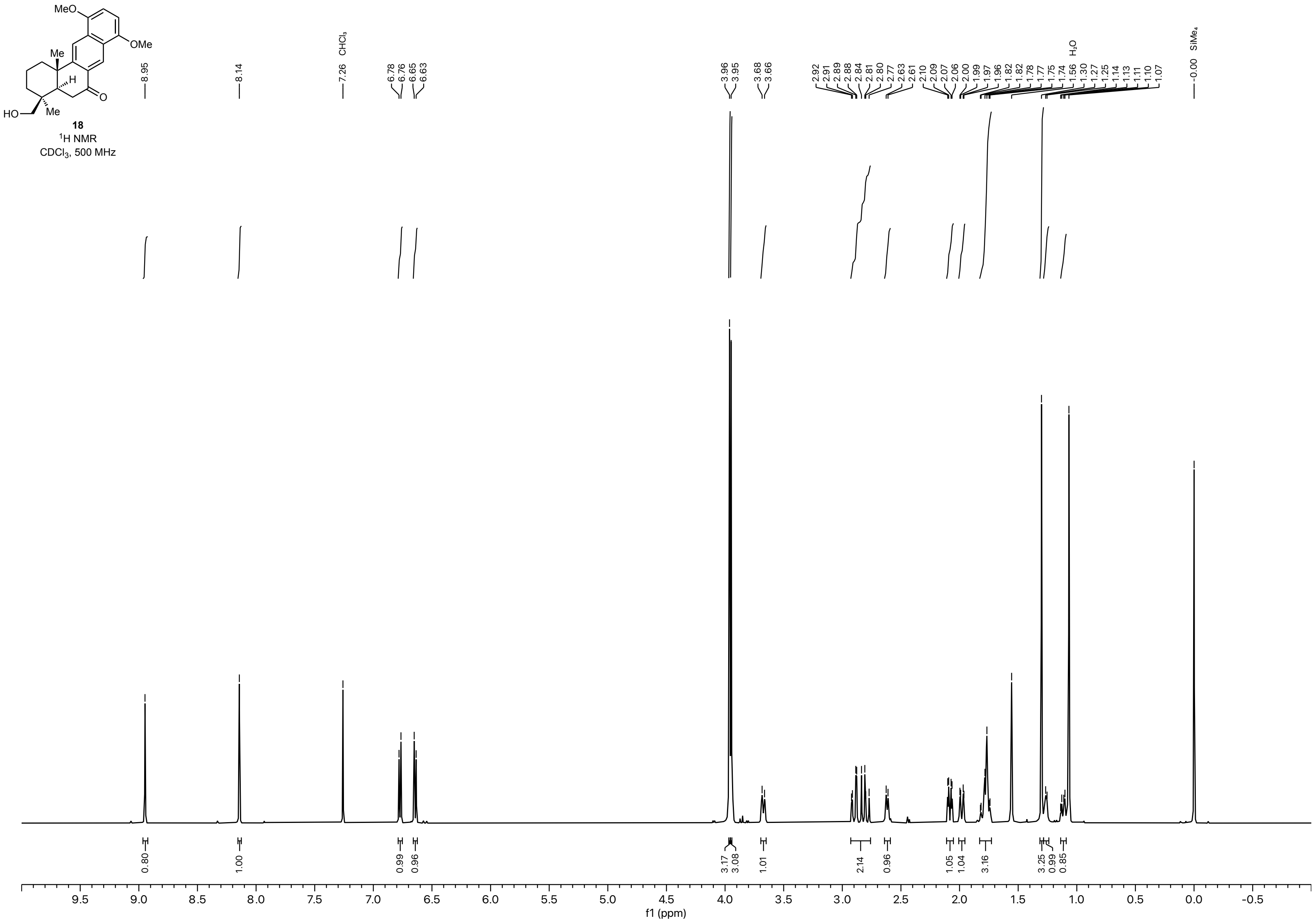

18

^1H NMR

$CDCl_3$, 500 MHz

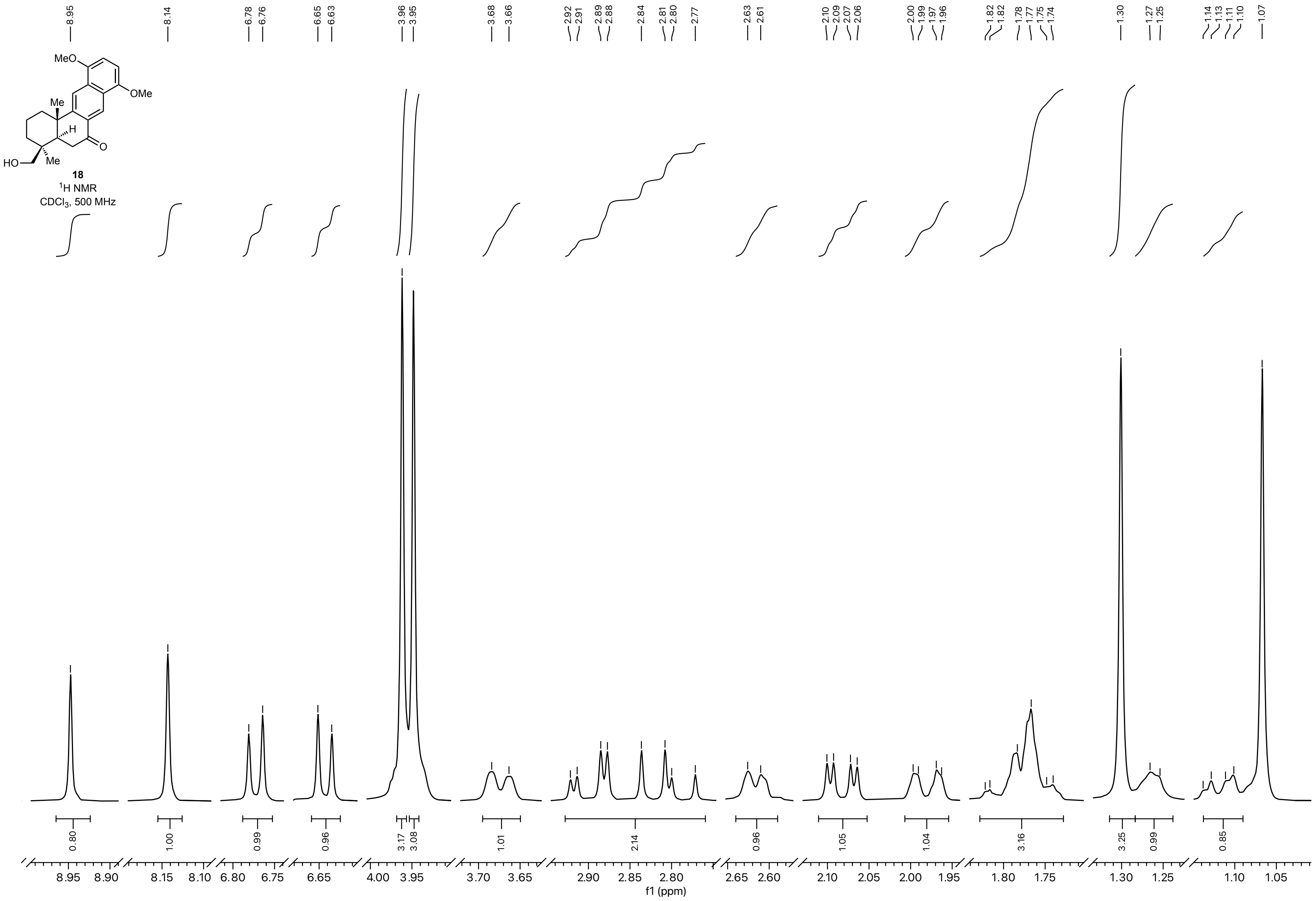

MeO
OMe
Me
H
O
HO
Me
18
^{1}H NMR
$CDCl_3$, 500 MHz
f1 (ppm)

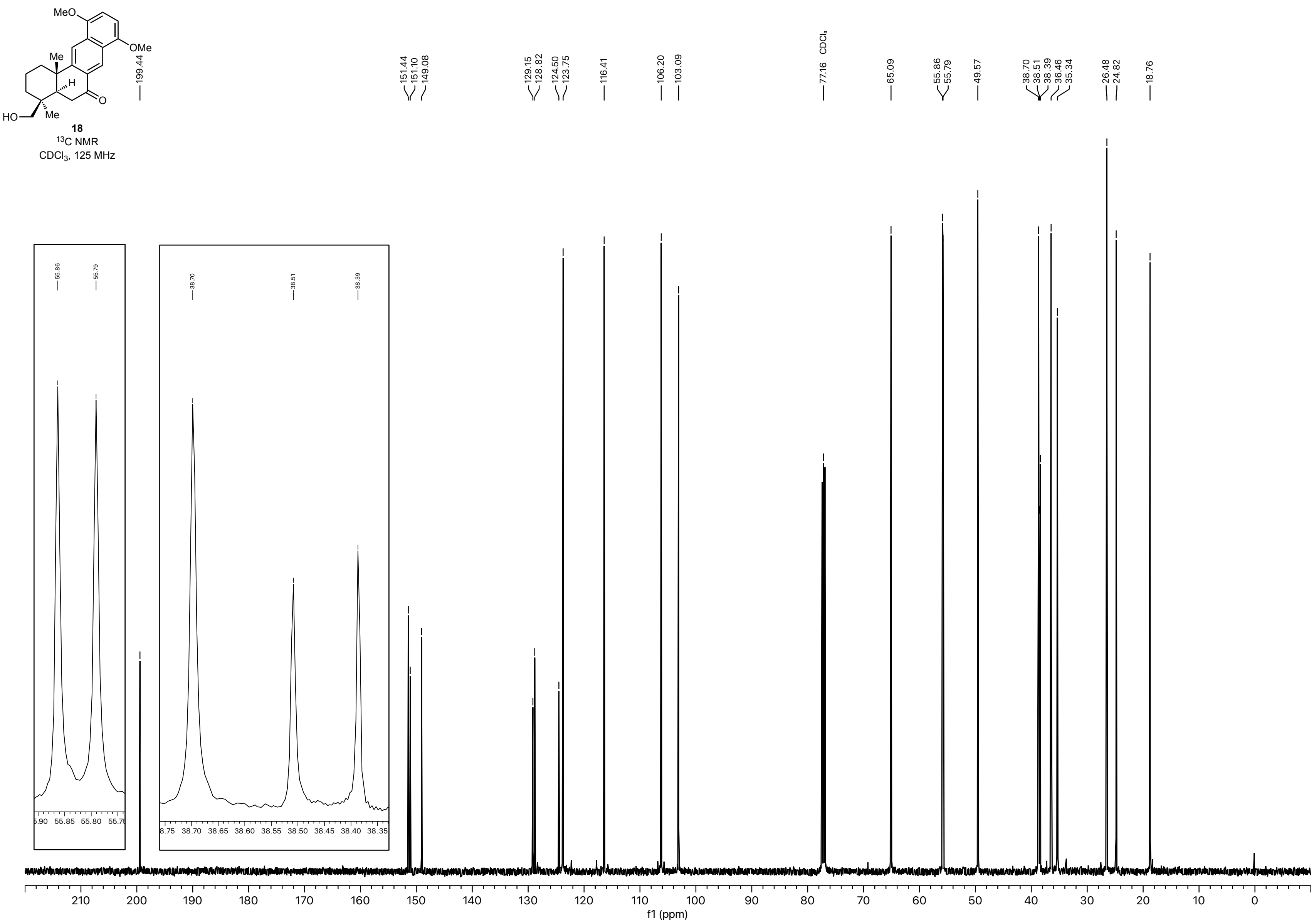
MeO
OMe
Me
H
O
HO
Me
18
^{13}C NMR
$CDCl_3$, 125 MHz
199.44
151.44
151.10
149.08
129.15
128.82
124.50
123.75
116.41
106.20
103.09
77.16 $CDCl_3$
65.09
55.86
55.79
49.57
38.70
38.51
38.39
36.46
35.34
26.48
24.82
18.76
f1 (ppm)

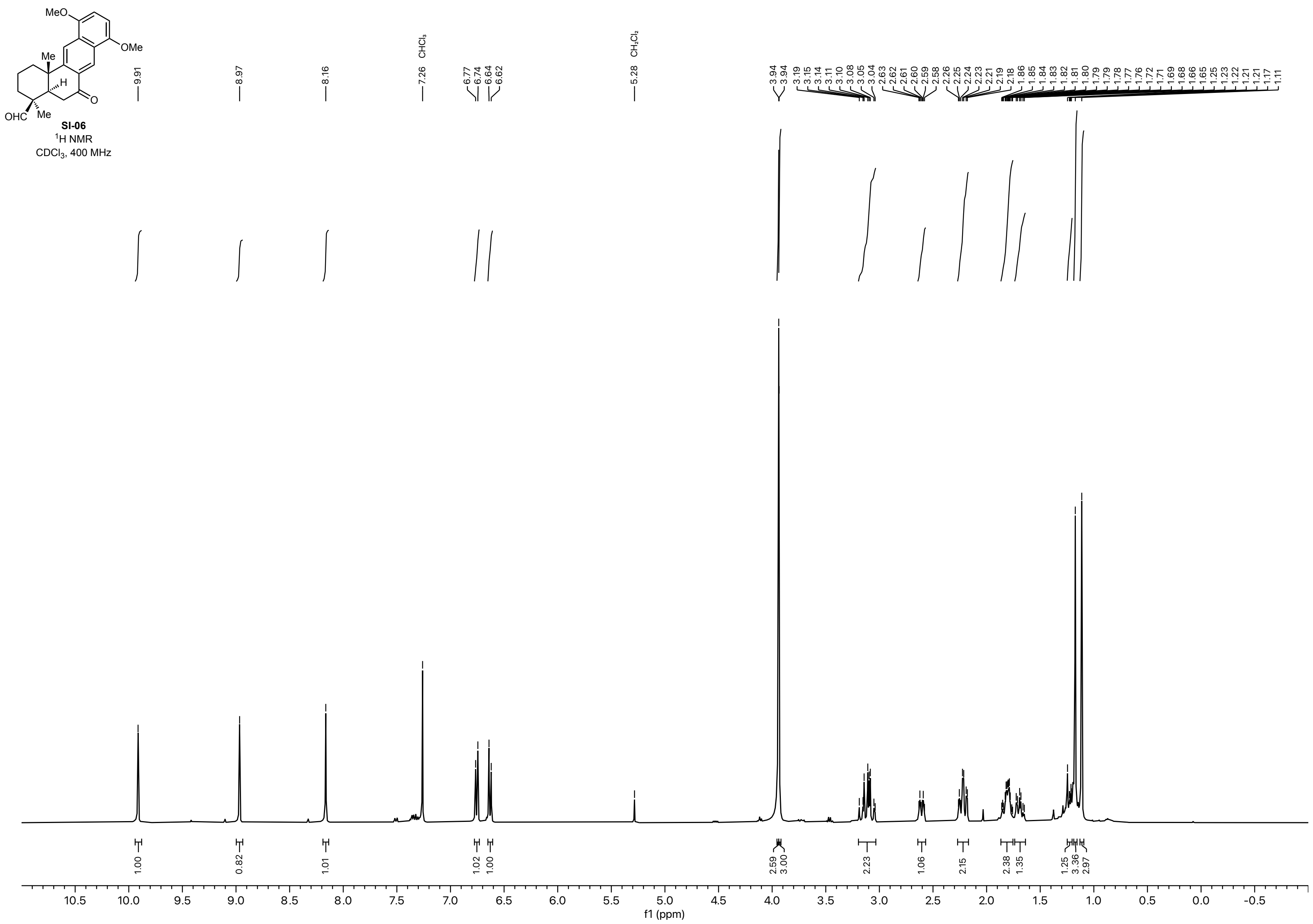
MeO
OMe
Me
H
O
OHC
Me
SI-06
1H NMR
CDCl3, 400 MHz
9.91
8.97
8.16
7.26 CHCl3
6.77
6.74
6.64
6.62
5.28 CH2Cl2
3.94
3.94
1.00
0.82
1.01
1.02
1.00
2.59
3.00
2.23
1.06
2.15
2.38
1.35
1.25
3.36
2.97
f1 (ppm)

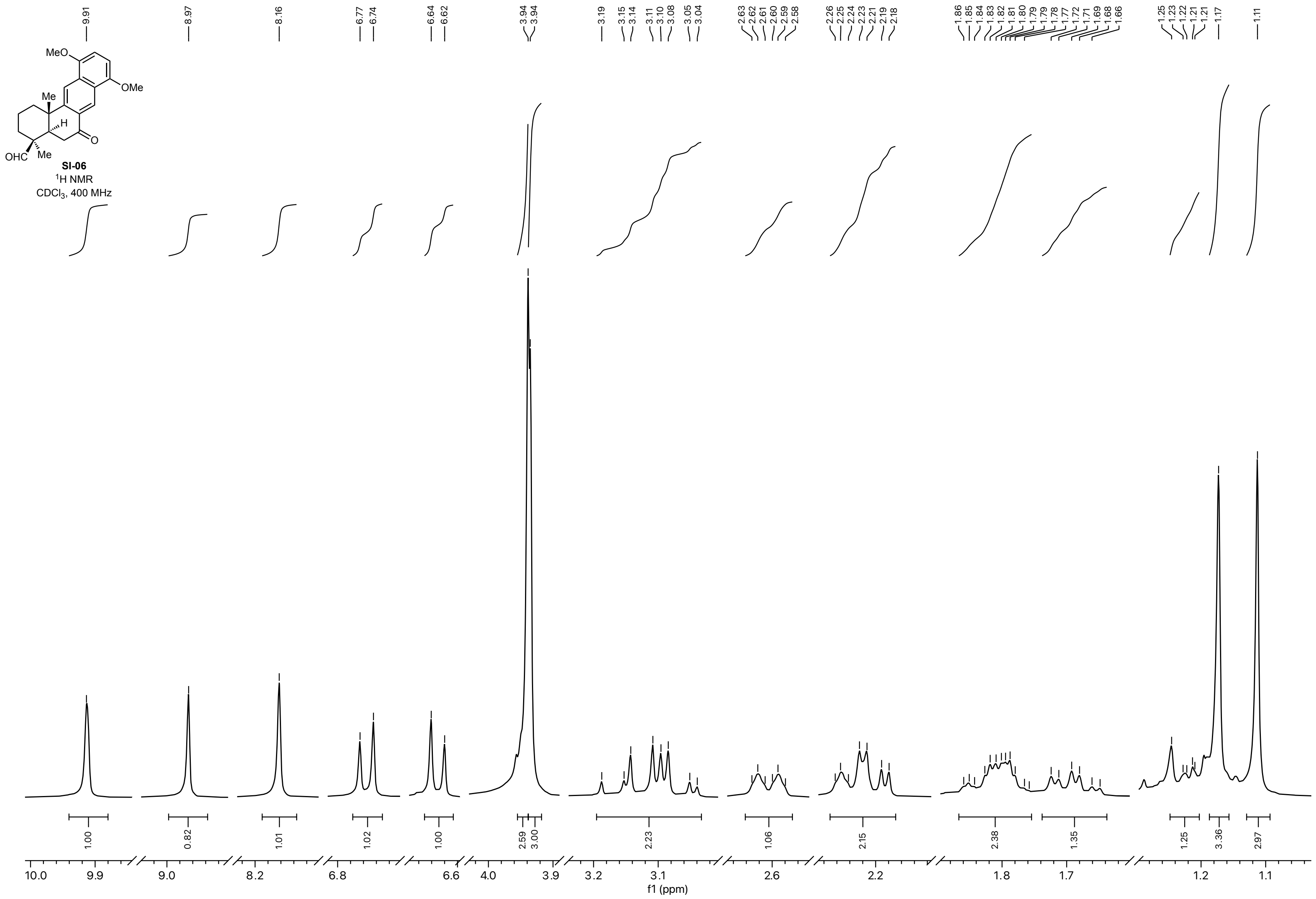
SI-06
¹H NMR
CDCl₃, 400 MHz
f1 (ppm)

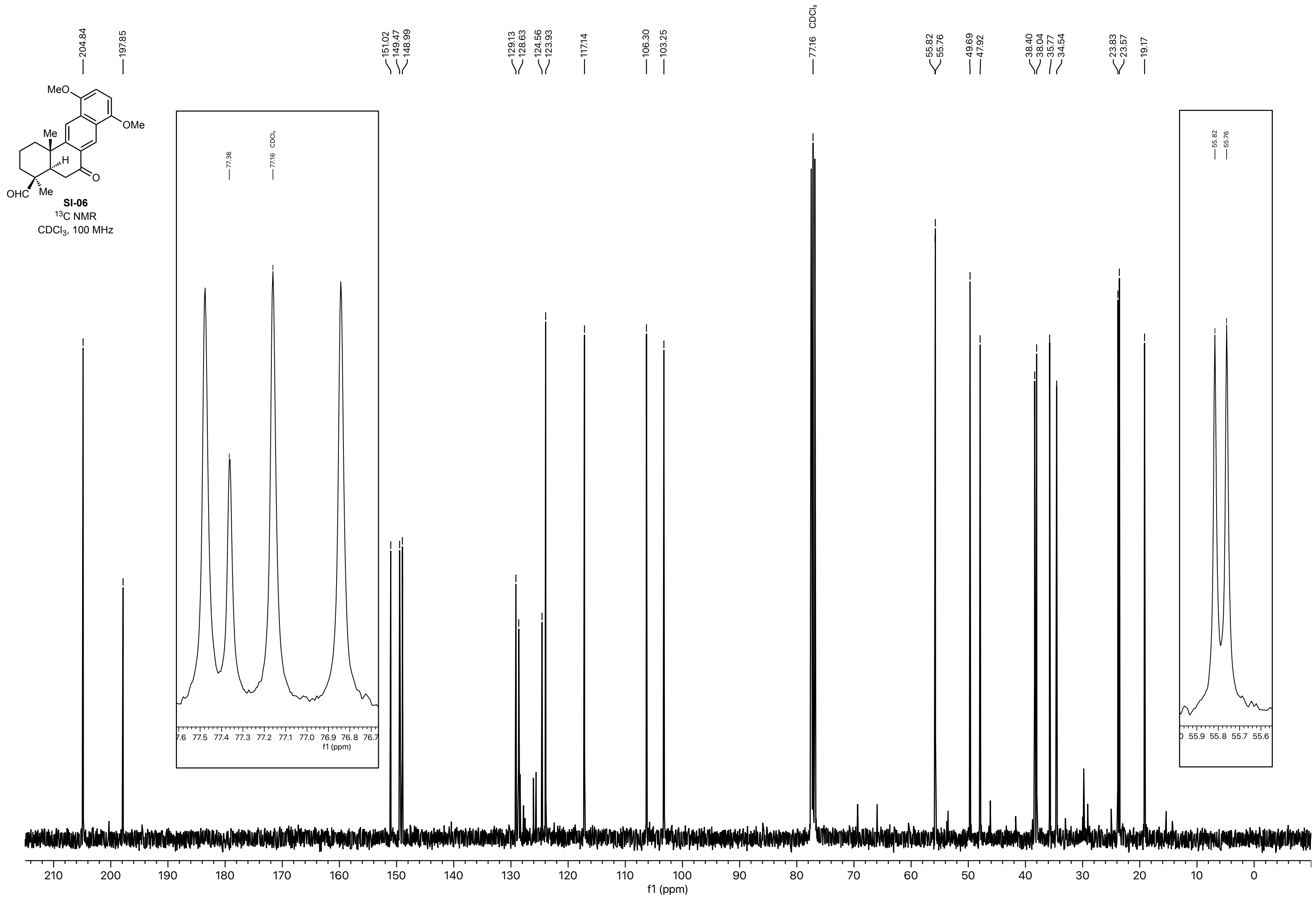

SI-06
^{13}C NMR
$CDCl_3$, 100 MHz

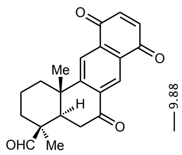

(+)-3: *ent*-neopetrosiquinone A
^1H NMR
CD_2Cl_2, 500 MHz

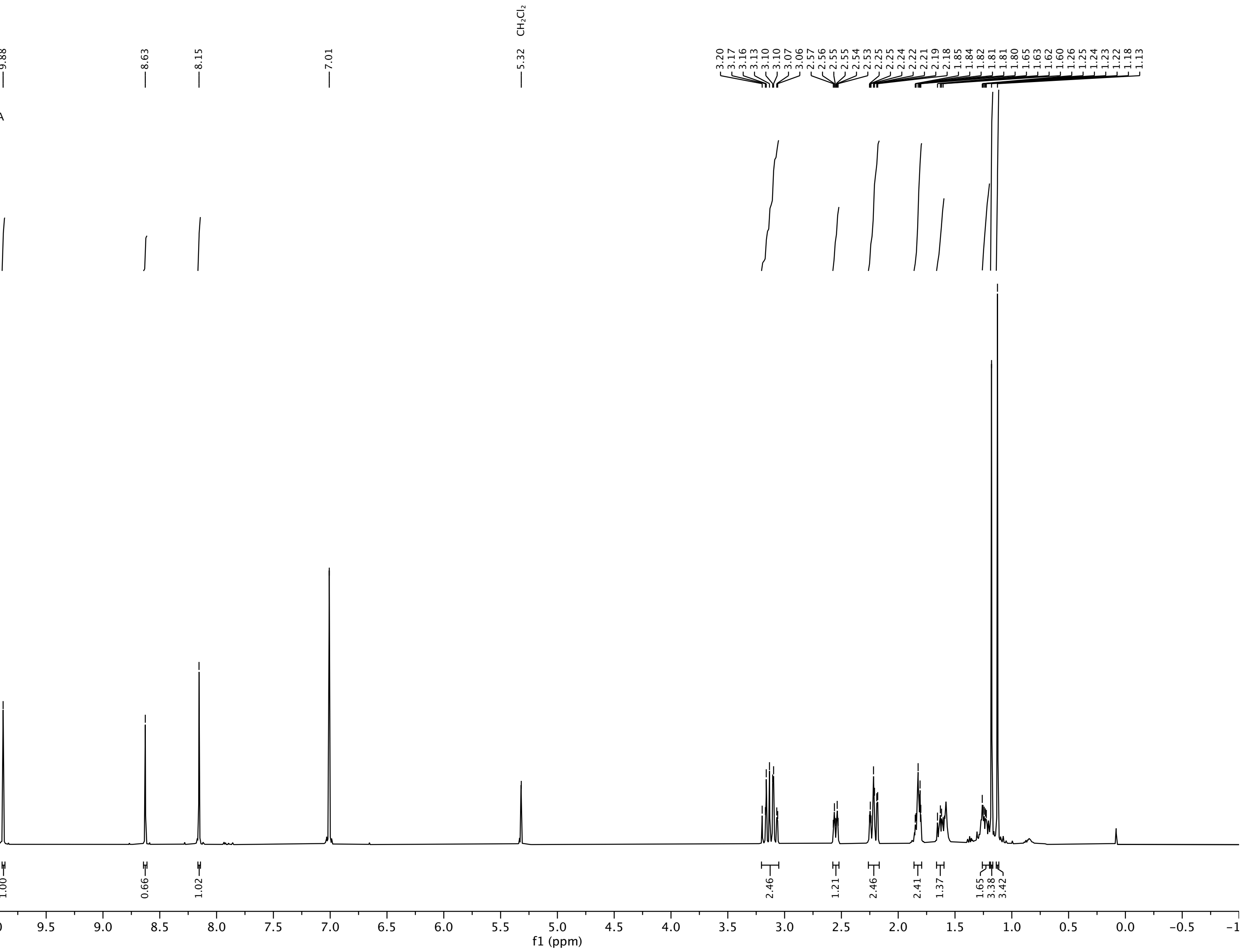

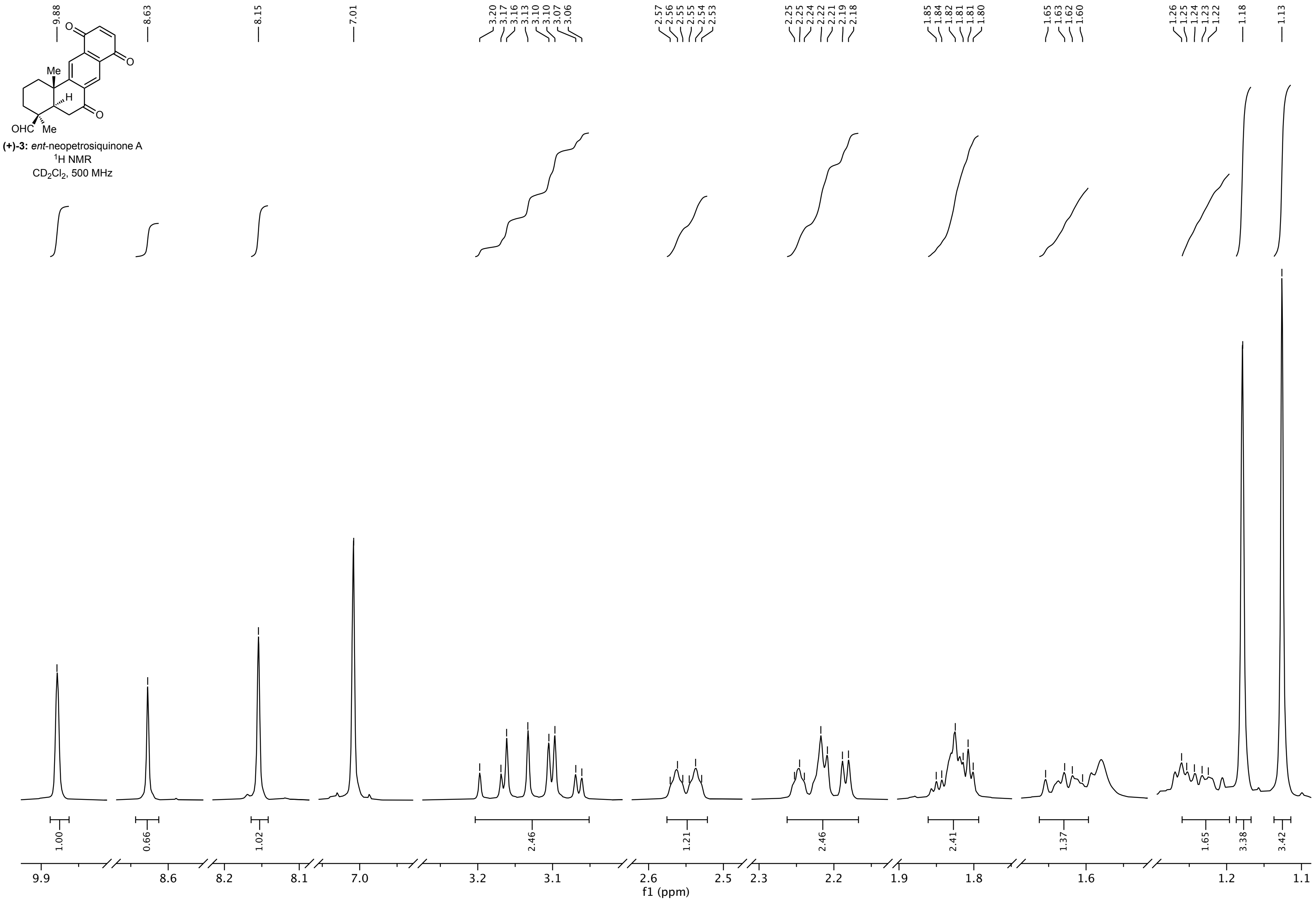
O
O
Me
H
O
OHC
Me
(+)-3: ent-neopetrosiquinone A
1H NMR
CD2Cl2, 500 MHz
9.88
8.63
8.15
7.01
3.20 3.17 3.16 3.13 3.10 3.10 3.07 3.06
2.57 2.56 2.55 2.55 2.54 2.53
2.25 2.25 2.24 2.22 2.21 2.19 2.18
1.85 1.84 1.82 1.81 1.81 1.80
1.65 1.63 1.62 1.60
1.26 1.25 1.24 1.23 1.22
1.18
1.13
1.00
0.66
1.02
2.46
1.21
2.46
2.41
1.37
1.65
3.38
3.42
9.9
8.6
8.2
8.1
7.0
3.2
3.1
2.6
2.5
2.3
2.2
1.9
1.8
1.6
1.2
1.1
f1 (ppm)

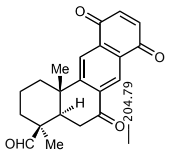

(+)-3: *ent*-neopetrosiquinone A
^{13}C NMR
CD_2Cl_2, 125 MHz

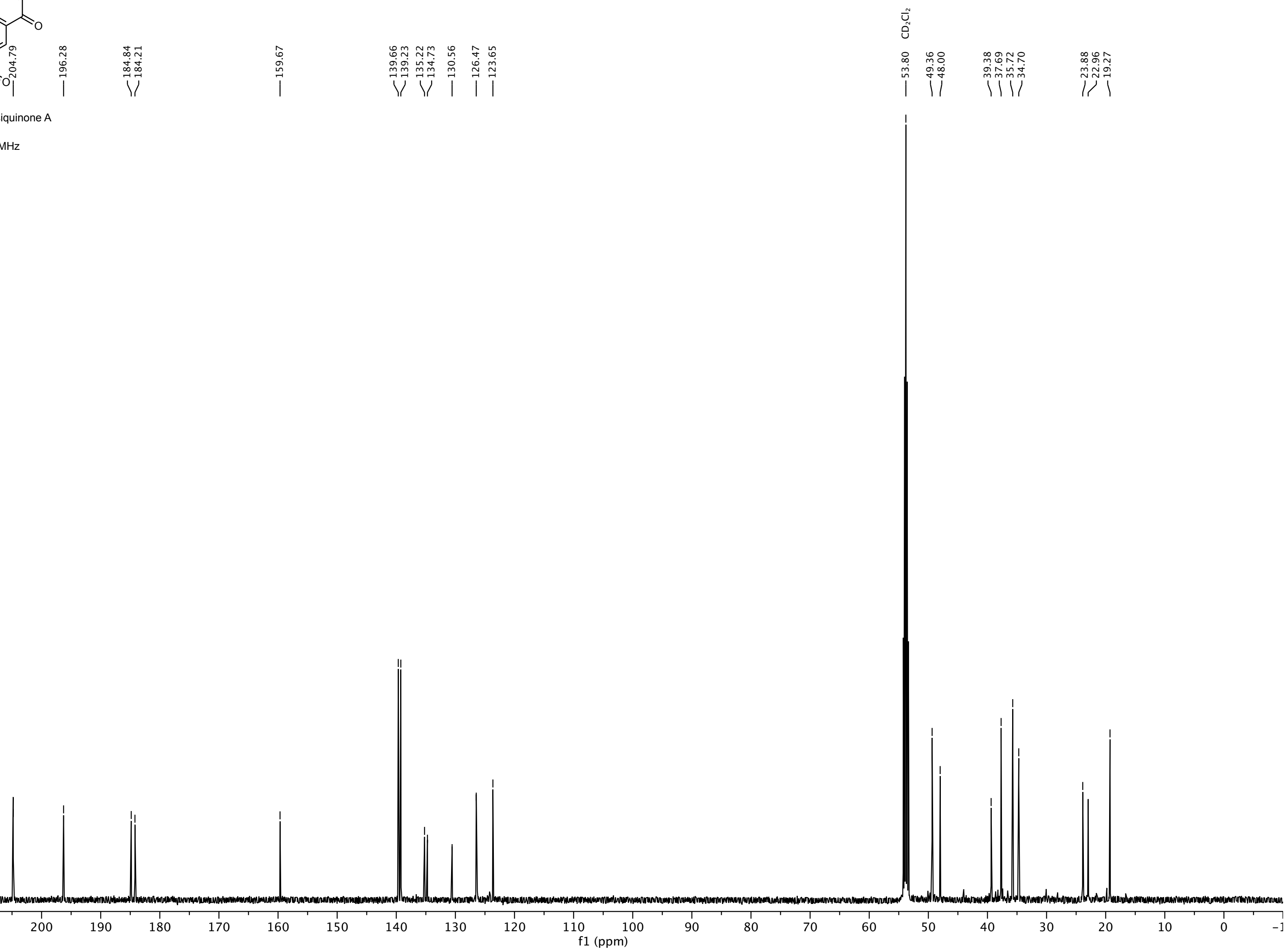

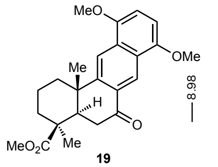

19

^1H NMR

$CDCl_3$, 500 MHz

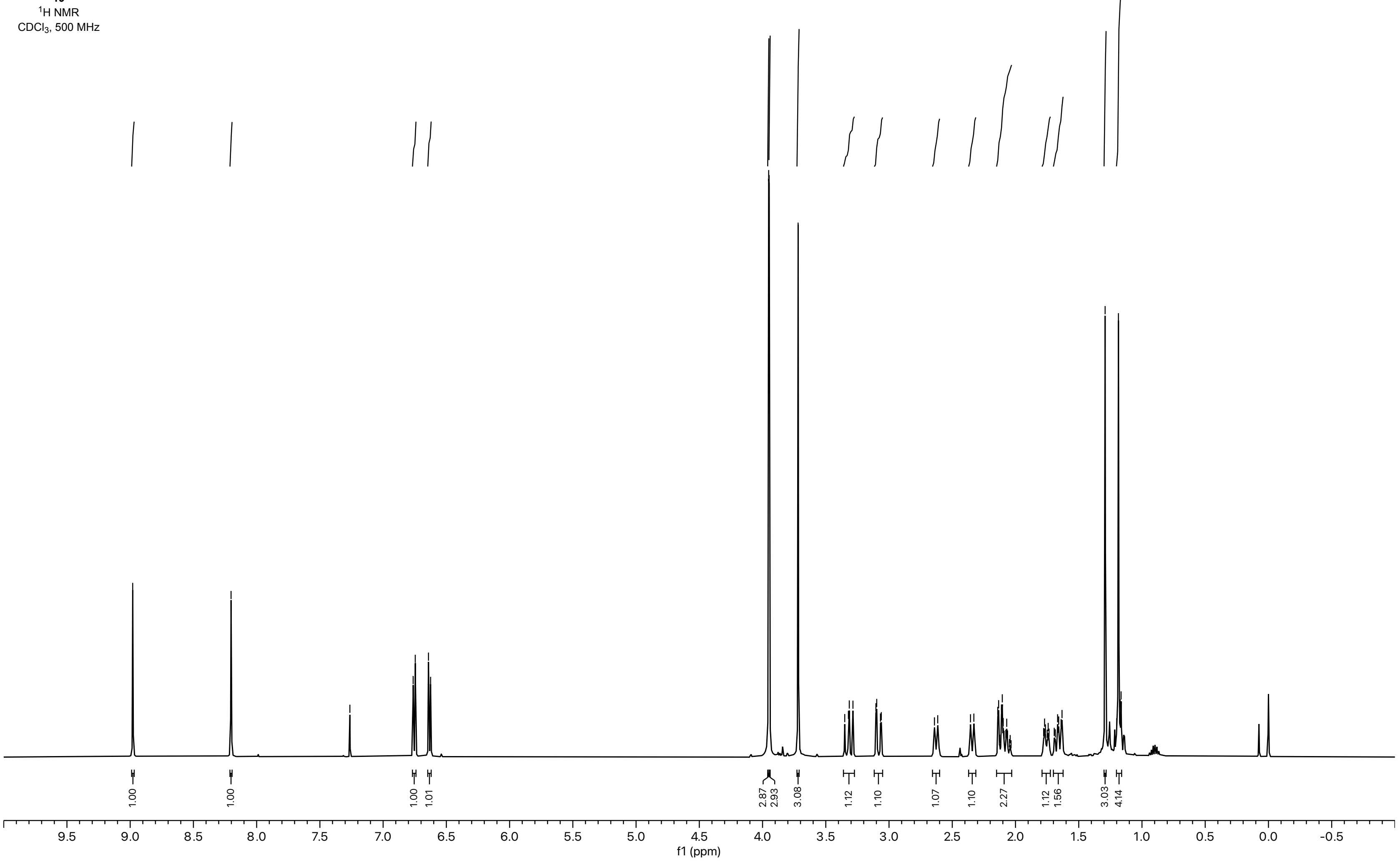

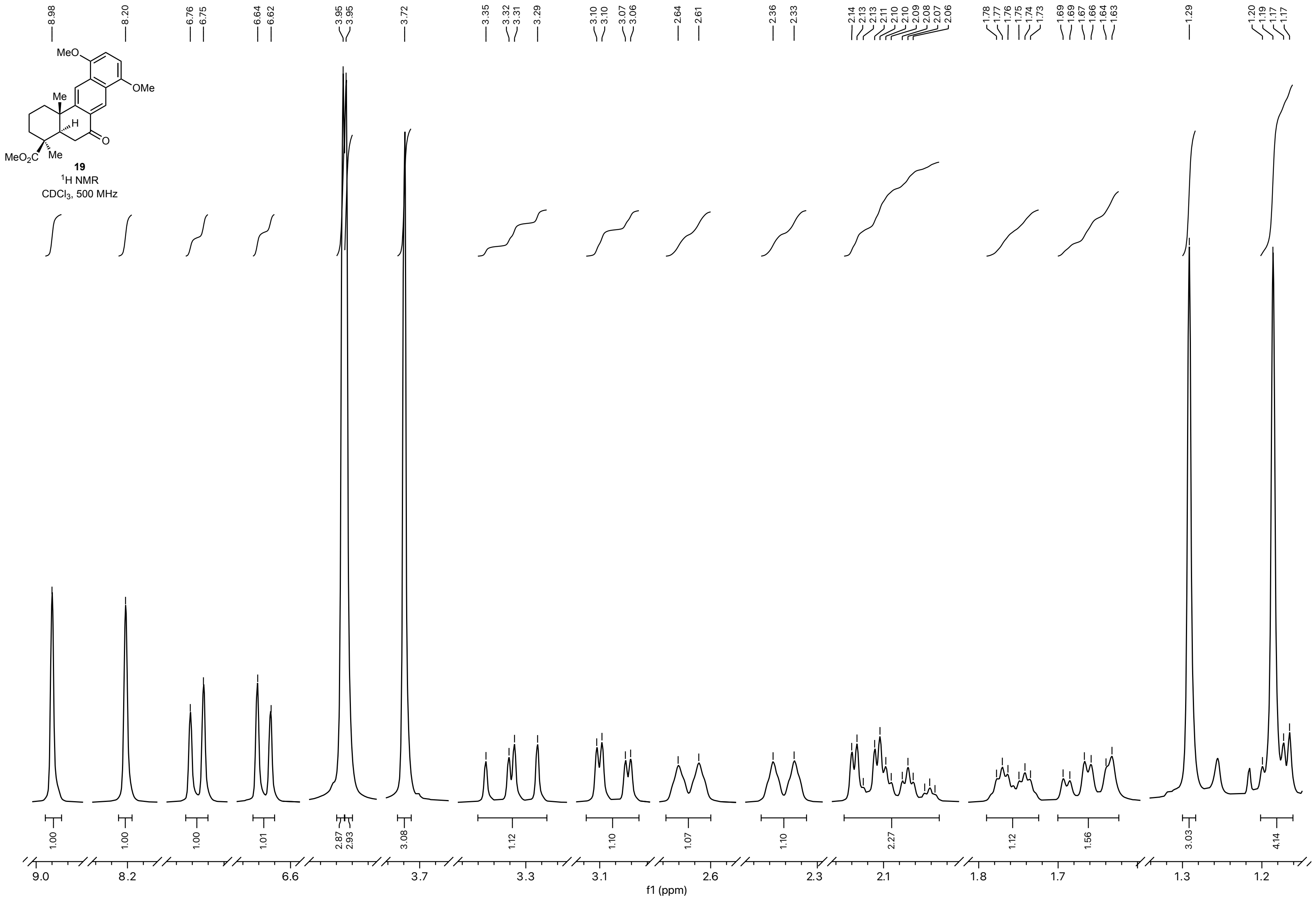
MeO
OMe
Me
H
O
MeO2C
Me
19
1H NMR
CDCl3, 500 MHz
8.98
8.20
6.76
6.75
6.64
6.62
3.95
3.95
3.72
3.35
3.32
3.31
3.29
3.10
3.10
3.07
3.06
2.64
2.61
2.36
2.33
2.14
2.13
2.13
2.11
2.10
2.10
2.09
2.08
2.07
2.06
1.78
1.77
1.76
1.75
1.74
1.73
1.69
1.69
1.67
1.66
1.64
1.63
1.29
1.20
1.19
1.17
1.17
1.00
1.00
1.00
1.01
2.87
2.93
3.08
1.12
1.10
1.07
1.10
2.27
1.12
1.56
3.03
4.14
9.0
8.2
6.6
3.7
3.3
3.1
2.6
2.3
2.1
1.8
1.7
1.3
1.2
f1 (ppm)

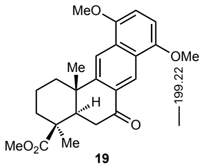

19

^{13}C NMR

$CDCl_3$, 125 MHz

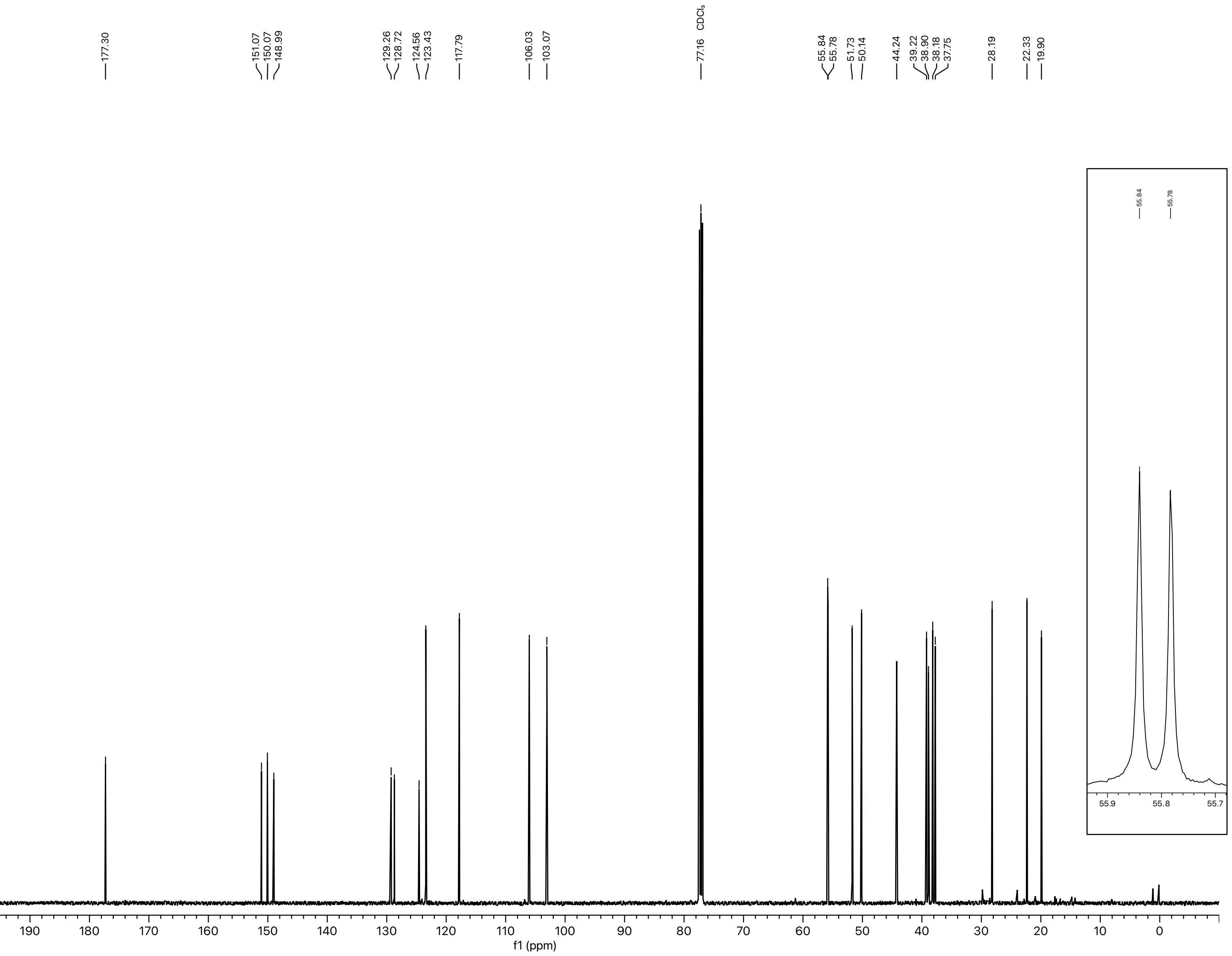

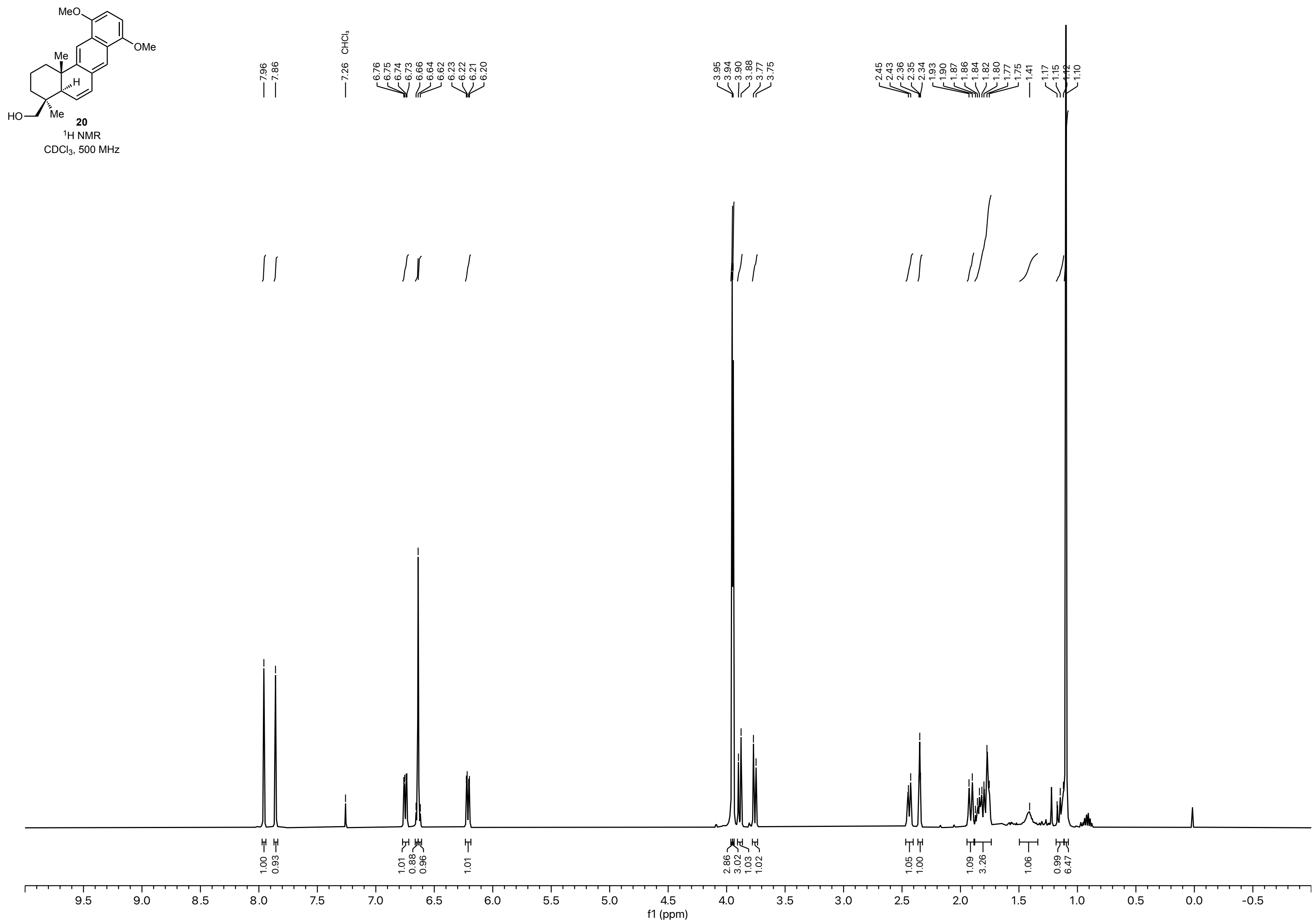
MeO
OMe
Me
H
HO
Me
20
^{1}H NMR
CDCl$_3$, 500 MHz
7.96
7.86
7.26 CHCl$_3$
6.76
6.75
6.74
6.73
6.66
6.64
6.62
6.23
6.22
6.21
6.20
3.95
3.94
3.90
3.88
3.77
3.75
2.45
2.43
2.36
2.35
2.34
1.93
1.90
1.87
1.86
1.84
1.82
1.80
1.77
1.75
1.41
1.17
1.15
1.12
1.10
1.00
0.93
1.01
0.88
0.96
1.01
2.86
3.02
1.03
1.02
1.05
1.00
1.09
3.26
1.06
0.99
6.47
9.5
9.0
8.5
8.0
7.5
7.0
6.5
6.0
5.5
5.0
4.5
4.0
3.5
3.0
2.5
2.0
1.5
1.0
0.5
0.0
-0.5
f1 (ppm)

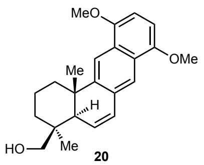

20

^{1}H NMR

$CDCl_3$, 500 MHz

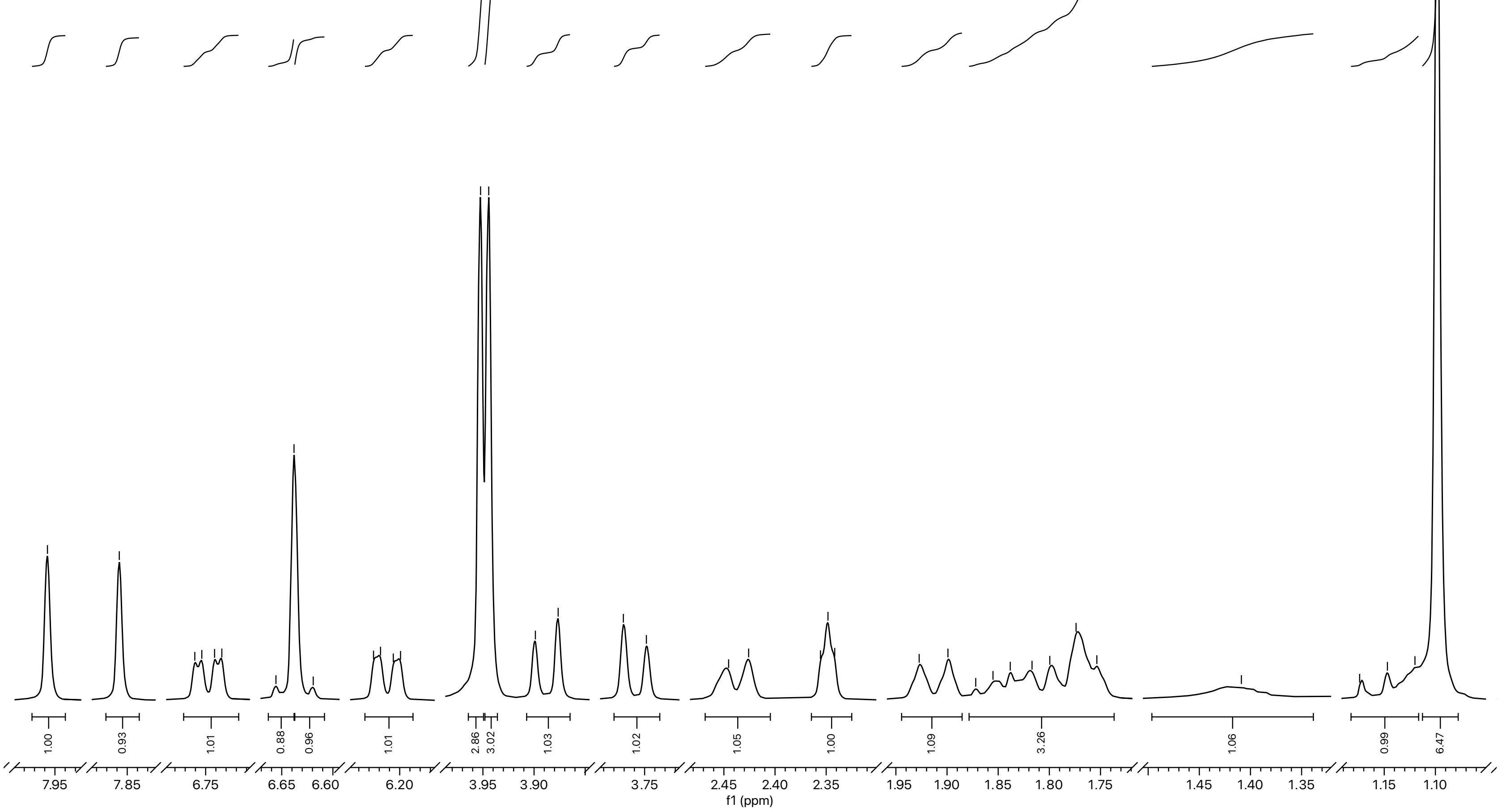

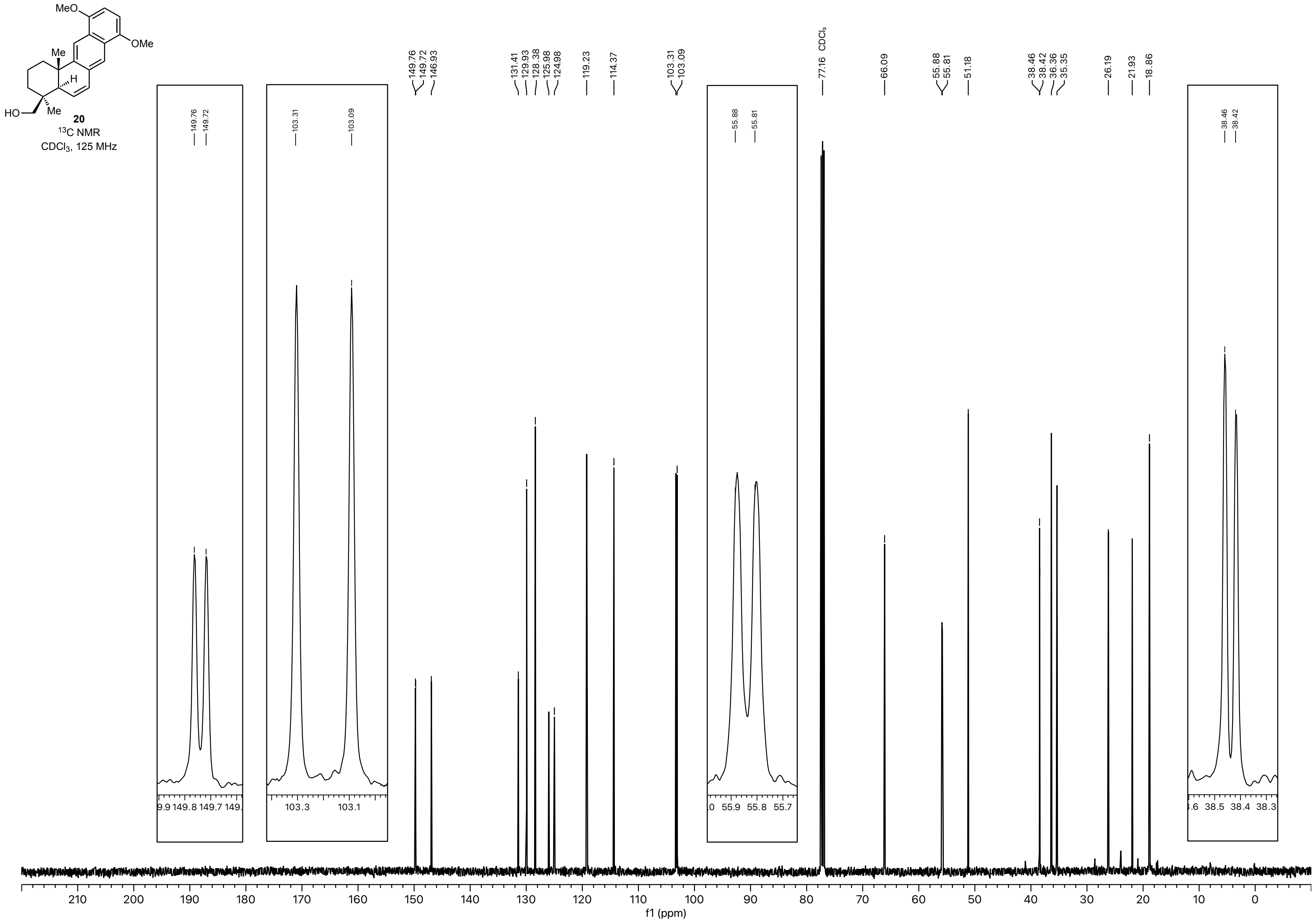

20

^{13}C NMR

$CDCl_3$, 125 MHz

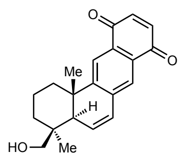

(−)-21: *ent*-neopetrosiquinone B
^{1}H NMR
$CDCl_3$, 500 MHz

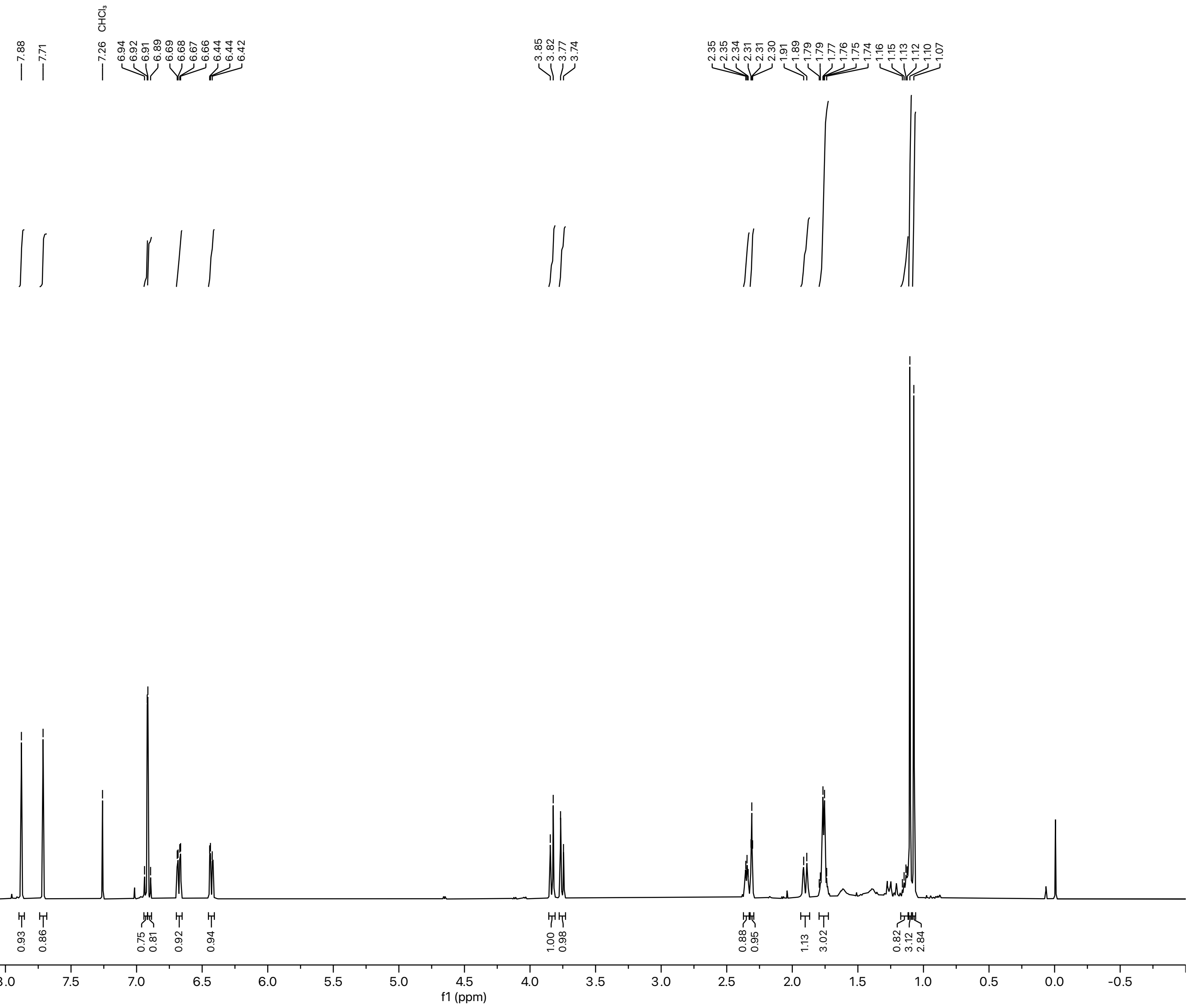

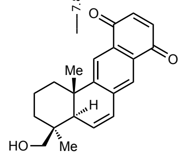

(−)-21: *ent*-neopetrosiquinone B
^{1}H NMR
$CDCl_3$, 500 MHz

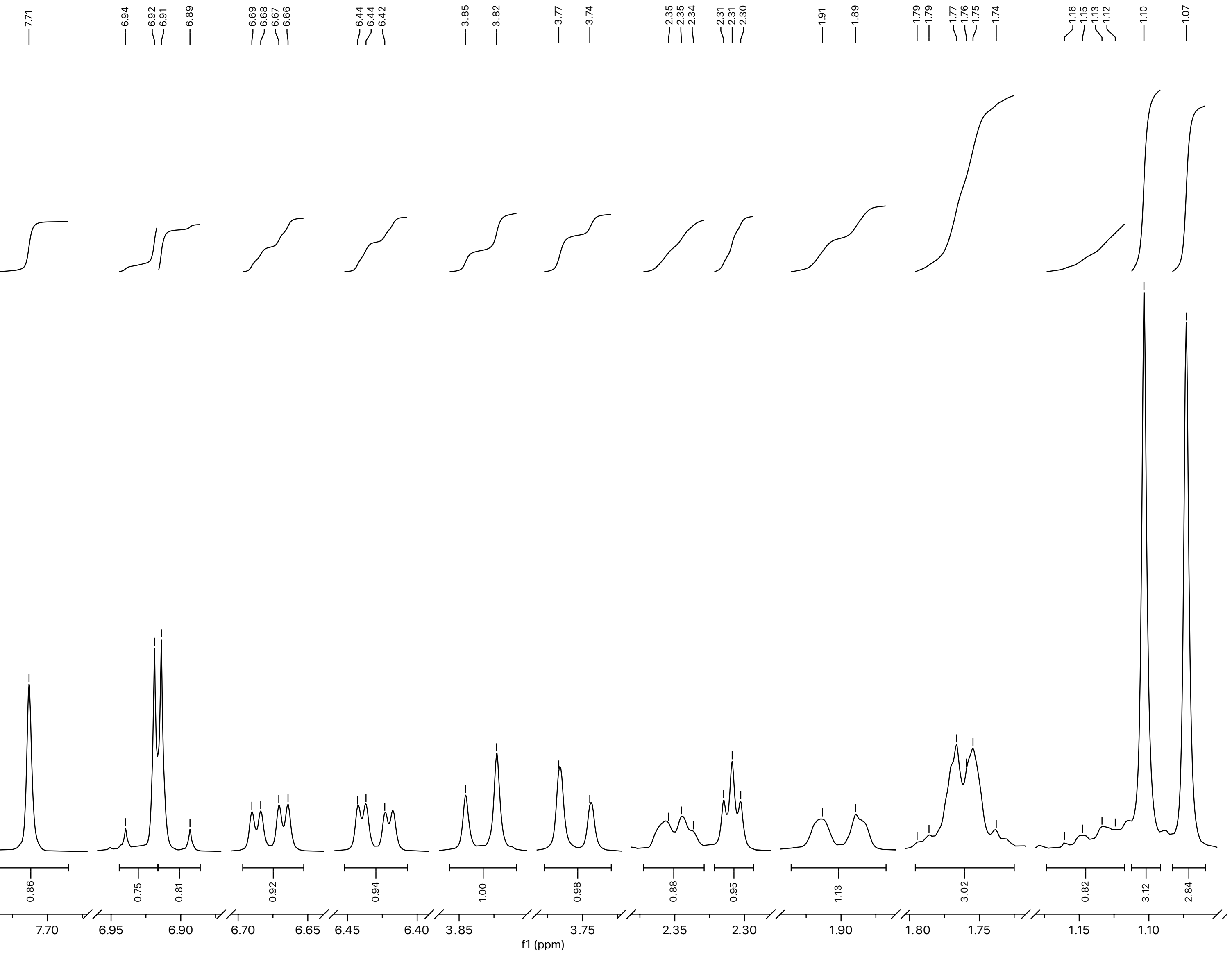

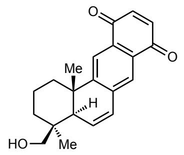

(−)-21: *ent*-neopetrosiquinone B
^{13}C NMR
$CDCl_3$, 125 MHz

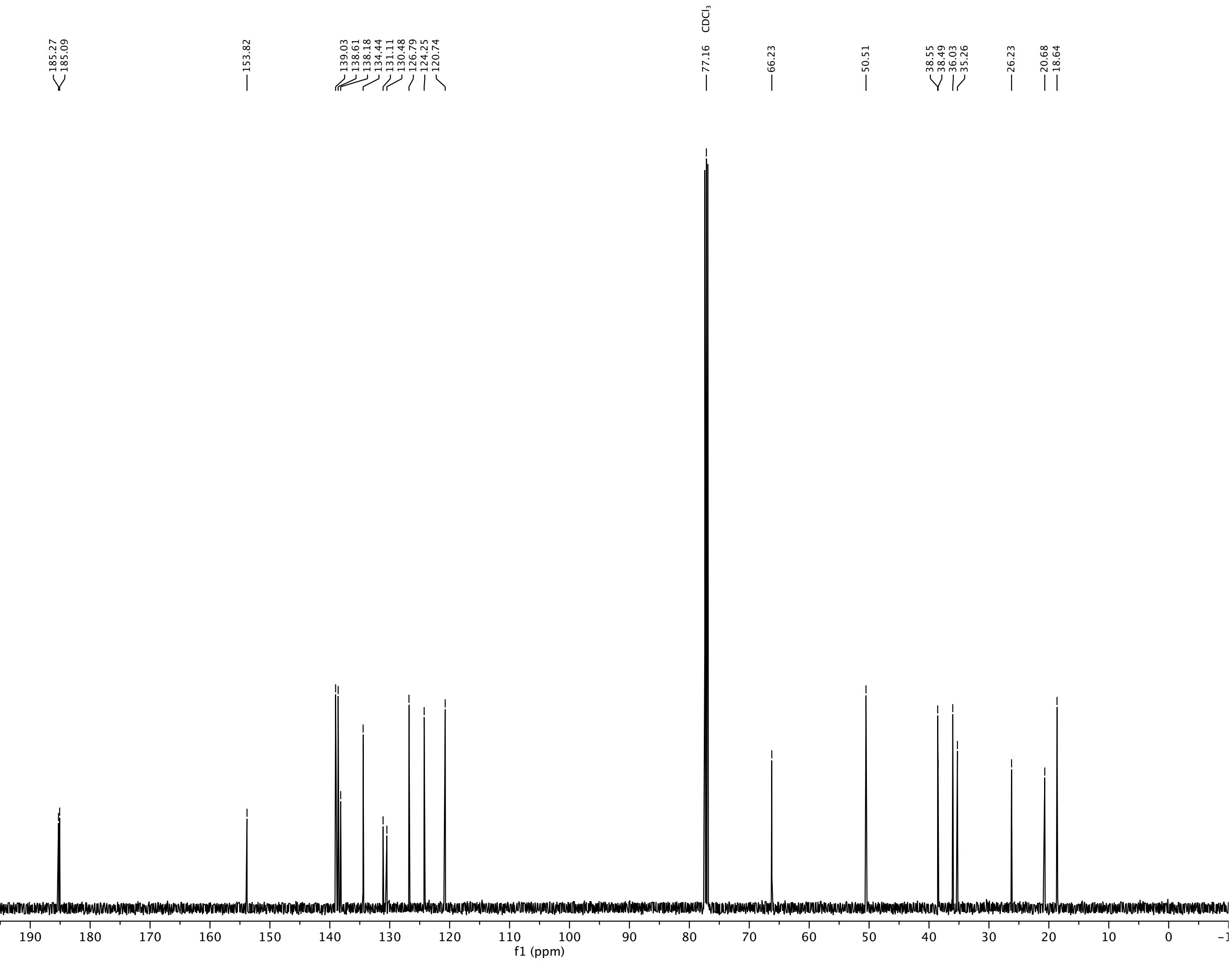

Part 3: Orhalquinone, Xestoquinone, and Halenaquinone

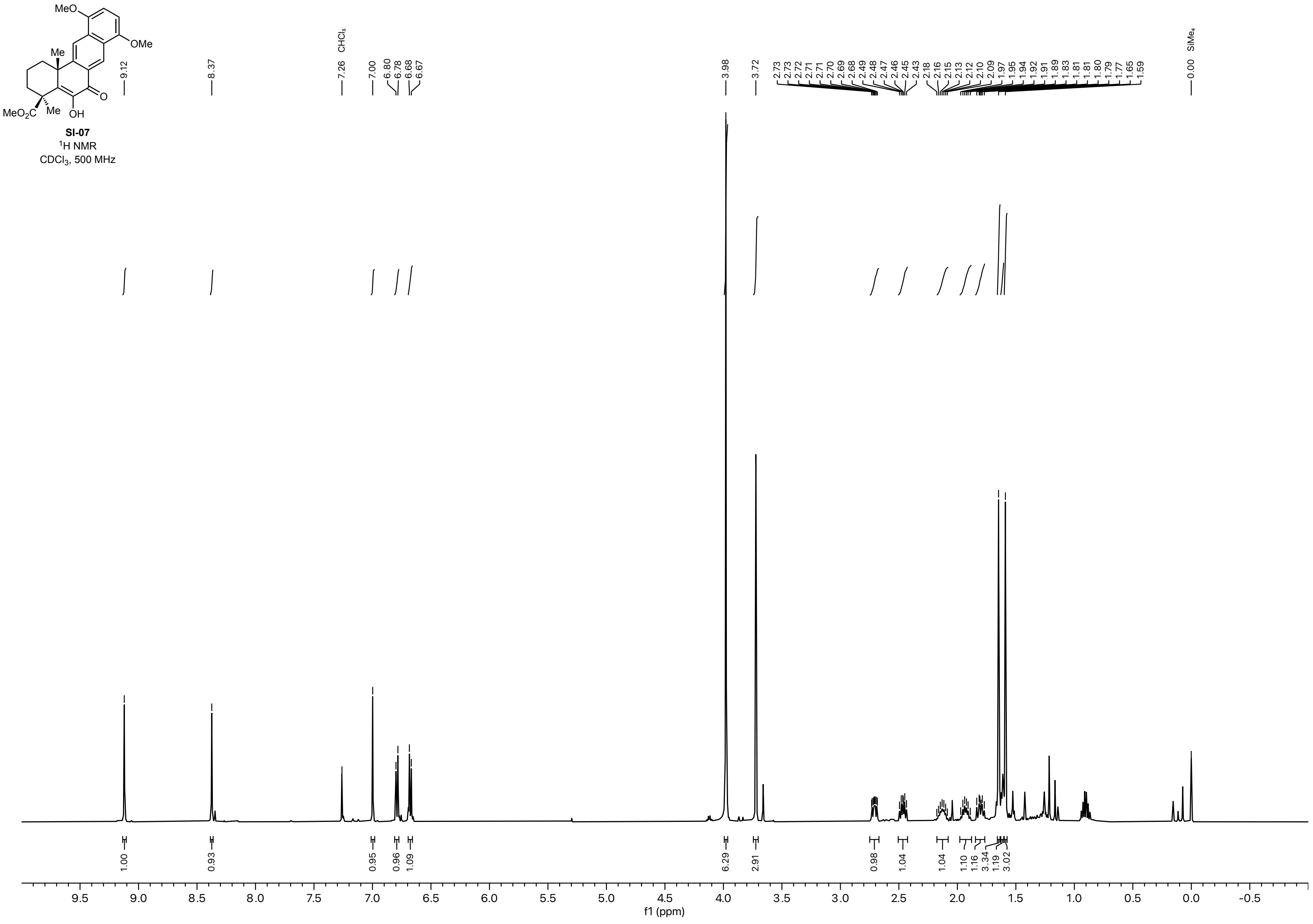

SI-07
^{1}H NMR
$CDCl_3$, 500 MHz

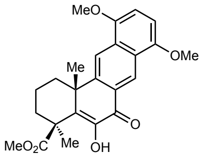

SI-07
^{1}H NMR
$CDCl_3$, 500 MHz

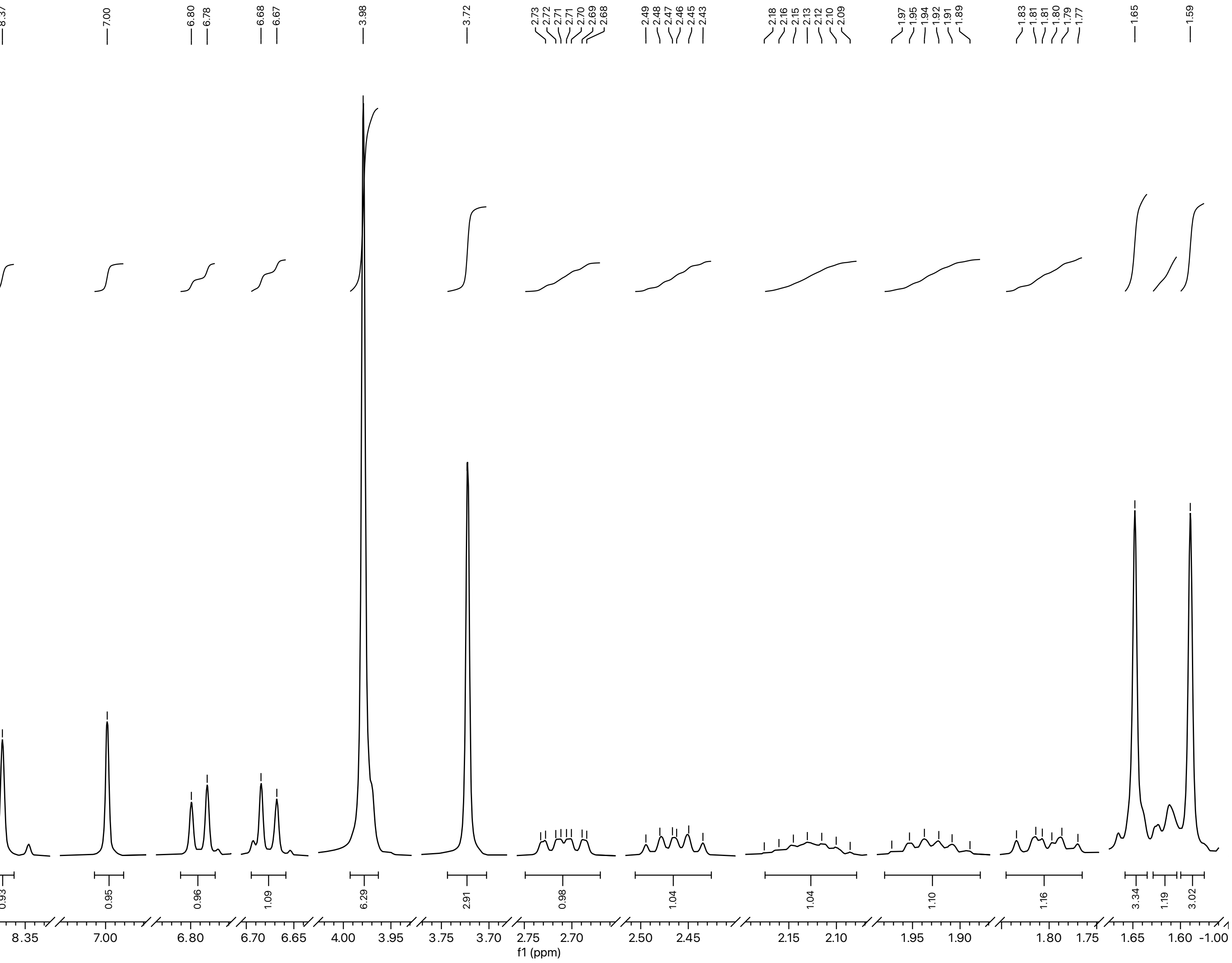

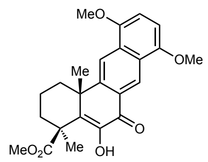

SI-07
^{13}C NMR
$CDCl_3$, 125 MHz

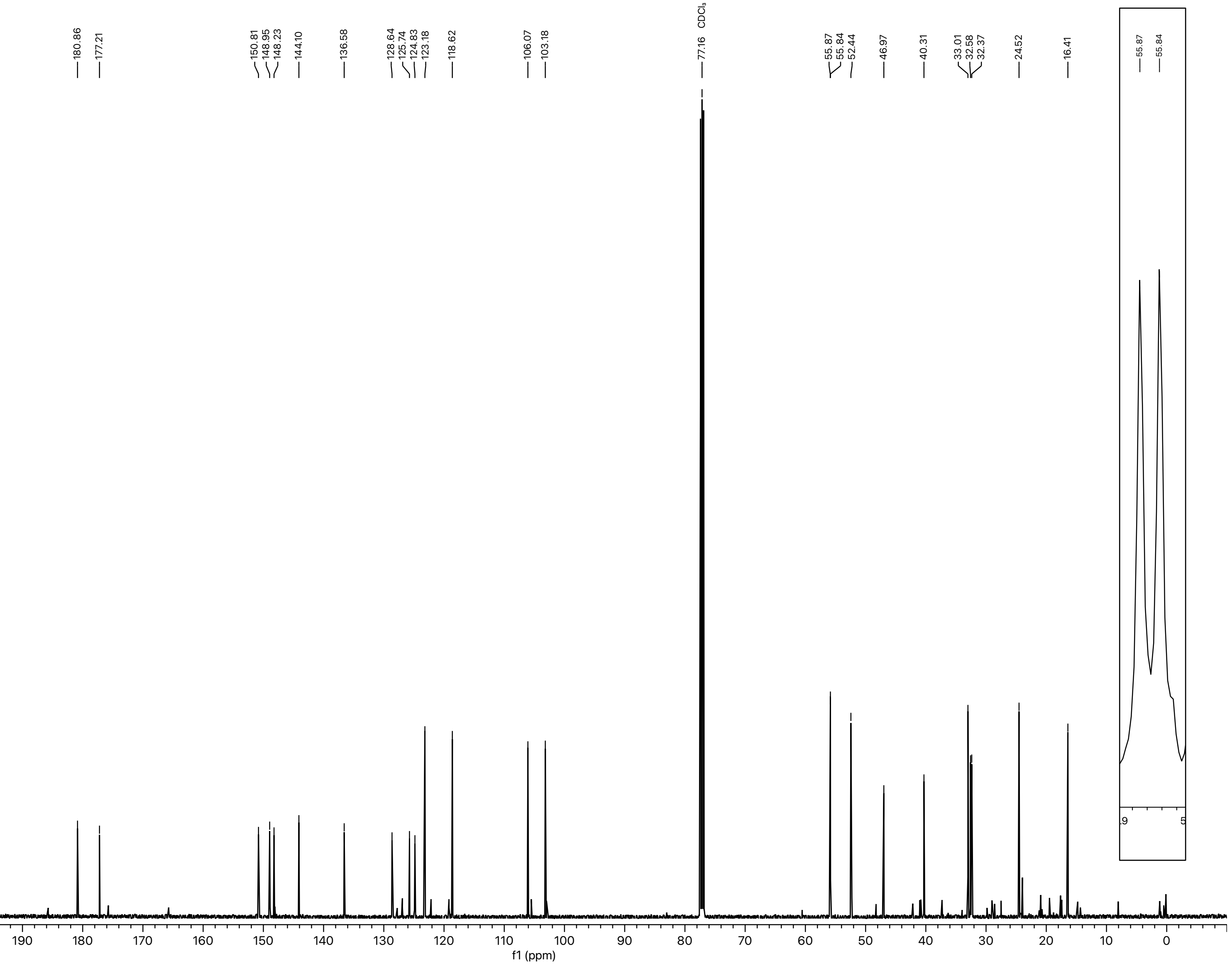

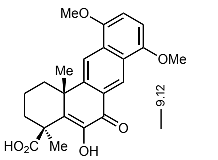

22
^1H NMR
$CDCl_3$, 500 MHz

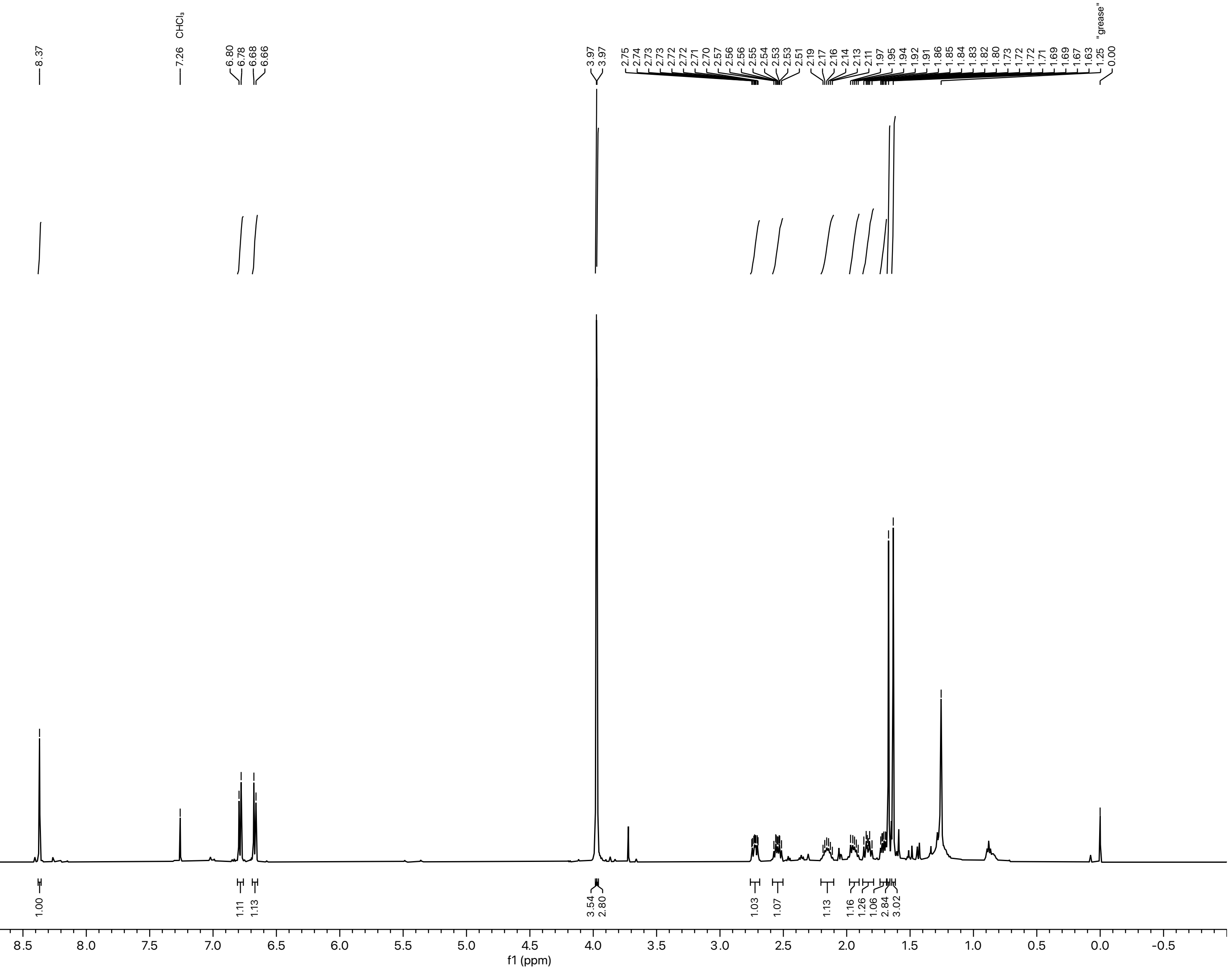

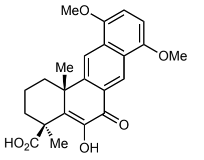

22

^{1}H NMR

$CDCl_3$, 500 MHz

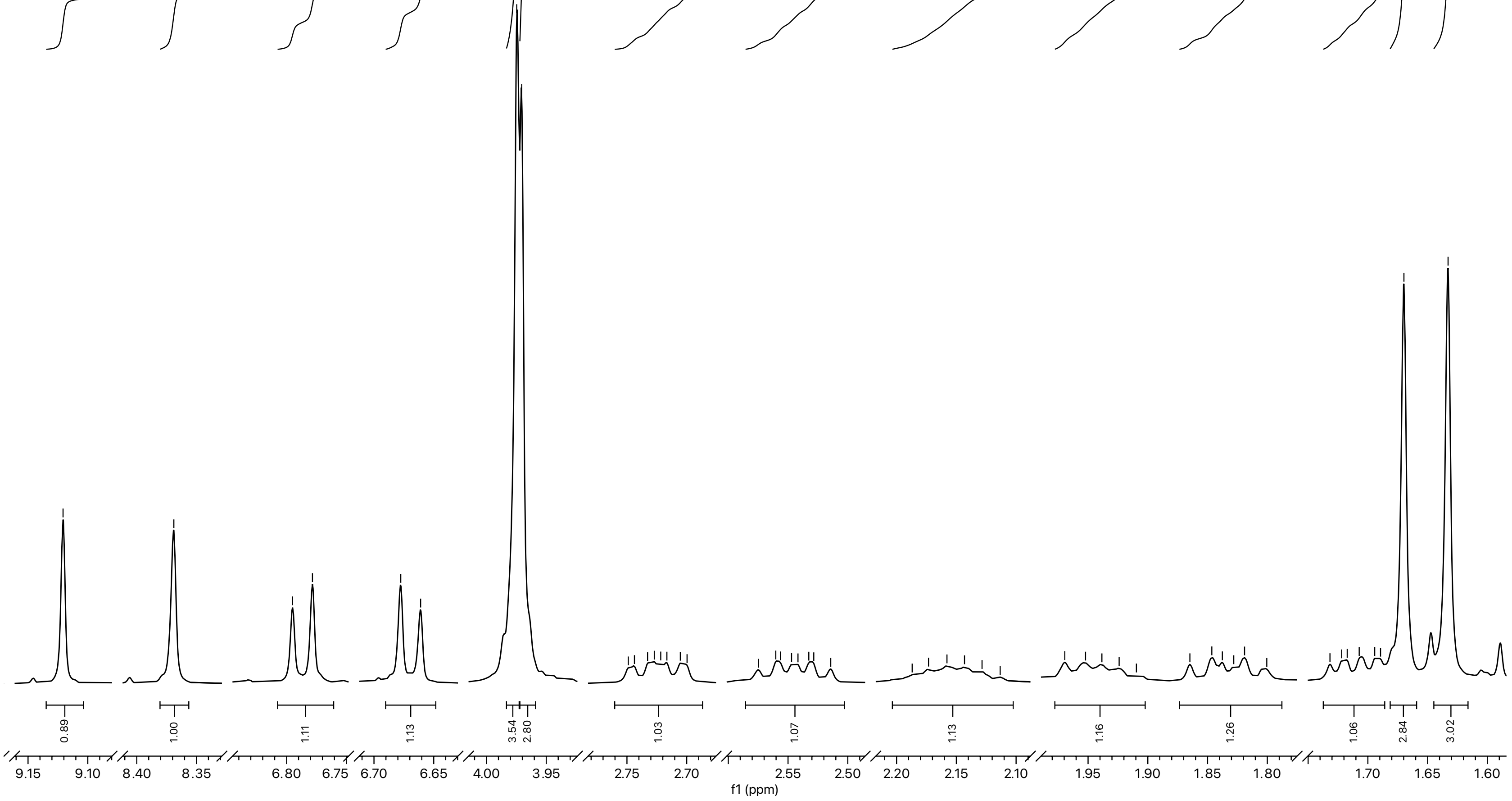

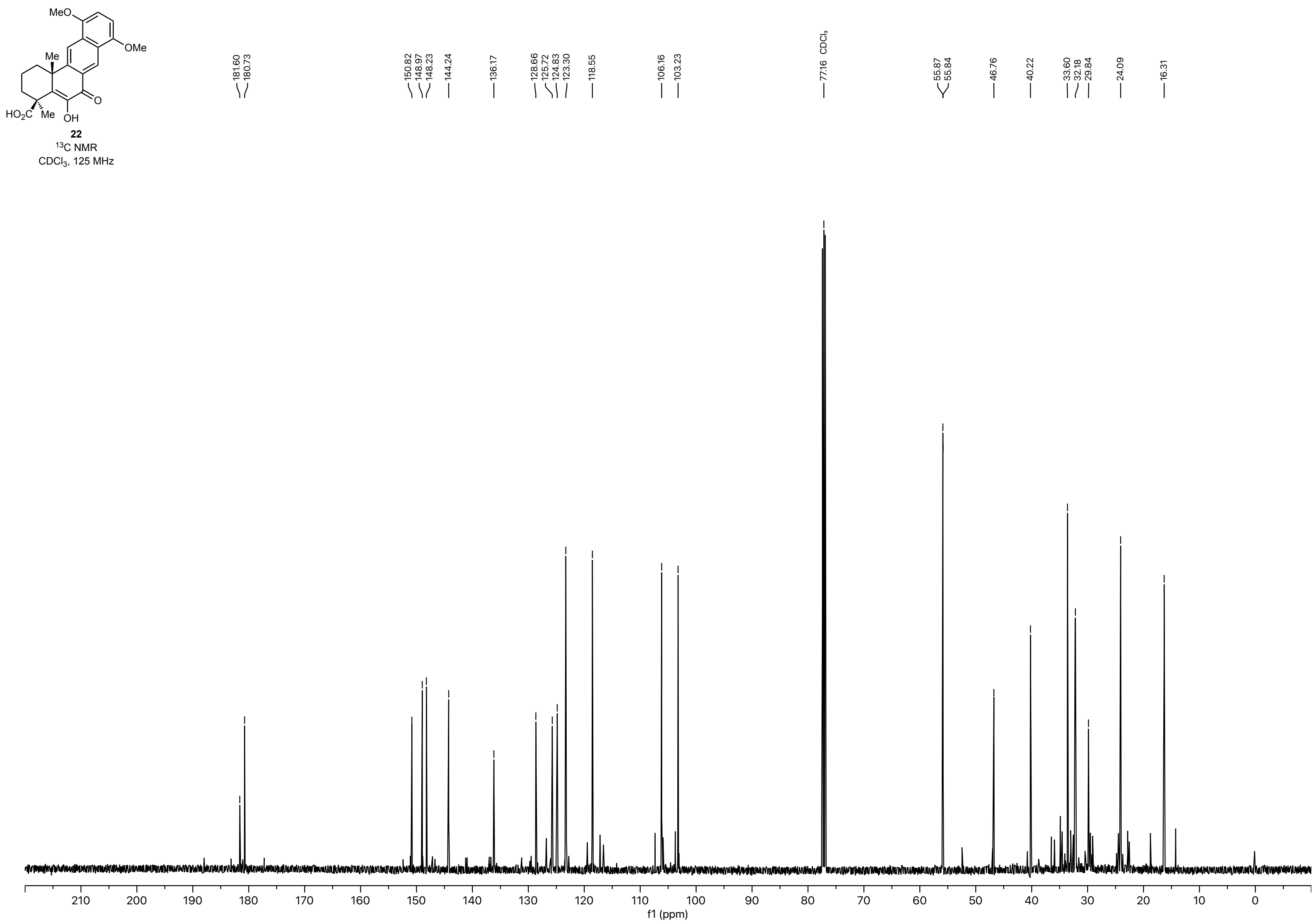

22
^{13}C NMR
$CDCl_3$, 125 MHz

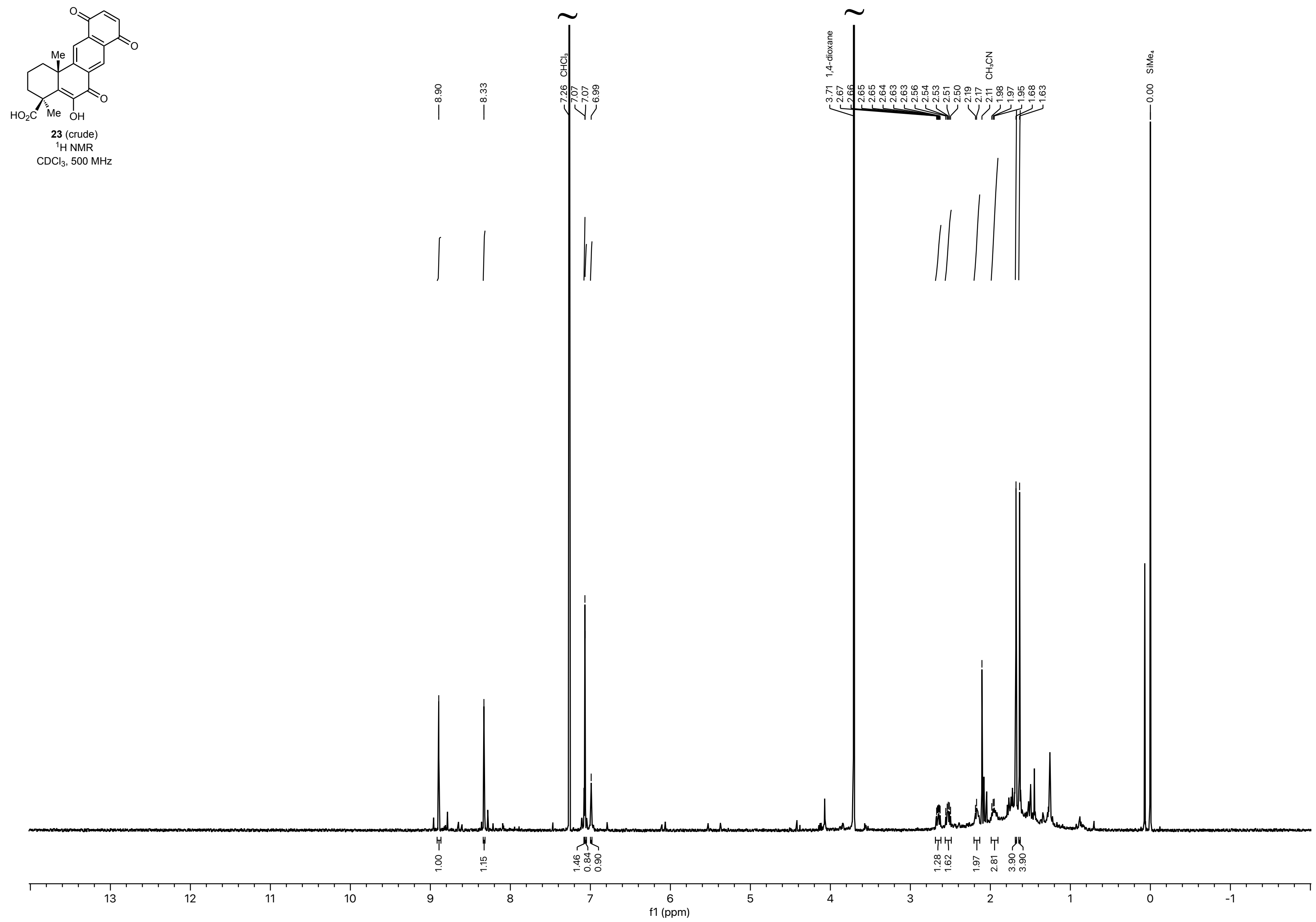

23 (crude)
^{1}H NMR
$CDCl_3$, 500 MHz

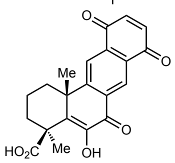

23 (crude)
^1H NMR
$CDCl_3$, 500 MHz

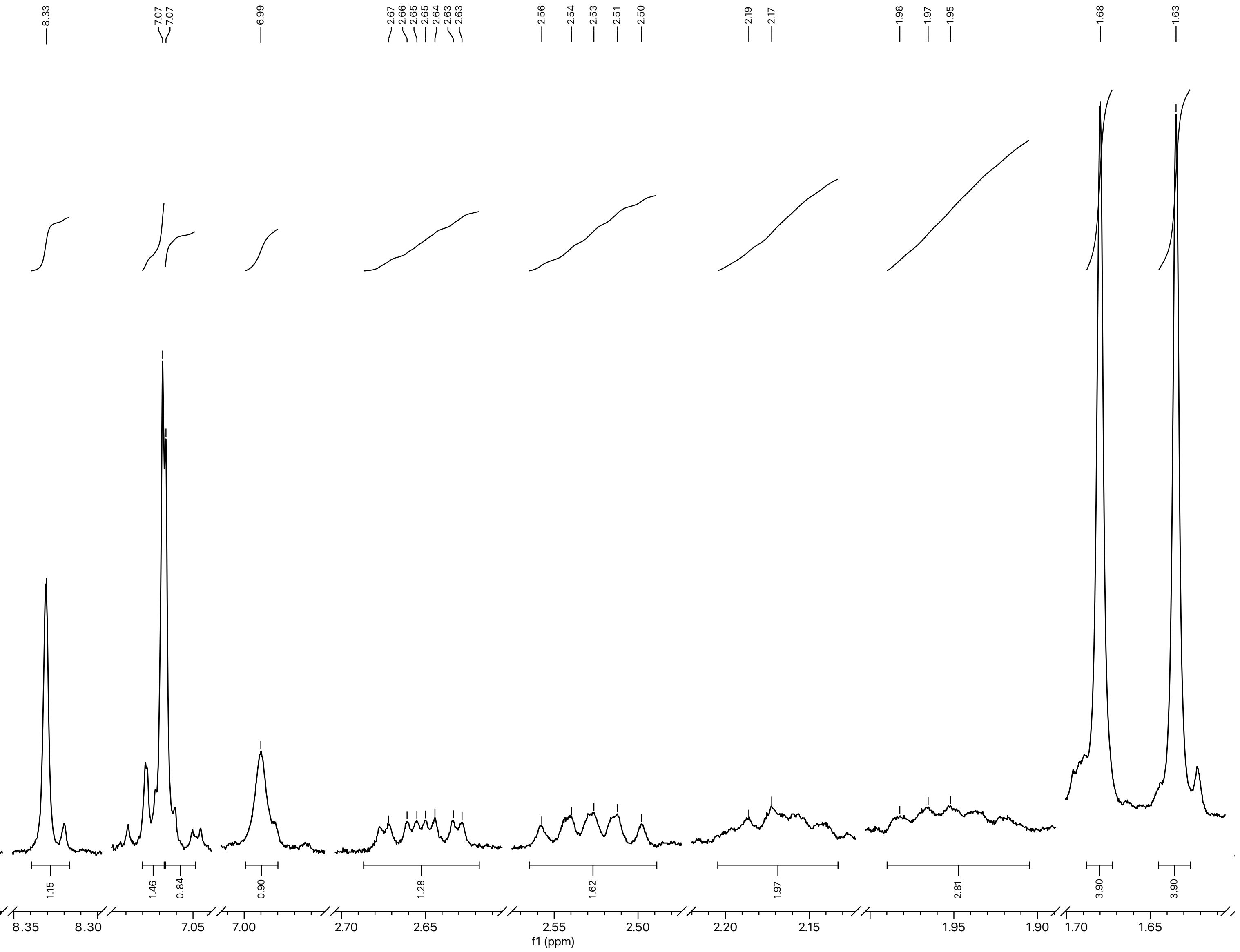

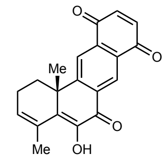

(−)-5: *ent*-orhalquinone
^{1}H NMR
$CDCl_3$, 500 MHz

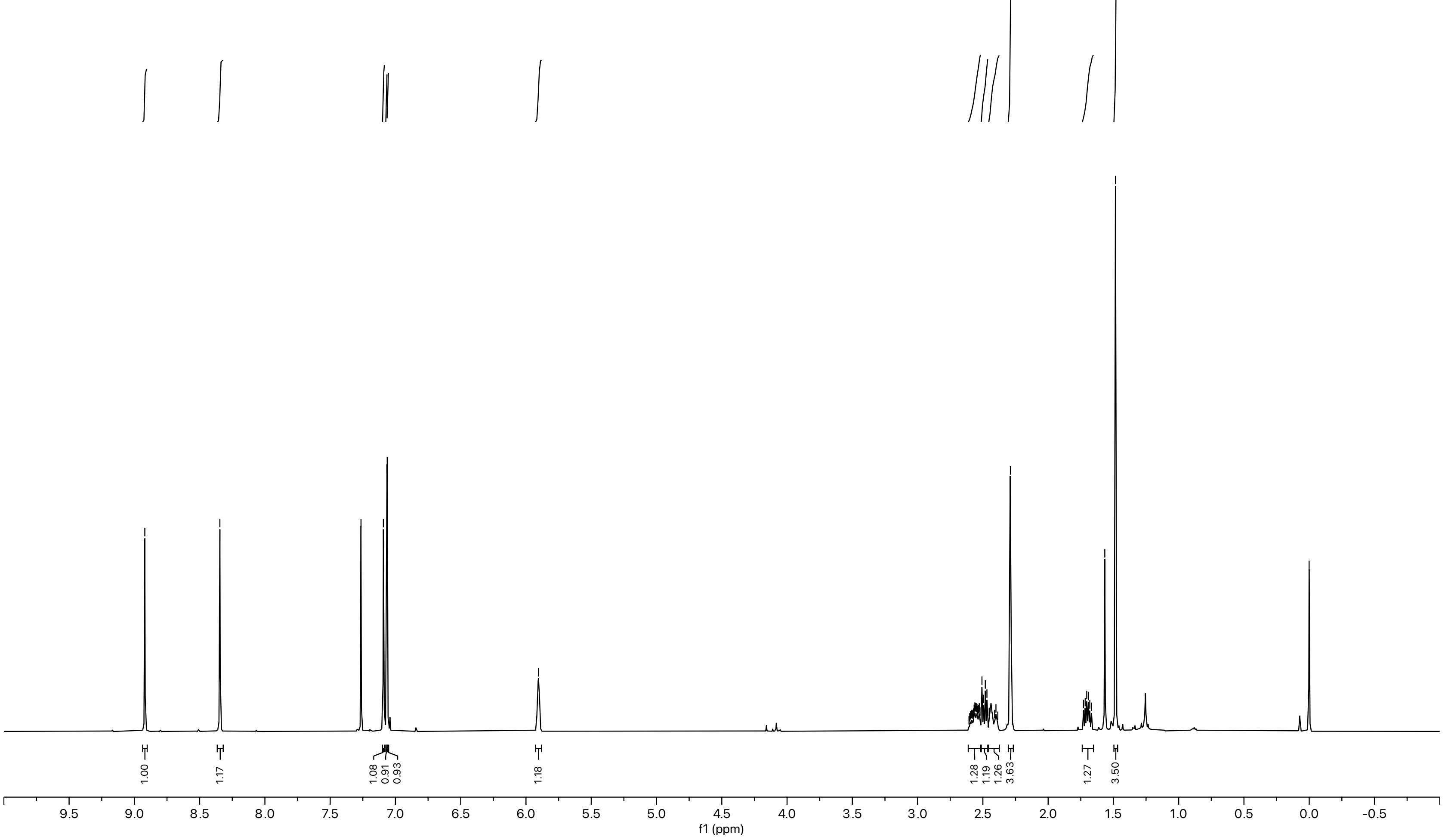

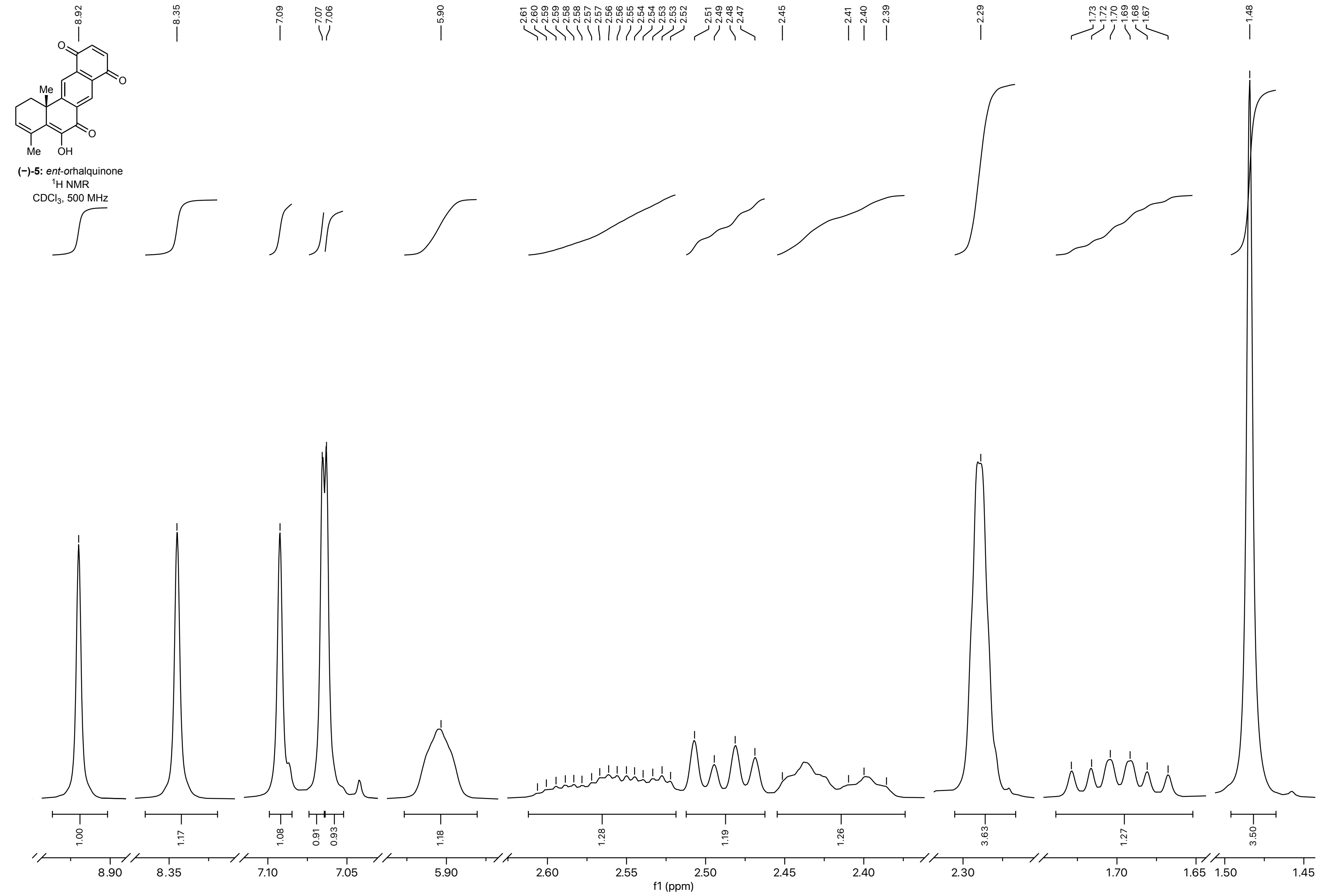
(−)-5: ent-orhalquinone
¹H NMR
CDCl3, 500 MHz
8.92
8.35
7.09
7.07
7.06
5.90
2.61 2.60 2.59 2.59 2.58 2.58 2.57 2.57 2.56 2.56 2.55 2.54 2.54 2.53 2.53 2.52
2.51 2.49 2.48 2.47
2.45
2.41 2.40 2.39
2.29
1.73 1.72 1.70 1.69 1.68 1.67
1.48
1.00 1.17 1.08 0.91 0.93 1.18 1.28 1.19 1.26 3.63 1.27 3.50
8.90 8.35 7.10 7.05 5.90 2.60 2.55 2.50 2.45 2.40 2.30 1.70 1.65 1.50 1.45
f1 (ppm)

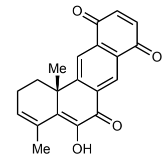

(−)-5: *ent*-orhalquinone
^{13}C NMR
$CDCl_3$, 125 MHz

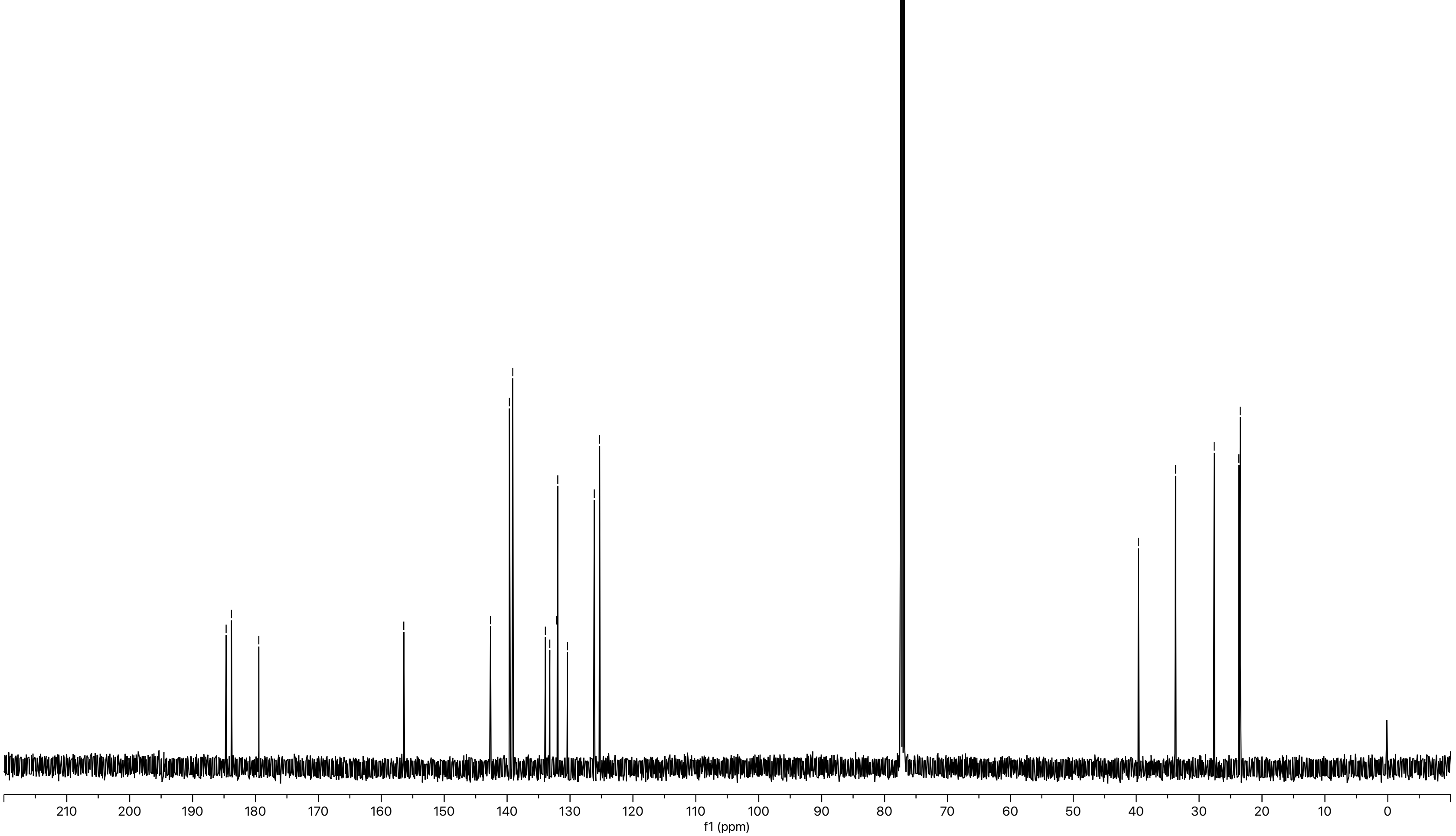

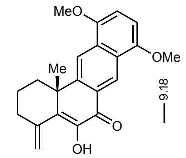

24
^{1}H NMR
$CDCl_3$, 500 MHz

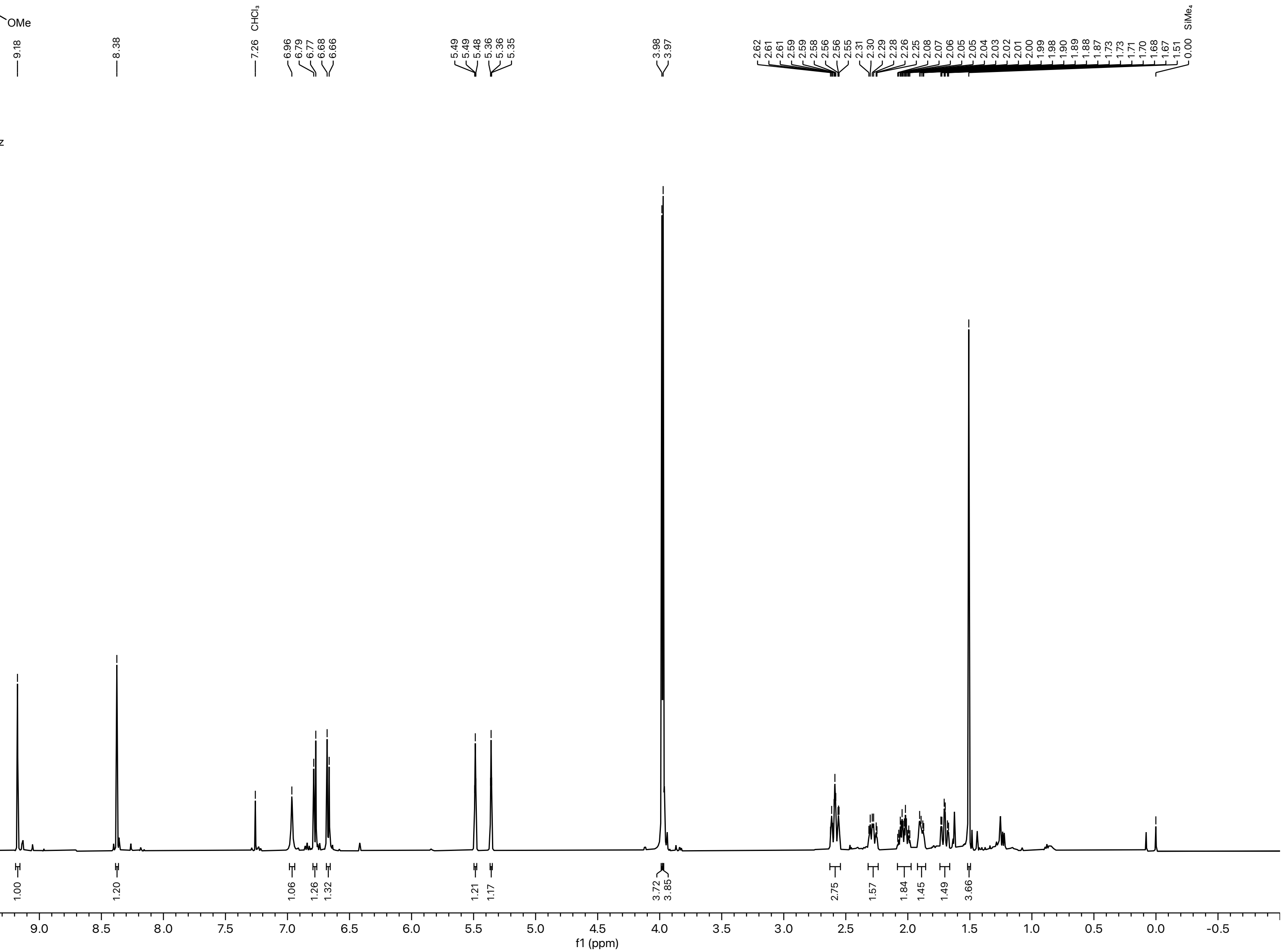

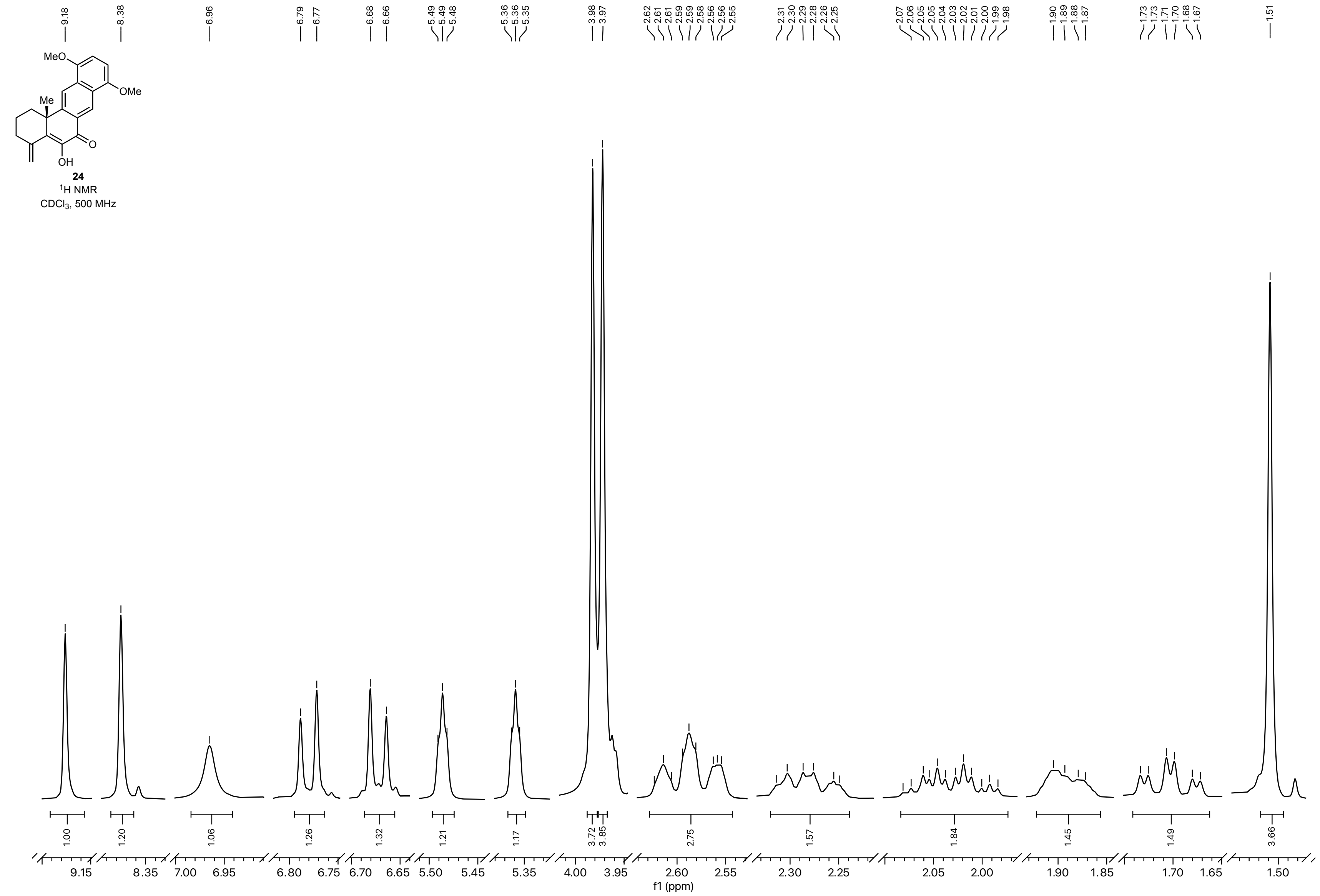

24
^{1}H NMR
$CDCl_3$, 500 MHz

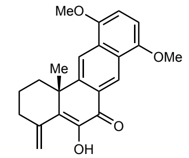

24
^{13}C NMR
$CDCl_3$, 125 MHz

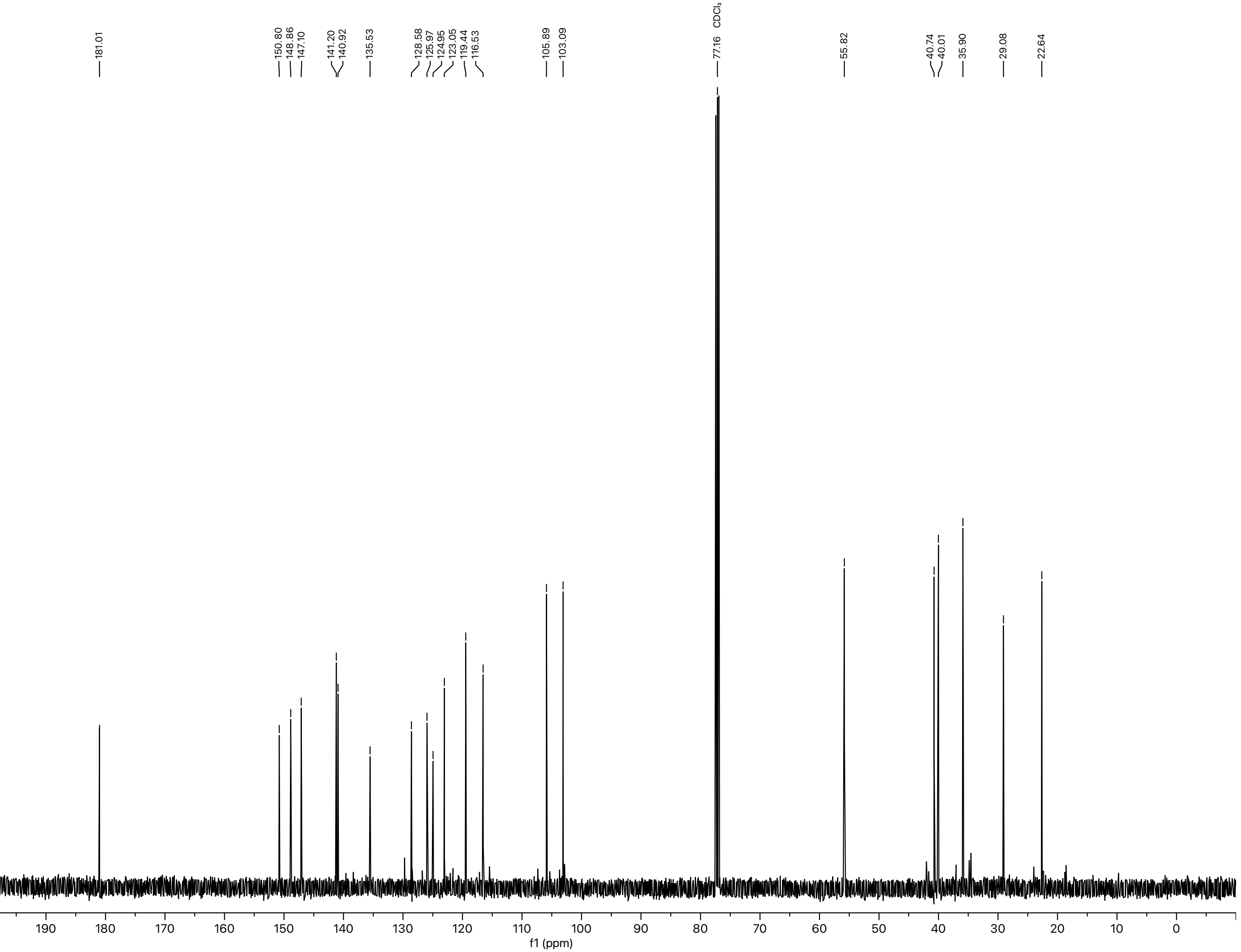

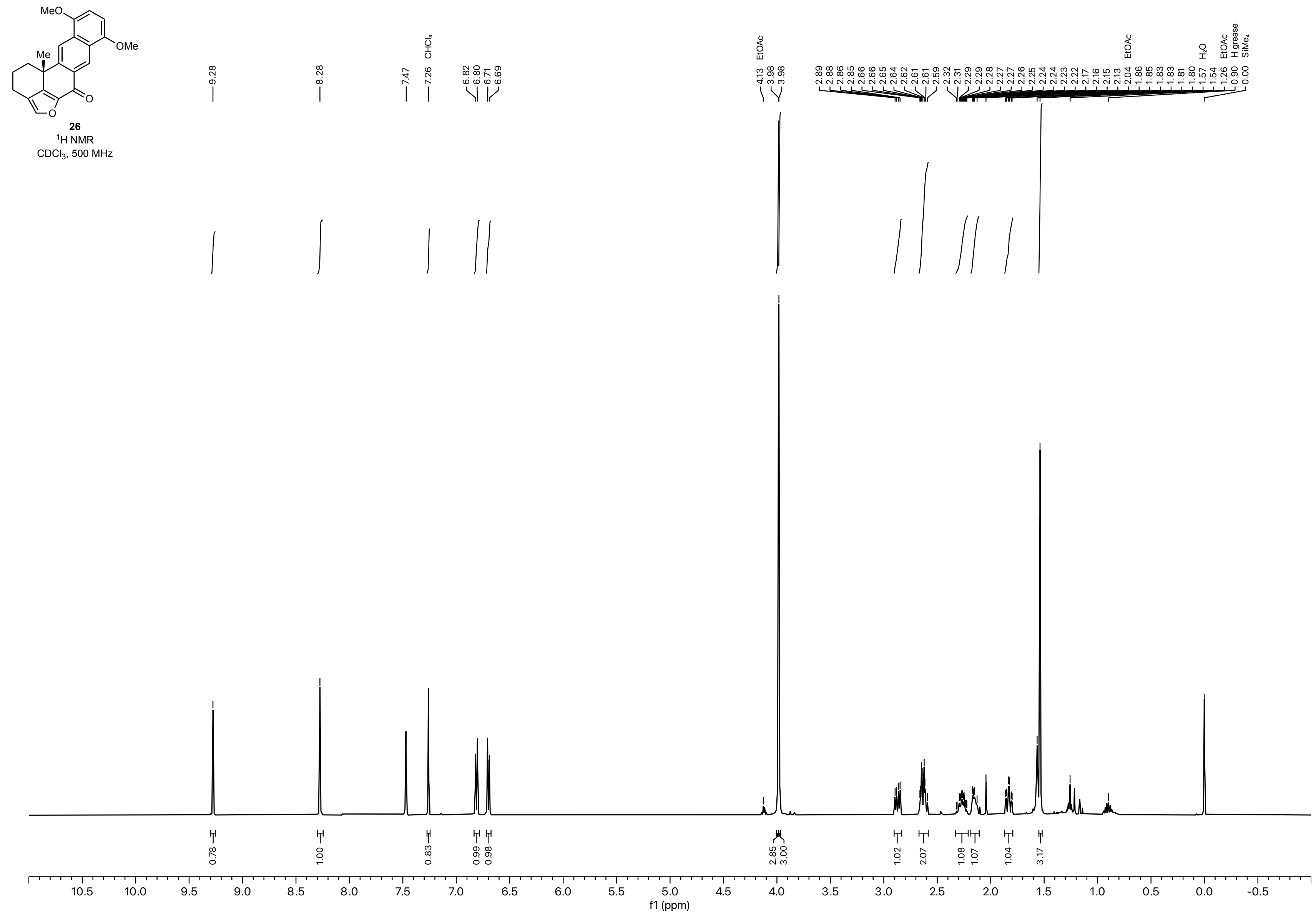
MeO
OMe
Me
O
O
26
^{1}H NMR
$CDCl_3$, 500 MHz
9.28
8.28
7.47
7.26 $CHCl_3$
6.82
6.80
6.71
6.69
4.13 EtOAc
3.98
3.98
2.89
2.88
2.86
2.85
2.66
2.66
2.65
2.64
2.62
2.61
2.61
2.59
2.32
2.31
2.29
2.29
2.28
2.27
2.27
2.26
2.25
2.24
2.24
2.23
2.22
2.17
2.16
2.15
2.13
2.04 EtOAc
1.86
1.85
1.83
1.83
1.81
1.80
1.57 H_2O
1.54
1.26 EtOAc
0.90 H grease
0.00 $SiMe_4$
0.78
1.00
0.83
0.99
0.98
2.85
3.00
1.02
2.07
1.08
1.07
1.04
3.17
10.5
10.0
9.5
9.0
8.5
8.0
7.5
7.0
6.5
6.0
5.5
5.0
4.5
4.0
3.5
3.0
2.5
2.0
1.5
1.0
0.5
0.0
-0.5
f1 (ppm)

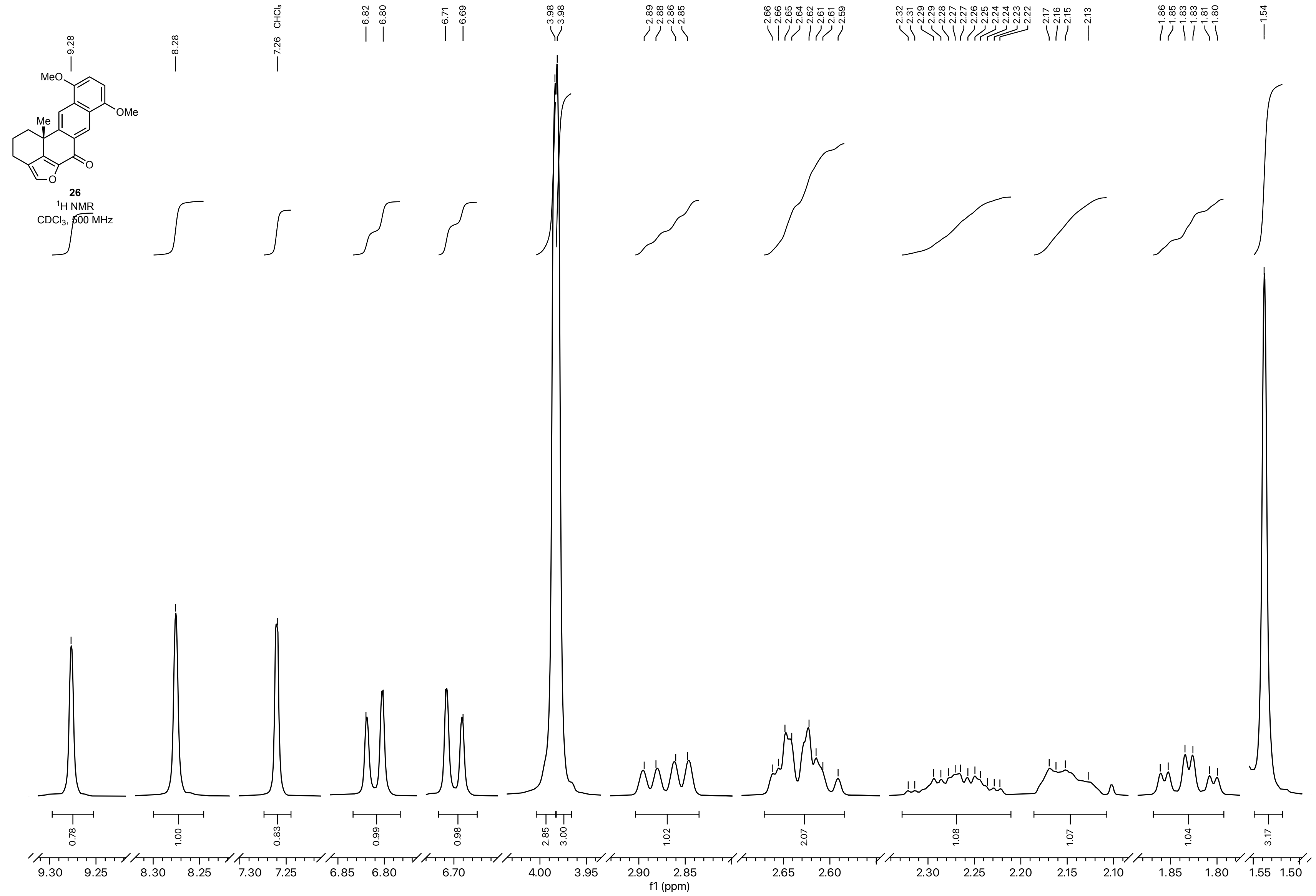
MeO
OMe
Me
O
O
26
^{1}H NMR
$CDCl_3$, 500 MHz
9.28
8.28
7.26 $CHCl_3$
6.82
6.80
6.71
6.69
3.98
3.98
2.89
2.88
2.86
2.85
2.66
2.66
2.65
2.64
2.62
2.61
2.61
2.59
2.32
2.31
2.29
2.29
2.28
2.27
2.27
2.26
2.25
2.24
2.24
2.23
2.22
2.17
2.16
2.15
2.13
1.86
1.85
1.83
1.83
1.81
1.80
1.54
0.78
1.00
0.83
0.99
0.98
2.85
3.00
1.02
2.07
1.08
1.07
1.04
3.17
9.30 9.25 8.30 8.25 7.30 7.25 6.85 6.80 6.70 4.00 3.95 2.90 2.85 2.65 2.60 2.30 2.25 2.20 2.15 2.10 1.85 1.80 1.55 1.50
f1 (ppm)

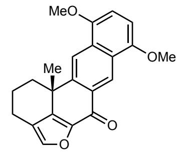

26
^{13}C NMR
$CDCl_3$, 125 MHz

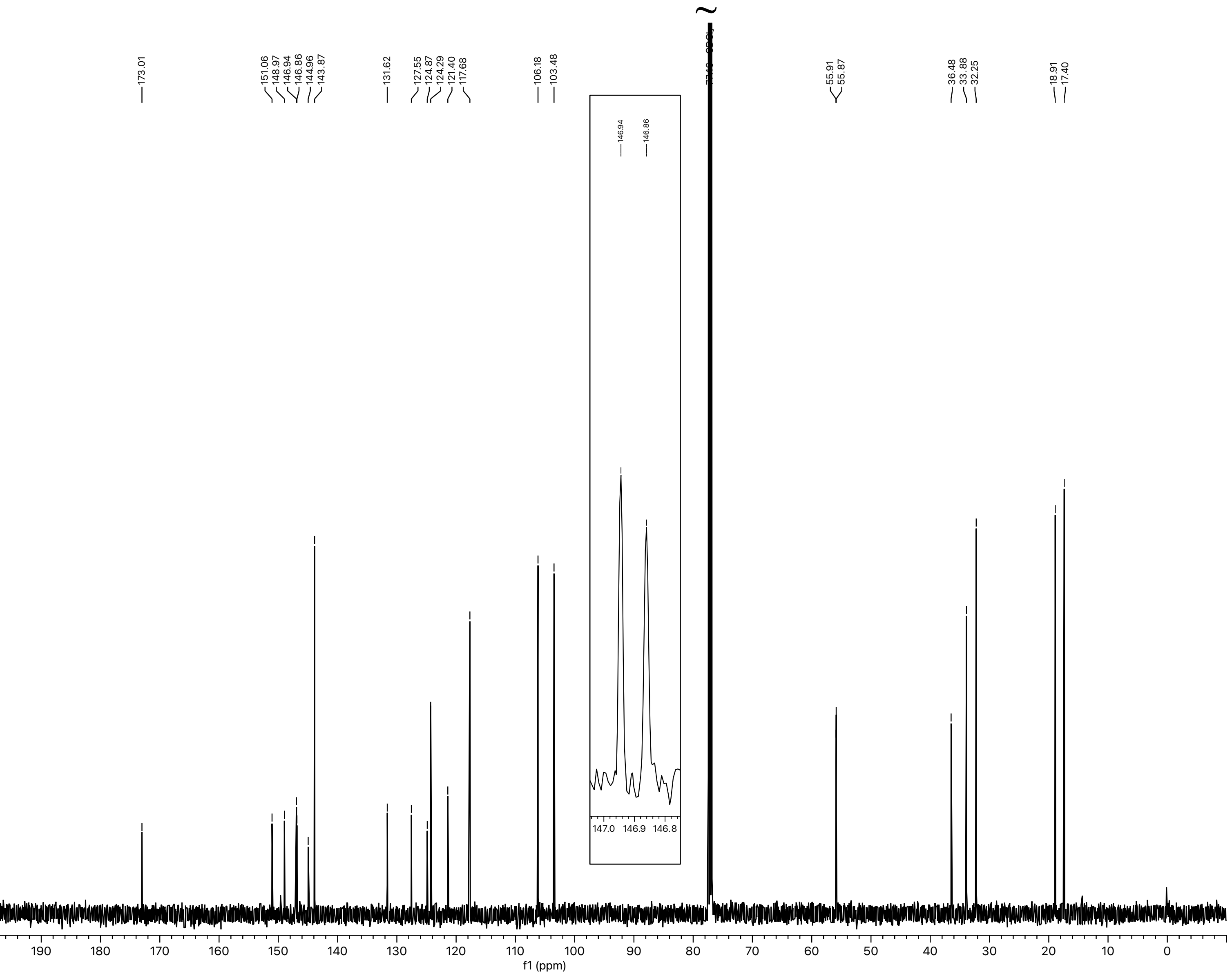

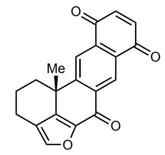

(−)-7: *ent*-xestoquinone
^{1}H NMR
$CDCl_3$, 400 MHz

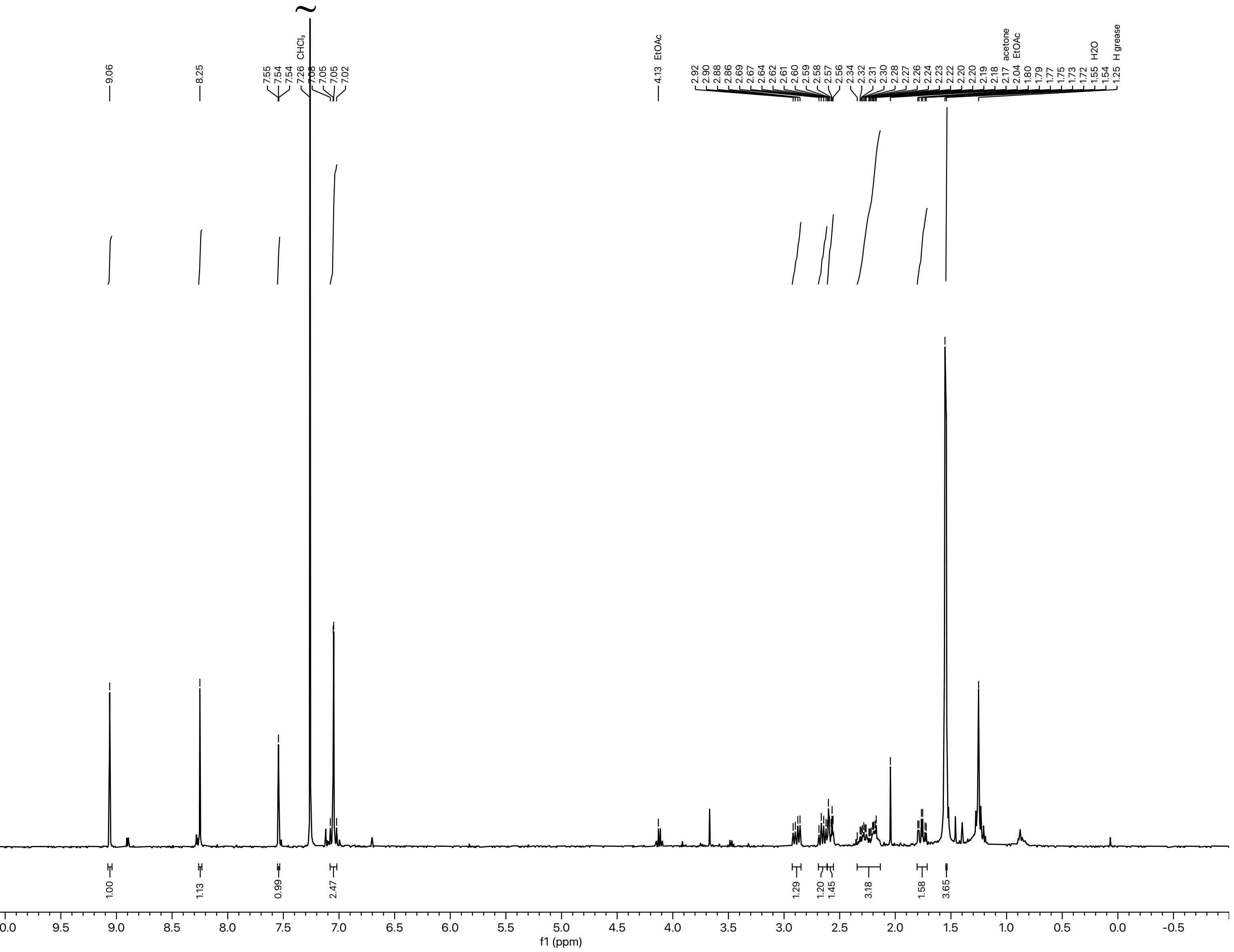

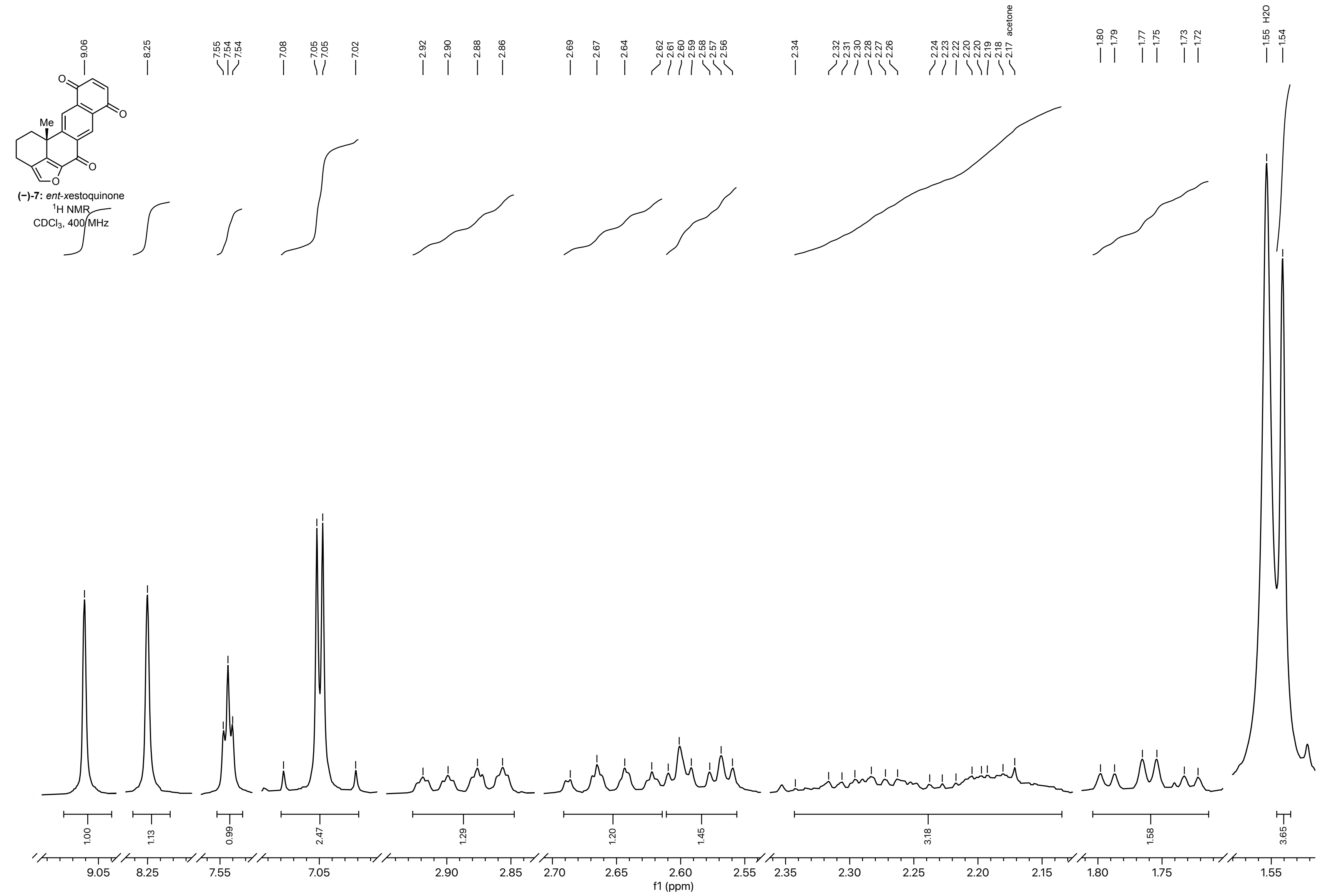
(−)-7: ent-xestoquinone
1H NMR
CDCl3, 400 MHz
Me
9.06
8.25
7.55 7.54 7.54
7.08 7.05 7.05 7.02
2.92 2.90 2.88 2.86
2.69 2.67 2.64 2.62 2.61 2.60 2.59 2.58 2.57 2.56
2.34 2.32 2.31 2.30 2.28 2.27 2.26 2.24 2.23 2.22 2.20 2.20 2.19 2.18 2.17 acetone
1.80 1.79 1.77 1.75 1.73 1.72
1.55 H2O 1.54
1.00 1.13 0.99 2.47 1.29 1.20 1.45 3.18 1.58 3.65
9.05 8.25 7.55 7.05 2.90 2.85 2.70 2.65 2.60 2.55 2.35 2.30 2.25 2.20 2.15 1.80 1.75 1.55
f1 (ppm)

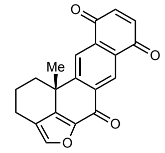

(−)-7: *ent*-xestoquinone
^{13}C NMR
$CDCl_3$, 150 MHz

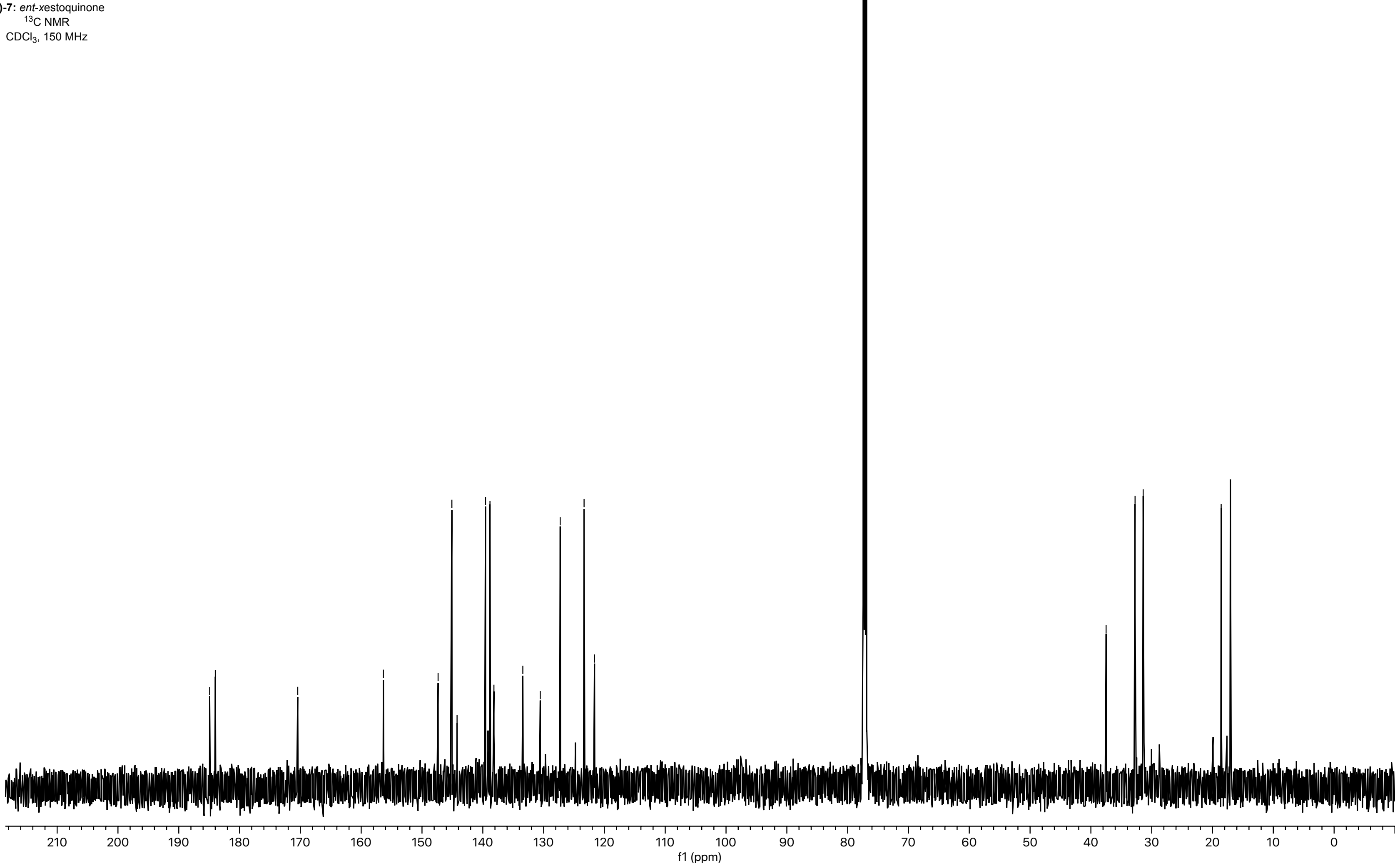

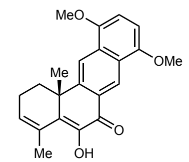

25
^{1}H NMR
$CDCl_3$, 500 MHz

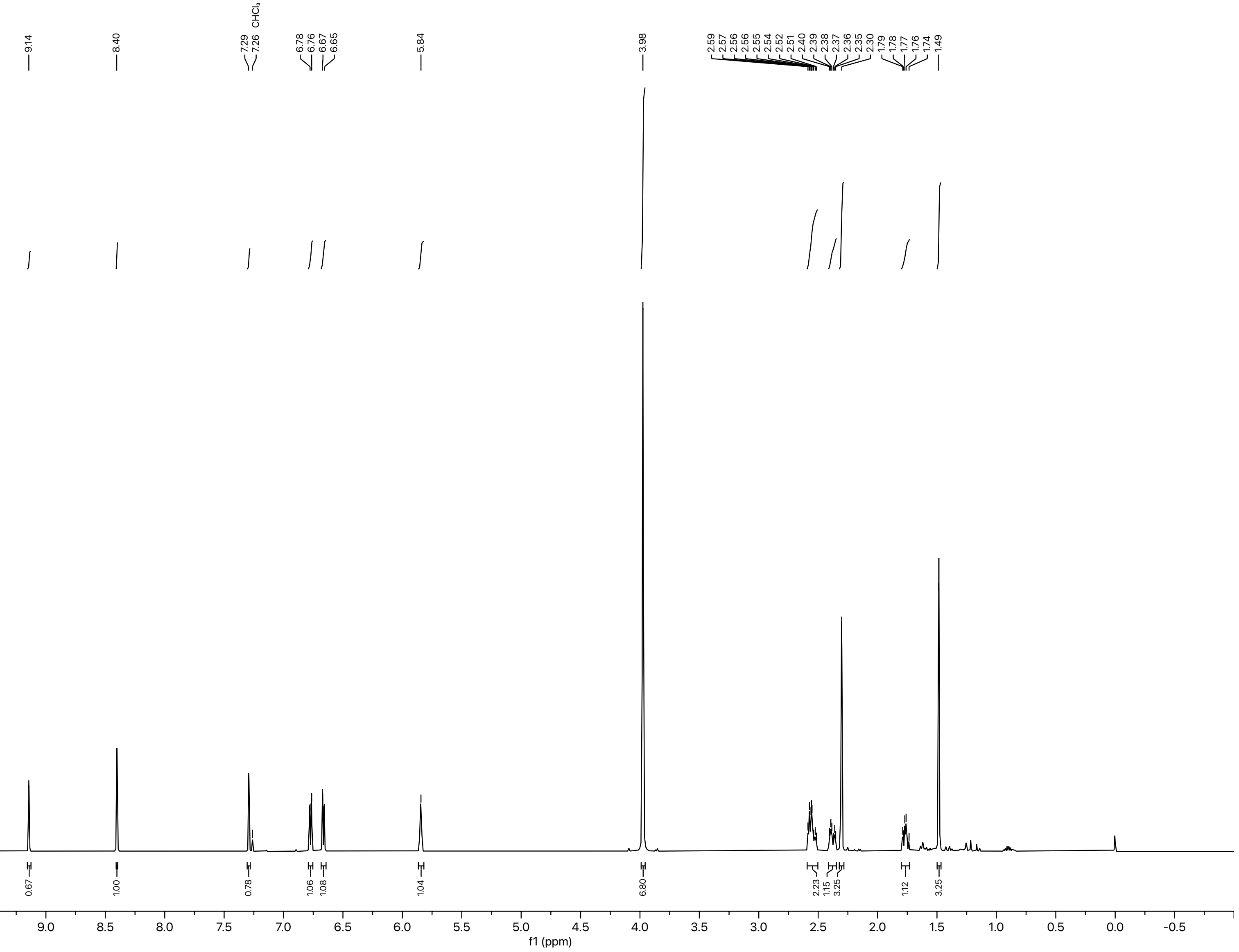

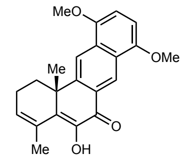

25
^1H NMR
$CDCl_3$, 500 MHz

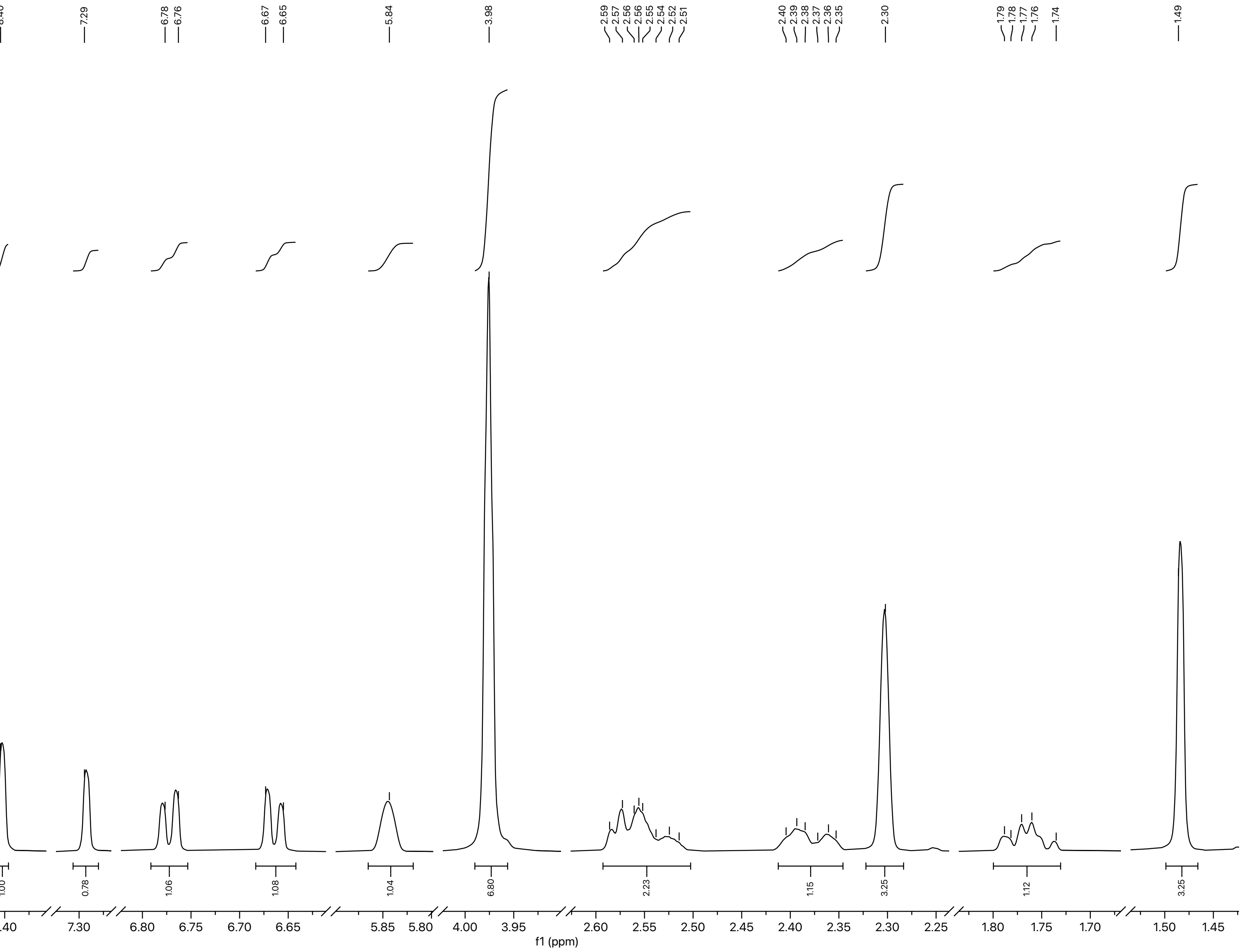

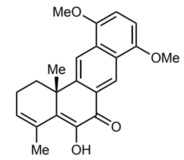

25

^{13}C NMR

$CDCl_3$, 125 MHz

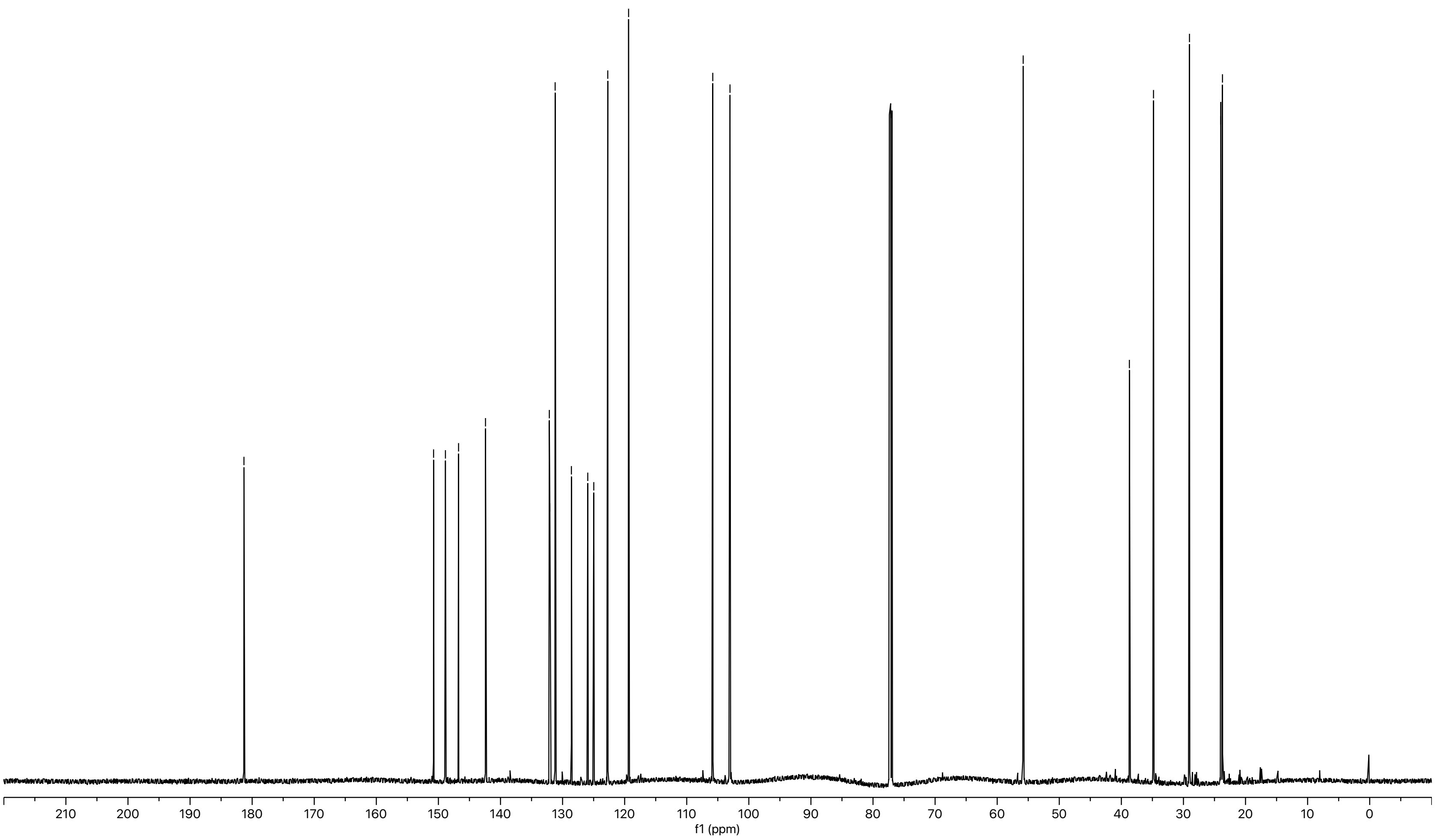

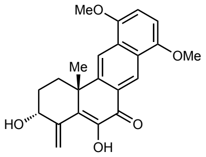

27
^{1}H NMR
$CDCl_3$, 500 MHz

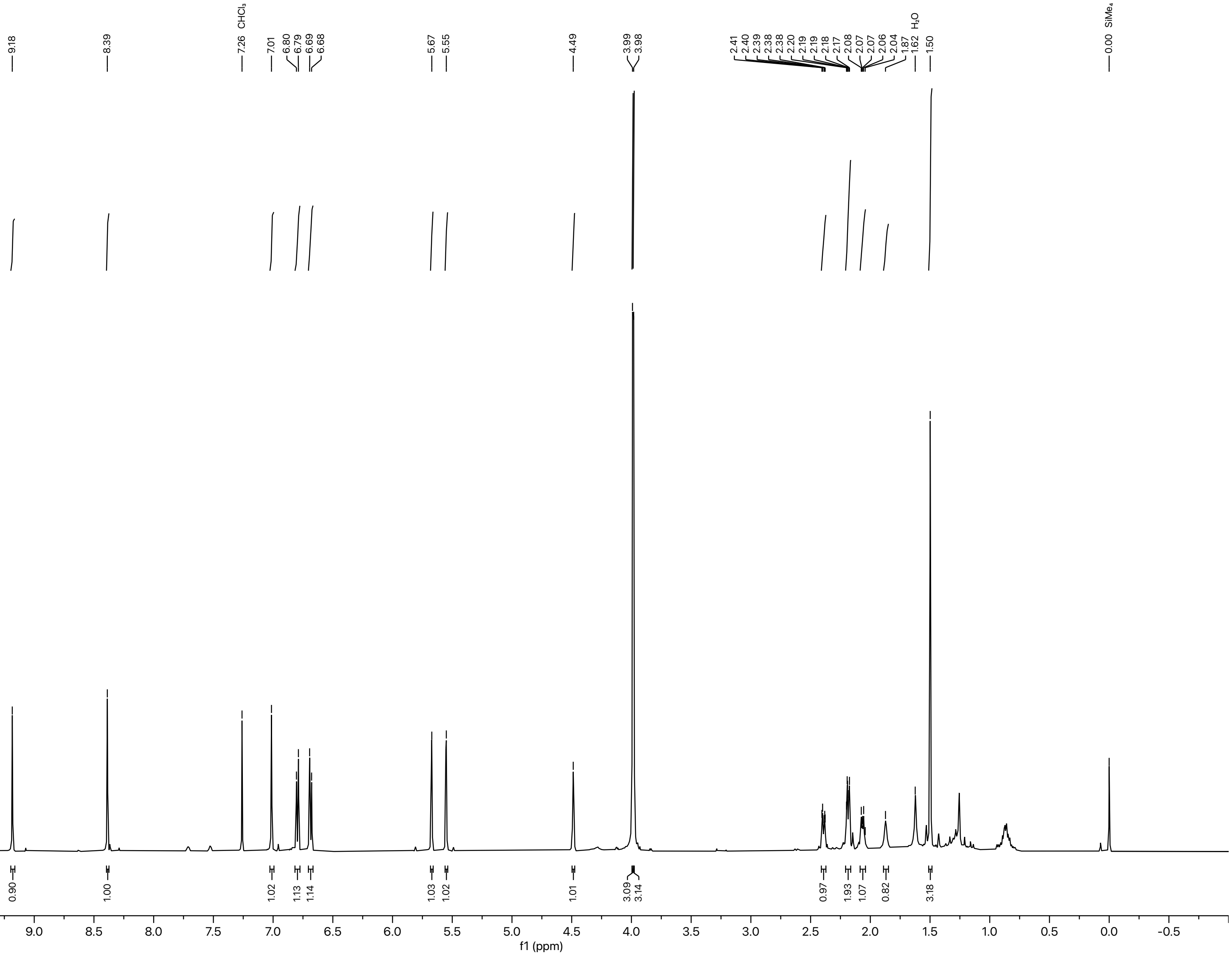

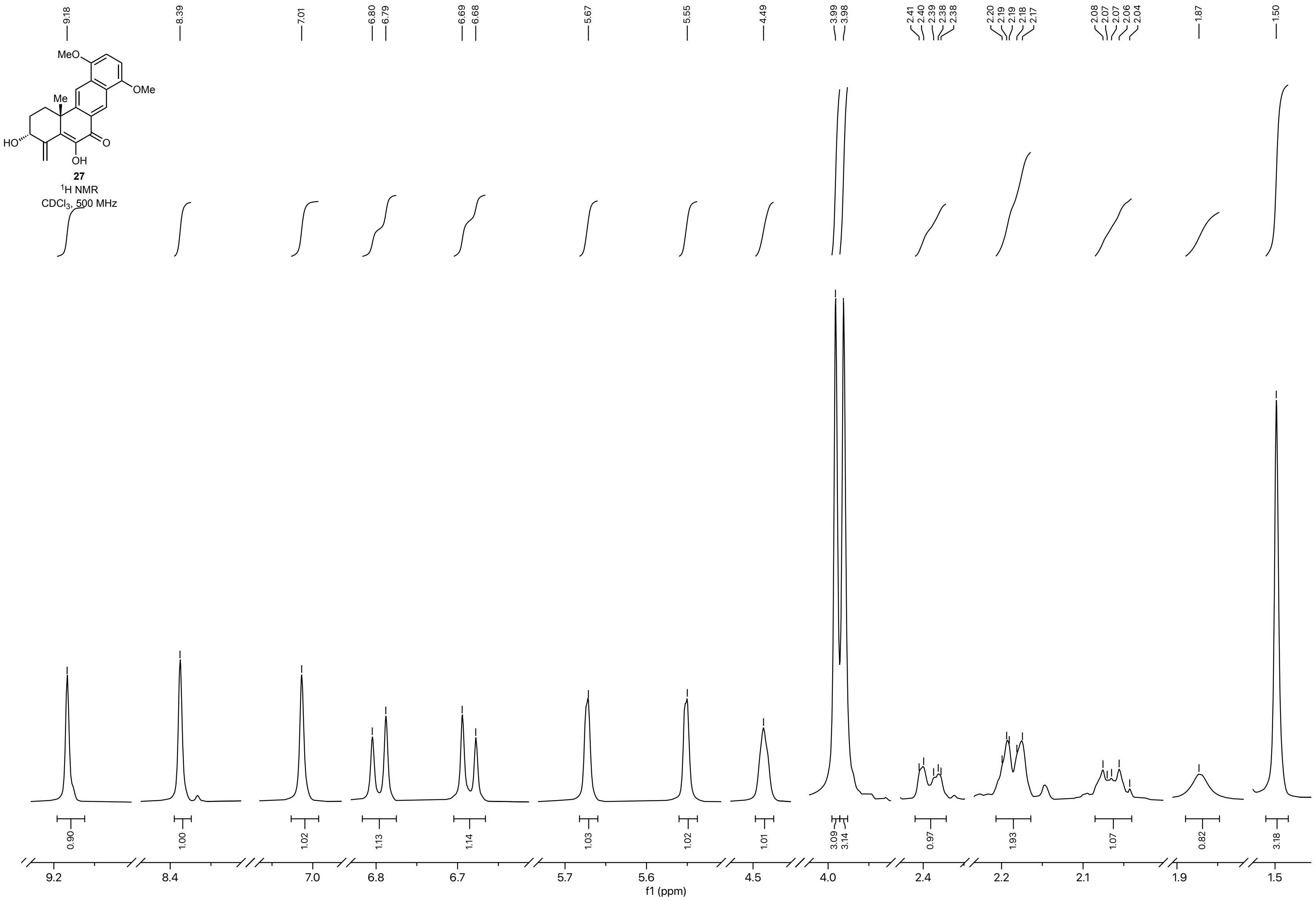
MeO
OMe
Me
HO
OH
O
27
^{1}H NMR
$CDCl_3$, 500 MHz
9.18
8.39
7.01
6.80
6.79
6.69
6.68
5.67
5.55
4.49
3.99
3.98
2.41
2.40
2.39
2.38
2.38
2.20
2.19
2.19
2.18
2.17
2.08
2.07
2.07
2.06
2.04
1.87
1.50
0.90
1.00
1.02
1.13
1.14
1.03
1.02
1.01
3.09
3.14
0.97
1.93
1.07
0.82
3.18
9.2
8.4
7.0
6.8
6.7
5.7
5.6
4.5
4.0
2.4
2.2
2.1
1.9
1.5
f1 (ppm)

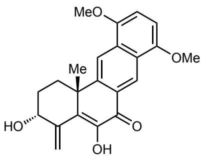

27

^{13}C NMR

$CDCl_3$, 125 MHz

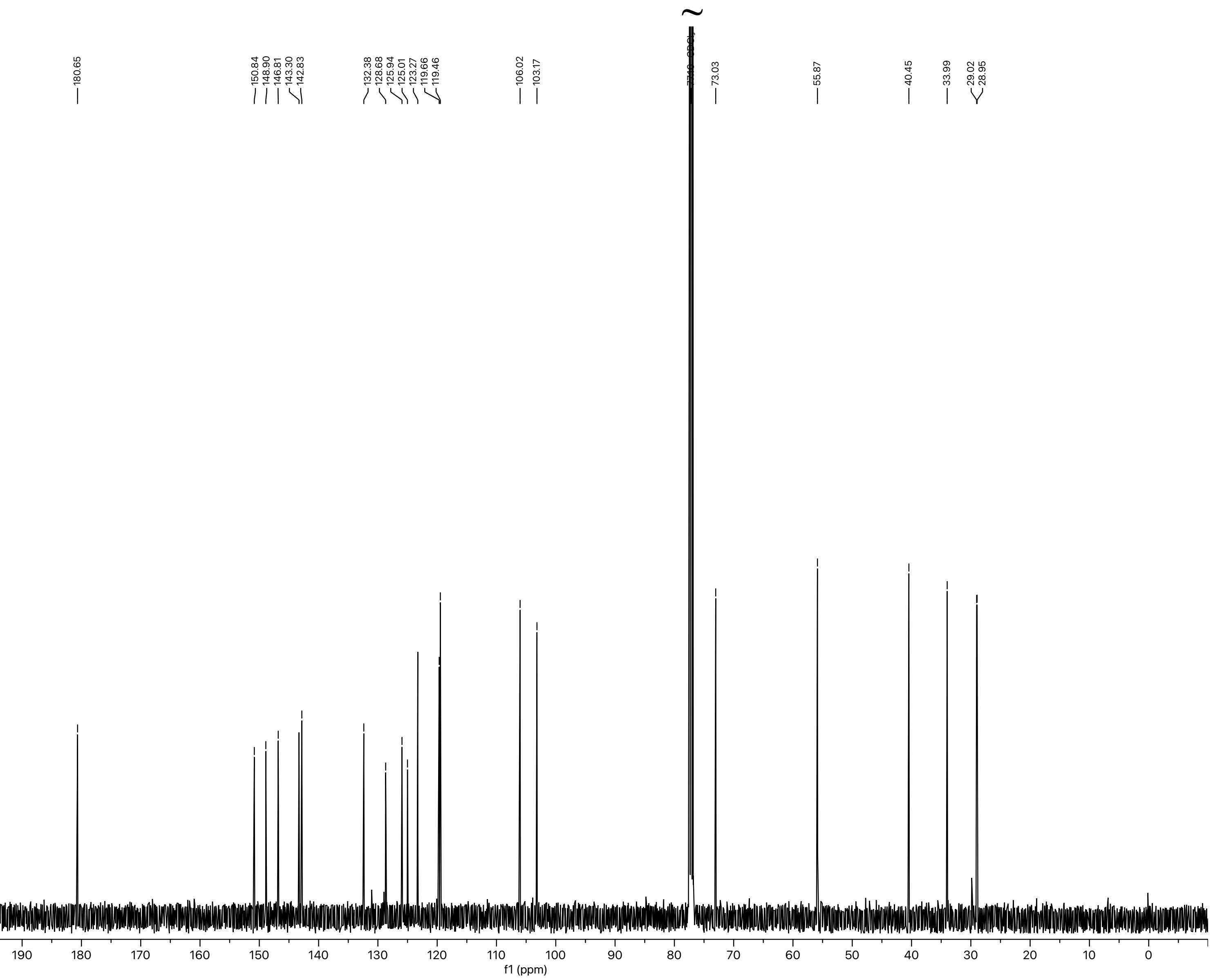

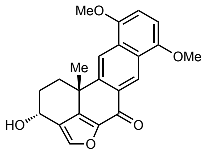

SI-09 (crude)
^1H NMR
$CDCl_3$, 500 MHz

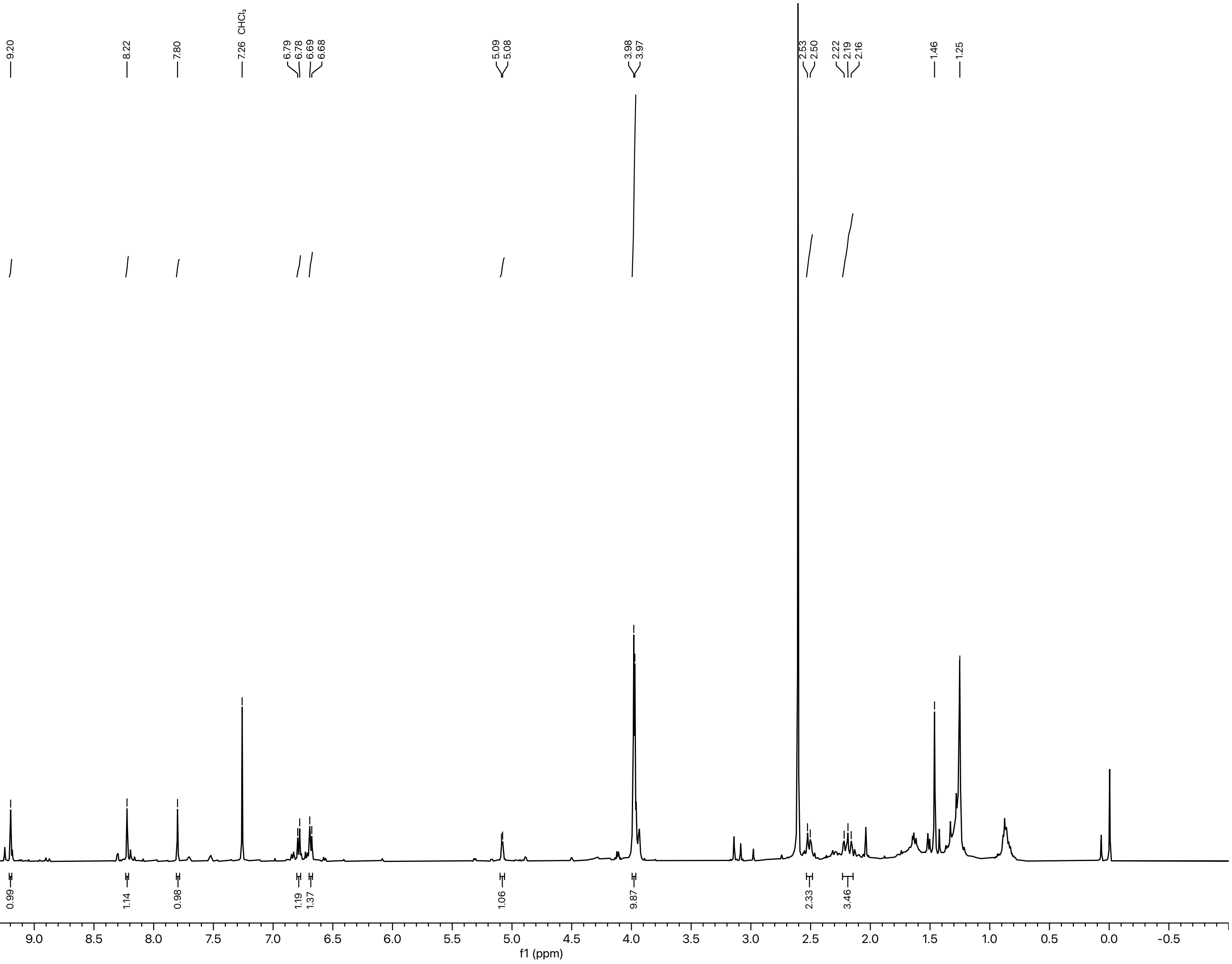

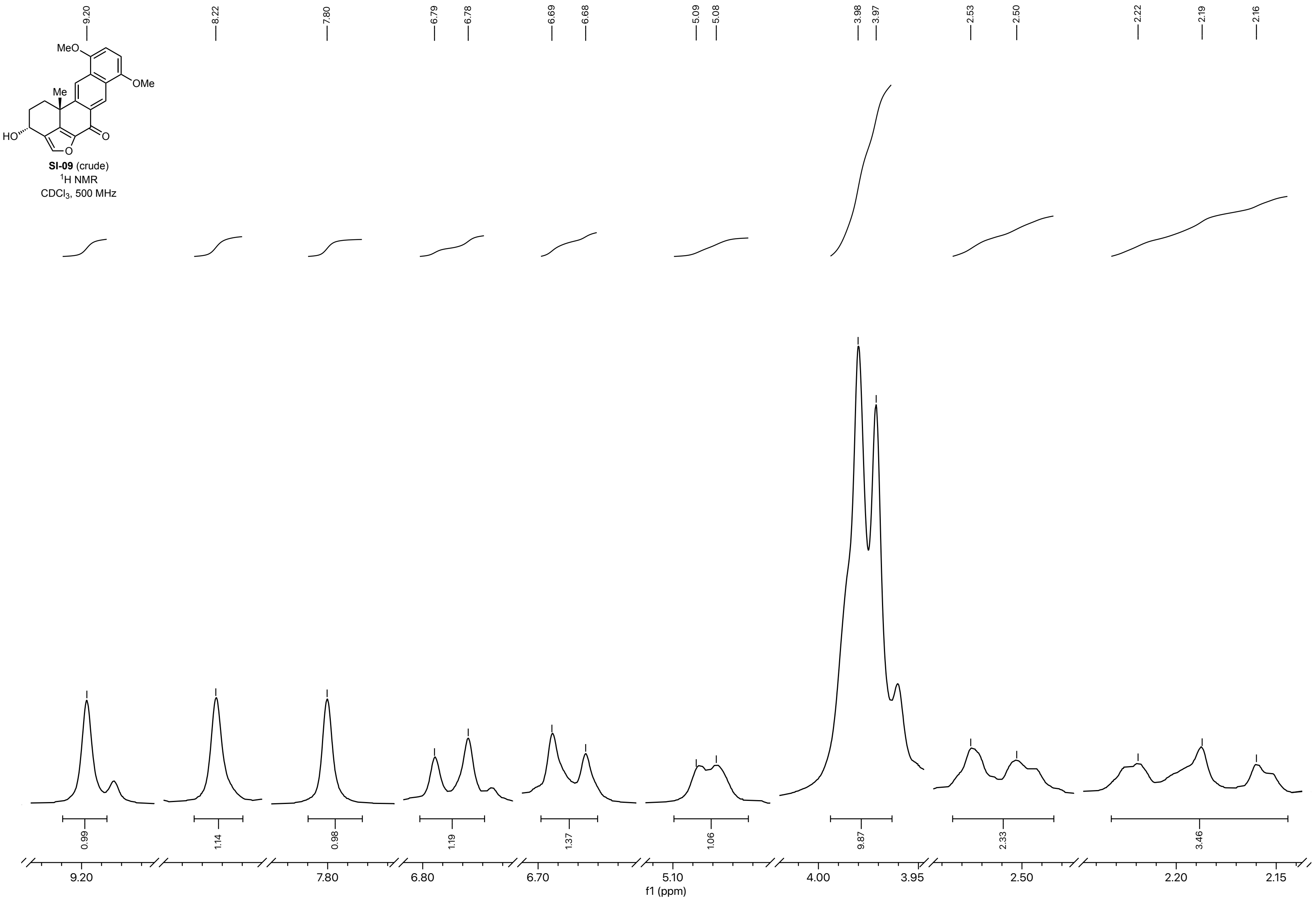
MeO
OMe
Me
HO
O
O
SI-09 (crude)
^{1}H NMR
$CDCl_3$, 500 MHz
9.20
8.22
7.80
6.79
6.78
6.69
6.68
5.09
5.08
3.98
3.97
2.53
2.50
2.22
2.19
2.16
0.99
1.14
0.98
1.19
1.37
1.06
9.87
2.33
3.46
9.20
7.80
6.80
6.70
5.10
4.00
3.95
2.50
2.20
2.15
f1 (ppm)

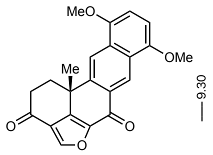

33

^1H NMR

$CDCl_3$, 500 MHz

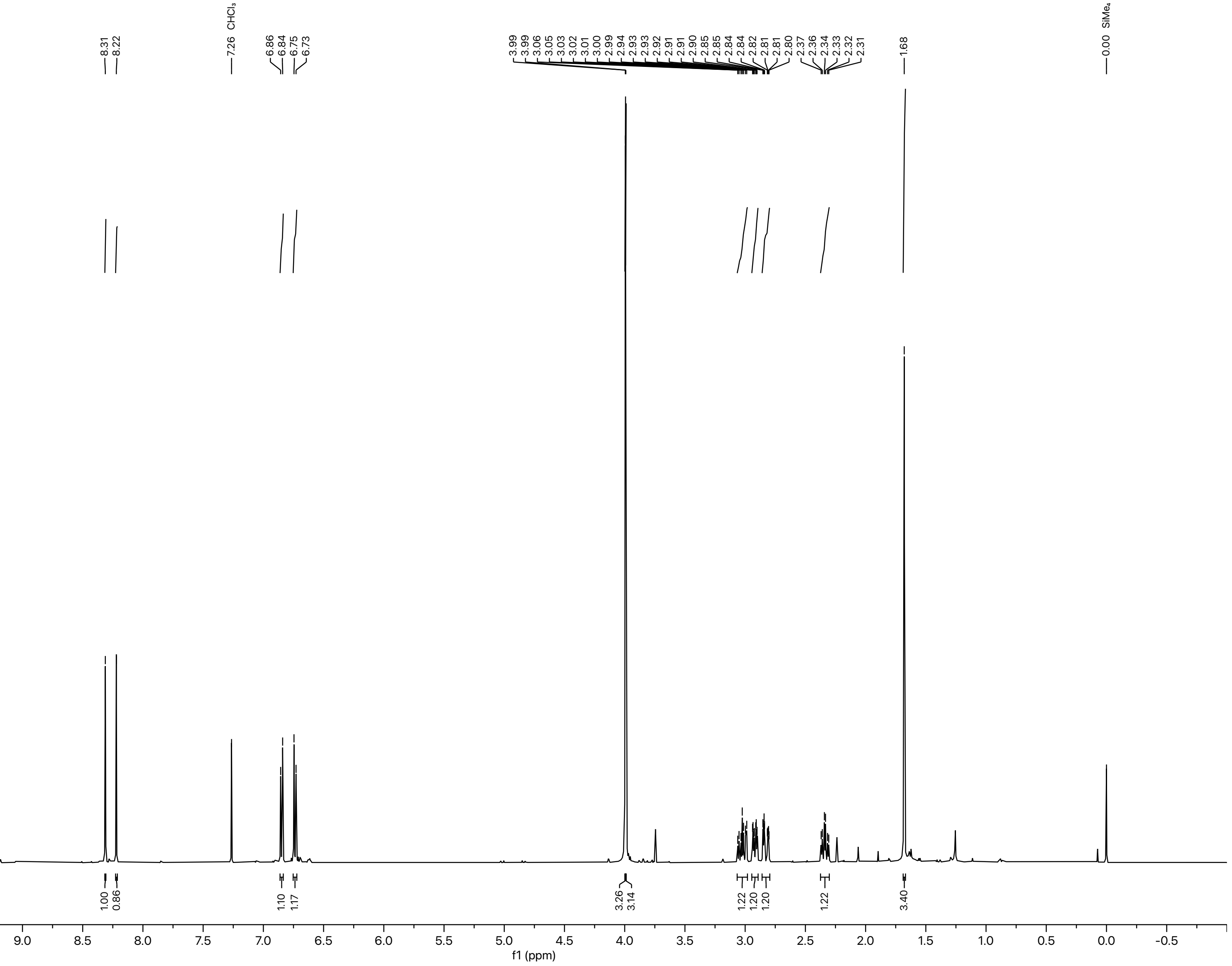

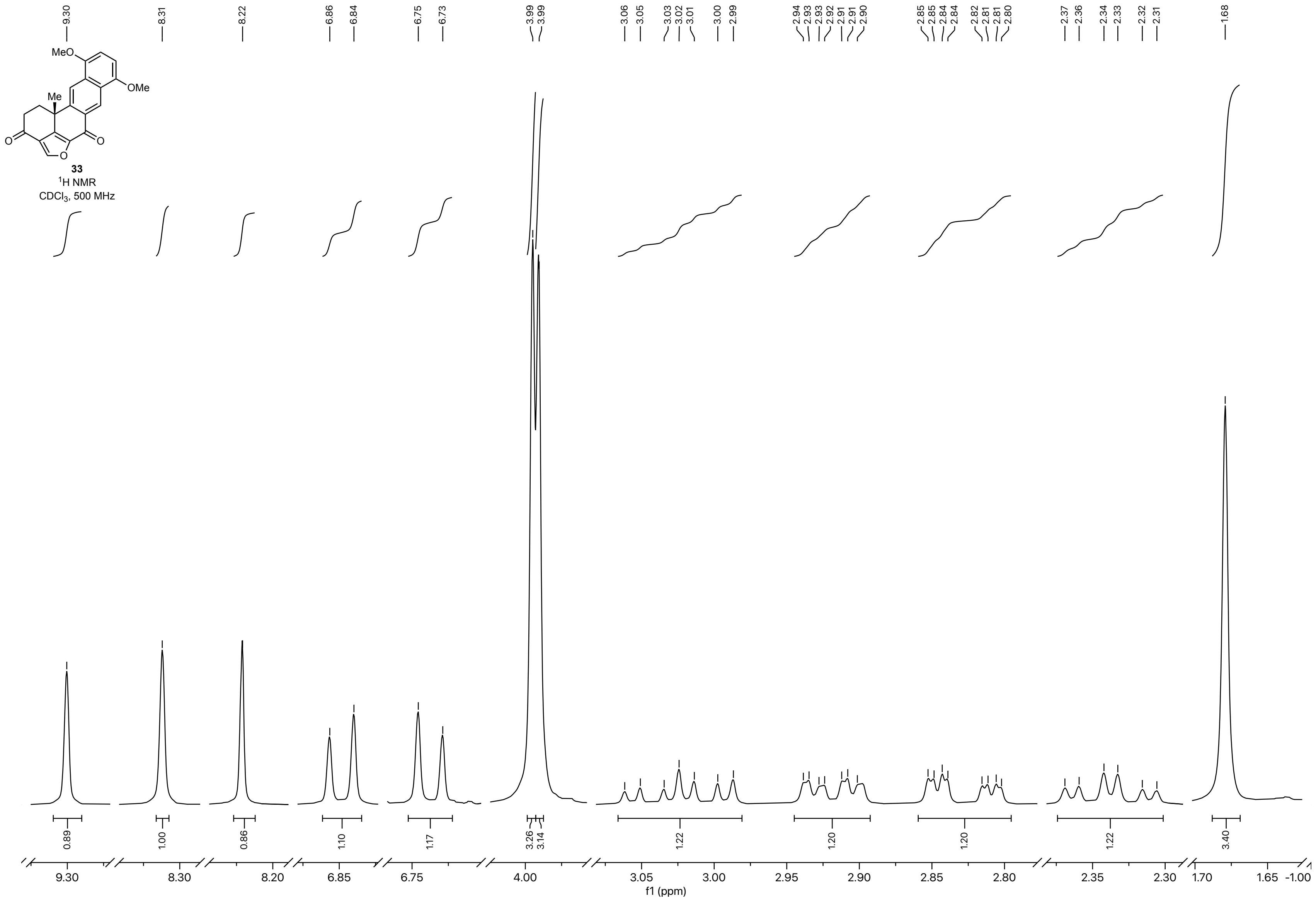
MeO
OMe
Me
O
O
O
33
^{1}H NMR
$CDCl_3$, 500 MHz
f1 (ppm)

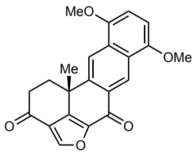

33
^{13}C NMR
$CDCl_3$, 125 MHz

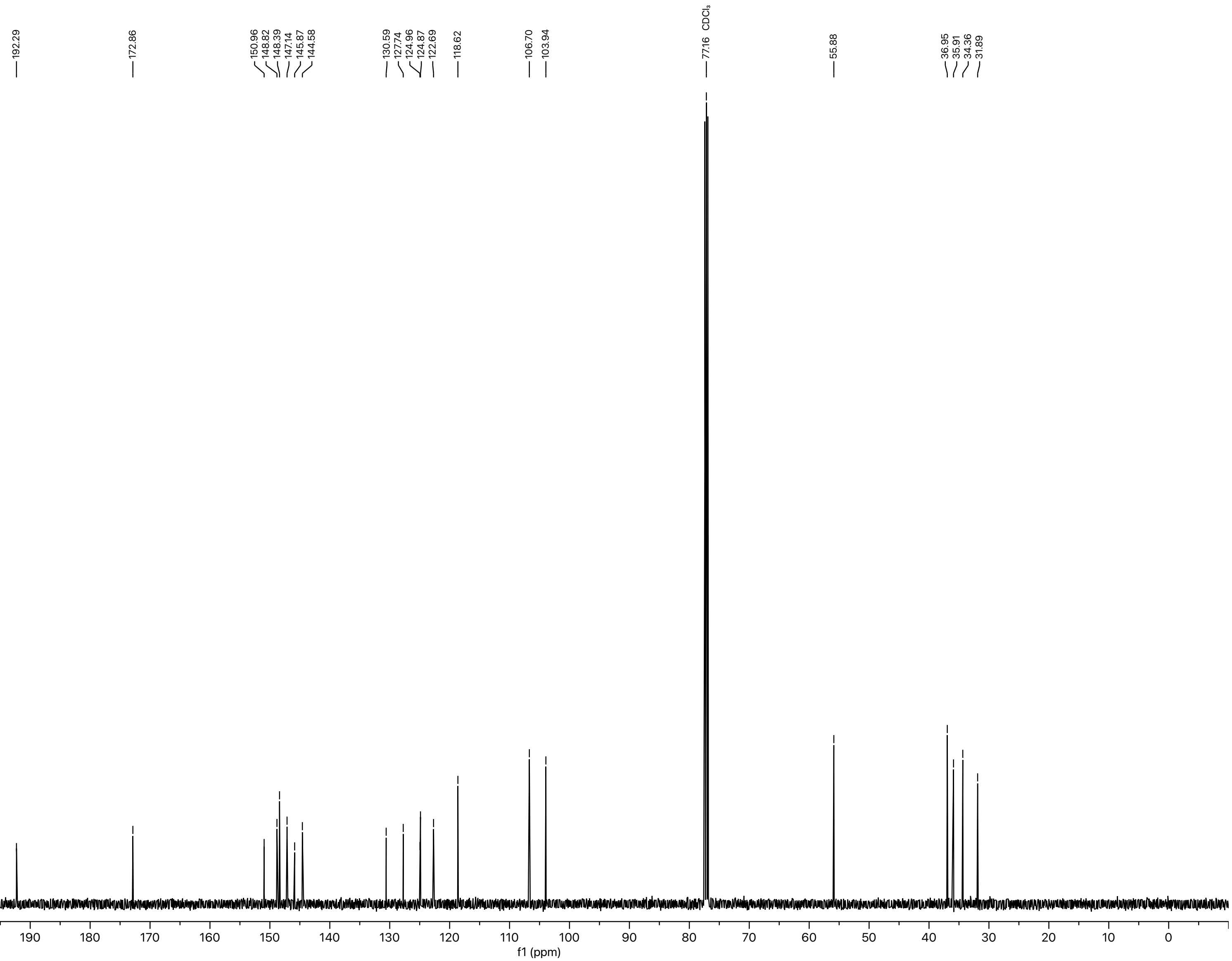

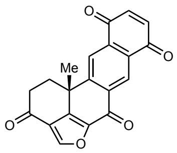

(−)-1: *ent-h*alenaquinone
^{1}H NMR
$(CD_3)_2SO$, 400 MHz

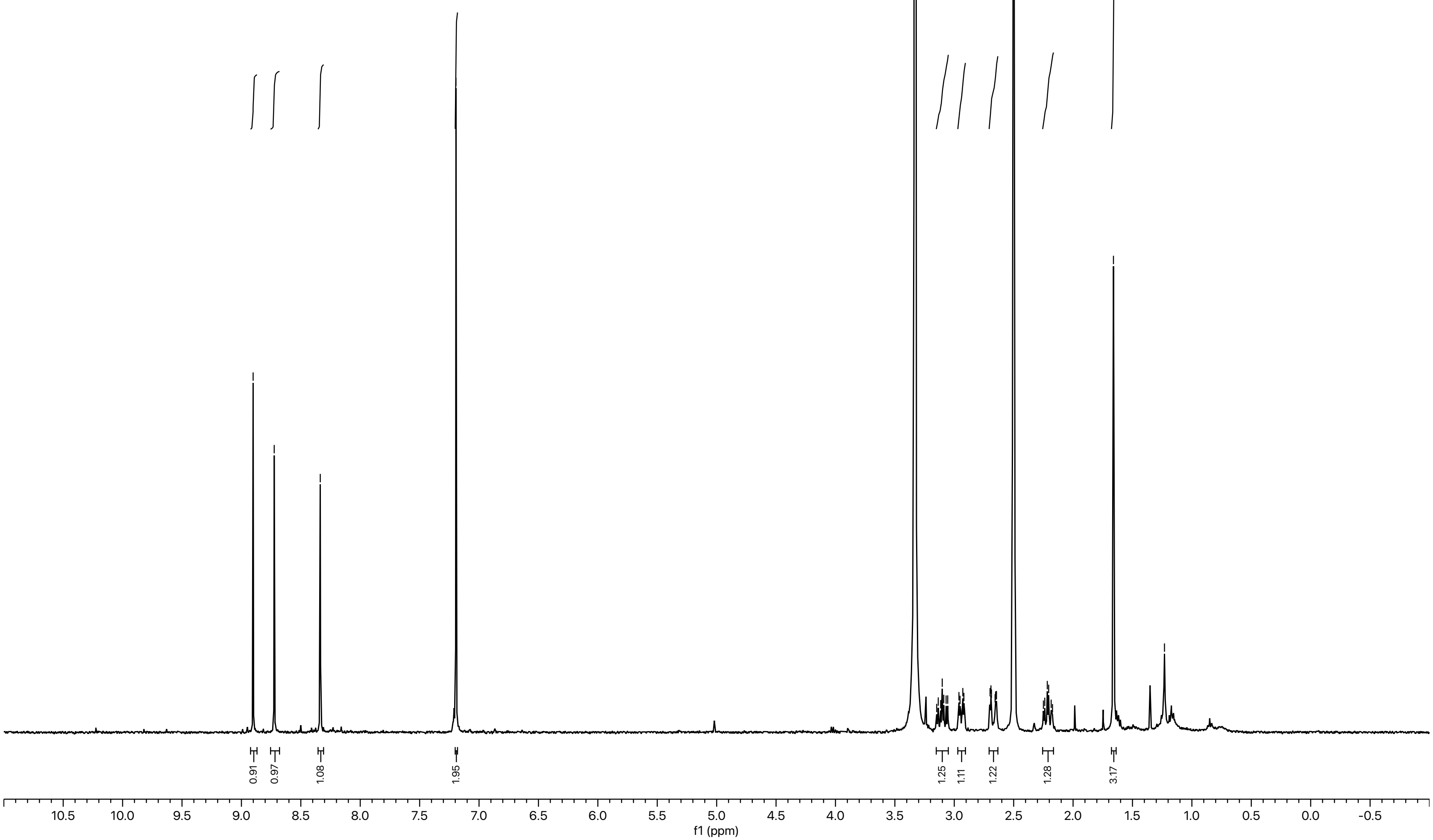

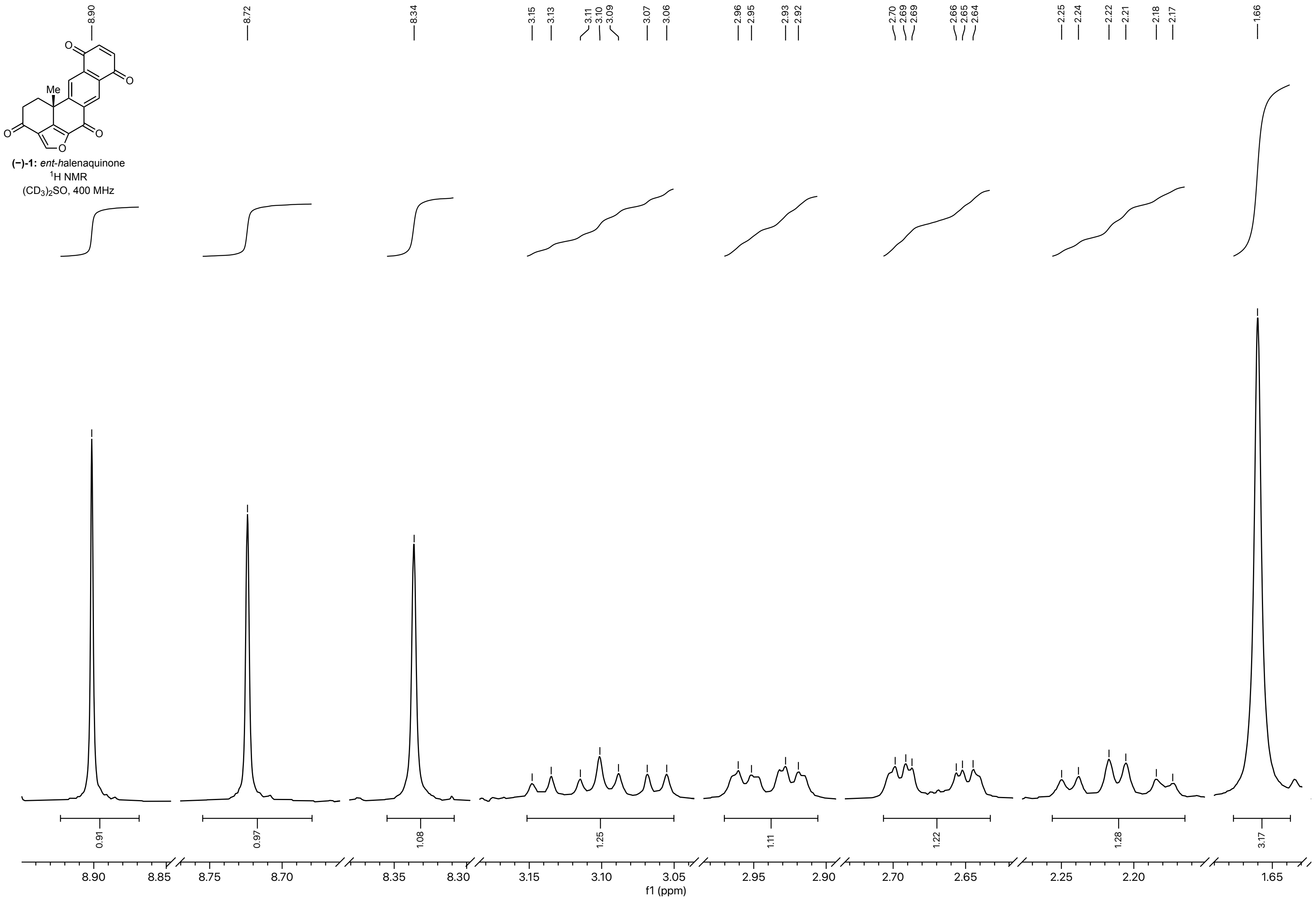
O
O
Me
O
O
O
O
(−)-1: ent-halenaquinone
^{1}H NMR
$(CD_3)_2SO$, 400 MHz
8.90
8.72
8.34
3.15
3.13
3.11
3.10
3.09
3.07
3.06
2.96
2.95
2.93
2.92
2.70
2.69
2.69
2.66
2.65
2.64
2.25
2.24
2.22
2.21
2.18
2.17
1.66
0.91
0.97
1.08
1.25
1.11
1.22
1.28
3.17
f1 (ppm)

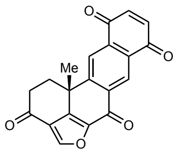

(−)-1: *ent*-halenaquinone
^{13}C NMR
$(CD_3)_2SO$, 225 MHz

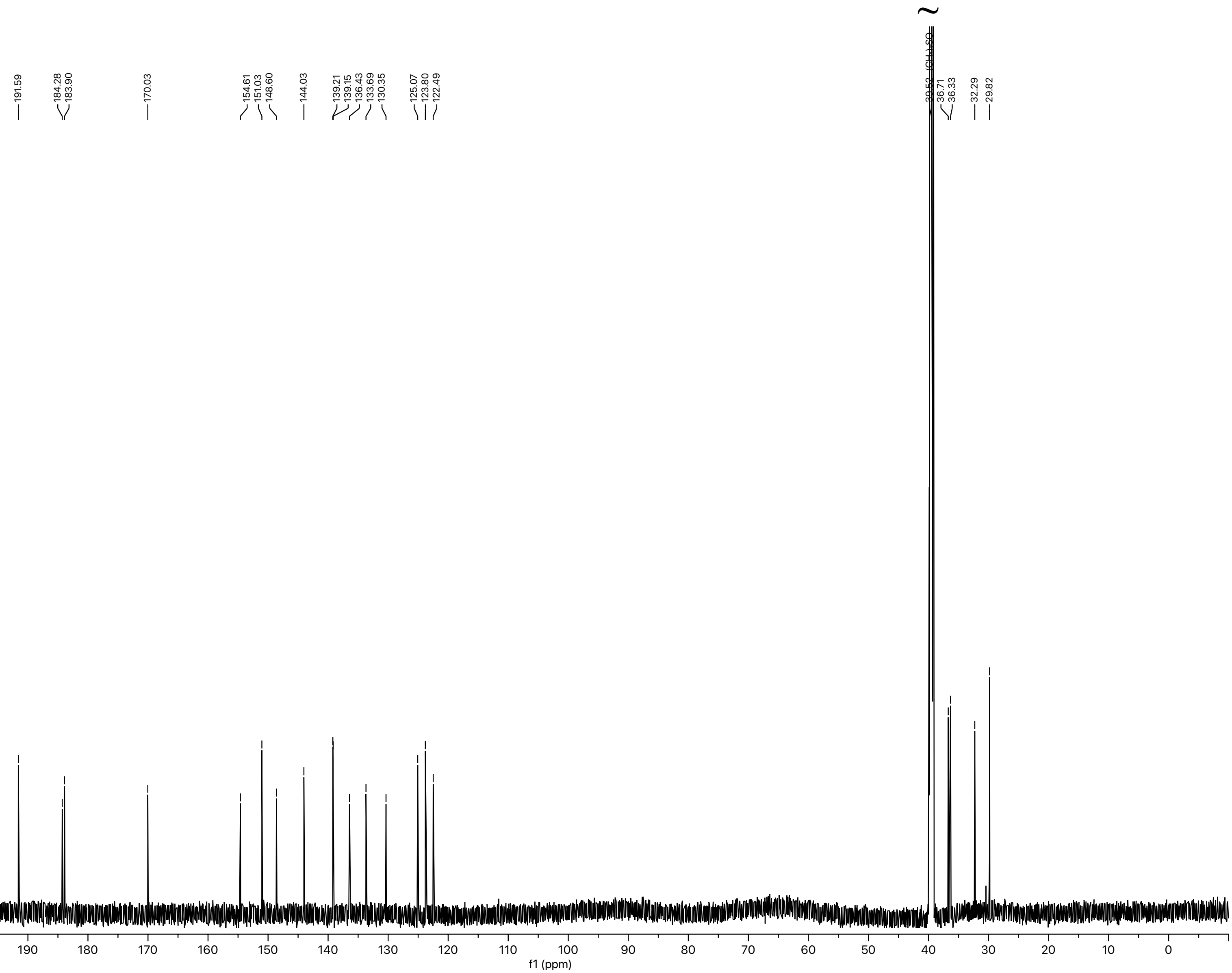

Part 4: Xestoquinolide B

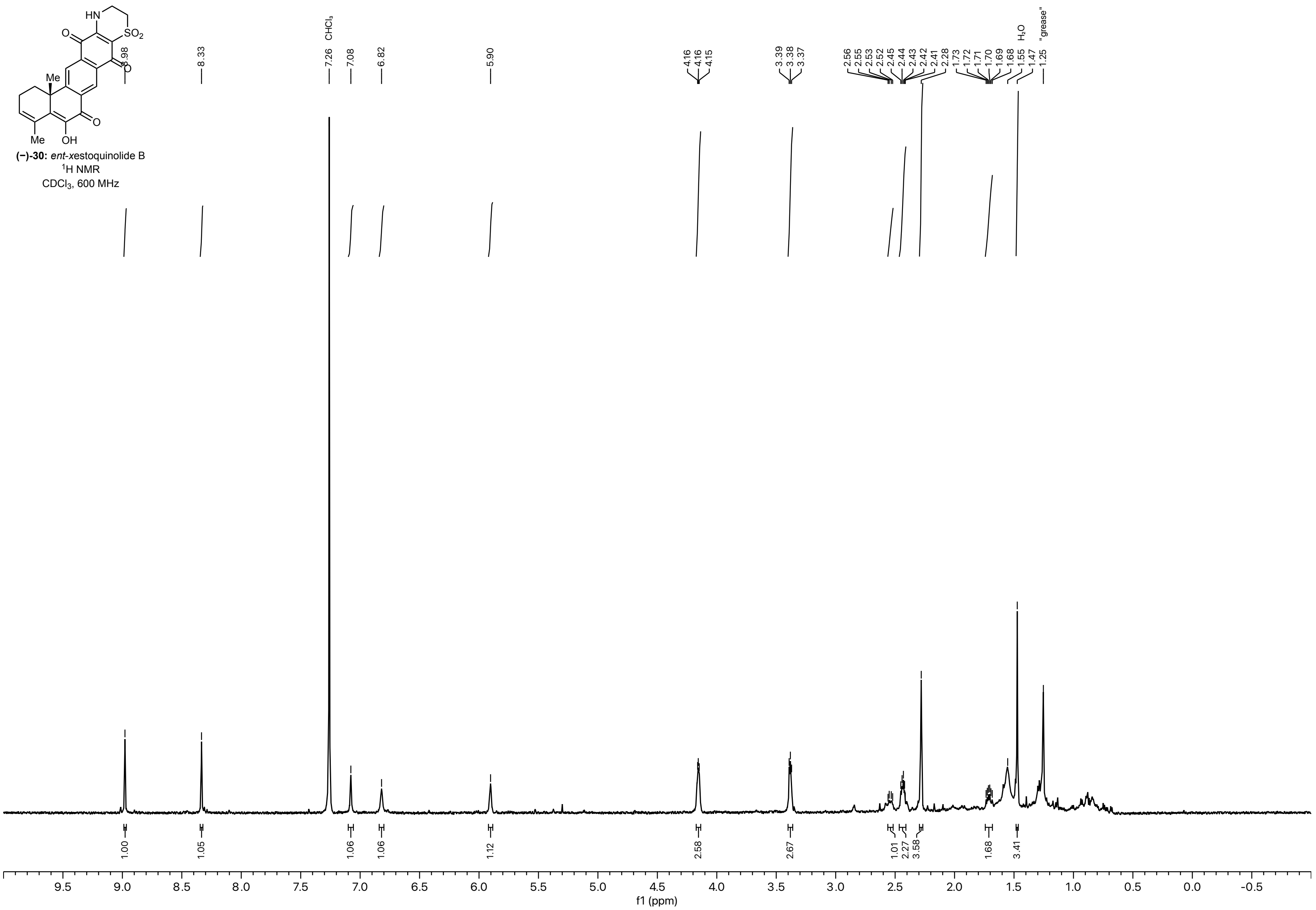

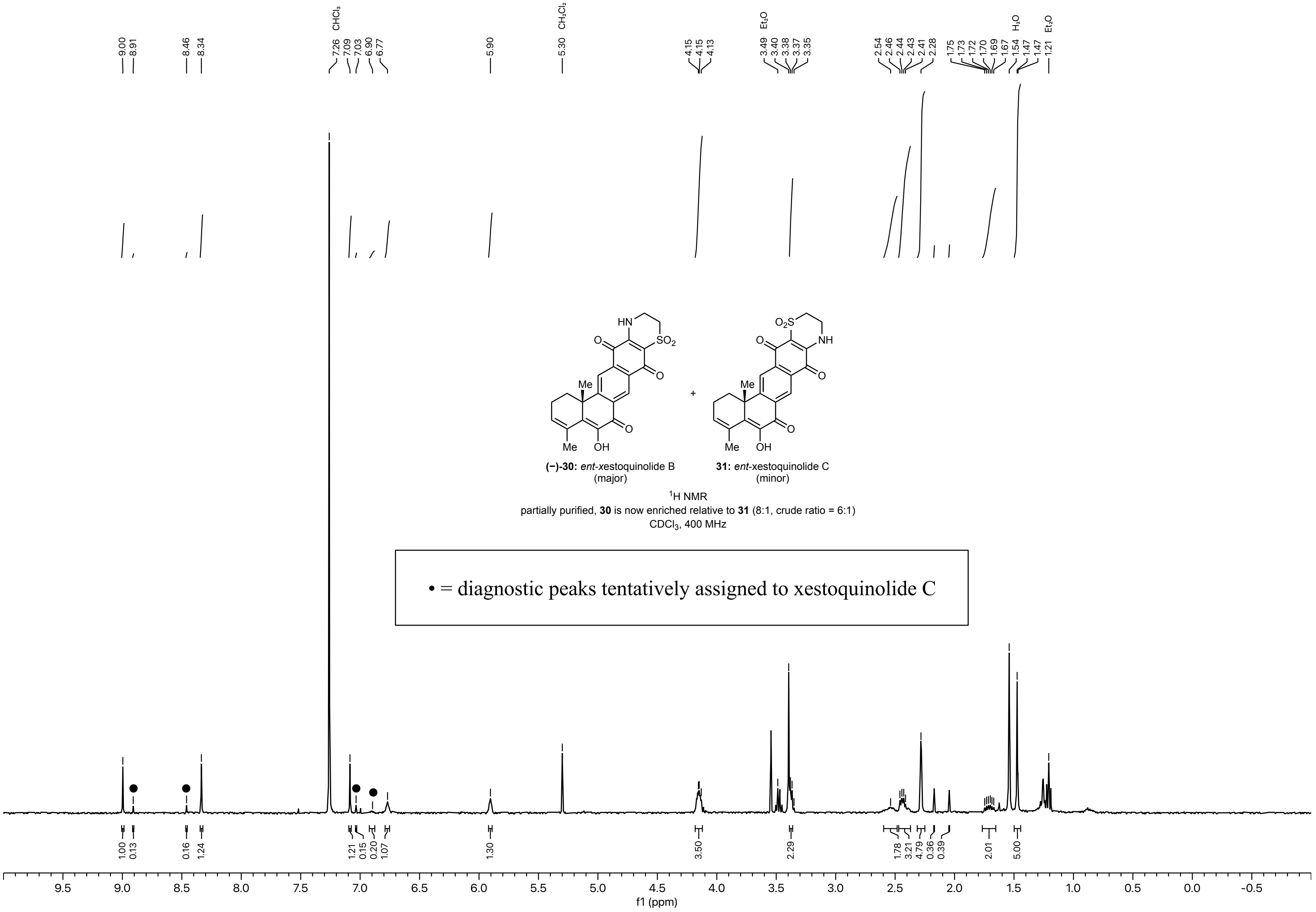
9.00 8.91 8.46 8.34 7.26 CHCl3 7.09 7.03 6.90 6.77 5.90 5.30 CH2Cl2 4.15 4.15 4.13 3.49 Et2O 3.40 3.38 3.37 3.35 2.54 2.46 2.44 2.43 2.41 2.28 1.75 1.73 1.72 1.70 1.69 1.67 1.54 H2O 1.47 1.47 1.21 Et2O
HN
SO2
O2S
NH
O
Me
OH
+
(−)-30: ent-xestoquinolide B
(major)
31: ent-xestoquinolide C
(minor)
1H NMR
partially purified, 30 is now enriched relative to 31 (8:1, crude ratio = 6:1)
CDCl3, 400 MHz
• = diagnostic peaks tentatively assigned to xestoquinolide C
1.00 0.13 0.16 1.24 1.21 0.15 0.20 1.07 1.30 3.50 2.29 1.78 3.21 4.79 0.36 0.39 2.01 5.00
9.5 9.0 8.5 8.0 7.5 7.0 6.5 6.0 5.5 5.0 4.5 4.0 3.5 3.0 2.5 2.0 1.5 1.0 0.5 0.0 -0.5
f1 (ppm)

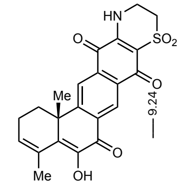

(−)-30: *ent*-xestoquinolide B
^{1}H NMR
$(CD_3)_2SO$, 600 MHz

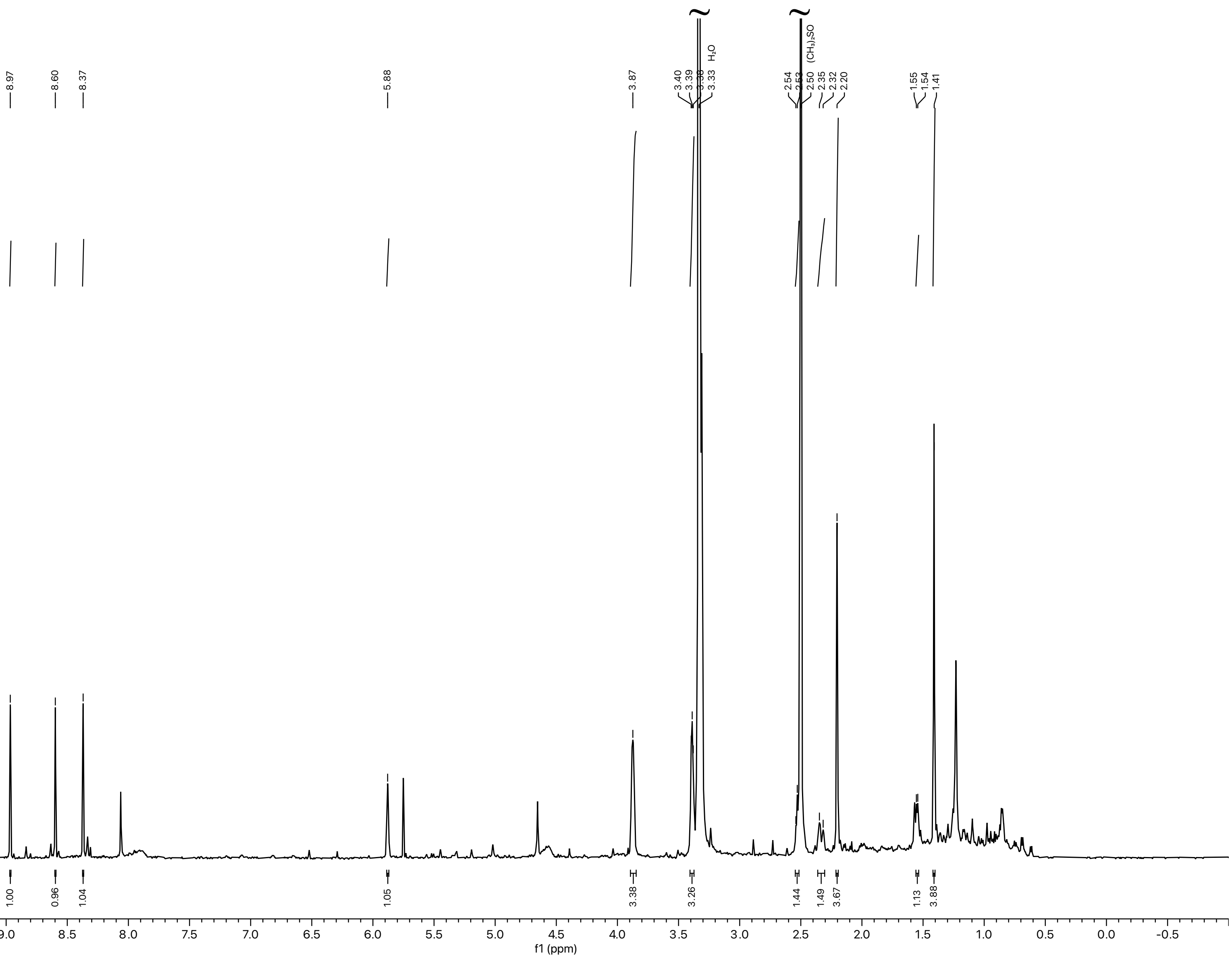

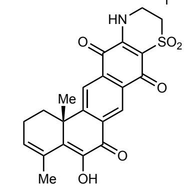

(−)-30: *ent*-xestoquinolide B
^{1}H NMR
$(CD_3)_2SO$, 600 MHz

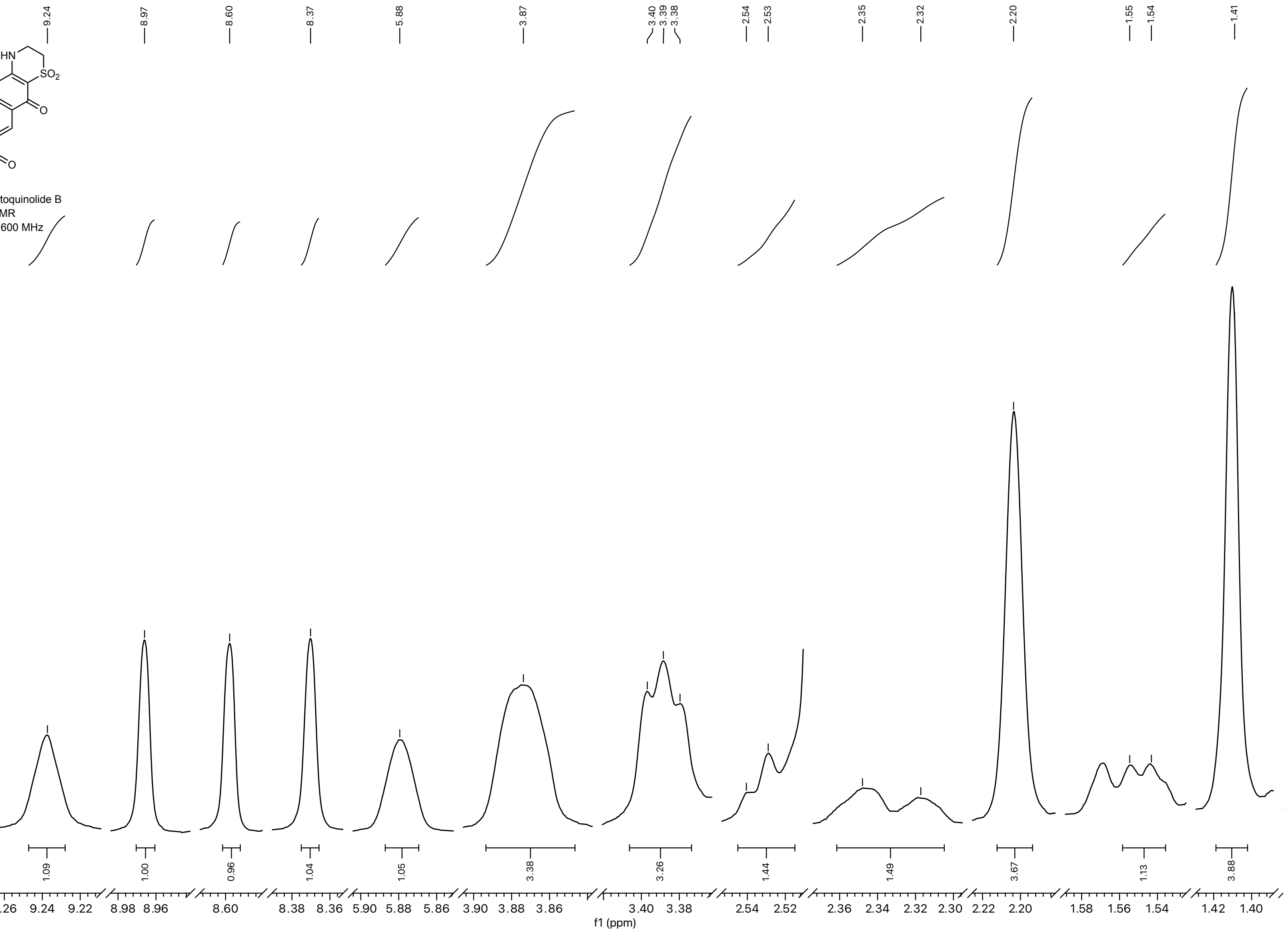

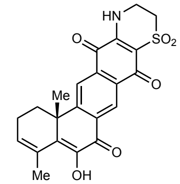

(−)-**30:** *ent*-xestoquinolide B
^{13}C NMR
$(CD_3)_2SO$, 150 MHz

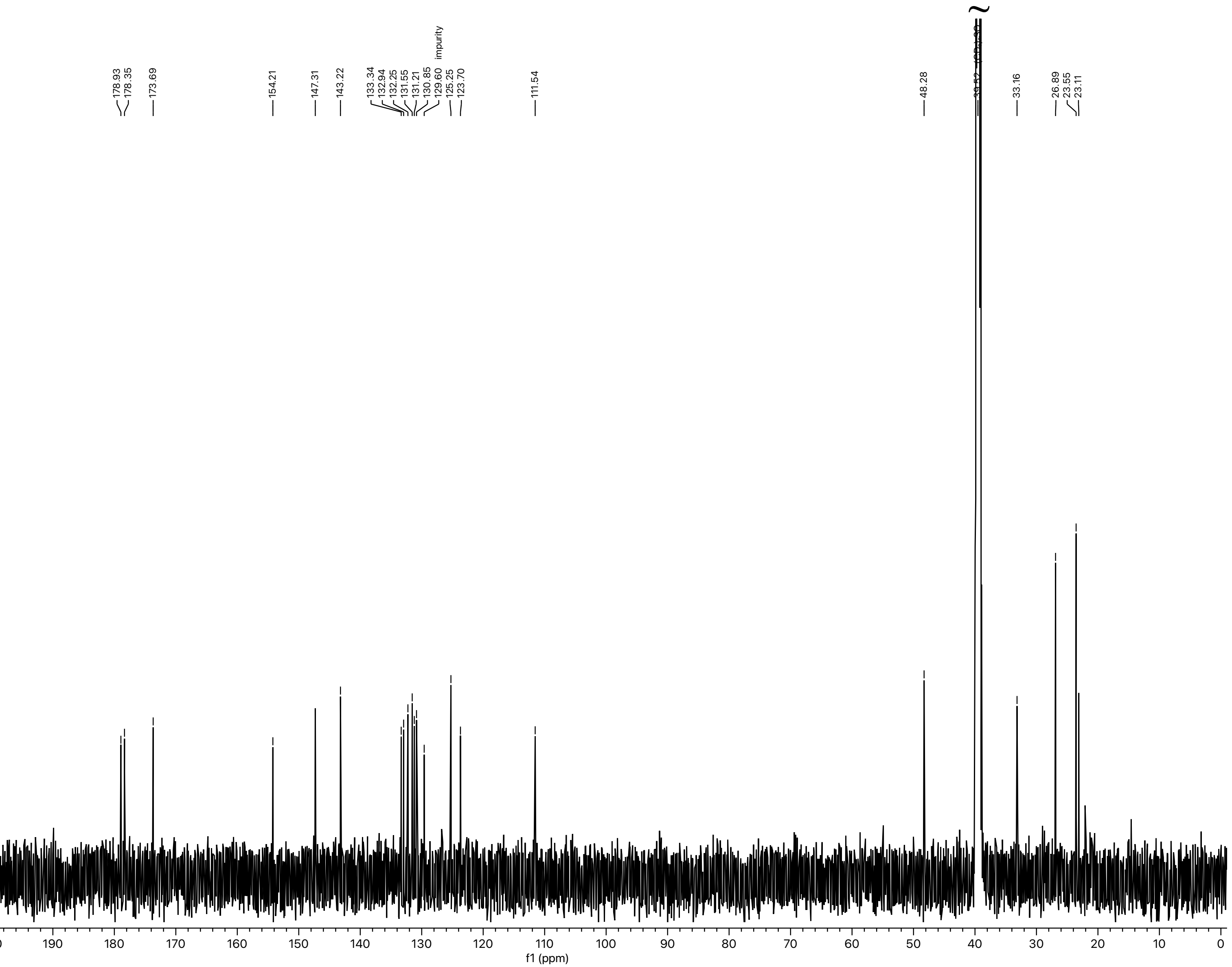

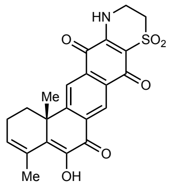

(−)-30: *ent*-xestoquinolide B
COSY
$CDCl_3$, 600 MHz

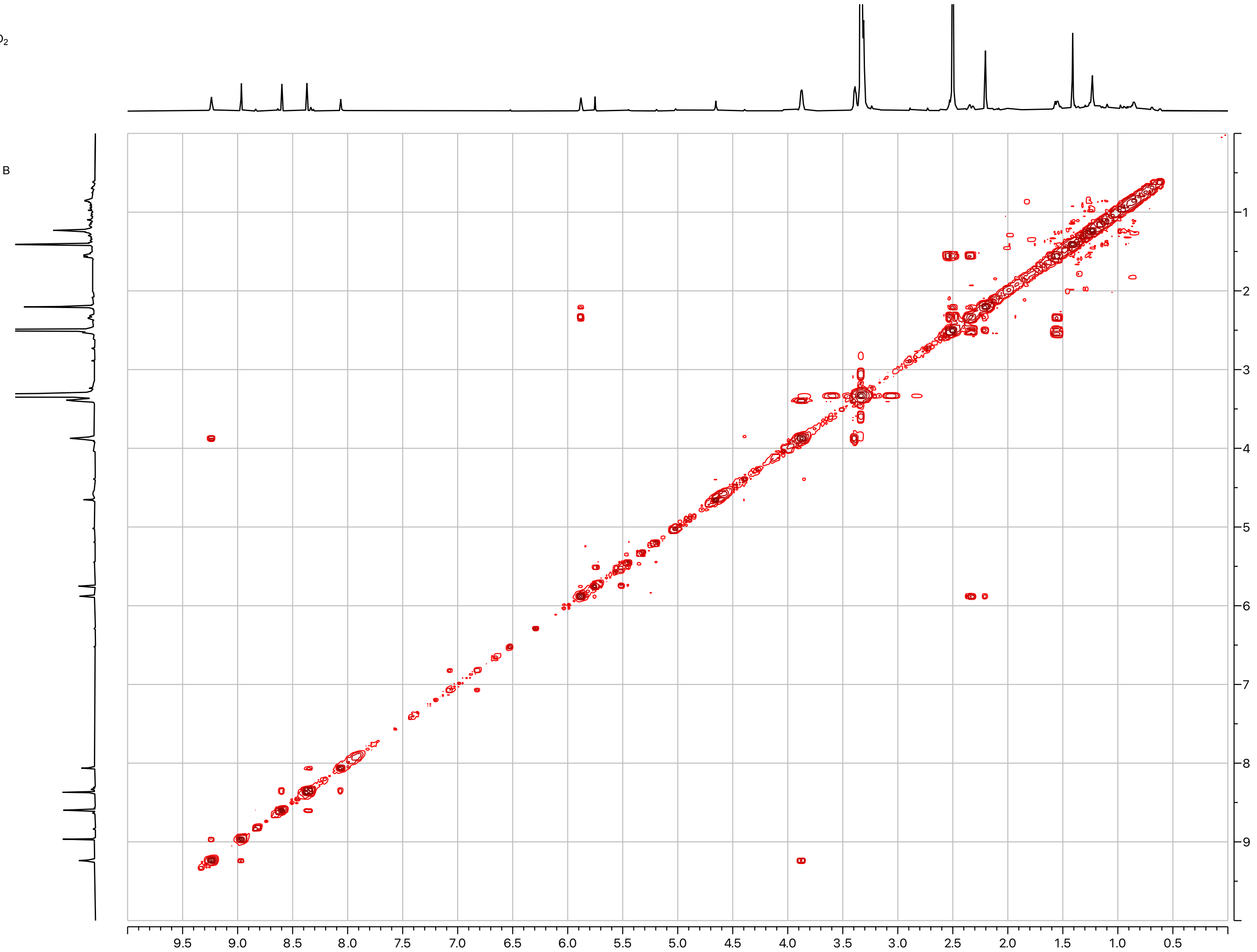

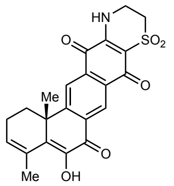

(−)-30: *ent*-xestoquinolide B
HSQC
$CDCl_3$, 600 MHz

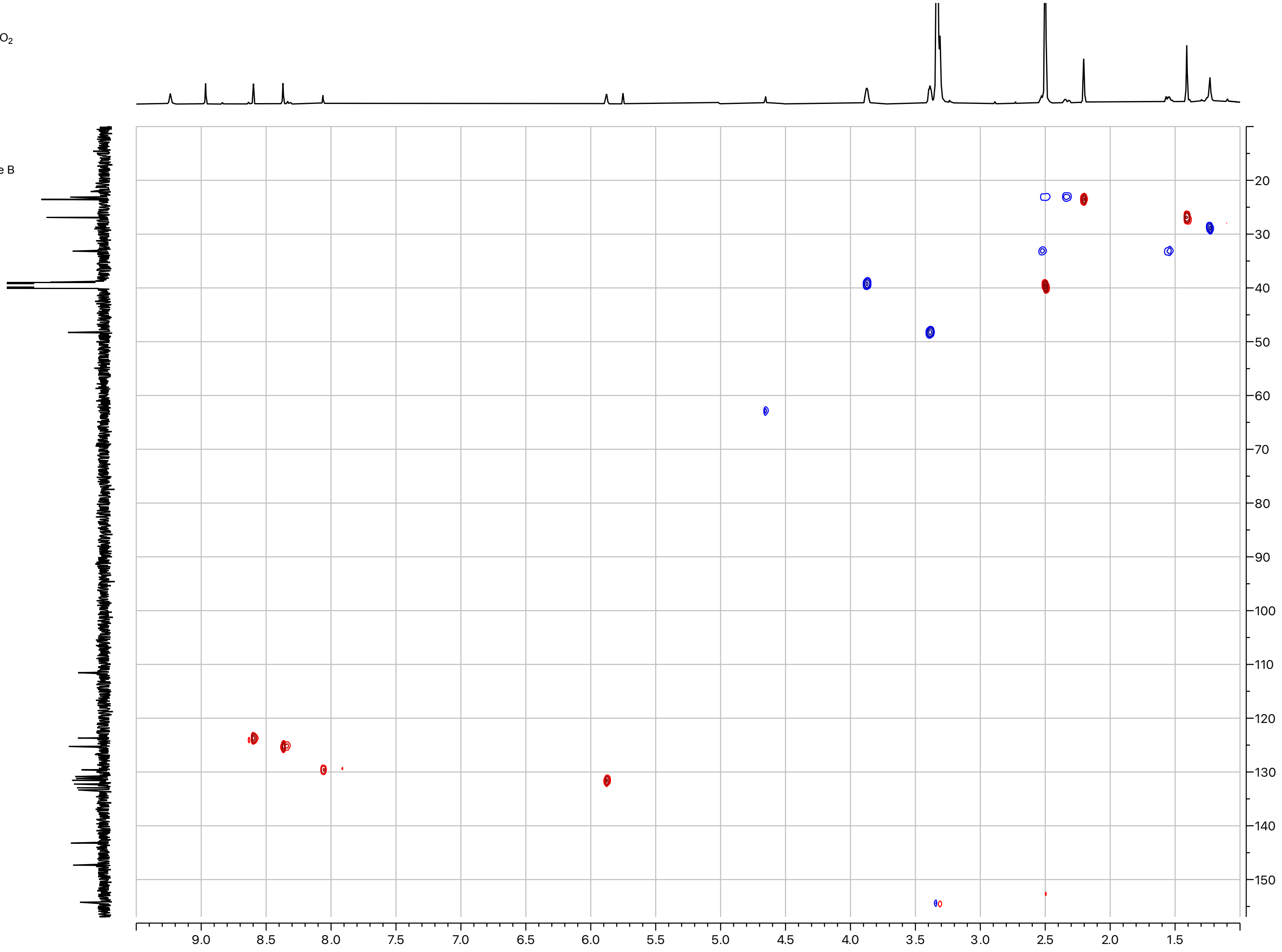

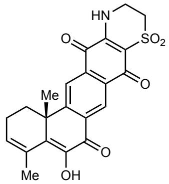

(−)-30: *ent*-xestoquinolide B
HMBC
$CDCl_3$, 600 MHz

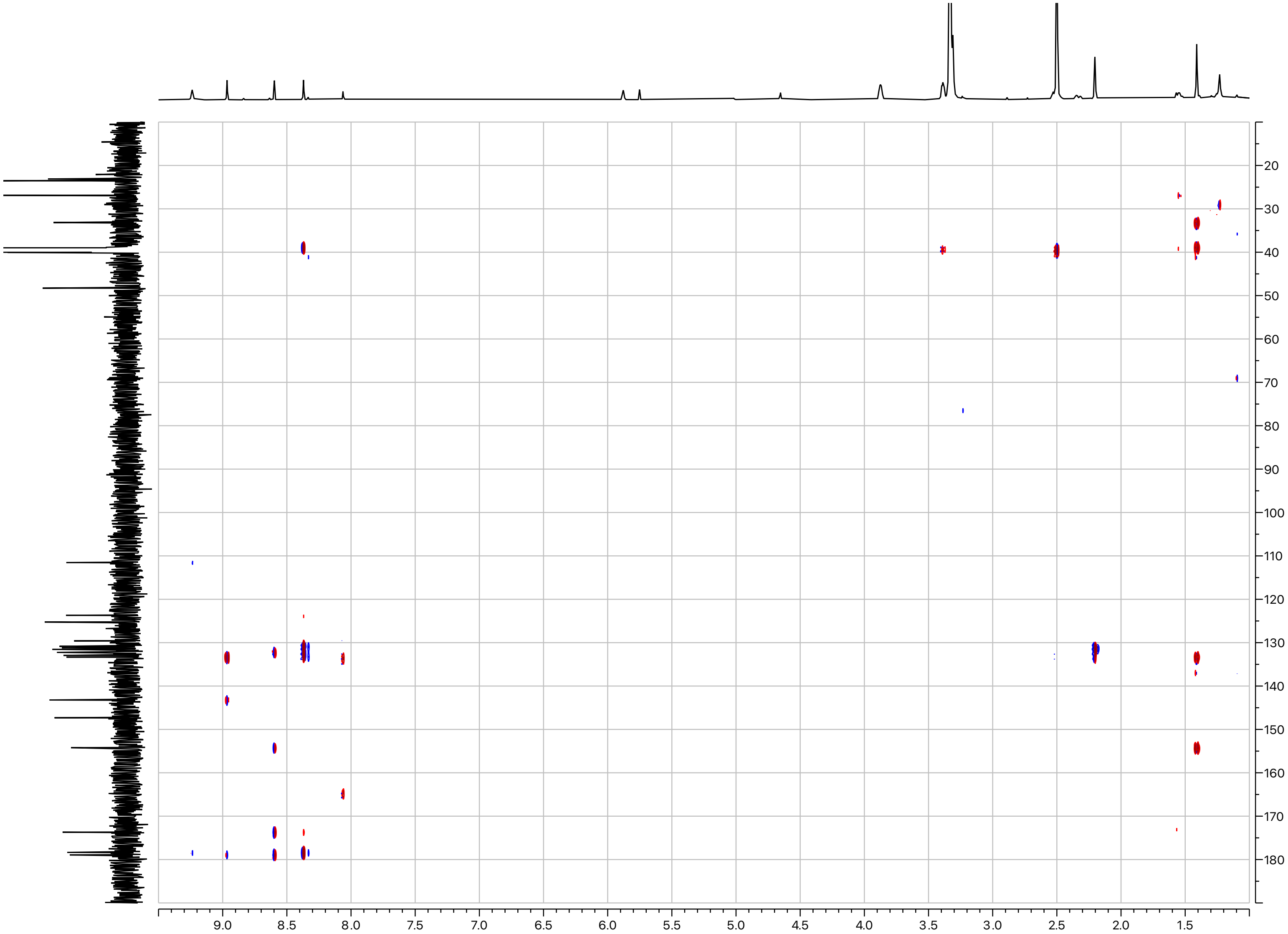

References

(1) Cortés, M.; Valderrama, J. A.; Cuellar, M.; Armstrong, V.; Preite, M. *J. Nat. Prod.* **2001**, *64*, 348. DOI: 10.1021/np0004146.
(2) Schröder, J.; Matthes, B.; Seifert, K. *Tetrahedron Lett.* **2001**, *42*, 8151. DOI: 10.1016/s0040-4039(01)01748-8.
(3) Villamizar, J.; Fuentes, J.; Tropper, E.; Orcajo, A. L.; Alonso, R. *Synth. Commun.* **2003**, *33*, 1121. DOI: 10.1081/scc-120017187.
(4) Delgado, V.; Armstrong, V.; Cortés, M.; Barrero, A. F. *J. Braz. Chem. Soc.* **2008**, *19*, 1258. DOI: 10.1590/s0103-50532008000700005.
(5) Rosales Martínez, A.; Pozo Morales, L.; Díaz Ojeda, E. *Synth. Commun.* **2019**, *49*, 2554. DOI: 10.1080/00397911.2019.1633671.
(6) Chayboun, I.; Boulifa, E.; Mansour, A. I.; Rodriguez-Serrano, F.; Carrasco, E.; Alvarez, P. J.; Chahboun, R.; Alvarez-Manzaneda, E. *J. Nat. Prod.* **2015**, *78*, 1026. DOI: 10.1021/np500975b.
(7) Harada, N.; Sugioka, T.; Uda, H.; Kuriki, T. *J. Org. Chem.* **1990**, *55*, 3158. DOI: 10.1021/jo00297a035.
(8) Kanematsu, K.; Soejima, S.; Wang, G. *Tetrahedron Lett.* **1991**, *32*, 4761. DOI: 10.1016/s0040-4039(00)92301-3.
(9) Maddaford, S. P.; Andersen, N. G.; Cristofoli, W. A.; Keay, B. A. *J. Am. Chem. Soc.* **1996**, *118*, 10766. DOI: 10.1021/ja960807k.
(10) Miyazaki, F.; Uotsu, K.; Shibasaki, M. *Tetrahedron* **1998**, *54*, 13073. DOI: 10.1016/s0040-4020(98)00797-2.
(11) Sutherland, H. S.; Higgs, K. C.; Taylor, N. J.; Rodrigo, R. *Tetrahedron* **2001**, *57*, 309. DOI: 10.1016/s0040-4020(00)00938-8.
(12) Lu, X.-L.; Qiu, Y.; Yang, B.; He, H.; Gao, S. *Chem. Sci.* **2021**, *12*, 4747. DOI: 10.1039/d0sc07089k.
(13) Harada, N.; Sugioka, T.; Ando, Y.; Uda, H.; Kuriki, T. *J. Am. Chem. Soc.* **1988**, *110*, 8483. DOI: 10.1021/ja00233a026.
(14) Kojima, A.; Takemoto, T.; Sodeoka, M.; Shibasaki, M. *J. Org. Chem.* **1996**, *61*, 4876. DOI: 10.1021/jo960773z.
(15) Sutherland, H. S.; Souza, F. E. S.; Rodrigo, R. G. A. *J. Org. Chem.* **2001**, *66*, 3639. DOI: 10.1021/jo010112o.
(16) Kienzler, M. A.; Suseno, S.; Trauner, D. *J. Am. Chem. Soc.* **2008**, *130*, 8604. DOI: 10.1021/ja8035042.
(17) Goswami, S.; Harada, K.; El‐Mansy, M. F.; Lingampally, R.; Carter, R. G. *Angew. Chem., Int. Ed.* **2018**, *57*, 9117. DOI: 10.1002/anie.201805370.
(18) Schmitz, F. J.; Bloor, S. J. *J. Org. Chem.* **1988**, *53*, 3922. DOI: 10.1021/jo00252a007.
(19) Rosenau, C. P.; Jelier, B. J.; Gossert, A. D.; Togni, A. *Angew. Chem., Int. Ed.* **2018**, *57*, 9528. DOI: 10.1002/anie.201802620.
(20) Budwitz, J. E.; Newton, C. G. *Org. Synth.* **2023**, *100*, 159. DOI: 10.15227/orgsyn.100.0159.
(21) Easterfield, H.; Aston, B. C. *Trans. & Proc. New Zealand Inst.* **1903**, *36*, 483.
(22) McKee, N. A.; Bradford, S.; Parish, D. E.; Neely, W. C.; Parish, E. J. *Austin J. Bioorg. & Org. Chem.* **2014**, *1*.
(23) Dissanayake, I.; Hart, J. D.; Becroft, E. C.; Sumby, C. J.; Newton, C. G. *J. Am. Chem. Soc.* **2020**, *142*, 13328. DOI: 10.1021/jacs.0c06306.
(24) Kofron, W. G.; Baclawski, L. M. *J. Org. Chem.* **1976**, *41*, 1879. DOI: 10.1021/jo00872a047.
(25) Lee, H.; Harvey, R. G. *J. Org. Chem.* **1988**, *53*, 4587. DOI: 10.1021/jo00254a035.
(26) Salihila, J.; Silva, L.; Pérez del Pulgar, H.; Quílez Molina, A.; González-Coloma, A.; Olmeda, A. S.; Quílez del Moral, J. F.; Barrero, A. F. *J. Org. Chem.* **2019**, *84*, 6886. DOI: 10.1021/acs.joc.9b00704.
(27) Kurata, K.; Taniguchi, K.; Suzuki, M. *Phytochemistry* **1996**, *41*, 749. DOI: 10.1016/0031-9422(95)00651-6.
(28) Winder, P. L.; Baker, H. L.; Linley, P.; Guzmán, E. A.; Pomponi, S. A.; Cristina Diaz, M.; Reed, J. K.; Wright, A. E. *Bioorg. Med. Chem.* **2011**, *19*, 6599. DOI: 10.1016/j.bmc.2011.09.026.
(29) Longeon, A.; Copp, B. R.; Roué, M.; Dubois, J.; Valentin, A.; Petek, S.; Debitus, C.; Bourguet-Kondracki, M.-L. *Bioorg. Med. Chem.* **2010**, *18*, 6006. DOI: 10.1016/j.bmc.2010.06.066.
(30) Nakamura, H.; Kobayashi, J. i.; Kobayashi, M.; Ohizumi, Y.; Hirata, Y. *Chem. Lett.* **1985**, *14*, 713. DOI: 10.1246/cl.1985.713.
(31) Roll, D. M.; Scheuer, P. J.; Matsumoto, G. K.; Clardy, J. *J. Am. Chem. Soc.* **1983**, *105*, 6177. DOI: 10.1021/ja00357a049.
(32) Wang, J.; Bourguet-Kondracki, M.-L.; Longeon, A.; Dubois, J.; Valentin, A.; Copp, B. R. *Bioorg. Med. Chem. Lett.* **2011**, *21*, 1261. DOI: 10.1016/j.bmcl.2010.12.056.
(33) Alvi, K. A.; Rodriguez, J.; Diaz, M. C.; Moretti, R.; Wilhelm, R. S.; Lee, R. H.; Slate, D. L.; Crews, P. *J. Org. Chem.* **1993**, *58*, 4871. DOI: 10.1021/jo00070a023.
(34) Scheuer, P. J.; Zhu, Y.; Y. Yoshida, W.; Kelly-Borges, M. *Heterocycles* **1998**, *49*, 355. DOI: 10.3987/com-98-s43.

(35) Cao, S.; Foster, C.; Brisson, M.; Lazo, J. S.; Kingston, D. G. I. *Bioorg. Med. Chem.* **2005**, *13*, 999. DOI: 10.1016/j.bmc.2004.11.039.
(36) *CrysAlisPro 1.171.38.43d (Rigaku Oxford Diffraction, **2015**)*.
(37) (a) Sheldrick, G. M. *Acta Crystallogr. A Found. Adv.* **2015**, *71*, 3. DOI: 10.1107/s2053273314026370. (b) Sheldrick, G. M. *Acta Crystallogr., Sect. A: Found. Crystallogr.* **2008**, *64*, 112. DOI: 10.1107/s0108767307043930.
(38) Sheldrick, G. M. *Acta Crystallogr. C Struct. Chem.* **2015**, *71*, 3. DOI: 10.1107/s2053229614024218.
(39) Barbour, L. J. *J. Supramol. Chem.* **2001**, *1*, 189. DOI: 10.1016/s1472-7862(02)00030-8.
(40) Dolomanov, O. V.; Bourhis, L. J.; Gildea, R. J.; Howard, J. A. K.; Puschmann, H. *J. Appl. Crystallogr.* **2009**, *42*, 339. DOI: 10.1107/s0021889808042726.
